# Supplementary material for: Enantioselective acyl-trifluoromethylation of olefins by bulky thiazolium carbene catalysis
Source: Nat Commun. 2025 Apr 7;16:3293. doi: 10.1038/s41467-025-58423-z (PMC11977197; doi:10.1038/s41467-025-58423-z)
Supplement: Supplementary file 1 — Supplementary Information [file 41467_2025_58423_MOESM1_ESM.pdf]

Supplementary Information

Enantioselective Acyl-Trifluoromethylation of Olefins by Bulky Thiazolium Carbene Catalysis

Sripati Jana,[1] Matthew D. Wodrich,[2] Nicolai Cramer[1*]

1. Laboratory of Asymmetric Catalysis and Synthesis, Institute of Chemical Sciences and Engineering, Ecole Polytechnique Fédérale de Lausanne (EPFL), 1015 Lausanne, Switzerland.

2. Computational Molecular Design Laboratory, Institute of Chemical Sciences and Engineering, Ecole Polytechnique Fédérale de Lausanne (EPFL), 1015 Lausanne, Switzerland.

Correspondence to: nicolai.cramer@epfl.ch

Table of content

General information S2

Synthesis of chiral thiazolium salts S3

- Procedure for the Synthesis of Synthesis of 2,6-bis(1-aryllvinyl)aniline derivatives (GP 1) S3
- Procedure for the Synthesis of Synthesis of 2,6-bis(1-aryllvinyl)aniline derivatives (GP 2) S4
- Procedure for the asymmetric hydrogenation of alkenyl anilines (GP 3) S5
- General procedure for the synthesis of chiral thiazolium salts (GP 4) S6
- Modified procedure for the synthesis of chiral thiazolium salts (GP 5) S7

Physical data of chiral thiazolium salts S8

Starting material synthesis S14

Reaction Optimization for Asymmetric Acyl-Trifluoromethylation of Olefins S17

- Table S1: Catalyst screening S17
- Table S2: Solvent screening S18
- Table S3: Base screening S18
- Table S4: Temperature and time screening S19
- Table S5: Stoichiometry screening S19
- Table S6: Concentration screening S19

Catalysis procedures S20

- Procedure for the thiazolium carbene-catalyzed acyl-trifluoromethylation reaction of olefins(GP 6) S20
- Procedure for the thiazolium carbene-catalyzed asymmetric acyl-trifluoromethylation reaction (GP 7) S20

Physical data S21

Scale up experiment S57

Control experiments S57

X-ray Crystallography data of **3d** S58

- Structure Quality Indicators S60

Computational Details. S62

NMR spectra S64

References S150

General information

All reactions were carried out under a nitrogen atmosphere in flame-dried glassware with magnetic stirring, either inside a MBRAUN glove box workstation or using Schlenk techniques, unless otherwise indicated. Dichloromethane (DCM), diethyl ether, toluene, acetonitrile (MeCN), and tetrahydrofuran (THF) were purified using an Innovative Technology Solvent Delivery System, degassed via the freeze-pump-thaw technique, and stored over 4 Å molecular sieves in a nitrogen-filled glove box. Reagents and solvents were purchased from Sigma Aldrich/Merck, Acros, Alfa Aesar, Abcr, or TCI, and used as received. Ethyl acetate (EtOAc), dichloromethane, and *n*-pentane, used for filtration, transfers, chromatography, and recrystallizations, were purchased from commercial sources and distilled before use. *tert*-Butyl methyl ether (TBME), used for enantioselective catalysis, was purchased from Thermo Scientific Chemicals and stored directly in the glove box.

Flash chromatography was performed with Silicycle silica gel 60 (40-60 µm, 230–400 mesh). Analytical thin-layer chromatography (TLC) was performed with commercial glass plates coated with 0.25 mm silica gel (E. Merck, Kieselgel 60 F254). Compounds were visualized either under UV light at 254 nm or by dipping the plates in an aqueous potassium permanganate solution followed by heating.

Proton nuclear magnetic resonance (^1H NMR) spectra were acquired on a Bruker AVANCE NEO-400 (400 MHz), AVANCE III-400 (400 MHz), or AVANCE II-800 (800 MHz) spectrometer at 298 K unless otherwise noted. Chemical shifts (*δ*) are reported in parts per million (ppm) relative to the residual signal of deuterated chloroform ($CDCl_3$, δ 7.26 ppm). Splitting patterns are designated as follows: bs (broad singlet), s (singlet), d (doublet), t (triplet), q (quartet), hept (heptet), m (multiplet), and br (broad). Proton-decoupled carbon-13 nuclear magnetic resonance ($^{13}C\{^1H\}$ NMR) spectra were acquired on a Bruker AVANCE III-400 (101 MHz) or AVANCE II-800 (201 MHz) spectrometer at 298 K unless otherwise noted. The assignment of primary (CH_3), secondary (CH_2), tertiary (CH), and quaternary (Cquat) carbon atoms was made using DEPT-135 spectra. Chemical shifts are reported relative to $CDCl_3$ (δ 77.16 ppm). Fluorine-19 NMR (^{19}F NMR) spectra were acquired on a Bruker AVANCE III-400 (376 MHz) spectrometer at 298 K unless otherwise noted. Chemical shifts are reported relative to $CFCl_3$ (δ_F = 0 ppm) signal. $CFCl_3$ used as references to calibrate the chemical shifts values.

Infrared (IR) spectra were recorded on an Alpha-P Bruker FT-IR spectrometer, with absorbance frequencies reported in reciprocal centimeters (cm^{-1}).

High-resolution mass spectrometry (HRMS) data were acquired on an Agilent LC-MS TOF (Multimode: ESI + APCI) or an LTQ Orbitrap FTMS instrument (LTQ Orbitrap Elite FTMS, Thermo Scientific, Bremen, Germany) equipped with an Ion Max APPI ionization source with a VUV Kr lamp (Syagen, CA, USA). Samples were diluted in DCM or THF and directly infused into the mass spectrometer. FT-MS spectra were recorded in the 100–1000 *m/z* range in positive mode with a resolution set to 120,000. HRMS analyses were performed by Dr. L. Menin's team at the mass spectrometry platform of ISIC at EPF Lausanne.

Optical rotations were measured on a Polartronic M polarimeter using a 10.0 cm cell with a Na 589 nm filter.

The enantiomeric ratio of compounds was measured on an Agilent HPLC 1260 Infinity or Shimadzu Prominence UFLC XR system using a chiral CHIRALPAK column.

X-ray analysis was performed by Dr. R. Scopelliti at EPFL, Lausanne.

Synthesis of chiral thiazolium salts

General procedure for the Synthesis of 2,6-bis(1-aryllvinyl)aniline derivatives (GP 1)

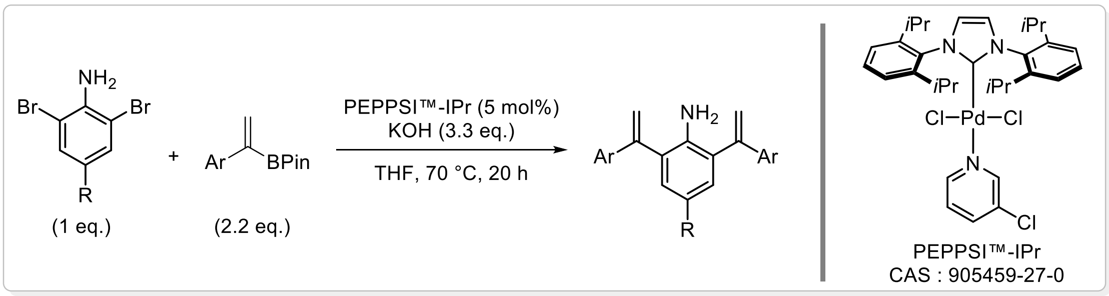

Based on the previously reported literature:[1,2] In a Schlenk tube, 2,6-dibromo-substituted aniline (1 eq.), PEPPSI™-IPr (5 mol%), and powdered KOH (2.2 eq.) were dissolved in THF (16.5 mL/mmol). To this mixture, a solution of boronate (2.2 eq.) in THF (3.5 mL/mmol) was added. The mixture was then degassed using the freeze-pump-thaw method and stirred at 70 °C for 12 hours. Following this, an additional portion of KOH (1.1 eq.) was added, and the mixture was further heated for 8 hours. The reaction mixture was then passed through a silica pad, washed with ethylacetate, and concentrated under reduced pressure. Finally, the residue was purified by silica gel column chromatography (*n*-pentane : ethylacetate) to yield the desired product.

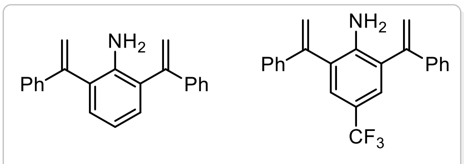

Compounds **9a** and **9b** have been previously reported in the literature.[2] They were synthesized according to the general procedure 1 (**GP 1**). The spectral data are in accordance with previous literature.[2]

2,6-Bis(1-(3,5-dimethylphenyl)vinyl)-4-(trifluoromethyl)aniline (9c)

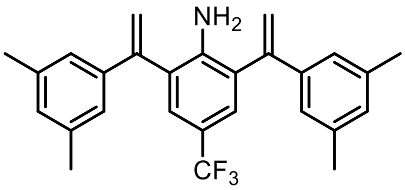

The title compound was synthesized on a 2.0 mmol scale according to the general procedure 1 (GP 1) and was obtained as a viscous yellow oil after silica gel column chromatography (*n*-pentane : ethylacetate 60:1). Yield: 69% (581 mg). $\mathbf{R_f}$ = 0.35 (*n*-pentane : EtOAc 60:1).

^{1}H NMR (400 MHz, Chloroform-*d*): δ = 7.40 (s, 2H), 6.97 – 6.93 (m, 6H), 5.80 (d, *J* = 1.4 Hz, 2H), 5.37 (d, *J* = 1.4 Hz, 2H), 3.12 (bs, 2H), 2.28 (s, 12H) ppm.

^{13}C NMR (101 MHz, Chloroform-*d*): δ = 146.5, 144.6, 138.7, 138.2, 130.1, 127.1 (q, *J* = 3.7 Hz), 127.1, 124.9 (d, *J* = 270.8 Hz), 124.2, 119.1 (q, *J* = 32.5 Hz), 117.0, 21.3 ppm.

^{19}F NMR (376 MHz, Chloroform-*d*): δ = -61.38 ppm.

HRMS (ESI/QTOF): *m/z*: $[M + H]^+$ Calcd for $C_{27}H_{27}F_3N^+$ 422.2090; Found 422.2085.

IR (ATR): 1618, 1597, 1303, 1111, 900, 852 cms^{-1}.

2,6-Bis(1-(3,5-di-*tert*-butylphenyl)vinyl)-4-(trifluoromethyl)aniline (9d)

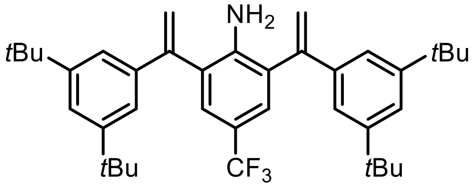

The title compound was synthesized on a 2.0 mmol scale according to the general procedure 1 (GP 1) and was obtained as a viscous yellow oil after silica gel column chromatography (*n*-pentane : ethylacetate 80:1). Yield: 57% (671 mg). $\mathbf{R_f}$ = 0.50 (*n*-pentane : EtOAc 80:1).

^{1}H NMR (400 MHz, Chloroform-*d*): δ = 7.38 (t, *J* = 1.8 Hz, 2H), 7.35 – 7.34 (m, 2H), 7.21 (d, *J* = 1.8 Hz, 4H), 5.85 (d, *J* = 1.3 Hz, 2H), 5.38 (d, *J* = 1.4 Hz, 2H), 5.04 (bs, 2H), 1.28 (s, 36H) ppm.

^{13}C NMR (101 MHz, Chloroform-*d*): δ = 151.0, 147.0, 144.8, 138.2, 127.01, 127.00 (q, *J* = 4.0 Hz), 124.9 (q, *J* = 270.8 Hz), 122.5, 120.9, 118.8 (q, *J* = 32.4 Hz), 116.4, 34.8, 31.4 ppm.

^{19}F NMR (376 MHz, Chloroform-*d*): δ = -61.49 ppm.

HRMS (ESI/QTOF): *m/z*: $[M + H]^+$ Calcd for $C_{39}H_{51}F_3N^+$ 590.3968; Found 590.3964.

IR (ATR): 2962, 1619, 1590, 1361, 1305, 1114, 879 cm^{-1}.

General procedure for the Synthesis of Synthesis of 2,6-bis(1-aryllvinyl)aniline derivatives (GP 2)

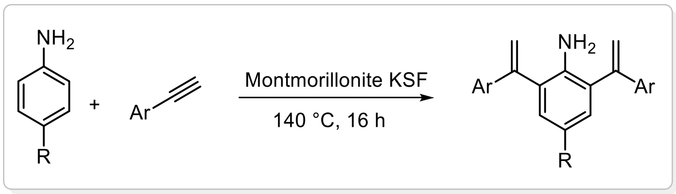

Based on the previously reported literature:[3] A 25 mL microwave tube equipped with a stirring bar was charged with para-substituted aniline (1 eq.), montmorillonite KSF (100 mg/mmol), and phenylacetylene (2.1 eq.) under a nitrogen atmosphere. The tube was then heated to 150 °C and stirred vigorously for 16 hours. After allowing the reaction to cool to room temperature, the mixture was diluted with ethylacetate and filtered. The solid residue was washed with ethylacetate, and the solvent was subsequently removed from the filtrate under reduced pressure. The resulting residue was purified by silica gel column chromatography (*n*-pentane: ethyl acetate) to yield the desired product.

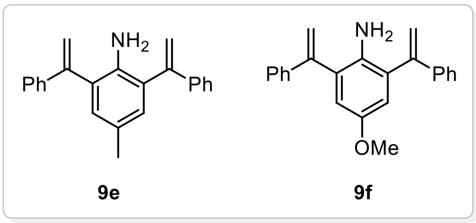

Compounds **9e** and **9f** have been previously reported in the literature.[3] They were synthesized according to the general procedure 2 (**GP 2**). The spectral data are in accordance with previous literature.[3]

General procedure for the asymmetric hydrogenation of alkenyl anilines (GP 3)

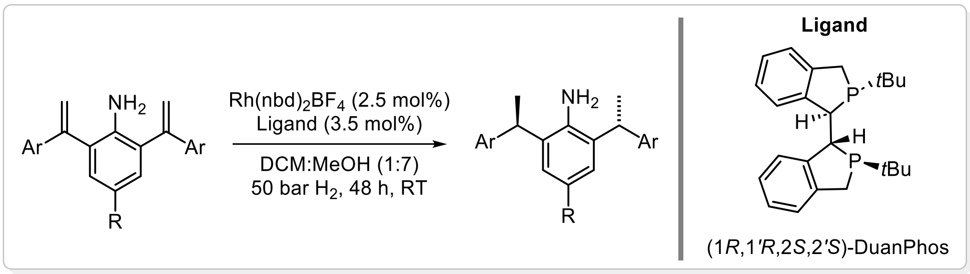

C2-Symmetric chiral anilines were synthesized according to a reported procedure.[1,2] In a microwave tube, $[Rh(nbd)_2]BF_4$ (2.5 mol%) and (1*R*,1'*R*,2*S*,2'*S*)-DuanPhos (3.5 mol%) were dissolved in DCM under a nitrogen atmosphere and stirred for 20 minutes. A solution of alkenyl aniline (1 eq.) in DCM was then added, followed by the addition of MeOH (MeOH/DCM ratio: >7/1, 0.25 M). The microwave tube was subsequently transferred to an autoclave, where the reactor was purged three times with H_2. The reactor was then pressurized to 50 bar H_2, and the mixture was stirred at ambient temperature for 48 hours. The reaction mixture was concentrated, and the resulting residue was purified by silica gel column chromatography (*n*-pentane: ethylacetate) to yield the desired chiral aniline.

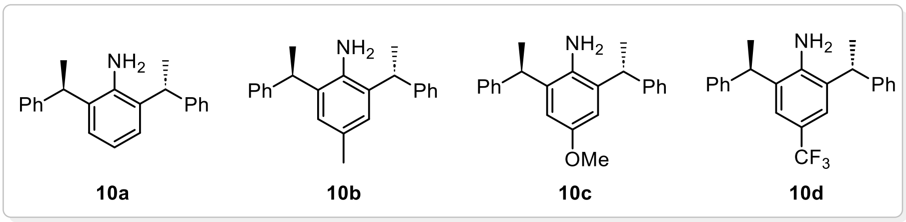

Compounds **10a** – **10d** have been previously reported in the literature.[1,2] They were synthesized according to the general procedure 3 (**GP 3**). The spectral data are in accordance with previous literature.[2]

2,6-Bis((*R*)-1-(3,5-dimethylphenyl)ethyl)-4-(trifluoromethyl)aniline (10e)

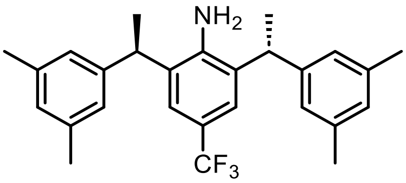

The title compound was synthesized on a 3.0 mmol scale according to the general procedure 3 (**GP 3**) and was obtained as a viscous pink oil after silica gel column chromatography (*n*-pentane : ethylacetate 90:1). Yield: 89% (1.1 g). $\mathbf{R_f}$ = 0.45 (*n*-pentane : ethylacetate 90:1).

^{1}H NMR (400 MHz, Chloroform-*d*): δ = 7.51 (s, 2H), 6.82 (s, 2H), 6.70 (s, 4H), 3.86 (q, J = 7.1 Hz, 2H), 3.72 (bs, 2H), 2.24 (s, 12H), 1.60 (d, J = 7.1 Hz, 6H) ppm.

^{13}C NMR (101 MHz, Chloroform-*d*): δ = 145.6, 145.0, 138.5, 129.1, 128.5, 125.6 (q, J = 270.9 Hz), 125.1, 122.5 (q, J = 4.0 Hz), 119.0 (q, J = 31.9 Hz), 40.7, 22.6, 21.4 ppm.

^{19}F NMR (376 MHz, Chloroform-*d*): δ = -61.17 ppm.

HRMS (ESI/QTOF): *m/z*: $[M + H]^+$ Calcd for $C_{27}H_{31}F_3N^+$ 426.2403; Found 426.2405.

IR (ATR): 2968, 1627, 1452, 1328,1150, 1108, 781 cm^{-1}.

$[\alpha]_D^{20}$ = +10.6 (c = 0.5, $CHCl_3$).

2,6-Bis((*R*)-1-(3,5-di-*tert*-butylphenyl)ethyl)-4-(trifluoromethyl)aniline (10f)

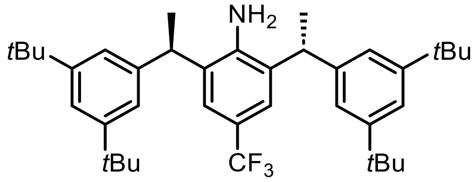

The title compound was synthesized on a 3.0 mmol scale according to the general procedure 3 (**GP 3**) and was obtained as a viscous colorless oil after silica gel column chromatography (*n*-pentane : ethylacetate 90:1). Yield: 92% (1.6 g); >99.9% ee. $\mathbf{R_f}$ = 0.48 (*n*-pentane : ethylacetate 90:1).

^{1}H NMR (400 MHz, Chloroform-*d*): δ = 7.45 (s, 2H), 7.25 (t, *J* = 1.8 Hz, 2H), 6.96 (d, *J* = 1.8 Hz, 4H), 3.97 (q, *J* = 7.1 Hz, 2H), 3.74 (bs, 2H), 1.62 (d, *J* = 7.1 Hz, 6H), 1.25 (s, 36H) ppm.

^{13}C NMR (101 MHz, Chloroform-*d*): δ = 151.2, 145.4, 143.8, 129.7, 125.6 (q, *J* = 270.7 Hz), 122.6 (q, *J* = 3.9 Hz), 121.5, 120.6, 119.3 (q, *J* = 31.9 Hz), 41.0, 34.9, 31.5, 22.3 ppm.

^{19}F NMR (376 MHz, Chloroform-*d*): δ = -61.20 ppm.

HRMS (ESI/QTOF): *m/z*: $[M + H]^+$ Calcd for $C_{39}H_{55}F_3N^+$ 594.4281; Found 594.4284.

IR (ATR): 2963, 1596, 1362, 1268, 1111, 758 cm^{-1}.

$[\boldsymbol{\alpha}]_{\boldsymbol{D}}^{\mathbf{20}}$ = +102.6 (c = 0.5, $CHCl_3$).

General procedure for the synthesis of chiral thiazolium salts (GP 4)

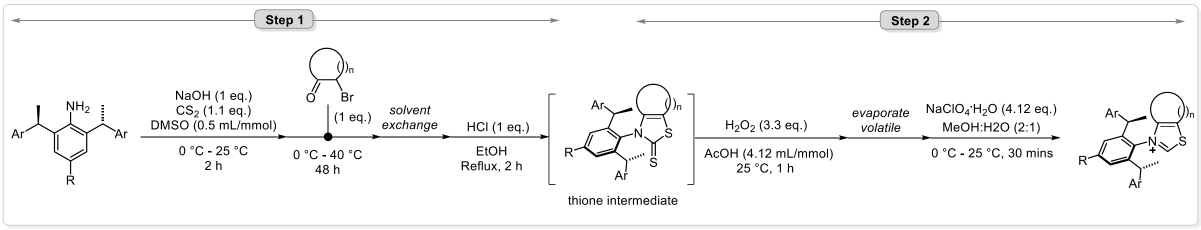

According to a slightly modified procedure previously reported in the literature by Bach[4] and Glorius.[5]

Step 1 **:** A solution of chiral aniline (1 eq.) in DMSO (0.5 mL/mmol) was treated with 20 N aqueous NaOH (1 eq.) at ambient temperature. The mixture was then cooled to 0 °C, and CS_2 (1.1 eq.) was added dropwise, causing a colour change from pale yellow to dark red. After allowing the reaction mixture to return to ambient temperature (25 °C) and stirring for 2 hours, the colour gradually shifted from dark red to yellow or orange. The mixture was then cooled to 0 °C again and stirred for 5 minutes. Subsequently, *α*-bromo ketone (1 eq.) was added dropwise (for liquid) or portion-wise (for solid) at 0 °C, and the mixture was stirred at 40 °C for 48 hours. Following this, the reaction mixture was cooled to 0 °C and quenched with water (1 mL/mmol). The mixture was extracted with ethyl acetate, and the organic fractions were dried over Na_2SO_4. The solvent was then evaporated under reduced pressure, yielding a yellow solid. This yellow solid was dissolved in EtOH (1 mL/mmol), and 37% fuming HCl (0.05 mL/mmol) was added. The resulting mixture was refluxed for 2 hours, during which the colour changed from yellow or orange to greenish-black. The reaction mixture was then cooled to room temperature, extracted with ethyl acetate, dried over Na_2SO_4, and the solvent was evaporated under reduced pressure to obtain a crude mixture of thione. This mixture was partially purified using silica gel flash column chromatography (*n*-pentane/ethyl acetate). (*Note: During purification, a mixture of unidentifiable products and the desired thione product was often encountered. Therefore, the thione's purity percentage was determined by 1H NMR before proceeding to the next step*)

Step 2 **:** Thione (1 eq.) was dissolved in acetic acid (4.5 mL/mmol), and 30% H_2O_2 (3.3 eq.) was added while cooling the reaction mixture with a water bath. The mixture was then stirred at ambient temperature (25 °C) for 1 hour. Afterward, the solvent was removed under reduced pressure, and the residue was dissolved in methanol (0.5 mL/mmol). The resulting mixture was cooled to 0 °C, and a solution of $NaClO_4 \cdot H_2O$ (4.12 eq.) in a methanol/water mixture (2:1, total volume 3.5 mL/mmol) was added slowly. After stirring for 10 minutes at 0 °C, the reaction mixture was stirred for an additional 20 minutes at ambient temperature. The reaction mixture was then diluted and extracted with DCM. The organic fractions were dried over Na_2SO_4 and evaporated under reduced pressure. The crude residue was purified by silica gel column chromatography using a DCM/MeOH mixture as the eluent, yielding pure chiral thiazolium salts.

Modified procedure for the synthesis of chiral thiazolium salts (GP 5)

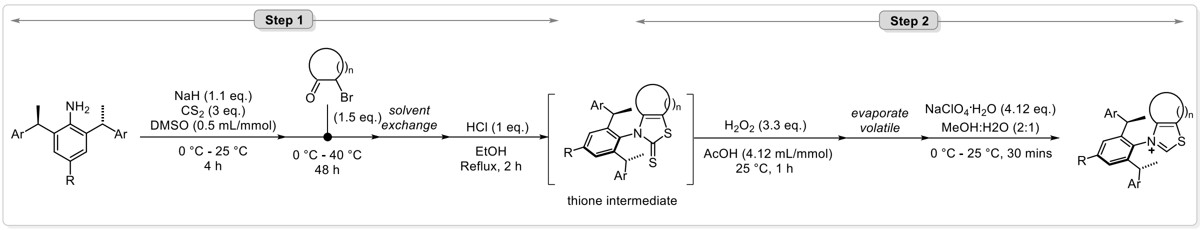

According to the slightly modified procedure previously reported in the literature by Bach[4] and Glorius.[5]

Step 1 **:** A solution of chiral aniline (1 eq.) in DMSO (0.5 mL/mmol) was treated with 60% NaH (1.1 eq.) at 0 °C. CS_2 (3 eq.) was added dropwise, resulting in a color change from pale yellow to dark red. The reaction mixture was allowed to return to ambient temperature (25 °C) and stirred for 4 hours, during which the color gradually changed from dark red to light red. The mixture was then cooled to 0 °C again and stirred for 5 minutes. After that, α-bromo ketone (1.5 eq.) was added portion-wise at 0 °C, and the mixture was stirred at 40 °C for 48 hours. Following this, the reaction mixture was cooled to 0 °C and quenched with water (1 mL/mmol). The mixture was extracted with ethylacetate, and the organic fractions were dried over Na_2SO_4. The solvent was then evaporated under reduced pressure, yielding a pale red solid. This solid was dissolved in EtOH (1 mL/mmol), and 37% fuming HCl (0.05 mL/mmol) was added. The resulting mixture was refluxed for 2 hours, during which the color changed from red to greenish black. The reaction mixture was then cooled to room temperature, extracted with ethylacetate, dried over Na_2SO_4, and the solvent was evaporated under reduced pressure to obtain a crude mixture of thione intermediate. This mixture was partially purified using silica gel flash column chromatography (*n*-pentane/ethylacetate). (*Note: During purification, a mixture of unidentifiable products and the desired thione intermediate was often encountered. Therefore, the percentage of purity of the thione was determined by ^{1}H NMR before proceeding to the next step*).

Step 2 **:** Thione (1 eq.) was dissolved in acetic acid (4.5 mL/mmol), and 30% H_2O_2 (3.3 eq.) was added while cooling the reaction mixture with a water bath. The mixture was then stirred at ambient temperature (25 °C) for 1 hour. Afterward, the solvent was removed under reduced pressure, and the residue was dissolved in methanol (0.5 mL/mmol). The resulting mixture was cooled to 0 °C, and a solution of $NaClO_4 \cdot H_2O$ (4.12 eq.) in a methanol/water mixture (2:1, total volume 3.5 mL/mmol) was added slowly. After stirring for 10 minutes at 0 °C, the reaction mixture was stirred for an additional 20 minutes at ambient temperature. The reaction mixture was then diluted and extracted with DCM. The organic fractions were dried over Na_2SO_4 and evaporated under reduced pressure. The crude residue was purified by silica gel column chromatography using a DCM/MeOH mixture as the eluent, yielding pure chiral thiazolium salts.

Physical data of chiral thiazolium salts

3-(2,6-Bis((*R*)-1-phenylethyl)phenyl)-5,6,7,8-tetrahydro-4*H*-cyclohepta[*d*]thiazol-3-ium perchlorate (NHC1)

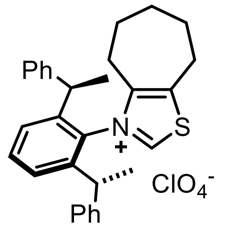

The title compound was synthesized on a 0.5 mmol scale according to the general procedure (**GP 4**) and was obtained as a colorless solid after silica gel column chromatography (DCM: methanol 20:1). Yield: 62% (166.5 mg). $\mathbf{R_f}$ = 0.28 (DCM: methanol 20:1).

MP: 181-182 °C

^{1}**H NMR** (400 MHz, Chloroform-*d*): δ = 8.77 (s, 1H), 7.85 – 7.76 (m, 2H), 7.68 (dd, *J* = 7.4, 1.9 Hz, 1H), 7.24 – 7.19 (m, 3H), 7.17 – 7.11 (m, 3H), 6.75 – 6.70 (m, 2H), 6.66 – 6.61 (m, 2H), 3.89 (q, *J* = 6.9 Hz, 1H), 3.37 (q, *J* = 7.1 Hz, 1H), 3.04 – 2.95 (m, 1H), 2.69 – 2.60 (m, 1H), 1.98 – 1.80 (m, 3H), 1.66 – 1.56 (m, 7H), 1.49 – 1.36 (m, 1H), 1.31 – 1.15 (m, 2H), 1.01 – 0.90 (m, 1H) ppm.

^{13}**C NMR** (101 MHz, Chloroform-*d*): δ = 156.4, 147.7, 144.1, 143.3, 142.1, 140.4, 138.4, 134.9, 131.8, 129.3, 128.6, 127.6, 127.4, 127.2, 126.7, 126.3, 125.8, 40.6, 39.8, 30.4, 27.9, 26.4, 26.2, 24.8, 22.7, 22.0 ppm.

HRMS (ESI/QTOF): *m/z*: $[M]^+$ Calcd for $C_{30}H_{32}NS^+$ 438.2250; Found 438.2257.

IR (ATR): 2931, 2856, 1493, 1450, 1087, 1028, 758, 572 cm^{-1}.

$[\alpha]_D^{20}$ = +90.0 (c = 0.5, $CHCl_3$).

3-(4-Methoxy-2,6-bis((*R*)-1-phenylethyl)phenyl)-5,6,7,8-tetrahydro-4*H*-cyclohepta[*d*]thiazol-3-ium perchlorate (NHC2)

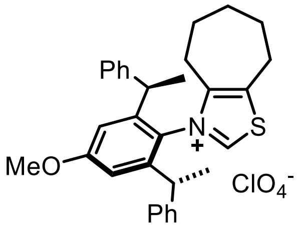

The title compound was synthesized on a 0.5 mmol scale according to the general procedure (**GP 4**) and was obtained as a pale-yellow solid after silica gel column chromatography (DCM: methanol 10:1). Yield: 67% (190 mg). $\mathbf{R_f}$ = 0.26 (DCM: methanol 10:1).

MP: 191-192 °C

^{1}**H NMR** (400 MHz, Chloroform-*d*): δ = 8.71 (s, 1H), 7.24 – 7.19 (m, 4H), 7.16 – 7.11 (m, 4H), 6.76 – 6.71 (m, 2H), 6.67 – 6.63 (m, 2H), 4.02 (s, 3H), 3.86 (q, *J* = 6.8 Hz, 1H), 3.33 (q, *J* = 7.0 Hz, 1H), 3.04 – 2.94 (m, 1H), 2.70 – 2.57 (m, 1H), 2.02 – 1.76 (m, 3H), 1.70 – 1.60 (m, 1H), 1.57 – 1.52 (m, 6H), 1.48 – 1.37 (m, 1H), 1.32 – 1.14 (m, 2H), 1.01 – 0.96 (m, 1H) ppm.

^{13}**C NMR** (101 MHz, Chloroform-*d*): δ = 161.9, 157.2, 148.4, 144.3, 144.1, 143.52, 142.3, 138.5, 129.6, 128.9, 127.94, 127.90, 127.4, 127.0, 126.1, 112.4, 112.3, 55.9, 41.0, 40.2, 30.8, 28.2, 26.8, 26.5, 25.1, 23.0, 22.2 ppm.

HRMS (ESI/QTOF): *m/z*: $[M]^+$ Calcd for $C_{31}H_{34}NOS^+$ 468.2356; Found 468.2359.

IR (ATR): 2962, 1598, 1508, 1091, 622 cm^{-1}.

$[\alpha]_D^{20}$ = +37.0 (c = 1.0, $CHCl_3$).

3-(2,6-Bis((*R*)-1-phenylethyl)-4-(trifluoromethyl)phenyl)-5,6,7,8-tetrahydro-4*H*-cyclohepta[*d*]thiazol-3-ium perchlorate (NHC3)

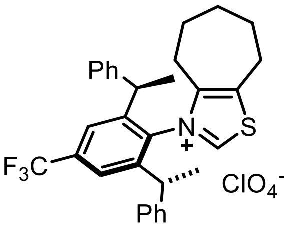

The title compound was synthesized on a 0.5 mmol scale according to the general procedure (**GP 4**) and was obtained as a colorless solid after silica gel column chromatography (DCM: methanol 20:1). Yield: 49% (148 mg). $\mathbf{R_f}$ = 0.32 (DCM: methanol 20:1).

MP: 184-185 °C

^{1}H NMR (400 MHz, Chloroform-*d*): δ = 8.89 (s, 1H), 8.03 (s, 1H), 7.91 (s, 1H), 7.25 – 7.22 (m, 3H), 7.18 – 7.12 (m, 3H), 6.70 (dd, *J* = 7.4, 2.1 Hz, 2H), 6.61 (dd, *J* = 6.6, 3.0 Hz, 2H), 4.02 (q, *J* = 6.9 Hz, 1H), 3.42 (q, *J* = 7.0 Hz, 1H), 3.06 – 2.96 (m, 1H), 2.72 – 2.58 (m, 1H), 2.05 – 1.82 (m, 3H), 1.65 – 1.59 (m, 7H), 1.49 – 1.38 (m, 1H), 1.32 – 1.24 (m, 2H), 0.99 – 0.85 (m, 1H) ppm.

^{13}C NMR (101 MHz, Chloroform-*d*): δ = 155.9, 146.4, 143.2, 142.4, 141.5, 141.2, 138.0, 136.7, 133.0 (q, *J* = 32.7 Hz), 128.7, 127.9, 127.1, 126.3, 126.2, 124.8, 123.6 (q, *J* = 3.8 Hz), 122.26 (q, *J* = 273.6 Hz), 122.4 (q, *J* = 3.7 Hz), 39.9, 39.1, 29.5, 27.0, 25.5, 25.4, 24.0, 21.7, 21.1 ppm.

^{19}F NMR (376 MHz, Chloroform-*d*): δ = -63.26 ppm.

HRMS (ESI/QTOF): *m/z*: $[M]^+$ Calcd for $C_{31}H_{31}F_3NS^+$ 506.2124; Found 506.2131.

IR (ATR): 2925, 1669, 1170, 1093, 623 cm^{-1}.

$[\alpha]_D^{20}$ = +32.0 (c = 1.0, $CHCl_3$).

3-(2,6-Bis((*R*)-1-phenylethyl)phenyl)-4,5,6,7,8,9-hexahydrocycloocta[*d*]thiazol-3-ium perchlorate (NHC4)

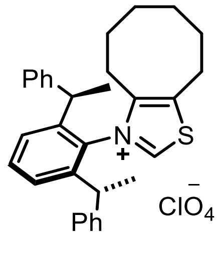

The title compound was synthesized on a 0.5 mmol scale according to the general procedure (**GP 4**) and was obtained as a colorless solid after silica gel column chromatography (DCM: methanol 20:1). Yield: 39% (107.5 mg). $\mathbf{R_f}$ = 0.28 (DCM: methanol 20:1).

MP: 176-177 °C

^{1}H NMR (400 MHz, Chloroform-*d*): δ = 8.73 (s, 1H), 7.87 – 7.78 (m, 2H), 7.71 (dd, *J* = 7.4, 1.8 Hz, 1H), 7.26 – 7.22 (m, 3H), 7.16 – 7.11 (m, 3H), 6.82 – 6.76 (m, 2H), 6.65 – 6.59 (m, 2H), 3.73 (q, *J* = 6.9 Hz, 1H), 3.43 (q, *J* = 7.1 Hz, 1H), 3.14 – 3.01 (m, 1H), 2.80 – 2.67 (m, 1H), 1.98 – 1.86 (m, 1H),

1.82 – 1.66 (m, 2H), 1.60 – 1.58 (m, 3H), 1.57 – 1.54 (m, 3H), 1.50 – 1.38 (m, 5H), 1.36 – 1.30 (m, 2H) ppm.

^{13}C NMR (101 MHz, $CDCl_3$): δ = 157.8, 145.8, 143.6, 143.4, 142.3, 140.5, 137.5, 134.8, 132.0, 129.6, 128.6, 128.0, 127.8, 127.4, 126.9, 126.5, 126.3, 41.0, 40.1, 29.3, 26.3, 25.8, 25.5, 24.5, 24.2, 23.2, 22.2 ppm.

HRMS (ESI/QTOF): *m/z*: $[M]^+$ Calcd for $C_{31}H_{34}NS^+$ 452.2406; Found 452.2422.

IR (ATR): 2930, 1450, 1089, 759, 702, 666 cm^{-1}.

$[\alpha]_D^{20}$ = +33.0 (c = 1.0, $CHCl_3$).

3-(2,6-Bis((*R*)-1-phenylethyl)-4-(trifluoromethyl)phenyl)-4,5,6,7,8,9,10,11,12,13-decahydrocyclododeca[*d*]thiazol-3-ium perchlorate (NHC5)

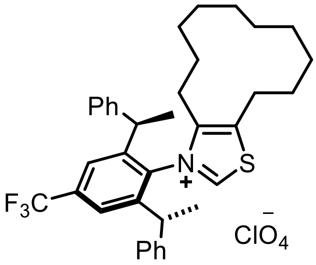

The title compound was synthesized on a 0.5 mmol scale according to the general procedure (**GP 4/GP 5**) and was obtained as a colorless foam after silica gel column chromatography (DCM: methanol 40:1). Yield: 19% (64 mg) by **GP A**; Yield: 37% (124 mg) by **GP B**. **R_f** = 0.32 (DCM: methanol 40:1).

^{1}H NMR (400 MHz, Chloroform-*d*): δ = 8.85 (s, 1H), 8.05 (s, 1H), 7.92 (s, 1H), 7.30 – 7.26 (m, 3H), 7.20 – 7.14 (m, 3H), 6.83 – 6.78 (m, 2H), 6.60 – 6.56 (m, 2H), 3.82 (q, *J* = 6.9 Hz, 1H), 3.45 (q, *J* = 7.0 Hz, 1H), 2.87 – 2.67 (m, 2H), 2.04 – 1.89 (m, 1H), 1.81 – 1.66 (m, 2H), 1.626 – 1.60 (m, 6H), 1.52 – 1.28 (m, 11H), 1.20 – 1.01 (m, 4H) ppm.

^{13}C NMR (101 MHz, Chloroform-*d*): δ = 159.3, 144.5, 144.1, 142.4, 142.3, 142.0, 141.0, 137.3, 134.0 (q, *J* = 32.9 Hz), 129.7, 128.8, 128.2, 127.2, 127.1, 126.0, 124.8 (q, *J* = 3.4 Hz), 123.14 (q, *J* = 3.6 Hz), 123.10 (d, *J* = 273.5 Hz), 41.3, 40.0, 29.3, 25.59, 25.55, 25.3, 25.1, 24.2, 23.8, 23.1, 23.1, 22.2, 21.8, 21.7 ppm.

^{19}F NMR (376 MHz, Chloroform-*d*): δ = -63.21 ppm.

HRMS (ESI/QTOF): *m/z*: $[M]^+$ Calcd for $C_{36}H_{41}F_3NS^+$ 576.2906; Found 576.2921.

IR (ATR): 2929, 2856, 1495, 1451, 1094, 763, 702 cm^{-1}.

$[\alpha]_D^{20}$ = +27.0 (c = 1.0, $CHCl_3$).

3-(2,6-Bis((*R*)-1-(3,5-dimethylphenyl)ethyl)-4-(trifluoromethyl)phenyl)-4,5,6,7,8,9,10,11,12,13-decahydrocyclododeca[*d*]thiazol-3-ium perchlorate (NHC6)

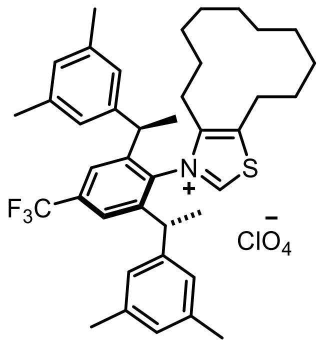

The title compound was synthesized on a 0.5 mmol scale according to the general procedure (**GP 5**) and was obtained as a colorless foam after silica gel column chromatography (DCM: methanol 50:1). Yield: 35% (128 mg). $\mathbf{R_f}$ = 0.30 (DCM: methanol 50:1).

^{1}**H NMR** (400 MHz, Chloroform-*d*): δ = 8.83 (s, 1H), 8.03 (d, *J* = 1.9 Hz, 1H), 7.90 (d, *J* = 1.9 Hz, 1H), 6.92 (s, 1H), 6.80 (s, 1H), 6.35 (s, 1H), 6.10 (s, 2H), 3.75 (q, *J* = 6.8 Hz, 1H), 3.41 (q, *J* = 7.0 Hz, 1H), 2.86 – 2.65 (m, 2H), 2.30 – 2.24 (m, 1H), 2.22 (s, 6H), 2.17 (s, 6H), 1.99 – 1.87 (m, 1H), 1.84 – 1.73 (m, 1H), 1.71 – 1.63 (m, 2H), 1.60 (d, *J* = 6.9 Hz, 3H), 1.54 – 1.58 (m, 3H), 1.48 – 1.34 (m, 10H), 1.21 – 1.02 (m, 4H) ppm.

^{13}**C NMR** (101 MHz, Chloroform-*d*): δ = 160.3, 144.9, 144.5, 142.9, 142.6, 142.4, 140.5, 139.7, 138.6, 137.7 (q, *J* = 1.3 Hz) 134.1 (q, *J* = 32.8 Hz), 130.2, 128.8, 125.3, 124.9 (q, *J* = 3.4 Hz), 124.0, 123.5 (q, *J* = 273.5 Hz), 123.2 (q, *J* = 3.8 Hz), 41.6, 40.2, 29.9, 29.8, 26.0, 25.7, 24.6, 24.5, 23.4, 23.2, 22.8, 22.2, 22.0, 21.4, 21.3 ppm.

^{19}**F NMR** (376 MHz, Chloroform-*d*): δ = -63.15 ppm.

HRMS (ESI/QTOF): *m/z*: $[M]^+$ Calcd for $C_{40}H_{49}F_3NS^+$ 632.3532; Found 632.3547.

IR (ATR): 2922, 2853, 1466, 1451, 1090, 754, 622 cm^{-1}.

$[\boldsymbol{\alpha}]_{\boldsymbol{D}}^{\mathbf{20}}$ = +41.0 (c = 1.0, $CHCl_3$).

3-(2,6-Bis((*R*)-1-(3,5-di-*tert*-butylphenyl)ethyl)-4-(trifluoromethyl)phenyl)-4,5,6,7,8,9,10,11,12,13-decahydrocyclododeca[*d*]thiazol-3-ium perchlorate (NHC7)

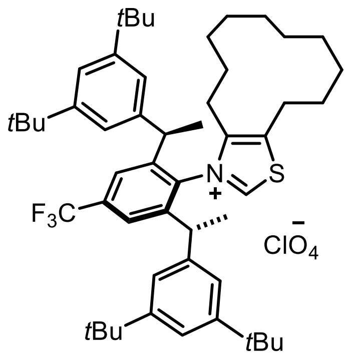

The title compound was synthesized on a 1.0 mmol scale according to the general procedure (**GP 5**) and was obtained as a colorless foam after silica gel column chromatography (DCM: methanol 80:1). Yield: 29% (260 mg). $\mathbf{R_f}$ = 0.30 (DCM: methanol 80:1).

^{1}**H NMR** (400 MHz, Chloroform-*d*): δ = 8.56 (s, 1H), 8.07 (s, 1H), 7.95 (s, 1H), 7.34 (t, *J* = 1.8 Hz, 1H), 7.22 (t, *J* = 1.7 Hz, 1H), 6.76 (d, *J* = 1.8 Hz, 2H), 6.39 (d, *J* = 1.7 Hz, 2H), 3.77 (q, *J* = 7.2 Hz,

1H), 3.36 (q, J = 7.2 Hz, 1H), 2.89 – 2.74 (m, 2H), 2.03 – 1.70 (m, 2H), 1.63 – 1.58 (m, 6H), 1.50 – 1.44 (m, 4H), 1.40 – 1.36 (m, 4H), 1.27 – 1.21 (m, 42H), 1.16 – 1.01 (m, 2H) ppm.

^{13}C NMR (101 MHz, Chloroform-*d*): δ = 160.1, 144.7, 144.3, 142.6, 142.4, 142.2, 140.3, 139.5, 138.4, 137.5, 133.9 (q, J = 33.0 Hz), 130.0, 128.6, 125.1, 124.7 (q, J = 3.6 Hz), 123.8, 123.3 (q, J = 273.7 Hz), 123.0 (q, J = 3.2 Hz), 41.4, 40.0, 36.3, 29.7, 29.6, 25.8, 25.5, 24.4, 24.3, 23.2, 23.0, 22.6, 22.0, 21.8, 21.2, 21.1 ppm.

^{19}F NMR (376 MHz, Chloroform-*d*): δ = -63.25 ppm.

HRMS (ESI/QTOF): *m/z*: $[M]^+$ Calcd for $C_{52}H_{73}F_3NS^+$ 800.5410; Found 800.5415.

IR (ATR): 2926, 2854, 1452, 1349, 1133, 622 cm^{-1}.

$[\alpha]_D^{20}$ = +46.0 (c = 1.0, $CHCl_3$).

3-(4-Methyl-2,6-bis((*R*)-1-phenylethyl)phenyl)-5,6,7,8-tetrahydro-4*H*-cyclohepta[*d*]thiazol-3-ium perchlorate (NHC10)

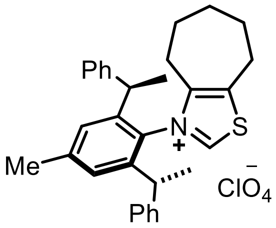

The title compound was synthesized on a 0.5 mmol scale according to the general procedure (**GP 4**) and was obtained as a colorless solid after silica gel column chromatography (DCM: methanol 20:1). Yield: 64% (177 mg). **R_f** = 0.30 (DCM: methanol 20:1).

MP: 186-187 °C

^{1}H NMR (400 MHz, Chloroform-*d*): δ = 8.73 (s, 1H), 7.54 (s, 1H), 7.43 (s, 1H), 7.24 – 7.18 (m, 3H), 7.16 – 7.11 (m, 3H), 6.75 – 6.69 (m, 2H), 6.67 – 6.61 (m, 2H), 3.84 (q, J = 6.9 Hz, 1H), 3.33 (q, J = 7.1 Hz, 1H), 3.04 – 2.94 (m, 1H), 2.67 – 2.57 (m, 4H), 1.99 – 1.89 (m, 2H), 1.87 – 1.79 (m, 1H), 1.65 – 1.58 (m, 1H), 1.57 – 1.52 (m, 6H), 1.47 – 1.36 (m, 1H), 1.27 – 1.17 (m, 2H), 1.0 – 0.9 (m, 1H) ppm.

^{13}C NMR (101 MHz, Chloroform-*d*): δ = 156.8, 148.1, 144.5, 143.7, 142.5, 142.0, 140.3, 138.6, 132.7, 129.5, 128.9, 128.2, 127.8, 127.5, 127.2, 127.0, 126.1, 40.8, 40.0, 30.8, 28.2, 26.7, 26.5, 25.1, 23.0, 22.3, 22.2 ppm.

HRMS (ESI/QTOF): *m/z*: $[M]^+$ Calcd for $C_{31}H_{34}NS^+$ 452.2406; Found 452.2419.

IR (ATR): 2931, 1450, 1088, 912, 763, 729 cm^{-1}.

$[\alpha]_D^{20}$ = +36.0 (c = 1.0, $CHCl_3$).

3-(2,6-Bis((*R*)-1-(3,5-dimethylphenyl)ethyl)phenyl)-5,6,7,8-tetrahydro-4*H*-cyclohepta[*d*]thiazol-3-ium perchlorate (NHC11)

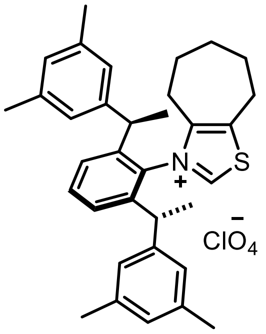

The title compound was synthesized on a 0.5 mmol scale according to the general procedure (**GP 4**) and was obtained as a colorless solid after silica gel column chromatography (DCM: methanol 20:1). Yield: 60% (178 mg). $\mathbf{R_f}$ = 0.32 (DCM: methanol 20:1).

MP: 178-179 °C

^{1}H NMR (400 MHz, Chloroform-*d*): δ = 8.75 (s, 1H), 7.83 – 7.74 (m, 2H), 7.68 – 7.63 (m, 1H), 6.86 (s, 1H), 6.77 (s, 1H), 6.31 (s, 2H), 6.18 (s, 2H), 3.79 (q, *J* = 6.8 Hz, 1H), 3.29 (q, *J* = 7.0 Hz, 1H), 3.02 – 2.93 (m, 1H), 2.70 – 2.60 (m, 1H), 2.24 – 2.12 (m, 13H), 2.03 – 1.92 (m, 2H), 1.89 – 1.80 (m, 1H), 1.69 – 1.61(m, 1H), 1.56 – 1.52 (m, 6H), 1.51 – 1.41 (m, 1H), 1.24 – 1.17 (m, 1H), 1.15 – 1.05 (m, 1H) ppm.

^{13}C NMR (101 MHz, $CDCl_3$): δ = 157.5, 147.9, 144.4, 143.6, 142.6, 140.8, 139.3, 138.4, 138.3, 135.1, 132.0, 129.6, 128.5, 127.7, 126.3, 125.2, 123.8, 40.9, 40.1, 31.0, 29.8, 28.1, 26.9, 26.7, 25.1, 23.0, 22.3, 21.4, 21.3 ppm.

HRMS (ESI/QTOF): *m/z*: $[M]^+$ Calcd for $C_{34}H_{40}NS^+$ 494.2876; Found 494.2892.

IR (ATR): 2924, 1953, 1558, 1265, 1092, 737 cm^{-1}.

$[\boldsymbol{\alpha}]_D^{20}$ = +57.4 (c = 1.0, $CHCl_3$).

3-(4-Methyl-2,6-bis((*R*)-1-phenylethyl)phenyl)-4,5,6,7,8,9,10,11,12,13-decahydrocyclododeca[*d*]thiazol-3-ium perchlorate (NHC12)

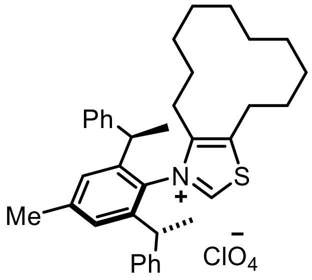

The title compound was synthesized on a 0.5 mmol scale according to the general procedure (**GP 5**) and was obtained as a colorless solid after silica gel column chromatography (DCM: methanol 40:1). Yield: 39% (121 mg). $\mathbf{R_f}$ = 0.30 (DCM: methanol 40:1).

^{1}H NMR (400 MHz, Chloroform-*d*): δ = 8.67 (s, 1H), 7.55 (s, 1H), 7.46 (s, 1H), 7.26 – 7.23 (m, 3H), 7.18 – 7.13 (m, 3H), 6.86 – 6.83 (m, 2H), 6.66 – 6.59 (m, 2H), 3.63 (q, *J* = 6.9 Hz, 1H), 3.34 (q, *J* = 7.0 Hz, 1H), 2.84 – 2.67 (m, 2H), 2.63 (s, 3H), 1.99 – 1.88 (m, 1H), 1.81 – 1.70 (m, 2H), 1.56 – 1.53 (m, 6H), 1.51 – 1.40 (m, 6H), 1.33 – 1.26 (m, 4H), 1.19 – 1.01 (m, 5H) ppm.

^{13}**C NMR** (101 MHz, Chloroform-*d*): δ = 159.4, 145.3, 143.8, 143.7, 142.6, 142.2, 140.8, 140.2, 132.4, 129.7, 128.8, 128.6, 128.1, 127.6, 127.2, 127.0, 126.5, 41.3, 40.0, 29.6, 25.9, 25.7, 25.5, 25.4, 24.6, 24.1, 23.7, 23.4, 22.5, 22.3, 22.2, 22.0 ppm.

HRMS (ESI/QTOF): *m/z*: $[M]^+$ Calcd for $C_{36}H_{44}NS^+$ 522.3189; Found 522.3200.

IR (ATR): 2929, 2857, 1493, 1450, 1093, 762, 730, 702 cm^{-1}.

$[\alpha]_D^{20}$ = +31.6 (c = 1.0, $CHCl_3$).

Starting material synthesis

Isopropyl 2-methyl-2-(4-(4-vinylbenzoyl)phenoxy)propanoate (5a)

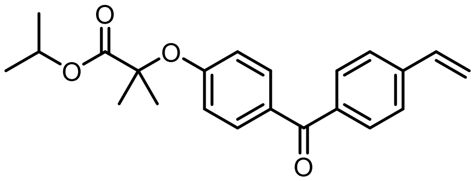

Synthesized according to the literature procedure.[6]

Fenofibrate (1 eq.), potassium vinyltrifluoroborate (1.75 eq.), palladium(II) chloride (5 mol%), RuPhos (10 mol%) and cesium carbonate (3 eq.) were added to a solution of in THF /water (7/1, 2.5 mL/mmol). The reaction mixture was degassed by nitrogen sparging for 5 min, and then stirred at 85 °C in oil bath for 48 h under nitrogen. More water was added, and the mixture was extracted three times with Et_2O. The combined organic fractions were washed once with water, once with brine and dried over anhydrous $MgSO_4$, concentrated in vacuum, and the crude product was purified by flash column chromatography using pentane/EtOAc (15:1 to 9:1) to afford the product **5a** as colorless solid (91% yield).

^{1}**H NMR** (400 MHz, Chloroform-*d*): δ = 7.74 (t, *J* = 8.7 Hz, 4H), 7.49 (d, *J* = 8.3 Hz, 2H), 6.89 – 6.84 (m, 2H), 6.78 (dd, *J* = 17.6, 10.9 Hz, 1H), 5.88 (d, *J* = 17.6 Hz, 1H), 5.39 (d, *J* = 10.9 Hz, 1H), 5.09 (hept, *J* = 6.3 Hz, 1H), 1.66 (s, 6H), 1.20 (d, *J* = 6.3 Hz, 6H) ppm.

Physical data is in accordance with the literature.[6]

(8*R*,9*S*,13*S*,14*S*)-13-methyl-3-vinyl-6,7,8,9,11,12,13,14,15,16-decahydro-17*H*-cyclopenta[*a*]phenanthren-17-one (5b)

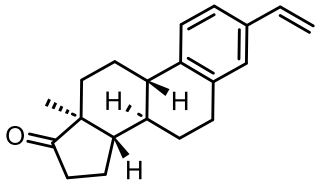

Synthesized according to the literature procedure.[7]

Step 1: An oven-dried Schlenk flask was charged under nitrogen atmosphere with (+)-Estrone (1.0 eq.), triethylamine (2.0 eq.), and dry DCM (2.5 mL/mmol). The mixture was cooled to 0 °C, and triflic anhydride (1.1 eq.) was added dropwise via syringe. The reaction was stirred at 0 °C for 2 hours, after which it was poured into a saturated aqueous solution of $NaHCO_3$. The resulting biphasic mixture was extracted with DCM. The combined organic layers were dried over $MgSO_4$, and the solvent was removed under reduced pressure. The crude product was purified by column chromatography (*n*-pentane : EtOAc, 4:1) to yield 3-(trifluoromethanesulfonyl)estrone (61%) as a colorless solid.

Step 2: The Schlenk flask was then charged with 3-(trifluoromethanesulfonyl)estrone (1.0 eq.), SPhos (0.1 eq.), K_3PO_4 (3.0 eq.), 4,4,5,5-tetramethyl-2-vinyl-1,3,2-dioxaborolane (2.0 eq.), 1,4-dioxane (7.5 ml), and water (1.0 mL/mmol). The reaction vessel was sealed with a septum, evacuated, and refilled with nitrogen (repeated 3 times). $Pd(OAc)_2$ (5 mol%) was added under nitrogen flow, and the reaction mixture was heated to 80 °C and stirred for 22 hours. After cooling to room temperature, the reaction mixture was diluted with EtOAc and filtered through a plug of silica. The organic phase was washed with brine dried over $MgSO_4$, and the solvent was removed under reduced pressure. The residue was purified by column chromatography (*n*-pentane : ethylacetate, 15:1) to yield the product **5b** (86% yield) as a colorless solid.

^{1}H NMR (400 MHz, Chloroform-*d*): δ = 7.27 – 7.17 (m, 2H), 7.14 – 7.11 (m, 1H), 6.65 (dd, *J* = 17.6, 10.9 Hz, 1H), 5.69 (d, *J* = 17.6 Hz, 1H), 5.18 (d, *J* = 10.9 Hz, 1H), 2.90 (dd, *J* = 9.0, 4.3 Hz, 1H), 2.54 – 2.37 (m, 2H), 2.33 – 2.22 (m, 1H), 2.19 – 1.92 (m, 4H), 1.67 – 1.36 (m, 7H), 0.89 (s, 3H) ppm.

Physical data is in accordance with the literature.[7]

(*R*)-2,8-dimethyl-2-((4*R*,8*R*)-4,8,12-trimethyltridecyl)-6-vinylchromane (5c)

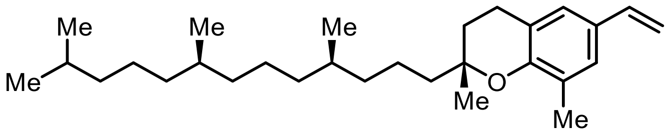

Synthesized according to the literature procedure.[8]

Step 1: To a solution of *δ*-tocopherol (1 eq.) in anhydrous DCM (6.5 mL/mmol), triethylamine (2 eq.) and trifluoromethanesulfonic anhydride (1.2 eq.) were added dropwise at 0 °C. The resulting brown mixture was gradually warmed to room temperature and stirred for 5 hours. The reaction was quenched by adding a saturated aqueous solution of $NaHCO_3$, and the crude product was extracted with DCM. The combined organic layers were dried over $MgSO_4$. After evaporation of the solvent, the concentrate was purified by silica gel column chromatography (*n*-pentane : ethylacetate, 20:1), yielding the desired trifluoromethanesulfonyl-substituted tocopherol derivative as a colorless oil (81% yield).

Step 2: A mixture of trifluoromethanesulfonyl-substituted tocopherol derivative (1 eq.), potassium trifluoroborate (2 eq.), palladium(II) chloride (10 mol%), RuPhos (20 moL%), and cesium carbonate (3 eq.) was placed in a round-bottom flask. The flask was evacuated and filled with nitrogen three times. To the flask, 2.8 mL/mmol of a THF/H_2O (8:1) mixture was added, and the resulting dark brown mixture was stirred at 85 °C for 24 hours. After cooling, additional water was added, and the crude product was extracted with diethyl ether. The combined organic layers were dried over $MgSO_4$. After solvent evaporation, the crude product was purified by silica gel column chromatography(*n*-pentane : ethylacetate, 50:1), yielding the desired vinyl derivative of tocopherol **5c** as a colorless oil (87% yield).

^{1}H NMR (400 MHz, Chloroform-*d*): δ = 7.05 (d, *J* = 2.3 Hz, 1H), 6.95 (d, *J* = 2.2 Hz, 1H), 6.59 (dd, *J* = 17.6, 10.9 Hz, 1H), 5.59 – 5.50 (m, 1H), 5.06 – 5.01 (m, 1H), 2.78 – 2.70 (m, 2H), 2.16 (s, 3H), 1.86 – 1.69 (m, 2H), 1.64 – 0.97 (m, 24H), 0.89 – 0.82 (m, 12H) ppm.

Physical data is in accordance with the literature.[8]

Synthesis of perfluoroalkyl substituted hypervlent iodine reagents (F4 and F5)

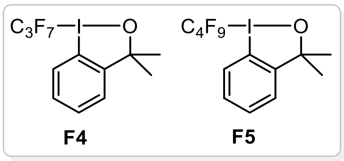

Synthesized according to the literature procedure.[9] Fluoroiodane (1.7 mmol, 1.3 equiv.) was suspended in acetonitrile (5 mL/mmol), and tetrabutylammonium difluorotriphenylsilicate (TBAT) (1 mol%) was introduced at –20 °C. The corresponding (perfluoroalkyl)trimethylsilane (1.0 equiv.) was then added dropwise over a period of 20 minutes. The resulting yellow-brown mixture was stirred for 1 hour at –20 °C, followed by an additional 30 minutes at ambient temperature. Product isolation was achieved either by evaporating the solvent to dryness, followed by purification using flash column chromatography, or by cooling the reaction mixture to –50 °C to promote complete precipitation of the product. The precipitate was then separated by cold filtration, washed with acetonitrile at –50 °C, and dried carefully under vacuum to yield a colorless crystalline.

Compounds **F4** and **F5** have been previously reported in the literature.[9] The spectral data are in accordance with previous literature.[9]

Reaction Optimization for Asymmetric Acyl-Trifluoromethylation of Olefins

Table S1: Catalyst screening

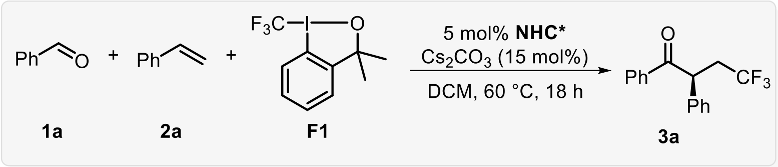

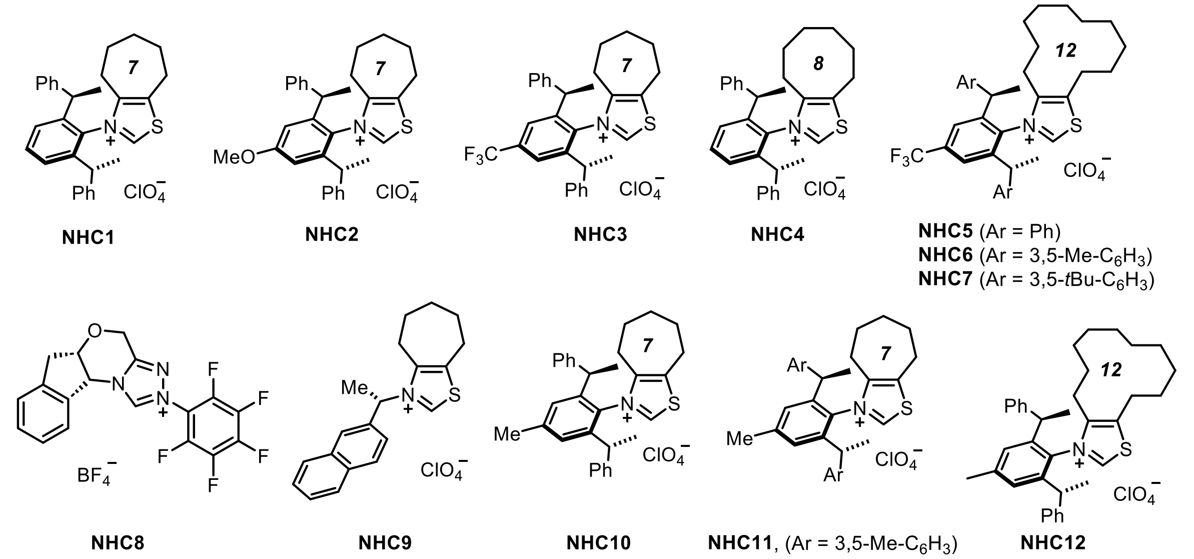

Entry[a]	NHC*	% Yield 3a[b]	er 3a[c]
1	**NHC1**	73 (71)[d]	60:40
2	**NHC2**	74 (73)[d]	60:40
3	**NHC3**	66 (64)[d]	67:33
4	**NHC4**	60	70:30
5	**NHC5**	58	82:18
6	**NHC6**	59	85:15
7	**NHC7**	55 (55)[d]	88:12
8	**NHC8**	58	60:40
9	**NHC9**	39	56:44
10	**NHC10**	71	60:40
11	**NHC11**	68	63:37
12	**NHC12**	64	77:23

[a] Conditions: 0.15 mmol **1a**, 0.1 mmol **2a**, 0.2 mmol **F1**, 5 µmol **NHC***, 15 µmmol Cs_2CO_3 in 1 mL DCM at 60 °C for 18 h. [b] ^{1}H NMR yield using 1,3,5-trimethoxybenzene as an internal standard. [c] Determined by chiral HPLC. [d] Isolated yield.

Table S2: Solvent screening

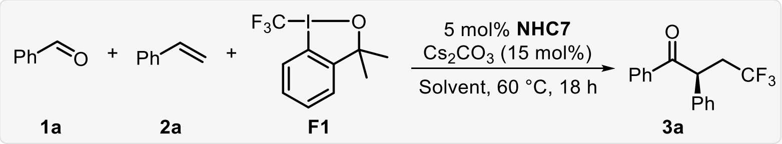

Entry[a]	Solvent	% Yield 3a[b]	er 3a[c]
1	**DCM**	55 (55)[d]	88:12
2	**Toluene**	19	89:11
3	**$PhCF_3$**	13	78:22
4	**THF**	20	85:15
5	**TBME**	61(60)[d]	91:9
6	**CPME**	57	90:10
7	**DIPE**	41	89:11

[a] Conditions: 0.15 mmol **1a**, 0.1 mmol **2a**, 0.2 mmol **F1**, 5 µmol **NHC7**, 15 µmmol Cs_2CO_3 in 1 mL solvent at 60 °C for 18 h. [b] ^1H NMR yield using 1,3,5-trimethoxybenzene as an internal standard. [c] Determined by chiral HPLC. [d] Isolated yield. DCM – Dichloromethane; THF – Tetrahydrofuran; CPME - Cyclopentyl methyl ether; DIPE - Diisopropyl ether; TBME - *tert*-Butyl methyl ether.

Table S3: Base screening

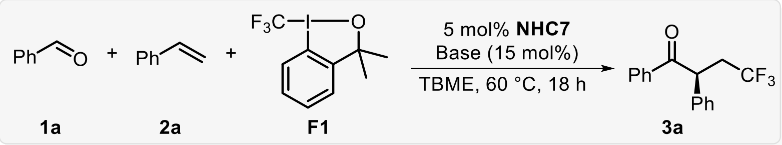

Entry[a]	Base	% Yield 3a[b]	er 3a[c]
1	**Cs_2CO_3**	61 (60)[d]	91:9
2	**K_2CO_3**	57	90:10
3	**Na_2CO_3**	*no reaction*	-
4	**CsOAc**	36	90:10
5	**KOAc**	40	90:10
6	**DBU**	41	86:14
7	**DIPEA**	trace	-
8	**DABCO**	trace	-
9	**Et_3N**	trace	-
10	**Cs_2CO_3 (20 mol%)**[e]	56	91:9
11	**Cs_2CO_3 (10 mol%)**[e]	57	91:9

[a] Conditions: 0.15 mmol **1a**, 0.1 mmol **2a**, 0.2 mmol **F1**, 5 µmol **NHC7**, 15 µmmol base in 1 mL TBME at 60 °C for 18 h. [b] ^1H NMR yield using 1,3,5-trimethoxybenzene as an internal standard. [c] Determined by chiral HPLC. [d] Isolated yield. [e] Different stoichiometries of Cs_2CO_3.

Table S4: Temperature and time screening

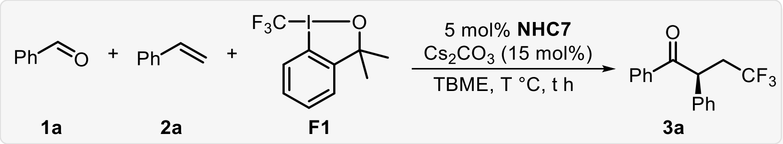

Entry[a]	Temperature (T °C)	Time (h)	% Yield 3a[b]	er 3a[c]
1	**60**	18	61 (60)[d]	91:9
2	**40**	18	39	96:4
3	**70**	18	63	89:11
4	40	**24**	44	96:4
5	40	**36**	55	96:4
6[e]	40	**36**	79 (76)[d]	96:4

[a] Conditions: 0.15 mmol **1a**, 0.1 mmol **2a**, 0.2 mmol **F1**, 5 µmol **NHC7**, 15 µmmol Cs_2CO_3 in 1 mL TBME at T °C for t h. [b] ^{1}H NMR yield using 1,3,5-trimethoxybenzene as an internal standard. [c] Determined by chiral HPLC. [d] Isolated yield. [e] 10 µmol of **NHC7** and 25 µmol of Cs_2CO_3 loadings.

Table S5: Stoichiometry screening

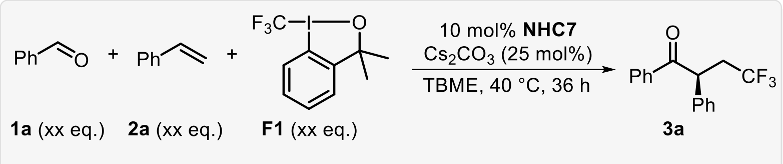

Entry[a]	1a (xx eq.)	2a (xx eq.)	F1 (xx eq.)	% Yield 3a[b]	er 3a[c]
1	**1.5**	**1**	**2**	79 (76)[d]	96:4
2	**1**	**1.5**	**2**	60	95:5
3	**1.5**	**1**	**1**	42	95:5
4	**2**	**1**	**2**	75	96:4
5	**1.5**	**1**	**3**	76.5	96:4

[a] Conditions: xx mmol **1a**, xx mmol **2a**, xx mmol **F1**, 10 µmol **NHC7**, 25 µmmol Cs_2CO_3 in 1 mL TBME at 40 °C for 36 h. [b] ^{1}H NMR yield using 1,3,5-trimethoxybenzene as an internal standard. [c] Determined by chiral HPLC. [d] Isolated yield.

Table S6: Concentration screening

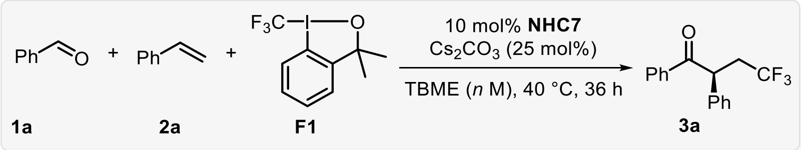

Entry[a]	TBME (mL)	Concentration (*n* M)	% Yield 3a[b]	er 3a[c]
1	**1**	0.1	79 (76)[d]	96:4
2	**0.5**	0.2	76	93:7
3	**2**	0.05	74	96:4

[a] Conditions: 0.15 mmol **1a**, 0.1 mmol **2a**, 0.2 mmol **F1**, 10 µmol **NHC7**, 25 µmmol Cs_2CO_3 in *n* mL TBME at 40 °C for 36 h. [b] ^{1}H NMR yield using 1,3,5-trimethoxybenzene as an internal standard. [c] Determined by chiral HPLC. [d] Isolated yield.

Catalysis procedures

General procedure for the thiazolium carbene-catalyzed three-component acyl-trifluoromethylation reaction (GP 6)

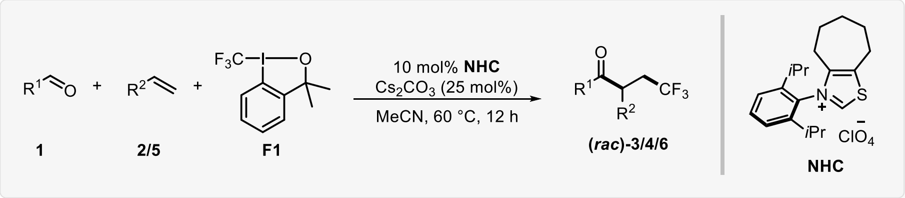

According to the literature procedure.[10]

In a nitrogen-filled glove box, an oven-dried 5 mL reaction tube was charged with the thiazolium salt **NHC** (0.01 mmol, 10 mol%). Dry, degassed MeCN (1 mL) was then added. Subsequently, aldehyde **1** (0.15 mmol, 1.5 eq.), olefin **2** or **5** (0.10 mmol, 1.0 eq.), Togni reagent I **F1** (0.2 mmol, 2 eq.), and Cs_2CO_3 (0.025 mmol, 25 mol%) were added to the reaction tube, which was then sealed with a crimper. The reaction tube was removed from the glove box and stirred at 60 °C for 12 hours. Afterward, the reaction mixture was filtered through a short silica gel pad (2 cm), and the solvent was evaporated under reduced pressure. The crude product mixture was purified by silica gel column chromatography using an *n*-pentane : ethylacetate mixture as the eluent to obtain the racemic mixture of desired product.

General procedure for the chiral thiazolium carbene-catalyzed asymmetric acyl-trifluoromethylation reaction of olefins (GP 7)

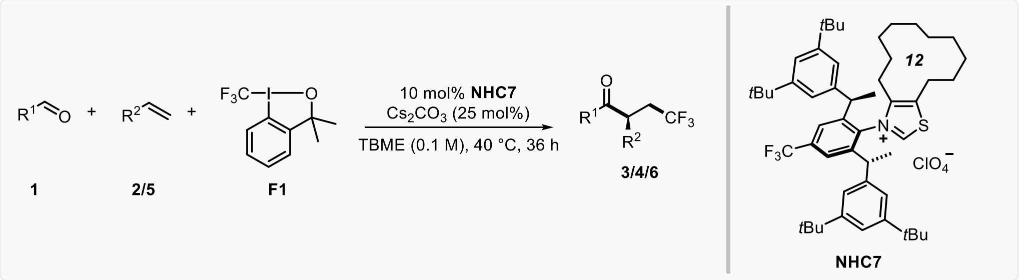

In a nitrogen-filled glove box, an oven-dried 5 mL reaction tube was charged with chiral thiazolium salt **NHC7** (0.01 mmol, 10 mol%). Then, 1 mL of dry and degassed *tert*-butyl methyl ether (TBME) was added. Subsequently, aldehyde **1** (0.15 mmol, 1.5 eq.), olefin **2** or **5** (0.1 mmol, 1.0 eq.), Togni reagent I **F1** (0.2 mmol, 2 eq.), and Cs_2CO_3 (0.025 mmol, 25 mol%) were added to the reaction tube, which was then sealed with a crimper. The reaction tube was removed from the glove box and stirred at 40 °C for 36 hours. The reaction mixture was then filtered through a short pad of silica gel (2 cm), and the solvent was evaporated under reduced pressure. The crude product mixture was purified by silica gel column chromatography using *n*-pentane : ethylacetate mixture as eluent to obtain corresponding enantioenriched β-trifluoromethylated α-chiral ketones (**3**/**4**/**6**).

Physical data

(*S*)-4,4,4-Trifluoro-1,2-diphenylbutan-1-one (3a)

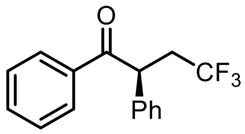

The title compound was synthesized according to the general procedure (**GP 7**) and was obtained after silica gel column chromatography (*n*-pentane : ethylacetate 50:1) as a colorless oil (76 % Yield, 21 mg). $\mathbf{R_f}$ = 0.34 (*n*-pentane : ethylacetate 50:1).

^{1}H NMR (400 MHz, Chloroform-*d*): δ = 8.00 – 7.93 (m, 2H), 7.54 – 7.48 (m, 1H), 7.44 – 7.37 (m, 2H), 7.32 – 7.30 (m, 4H), 7.27 – 7.21 (m, 1H), 4.91 (dd, *J* = 7.7, 5.4 Hz, 1H), 3.44 – 3.19 (m, 1H), 2.69 – 2.38 (m, 1H) ppm.

^{13}C NMR (101 MHz, Chloroform-*d*): δ = 196.8, 137.5, 135.8, 133.5, 129.4, 128.9, 128.8, 128.2, 127.9, 126.5 (q, *J* = 277.2 Hz), 47.3 (q, *J* = 2.6 Hz), 37.5 (q, *J* = 28.2 Hz) ppm.

^{19}F NMR (376 MHz, Chloroform-*d*): δ = -65.13 (t, *J* = 10.6 Hz) ppm.

HRMS (APCI/QTOF) *m/z*: $[M + H]^+$ Calcd for $C_{16}H_{14}F_3O^+$ 279.0991; Found 279.0987.

IR (ATR): 1685, 1259, 1107, 1074, 697 cm^{-1}.

$[\alpha]_D^{20}$ = +49.2 (c = 0.5, $CHCl_3$).

Chiral HPLC: (Chiralpak IA, 0.1 % *i*PrOH/hexane, 1.0 mL/min, 210 nm): tR (major) 5.75 min, tR (minor) 6.84 min, 96:4 *er*.

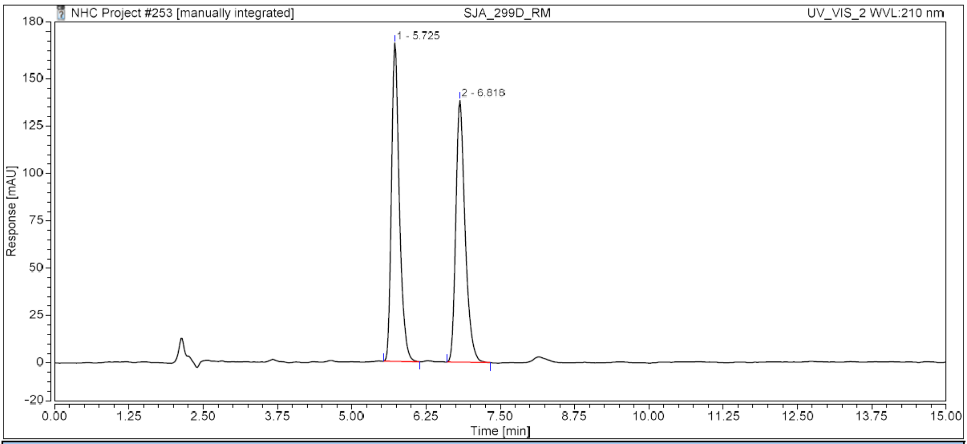

Integration Results

No.	Peak Name	Retention Time min	Area mAU*min	Height mAU	Relative Area %	Relative Height %	Amount n.a.
1		5.725	25.651	168.177	50.38	54.87	n.a.
2		6.818	25.261	138.299	49.62	45.13	n.a.
**Total:**			**50.911**	**306.476**	**100.00**	**100.00**	

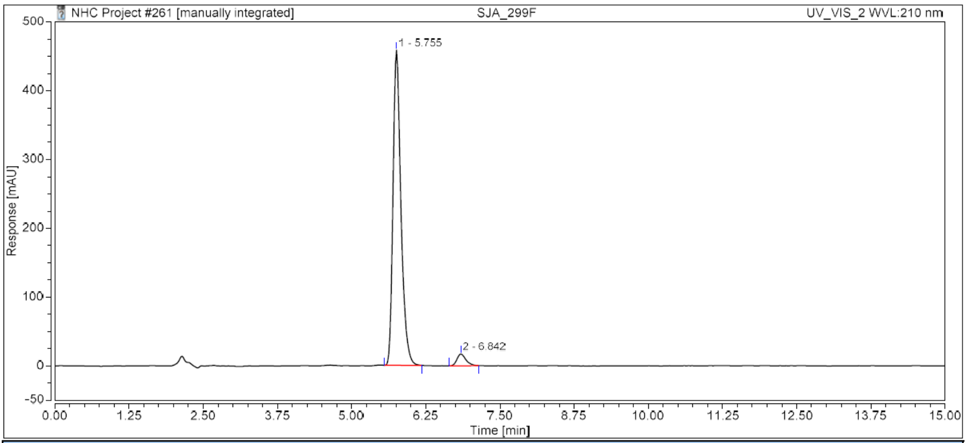

Integration Results							
No.	Peak Name	Retention Time min	Area mAU*min	Height mAU	Relative Area %	Relative Height %	Amount n.a.
1		5.755	70.415	458.550	95.87	96.38	n.a.
2		6.842	3.034	17.237	4.13	3.62	n.a.
Total:			73.449	475.787	100.00	100.00	

(*S*)-4,4,4-Trifluoro-1-(4-methoxyphenyl)-2-phenylbutan-1-one (3b)

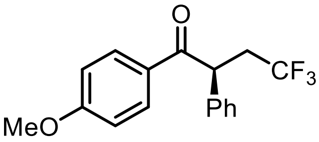

The title compound was synthesized according to the general procedure (**GP 7**) and was obtained after silica gel column chromatography (*n*-pentane : ethylacetate 30:1) as a colorless viscous oil (72 % Yield, 22 mg). **R$_f$** = 0.26 (*n*-pentane : ethylacetate 30:1).

^{1}H NMR (400 MHz, Chloroform-*d*): δ = 7.99 – 7.92 (m, 2H), 7.30 (d, *J* = 4.4 Hz, 4H), 7.26 – 7.20 (m, 1H), 6.90 – 6.85 (m, 2H), 4.85 (dd, *J* = 7.6, 5.5 Hz, 1H), 3.82 (s, 3H), 3.37 – 3.21 (m, 1H), 2.60 – 2.45 (m, 1H) ppm.

^{13}C NMR (101 MHz, Chloroform-*d*): δ = 195.3, 163.8, 138.0, 131.3, 129.4, 128.7, 128.1, 127.8, 126.6 (q, *J* = 277.2 Hz), 114.0, 55.6, 46.9 (q, *J* = 2.4 Hz), 37.5 (q, *J* = 28.1 Hz) ppm.

^{19}F NMR (376 MHz, Chloroform-*d*): δ = -65.10 (t, *J* = 10.8 Hz) ppm.

HRMS (ESI/QTOF) *m/z*: $[M + H]^+$ Calcd for $C_{17}H_{16}F_3O_2^+$ 309.1097; Found 309.1101.

IR (ATR): 1674, 1600, 1258, 1237, 938 cm^{-1}.

$[\alpha]_D^{20}$ = +44.2 (c = 0.5, $CHCl_3$).

Chiral HPLC: (Chiralpak IC, 0.5 % *i*PrOH/hexane, 1.0 mL/min, 254 nm): tR (major) 10.87 min, tR (minor) 12,26 min, 97:3 *er.*

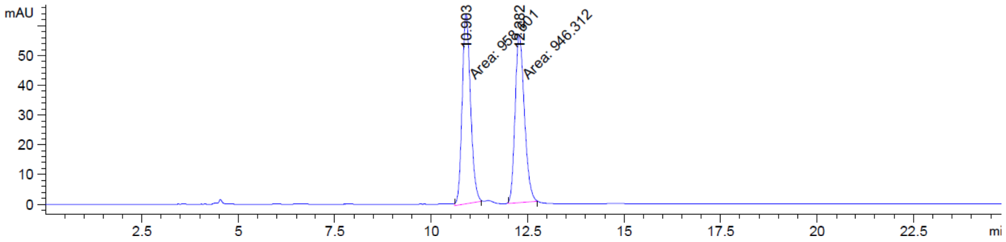

Peak #	RetTime [min]	Type	Width [min]	Area [mAU*s]	Height [mAU]	Area %
1	10.903	MM	0.2509	958.60126	63.67181	50.3226
2	12.282	MM	0.2802	946.31165	56.28990	49.6774

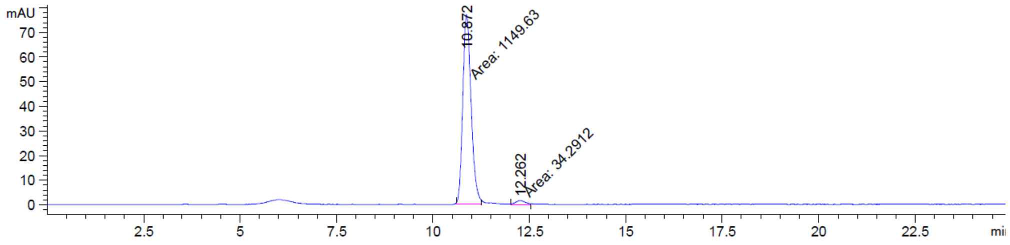

Peak #	RetTime [min]	Type	Width [min]	Area [mAU*s]	Height [mAU]	Area %
1	10.872	MM	0.2493	1149.63025	76.86598	97.1036
2	12.262	MM	0.3175	34.29121	1.80019	2.8964

(*S*)-4,4,4-Trifluoro-1-(4-nitrophenyl)-2-phenylbutan-1-one (3c)

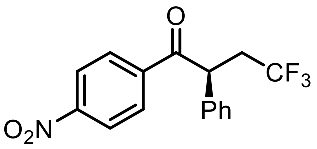

The title compound was synthesized according to the general procedure (**GP 7**) and was obtained after silica gel column chromatography (*n*-pentane : ethylacetate 20:1) as a yellow oil (60 % Yield, 20 mg). $\mathbf{R_f}$ = 0.24 (*n*-pentane : ethylacetate 20:1).

^{1}H NMR (400 MHz, Chloroform-*d*): δ = 8.19 – 8.13 (m, 2H), 8.04 – 7.97 (m, 2H), 7.29 – 7.24 (m, 2H), 7.23 – 7.17 (m, 3H), 4.79 (dd, *J* = 7.8, 5.4 Hz, 1H), 3.25 (dqd, *J* = 15.1, 10.7, 7.7 Hz, 1H), 2.49 (dqd, *J* = 15.0, 10.6, 5.4 Hz, 1H) ppm.

^{13}C NMR (101 MHz, Chloroform-*d*): δ = 195.3, 150.3, 140.2, 136.2, 129.8, 129.7, 128.3, 128.0, 126.1 (q, *J* = 277.2 Hz), 123.8, 48.0 (q, *J* = 2.5 Hz), 37.2 (q, *J* = 28.5 Hz) ppm.

^{19}F NMR (376 MHz, Chloroform-*d*): δ = -65.12 (t, *J* = 10.4 Hz) ppm.

HRMS (ESI/QTOF): m/z: $[M + H_{-1}]^-$ Calcd for $C_{16}H_{11}F_3NO_3^-$ 322.0697; Found 322.0687

IR (ATR): 1711, 1672, 1558, 1379, 1211, 1060, 836 cm^{-1}.

$[\alpha]_D^{20}$ = +54.6 (c = 0.5, $CHCl_3$).

Chiral HPLC: (Chiralpak IB, 1 % *i*PrOH/hexane, 1.0 mL/min, 254 nm): tR (major) 8.01 min, tR (minor) 10.73 min, 93.5:6.5 *er*.

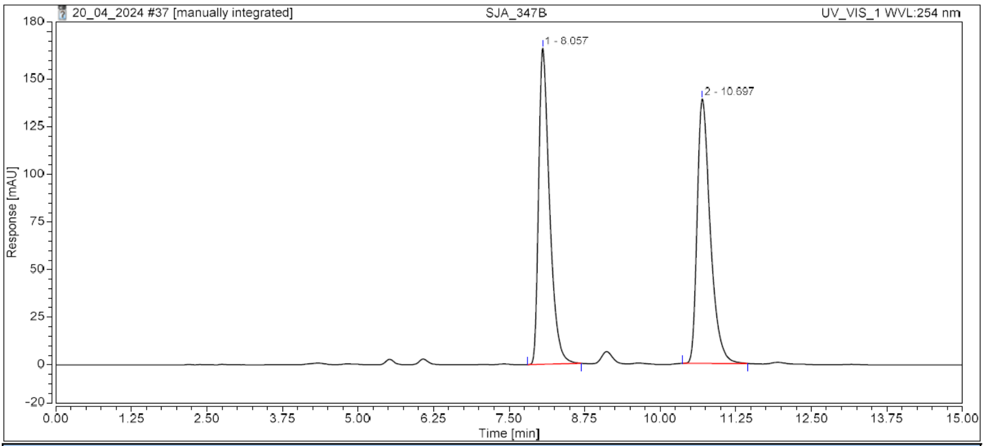

Integration Results							
No.	Peak Name	Retention Time min	Area mAU*min	Height mAU	Relative Area %	Relative Height %	Amount n.a.
1		8.057	34.917	165.957	50.07	54.44	n.a.
2		10.697	34.825	138.874	49.93	45.56	n.a.
**Total:**			**69.742**	**304.831**	**100.00**	**100.00**	

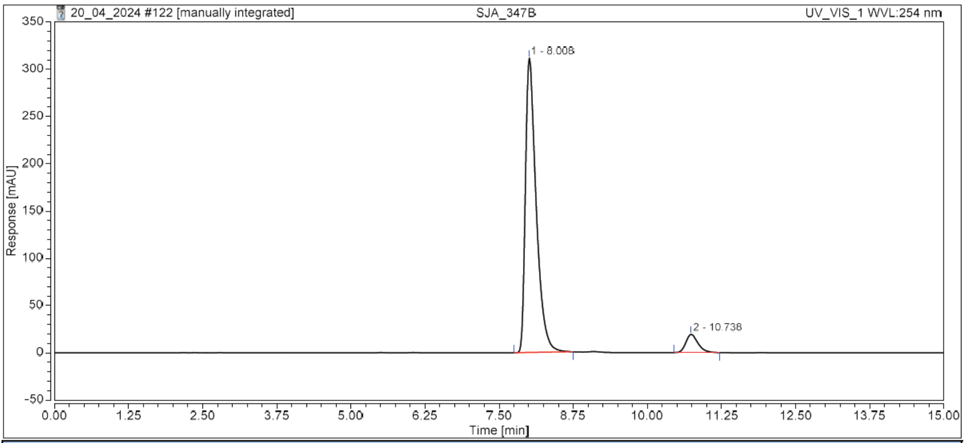

Integration Results							
No.	Peak Name	Retention Time min	Area mAU*min	Height mAU	Relative Area %	Relative Height %	Amount n.a.
1		8.008	65.811	311.434	93.49	94.19	n.a.
2		10.738	4.584	19.224	6.51	5.81	n.a.
**Total:**			**70.395**	**330.658**	**100.00**	**100.00**	

Methyl (*S*)-4-(4,4,4-trifluoro-2-phenylbutanoyl)benzoate (3d)

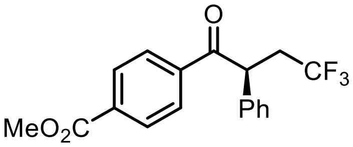

The title compound was synthesized according to the general procedure (**GP 7**) and was obtained after silica gel column chromatography (*n*-pentane : ethylacetate 20:1) as a colorless solid (64 % Yield, 21.5 mg). $\mathbf{R_f}$ = 0.28 (*n*-pentane : ethylacetate 20:1).

^{1}**H NMR** (400 MHz, Chloroform-*d*): δ = 8.02 – 7.97 (m, 2H), 7.96 – 7.91 (m, 2H), 7.29 – 7.16 (m, 5H), 4.84 (dd, *J* = 7.6, 5.5 Hz, 1H), 3.86 (s, 3H), 3.35 – 3.18 (m, 1H), 2.58 – 2.43 (m, 1H) ppm.

^{13}**C NMR** (101 MHz, Chloroform-*d*): δ = 196.4, 166.1, 139.0, 136.9, 134.1, 129.9, 129.6, 128.8, 128.2, 126.4 (q, *J* = 277.2 Hz), 52.6, 47.8 (q, *J* = 2.6 Hz), 37.3 (q, *J* = 28.4 Hz) ppm.

^{19}**F NMR** (376 MHz, Chloroform-*d*): δ = -65.10 (t, *J* = 10.5 Hz) ppm.

HRMS (ESI/QTOF) m/z: $[M + H]^+$ Calcd for $C_{18}H_{16}F_3O_3^+$ 337.1046; Found 337.1041

IR (ATR): 1721, 1380, 1277, 1258, 1140, 1108, 720 cm^{-1}.

$[\alpha]_D^{20}$ = +37.2 (c = 1.0, $CHCl_3$).

Chiral HPLC: (Chiralpak IC, 1 % *i*PrOH/hexane, 1.0 mL/min, 210 nm): tR (major) 7.72 min, tR (minor) 9.17 min, 93:7 *er*.

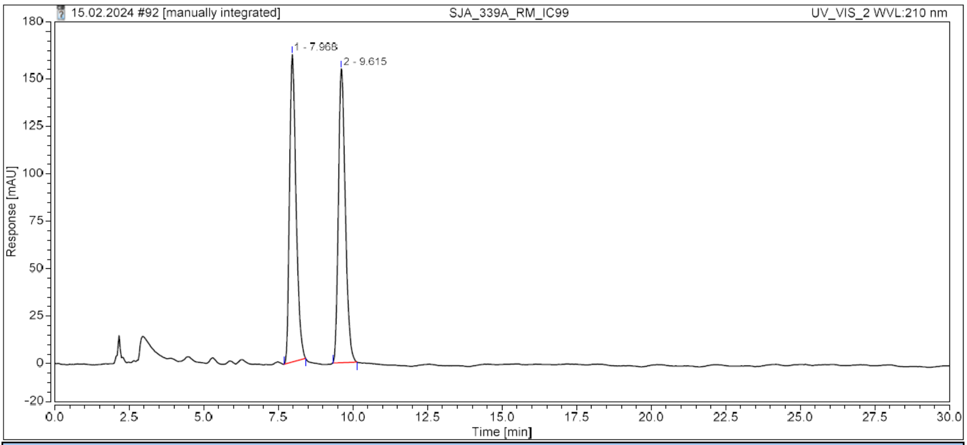

Integration Results

No.	Peak Name	Retention Time min	Area mAU*min	Height mAU	Relative Area %	Relative Height %	Amount n.a.
1		7.968	41.228	162.123	49.74	51.15	n.a.
2		9.615	41.656	154.838	50.26	48.85	n.a.
**Total:**			**82.884**	**316.961**	**100.00**	**100.00**	

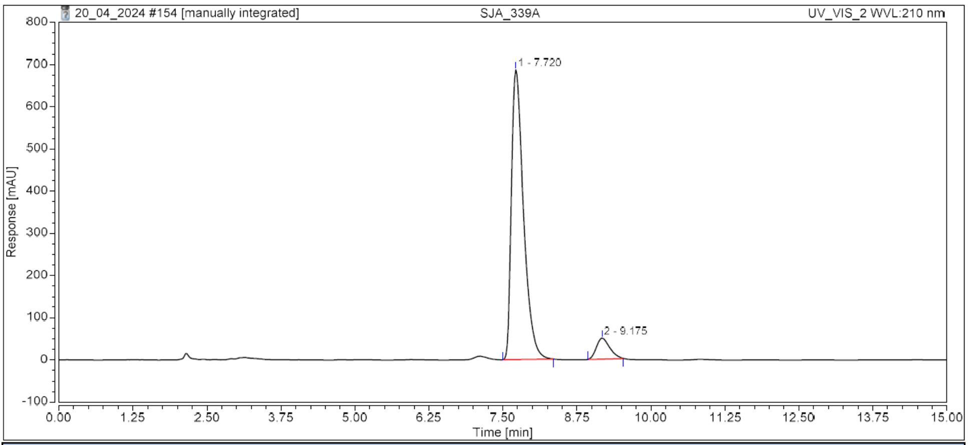

Integration Results

No.	Peak Name	Retention Time min	Area mAU*min	Height mAU	Relative Area %	Relative Height %	Amount n.a.
1		7.720	162.189	687.172	92.63	93.31	n.a.
2		9.175	12.898	49.299	7.37	6.69	n.a.
**Total:**			**175.087**	**736.471**	**100.00**	**100.00**	

(*S*)-4,4,4-Trifluoro-1-(3-methoxyphenyl)-2-phenylbutan-1-one (3e)

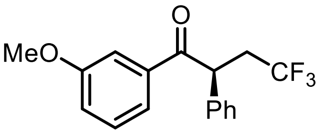

The title compound was synthesized according to the general procedure (**GP 7**) and was obtained after silica gel column chromatography (*n*-pentane : ethylacetate 30:1) as a colorless oil (82 % Yield, 25 mg). $\mathbf{R_f}$ = 0.28 (*n*-pentane : ethylacetate 30:1).

^{1}H NMR (400 MHz, Chloroform-*d*): δ = 7.58 – 7.53 (m, 1H), 7.49 (dd, *J* = 2.7, 1.6 Hz, 1H), 7.35 – 7.29 (m, 5H), 7.27 – 7.22 (m, 1H), 7.06 (ddd, *J* = 8.2, 2.7, 0.9 Hz, 1H), 4.89 (dd, *J* = 7.8, 5.4 Hz, 1H), 3.82 (s, 3H), 3.31 (dqd, *J* = 15.0, 10.8, 7.7 Hz, 1H), 2.55 (dqd, *J* = 15.0, 10.8, 5.4 Hz, 1H) ppm.

^{13}C NMR (101 MHz, Chloroform-*d*): δ = 196.5, 159.8, 137.4, 137.0, 129.6, 129.3, 128.0, 127.8, 126.38 (q, *J* = 277.2 Hz), 121.3, 119.8, 113.2, 55.4, 47.3 (q, *J* = 2.5 Hz), 37.3 (q, *J* = 28.2 Hz) ppm.

^{19}F NMR (376 MHz, Chloroform-*d*): δ = -65.14 (t, *J* = 10.8 Hz) ppm.

HRMS (ESI/QTOF) m/z: $[M + Na]^+$ Calcd for $C_{17}H_{15}F_3NaO_2^+$ 331.0916; Found 331.0905.

IR (ATR): 1685, 1597, 1454, 1263, 1106, 680 cm^{-1}.

$[\alpha]_D^{20}$ = +52.4 (c = 0.5, $CHCl_3$).

Chiral HPLC: (Chiralpak IB, 1 % *i*PrOH/hexane, 1.0 mL/min, 254 nm): tR (major) 3.62 min, tR (minor) 5.15 min, 94.5:5.5 *er*.

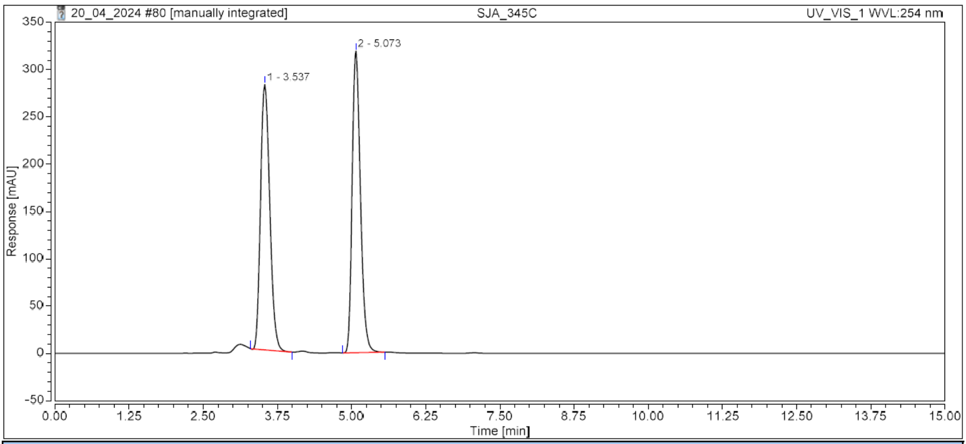

Integration Results							
No.	Peak Name	Retention Time min	Area mAU*min	Height mAU	Relative Area %	Relative Height %	Amount n.a.
1		3.537	50.731	280.545	49.67	46.81	n.a.
2		5.073	51.408	318.834	50.33	53.19	n.a.
**Total:**			**102.139**	**599.380**	**100.00**	**100.00**	

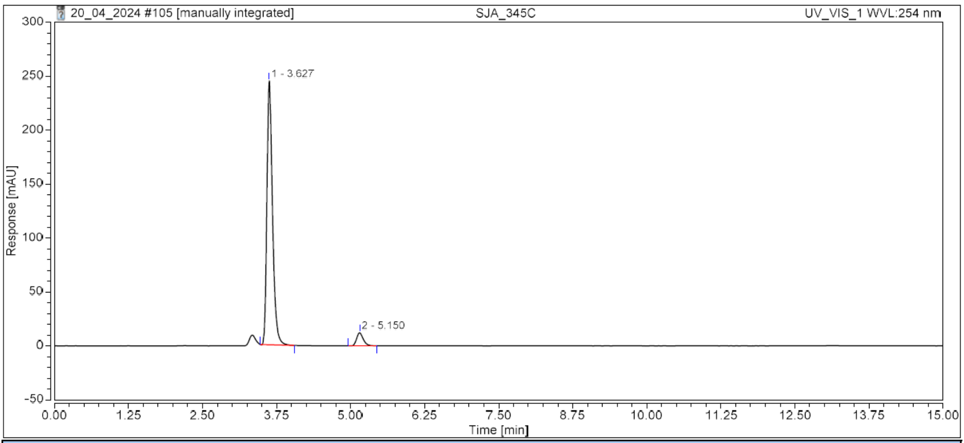

Integration Results							
No.	Peak Name	Retention Time min	Area mAU*min	Height mAU	Relative Area %	Relative Height %	Amount n.a.
1		3.627	26.683	244.567	94.45	95.24	n.a.
2		5.150	1.569	12.214	5.55	4.76	n.a.
**Total:**			**28.252**	**256.781**	**100.00**	**100.00**	

(*S*)-4,4,4-Trifluoro-1-(2-fluorophenyl)-2-phenylbutan-1-one (3f)

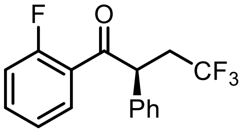

The title compound was synthesized according to the general procedure (**GP 7**) and was obtained after silica gel column chromatography (*n*-pentane : ethylacetate 40:1) as a colorless oil (64 % Yield, 19 mg). **R_f** = 0.3 (*n*-pentane : ethylacetate 40:1).

^{1}H NMR (400 MHz, Chloroform-*d*): δ = 7.76 (td, *J* = 7.6, 1.9 Hz, 1H), 7.49 – 7.42 (m, 1H), 7.32 – 7.19 (m, 5H), 7.19 – 7.12 (m, 1H), 7.09 – 7.02 (m, 1H), 4.89 (dd, *J* = 7.6, 5.6 Hz, 1H), 3.43 – 3.24 (m, 1H), 2.60 – 2.42 (m, 1H) ppm.

^{13}C NMR (101 MHz, Chloroform-*d*): δ : 195.87 (d, *J* = 4.4 Hz), 161.22 (d, *J* = 254.5 Hz), 136.62, 134.94 (d, *J* = 9.2 Hz), 131.47 (d, *J* = 2.6 Hz), 129.21, 128.54, 128.04, 126.49 (d, *J* = 277.2 Hz), 125.01 (d, *J* = 12.3 Hz), 124.69 (d, *J* = 3.4 Hz), 116.84 (d, *J* = 24.0 Hz), 51.42 – 50.79 (m), 37.17 (q, *J* = 28.3 Hz) ppm.

^{19}F NMR (376 MHz, Chloroform-*d*): δ = -65.13 (t, *J* = 10.6 Hz), -109.95 (dt, *J* = 12.3, 6.7 Hz) ppm.

HRMS (ESI/QTOF) m/z: $[M + H]^+$ Calcd for $C_{16}H_{13}F_4O^+$ 297.0897; Found 297.0895.

IR (ATR): 1686, 1609, 1482, 1256, 1137, 700 cm^{-1}.

$[\alpha]_D^{20}$ = +74.6 (c = 0.5, $CHCl_3$).

Chiral HPLC: (Chiralpak IA, 0.1 % *i*PrOH/hexane, 1.0 mL/min, 254 nm): tR (major) 7.00 min, tR (minor) 7.52 min, 95.5:4.5 *er*.

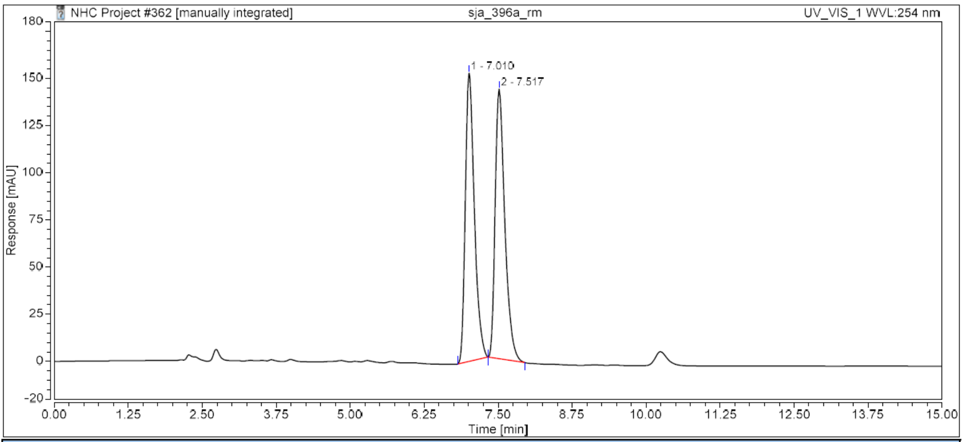

Integration Results

No.	Peak Name	Retention Time min	Area mAU*min	Height mAU	Relative Area %	Relative Height %	Amount n.a.
1		7.010	26.933	152.832	49.93	51.66	n.a.
2		7.517	27.007	143.011	50.07	48.34	n.a.
Total:			53.941	295.843	100.00	100.00	

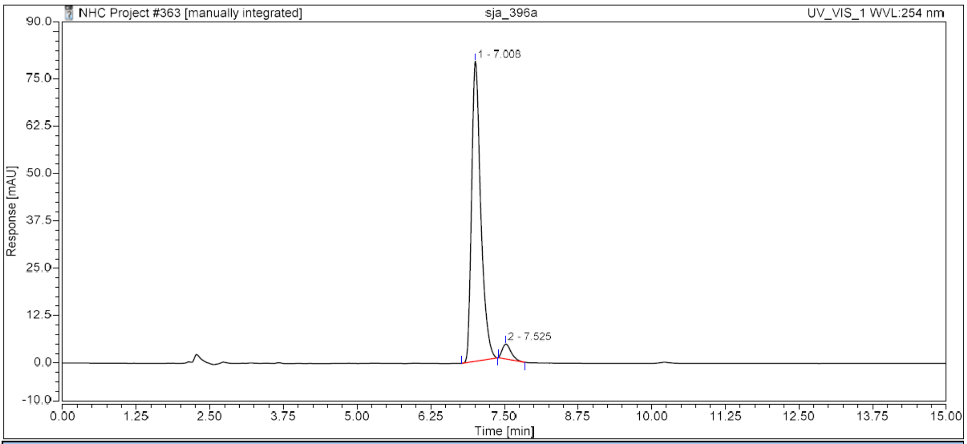

Integration Results

No.	Peak Name	Retention Time min	Area mAU*min	Height mAU	Relative Area %	Relative Height %	Amount n.a.
1		7.008	14.259	79.206	95.48	95.26	n.a.
2		7.525	0.674	3.945	4.52	4.74	n.a.
Total:			14.933	83.151	100.00	100.00	

(*S*)-4,4,4-Trifluoro-1-(2-hydroxyphenyl)-2-phenylbutan-1-one (3g)

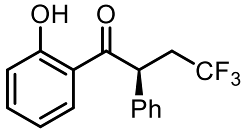

The title compound was synthesized according to the general procedure (**GP 7**) and was obtained after silica gel column chromatography (*n*-pentane : ethylacetate 30:1) as a colorless oil (61 % Yield, 18 mg). **R_f** = 0.26 (*n*-pentane : ethylacetate 30:1).

^{1}H NMR (400 MHz, Chloroform-*d*): δ = 12.11 (s, 1H), 7.81 (dd, *J* = 8.1, 1.6 Hz, 1H), 7.46 – 7.39 (m, 1H), 7.37 – 7.31 (m, 4H), 7.30 – 7.24 (m, 1H), 6.96 (dd, *J* = 8.5, 1.2 Hz, 1H), 6.87 – 6.81 (m, 1H), 4.95 (dd, *J* = 7.9, 5.2 Hz, 1H), 3.38 – 3.22 (m, 1H), 2.63 – 2.48 (m, 1H) ppm.

^{13}C NMR (101 MHz, Chloroform-*d*): δ = 202.4, 163.5, 137.3, 136.9, 130.2, 129.6, 128.2, 127.9, 126.3 (q, *J* = 277.3 Hz), 119.2, 118.9, 118.3, 46.6 (q, *J* = 2.5 Hz), 37.1 (q, *J* = 28.4 Hz) ppm.

^{19}F NMR (376 MHz, Chloroform-*d*): δ = -65.17 (t, *J* = 10.8 Hz) ppm.

HRMS (ESI/QTOF) m/z: $[M + H]^+$ Calcd for $C_{16}H_{14}F_3O_2^+$ 295.0940; Found 295.0942.

IR (ATR): 1638, 1487, 1447, 1267, 1140, 751 cm^{-1}.

$[\alpha]_D^{20}$ = +62.4 (c = 0.5, $CHCl_3$).

Chiral HPLC: (Chiralpak IB, 1 % *i*PrOH/hexane, 1.0 mL/min, 254 nm): tR (major) 7.42 min, tR (minor) 8.74 min, 94:6 *er*.

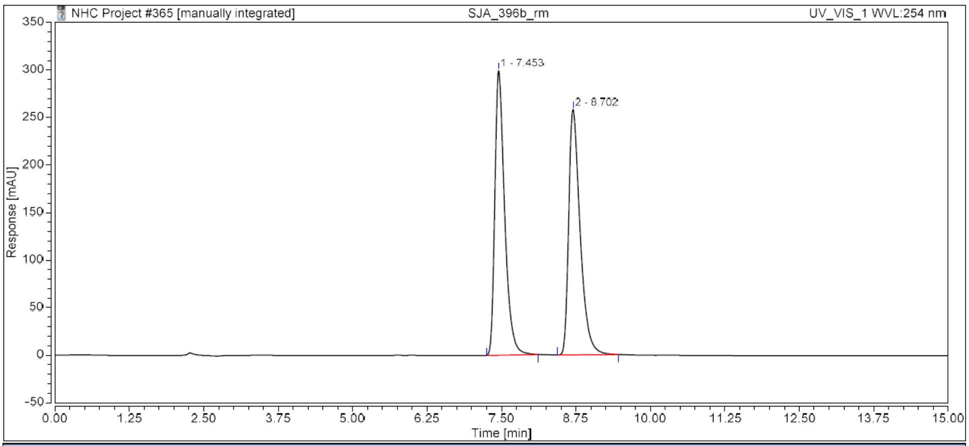

Integration Results

No.	Peak Name	Retention Time min	Area mAU*min	Height mAU	Relative Area %	Relative Height %	Amount n.a.
1		7.453	57.535	299.415	49.99	53.69	n.a.
2		8.702	57.556	258.290	50.01	46.31	n.a.
**Total:**			**115.091**	**557.705**	**100.00**	**100.00**	

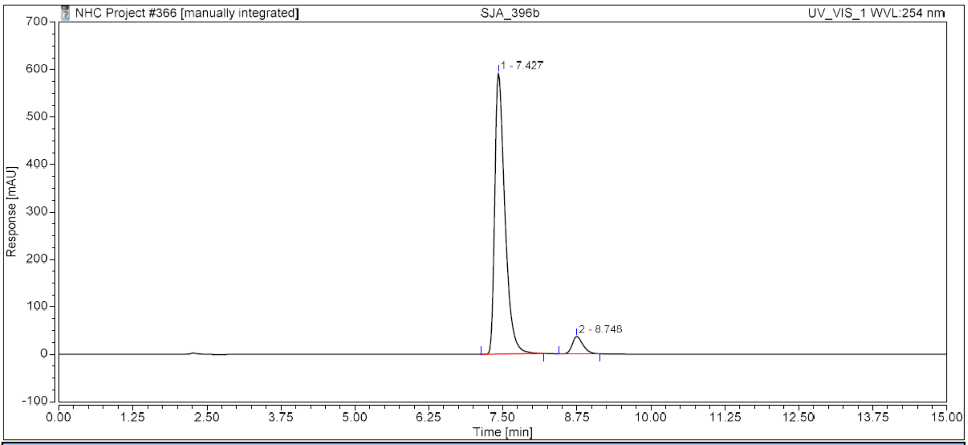

Integration Results							
No.	Peak Name	Retention Time min	Area mAU*min	Height mAU	Relative Area %	Relative Height %	Amount n.a.
1		7.427	117.742	592.509	93.89	94.19	n.a.
2		8.748	7.667	36.554	6.11	5.81	n.a.
Total:			125.409	629.063	100.00	100.00	

(*S*)-4,4,4-Trifluoro-1-(naphthalen-2-yl)-2-phenylbutan-1-one (3h)

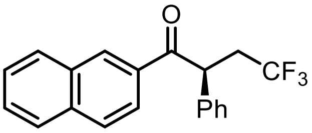

The title compound was synthesized according to the general procedure (**GP 7**) and was obtained after silica gel column chromatography (*n*-pentane : ethylacetate 40:1) as a colorless oil (71 % Yield, 23 mg). $\mathbf{R_f}$ = 0.32 (*n*-pentane : ethylacetate 40:1).

^{1}H NMR (400 MHz, Chloroform-*d*): δ = 8.50 (d, *J* = 1.8 Hz, 1H), 8.01 (dd, *J* = 8.7, 1.8 Hz, 1H), 7.95 – 7.90 (m, 1H), 7.86 – 7.80 (m, 2H), 7.61 – 7.50 (m, 2H), 7.40 – 7.35 (m, 2H), 7.35 – 7.28 (m, 2H), 7.26 – 7.20 (m, 1H), 5.07 (dd, *J* = 7.7, 5.4 Hz, 1H), 3.38 (dqd, *J* = 15.1, 10.8, 7.7 Hz, 1H), 2.68 – 2.54 (m, 1H) ppm.

^{13}C NMR (101 MHz, Chloroform-*d*): δ = 196.7, 137.5, 135.6, 133.0, 132.4, 130.7, 129.7, 129.3, 128.7, 128.5, 128.0, 127.8, 127.7, 126.8, 124.3, 126.4 (q, *J* = 277.2 Hz), 47.27 (q, *J* = 2.4 Hz), 37.44 (q, *J* = 28.2 Hz) ppm.

^{19}F NMR (376 MHz, Chloroform-*d*): δ = -65.05 (t, *J* = 10.6 Hz) ppm.

HRMS (ESI/QTOF) m/z: $[M + Na]^+$ Calcd for $C_{20}H_{15}F_3NaO^+$ 351.0967; Found 351.0959

IR (ATR): 1678, 1626, 1332, 1125, 699, 749 cm^{-1}.

$[\alpha]_D^{20}$ = -27.4 (c = 1, $CHCl_3$).

Chiral HPLC: (Chiralpak IB, 1 % *i*PrOH/hexane, 1.0 mL/min, 210 nm): tR (major) 3.82 min, tR (minor) 5.04 min, 96:4 *er*.

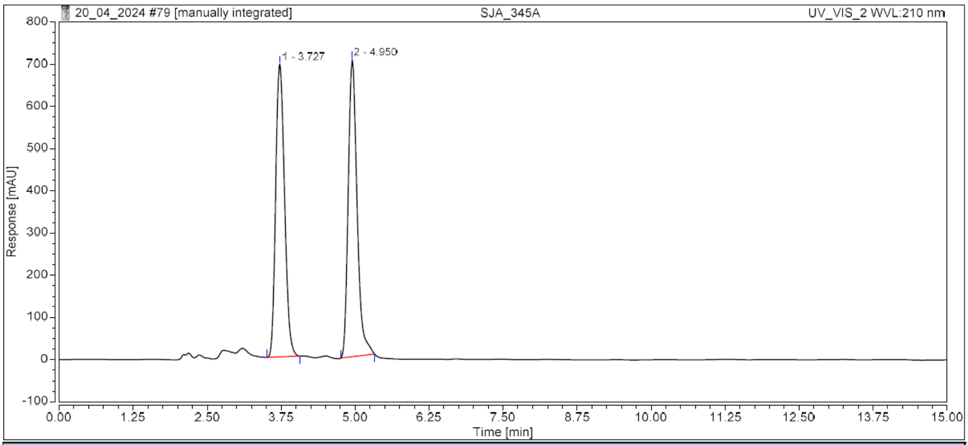

Integration Results

No.	Peak Name	Retention Time min	Area mAU*min	Height mAU	Relative Area %	Relative Height %	Amount n.a.
1		3.727	120.279	694.550	49.56	49.65	n.a.
2		4.950	122.428	704.425	50.44	50.35	n.a.
**Total:**			**242.707**	**1398.975**	**100.00**	**100.00**	

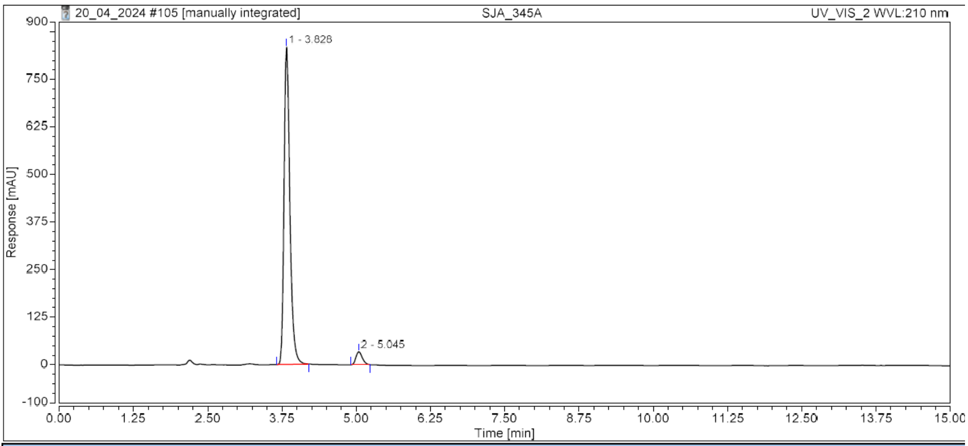

Integration Results

No.	Peak Name	Retention Time min	Area mAU*min	Height mAU	Relative Area %	Relative Height %	Amount n.a.
1		3.828	95.281	834.049	95.78	96.07	n.a.
2		5.045	4.203	34.107	4.22	3.93	n.a.
**Total:**			**99.484**	**868.156**	**100.00**	**100.00**	

(*S*)-4,4,4-Trifluoro-2-phenyl-1-(pyridin-3-yl)butan-1-one (3i)

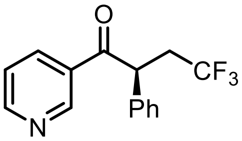

The title compound was synthesized according to the general procedure (**GP 7**) and was obtained after silica gel column chromatography (*n*-pentane : ethylacetate 20:1 to 9:1) as a yellow oil (66 % Yield, 18.5 mg). $\mathbf{R_f}$ = 0.26 (*n*-pentane : ethylacetate 9:1).

^{1}H NMR (400 MHz, Chloroform-*d*): δ = 9.10 (dd, *J* = 2.3, 0.9 Hz, 1H), 8.64 (dd, *J* = 4.8, 1.7 Hz, 1H), 8.14 (ddd, *J* = 8.0, 2.3, 1.8 Hz, 1H), 7.36 – 7.14 (m, 6H), 4.78 (dd, *J* = 7.7, 5.4 Hz, 1H), 3.34 – 3.18 (m, 1H), 2.56 – 2.41 (m, 1H) ppm.

^{13}C NMR (101 MHz, Chloroform-*d*): δ = 195.7, 153.7, 150.3, 136.6, 136.2, 131.1, 129.7, 128.3, 128.2, 126.3 (q, *J* = 277.2 Hz), 123.8, 47.9 (q, *J* = 2.5 Hz), 37.2 (q, *J* = 28.4 Hz) ppm.

^{19}F NMR (376 MHz, Chloroform-*d*): δ = -65.12 (t, *J* = 10.5 Hz) ppm.

HRMS (ESI/QTOF) m/z: $[M + H]^+$ Calcd for $C_{15}H_{13}F_3NO^+$ 280.0944; Found 280.0952.

IR (ATR): 1689, 1585, 1260, 1138, 700 cm^{-1}.

$[\alpha]_D^{20}$ = +47.4 (c = 0.5, $CHCl_3$).

Chiral HPLC: (Chiralpak IC, 3 % *i*PrOH/hexane, 1.0 mL/min, 210 nm): tR (minor) 7.58 min, tR (major) 8.15 min, 91:9 *er*.

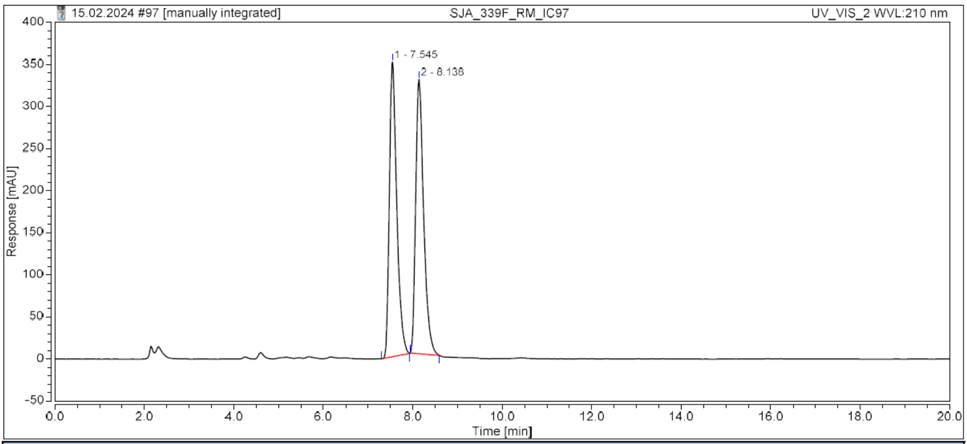

Integration Results							
No.	Peak Name	Retention Time min	Area mAU*min	Height mAU	Relative Area %	Relative Height %	Amount n.a.
1		7.545	67.279	350.123	50.11	51.78	n.a.
2		8.138	66.980	326.090	49.89	48.22	n.a.
Total:			134.258	676.213	100.00	100.00	

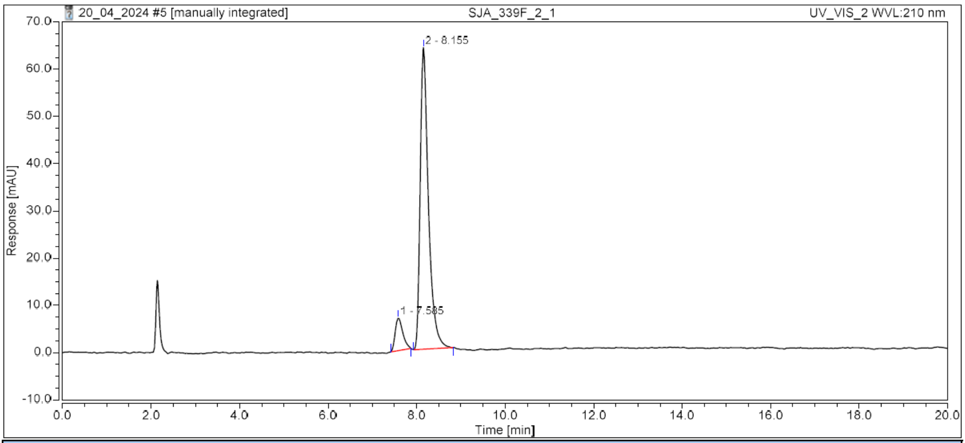

Integration Results							
No.	Peak Name	Retention Time min	Area mAU*min	Height mAU	Relative Area %	Relative Height %	Amount n.a.
1		7.585	1.344	6.922	8.88	9.78	n.a.
2		8.155	13.785	63.858	91.12	90.22	n.a.
Total:			15.129	70.780	100.00	100.00	

(*S*)-1-(9-Ethyl-9*H*-carbazol-3-yl)-4,4,4-trifluoro-2-phenylbutan-1-one (3j)

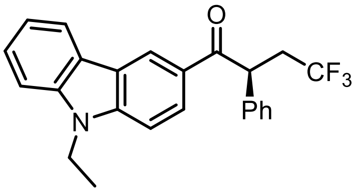

The title compound was synthesized according to the general procedure (**GP 7**) and was obtained after silica gel column chromatography (*n*-pentane : ethylacetate 20:1 to 9:1) as a colorless solid (70 % Yield, 28 mg). **R**$_f$ = 0.24 (*n*-pentane : ethylacetate 9:1).

MP: 140-141 °C

[1]**H NMR** (400 MHz, Chloroform-*d*): δ = 8.78 (d, *J* = 1.7 Hz, 1H), 8.16 – 8.10 (m, 2H), 7.51 (ddd, *J* = 8.3, 7.1, 1.2 Hz, 1H), 7.44 – 7.39 (m, 3H), 7.38 – 7.28 (m, 4H), 7.25 – 7.19 (m, 1H), 5.09 (dd, *J* = 7.6, 5.6 Hz, 1H), 4.35 (q, *J* = 7.2 Hz, 2H), 3.47 – 3.31 (m, 1H), 2.62 (dqd, *J* = 15.0, 10.8, 5.6 Hz, 1H), 1.42 (t, *J* = 7.3 Hz, 3H) ppm.

[13]**C NMR** (101 MHz, Chloroform-*d*): δ = 196.1, 142.9, 140.7, 138.5, 129.6 (q, *J* = 243.7 Hz), 129.3, 128.1, 127.7, 127.0, 126.7, 125.3, 123.3, 123.0, 122.6, 120.8, 120.2, 109.1, 108.3, 47.1 (q, *J* = 2.5 Hz), 37.9, 37.7 (q, *J* = 28.1 Hz), 13.9 ppm.

[19]**F NMR** (376 MHz, Chloroform-*d*): δ = -64.97 (t, *J* = 10.8 Hz) ppm.

HRMS (ESI/QTOF) m/z: $[M + Na]^+$ Calcd for $C_{24}H_{20}F_3NNaO^+$ 418.1389; Found 418.1393.

IR (ATR): 1668, 1625, 1232, 1131, 784 cm^{-1}.

$[\alpha]_D^{20}$ = +41.4 (c = 0.5, $CHCl_3$).

Chiral HPLC: (Chiralpak IC, 3 % *i*PrOH/hexane, 1.0 mL/min, 210 nm): tR (minor) 5.91 min, tR (major) 6.49 min, 94:6 *er*.

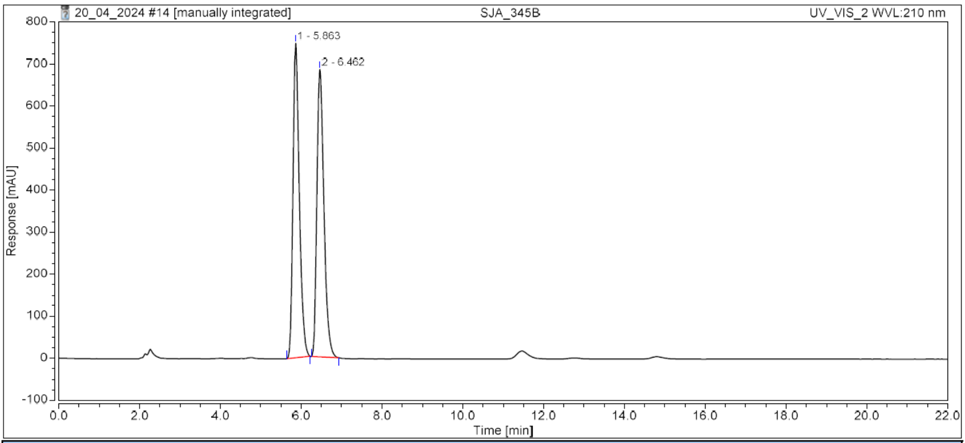

Integration Results							
No.	Peak Name	Retention Time min	Area mAU*min	Height mAU	Relative Area %	Relative Height %	Amount n.a.
1		5.863	136.700	748.395	50.01	52.24	n.a.
2		6.462	136.653	684.336	49.99	47.76	n.a.
Total:			273.353	1432.731	100.00	100.00	

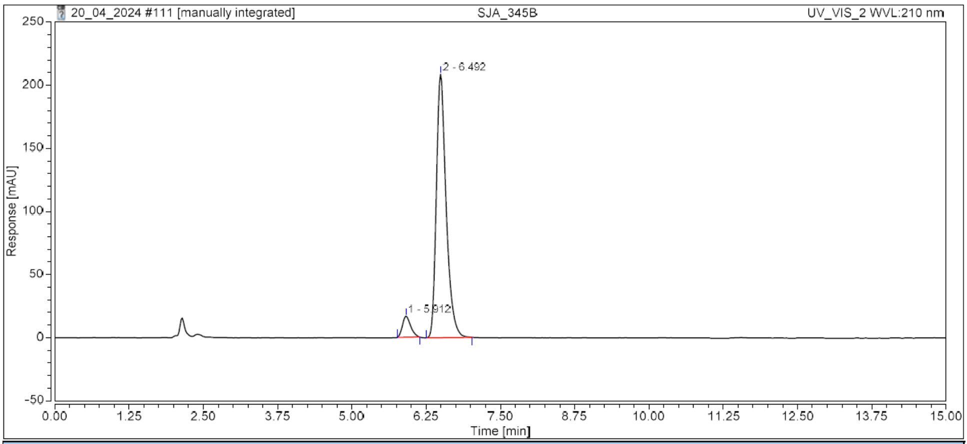

Integration Results							
No.	Peak Name	Retention Time min	Area mAU*min	Height mAU	Relative Area %	Relative Height %	Amount n.a.
1		5.912	2.686	16.749	6.28	7.43	n.a.
2		6.492	40.092	208.809	93.72	92.57	n.a.
Total:			42.778	225.558	100.00	100.00	

(*S*)-4,4,4-Trifluoro-1-(furan-2-yl)-2-phenylbutan-1-one (3k)

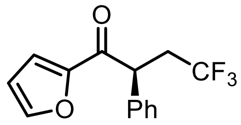

The title compound was synthesized according to the general procedure (**GP 7**) and was obtained after silica gel column chromatography (*n*-pentane : ethylacetate 30:1 to 20:1) as a colorless oil (78 % Yield, 21 mg). $\mathbf{R_f}$ = 0.30 (*n*-pentane : ethylacetate 20:1).

^{1}H NMR (400 MHz, Chloroform-*d*): δ = 7.54 (dd, *J* = 1.7, 0.8 Hz, 1H), 7.36 – 7.19 (m, 6H), 6.48 (dd, *J* = 3.6, 1.7 Hz, 1H), 4.72 (dd, *J* = 8.1, 5.4 Hz, 1H), 3.27 (dqd, *J* = 15.0, 10.7, 8.1 Hz, 1H), 2.52 (dqd, *J* = 14.9, 10.7, 5.4 Hz, 1H) ppm.

^{13}C NMR (101 MHz, Chloroform-*d*): δ = 185.8, 151.6, 147.0, 137.1, 129.2, 128.2, 128.0, 126.4 (q, *J* = 277.1 Hz), 118.7, 112.7, 47.3 (q, *J* = 2.6 Hz), 36.5 (q, *J* = 28.5 Hz) ppm.

^{19}F NMR (376 MHz, Chloroform-*d*): δ = -65.35 (t, *J* = 10.7 Hz) ppm.

HRMS (ESI/QTOF): *m/z*: $[M + Na]^+$ Calcd for $C_{14}H_{11}F_3NaO_2^+$ 291.0603; Found 291.0597.

IR (ATR): 1672, 1597, 1464, 1261, 1137, 1114, 698 cm^{-1}.

$[\alpha]_D^{20}$ = +32.4 (c = 0.5, $CHCl_3$).

Chiral HPLC: (Chiralpak IB, 1 % *i*PrOH/hexane, 1.0 mL/min, 210 nm): tR (major) 4.19 min, tR (minor) 4.81 min, 96.5:3.5 *er*.

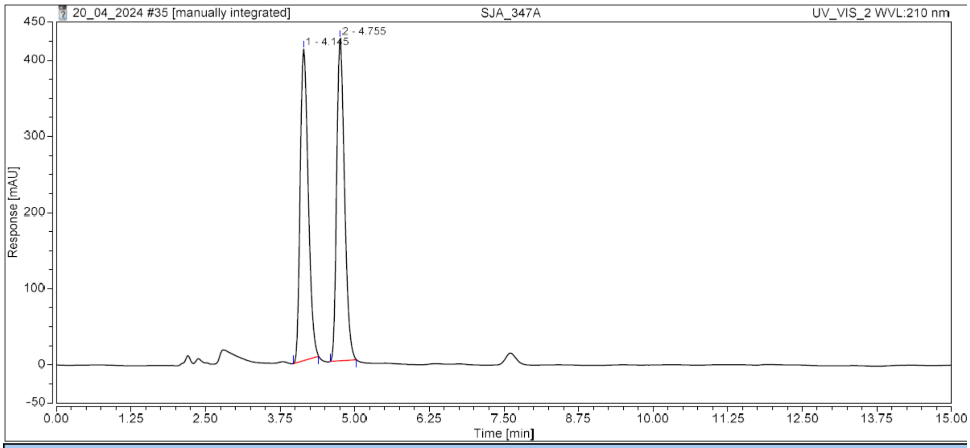

Integration Results

No.	Peak Name	Retention Time min	Area mAU*min	Height mAU	Relative Area %	Relative Height %	Amount n.a.
1		4.145	63.919	409.014	49.87	49.17	n.a.
2		4.755	64.256	422.874	50.13	50.83	n.a.
**Total:**			**128.175**	**831.888**	**100.00**	**100.00**	

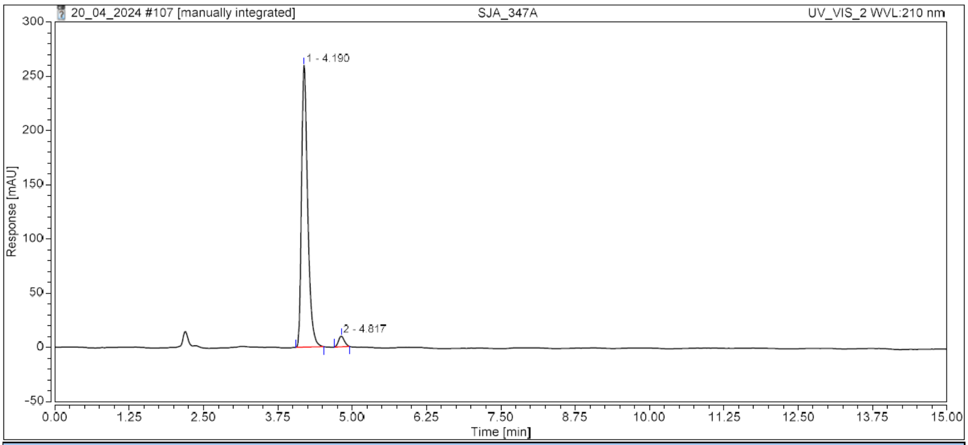

Integration Results

No.	Peak Name	Retention Time min	Area mAU*min	Height mAU	Relative Area %	Relative Height %	Amount n.a.
1		4.190	30.994	259.991	96.50	96.33	n.a.
2		4.817	1.123	9.918	3.50	3.67	n.a.
**Total:**			**32.117**	**269.910**	**100.00**	**100.00**	

(*S*)-4,4,4-Trifluoro-2-phenyl-1-(thiophen-2-yl)butan-1-one (3l)

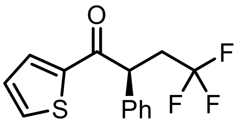

The title compound was synthesized according to the general procedure (**GP 7**) and was obtained after silica gel column chromatography (*n*-pentane : ethylacetate 30:1 to 20:1) as a pale orange oil (81 % Yield, 23 mg). $\mathbf{R_f}$ = 0.32 (*n*-pentane : ethylacetate 20:1).

^{1}H NMR (400 MHz, Chloroform-*d*): δ = 7.75 (dd, *J* = 3.9, 1.1 Hz, 1H), 7.62 (dd, *J* = 4.9, 1.1 Hz, 1H), 7.37 – 7.25 (m, 5H), 7.08 (dd, *J* = 4.9, 3.9 Hz, 1H), 4.71 (dd, *J* = 7.7, 5.5 Hz, 1H), 3.38 – 3.21 (m, 1H), 2.63 – 2.48 (m, 1H) ppm.

^{13}C NMR (101 MHz, Chloroform-*d*): δ = 189.7, 142.8, 137.6, 134.6, 133.0, 129.4, 128.3, 128.13, 128.11, 126.4 (q, *J* = 277.2 Hz), 48.7 (q, *J* = 2.6 Hz), 37.2 (q, *J* = 28.4 Hz) ppm.

^{19}F NMR (376 MHz, Chloroform-*d*): δ = -65.19 (t, *J* = 10.5 Hz) ppm.

HRMS (ESI/QTOF): *m/z*: $[M + H]^+$ Calcd for $C_{14}H_{12}F_3OS^+$ 285.0555; Found 285.0556.

IR (ATR): 1663, 1413, 1260, 1139, 1114, 750, 724 cm^{-1}.

$[\alpha]_D^{20}$ = +36.2 (c = 0.5, $CHCl_3$).

Chiral HPLC: (Chiralpak IB, 1 % *i*PrOH/hexane, 1.0 mL/min, 210 nm): tR (major) 4.09 min, tR (minor) 5.43 min, 96:4 *er*.

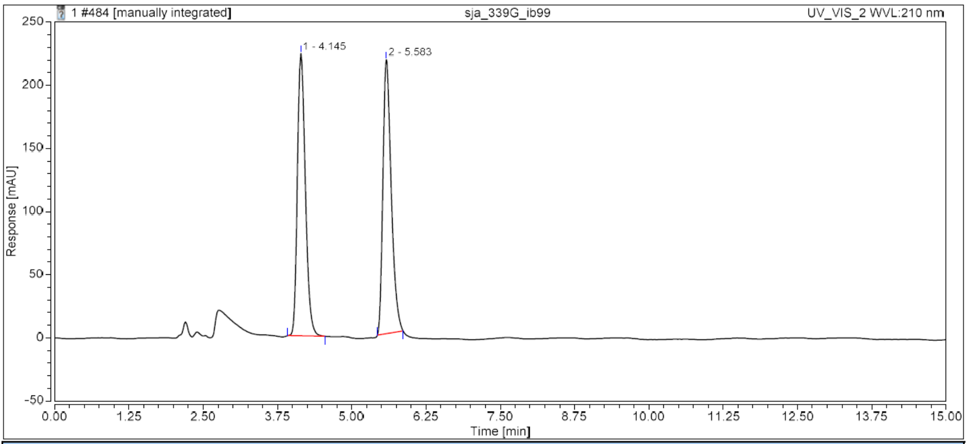

Integration Results

No.	Peak Name	Retention Time min	Area mAU*min	Height mAU	Relative Area %	Relative Height %	Amount n.a.
1		4.145	34.866	223.508	49.59	50.74	n.a.
2		5.583	35.449	216.985	50.41	49.26	n.a.
**Total:**			**70.315**	**440.494**	**100.00**	**100.00**	

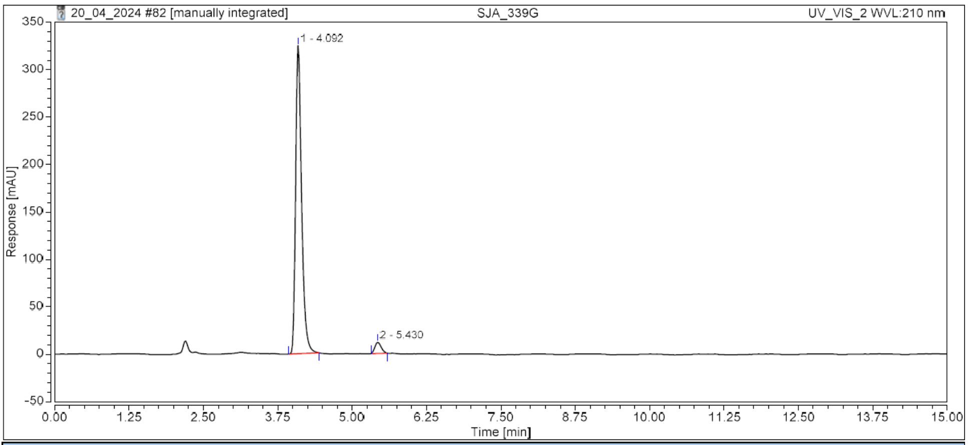

Integration Results

No.	Peak Name	Retention Time min	Area mAU*min	Height mAU	Relative Area %	Relative Height %	Amount n.a.
1		4.092	38.034	324.839	96.39	96.51	n.a.
2		5.430	1.425	11.748	3.61	3.49	n.a.
**Total:**			**39.459**	**336.587**	**100.00**	**100.00**	

(*S*)-6,6,6-Trifluoro-4-(4-methoxyphenyl)hexan-3-one (3m)

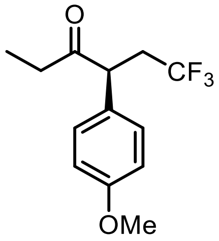

The title compound was synthesized according to the general procedure (**GP 7**) and was obtained after silica gel column chromatography (*n*-pentane : ethylacetate 30:1 to 20:1) as a colorless oil (62 % Yield, 16 mg). $\mathbf{R_f}$ = 0.30 (*n*-pentane : ethylacetate 30:1).

^{1}H NMR (400 MHz, Chloroform-*d*): δ = 7.14 – 7.09 (m, 2H), 6.90 – 6.85 (m, 2H), 3.92 (t, *J* = 6.7 Hz, 1H), 3.80 (s, 3H), 3.11 (dqd, *J* = 15.0, 10.9, 7.j3 Hz, 1H), 2.51 – 2.27 (m, 3H), 0.98 (t, *J* = 7.3 Hz, 3H) ppm.

^{13}C NMR (101 MHz, Chloroform-*d*): δ = 208.0, 159.2, 129.1, 126.4 (q, *J* = 277.0 Hz), 114.6, 55.2, 50.9 (q, *J* = 2.4 Hz), 36.1 (q, *J* = 28.1 Hz), 34.6, 7.9 ppm.

^{19}F NMR (376 MHz, Chloroform-*d*): δ = -65.26 (t, *J* = 10.9 Hz) ppm.

HRMS (APCI/QTOF): *m/z*: [M + H]$^+$ Calcd for $C_{13}H_{16}F_3O_2^+$ 261.1097; Found 261.1102.

IR (ATR): 1719, 1611, 1512, 1248, 1136, 1110, 807 cm^{-1}.

$[\alpha]_D^{20}$ = +12.2 (c = 0.5, $CHCl_3$).

Chiral HPLC: (Chiralpak IA, 0.2 % *i*PrOH/hexane, 1.0 mL/min, 254 nm): tR (minor) 17.38 min, tR (major) 23.11 min, 94.5:5.5 *er*.

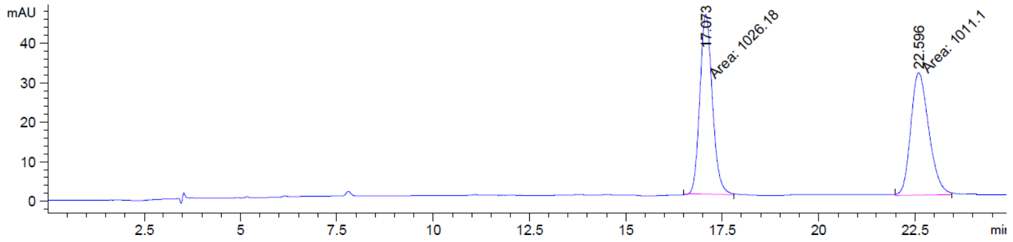

Peak #	RetTime [min]	Type	Width [min]	Area [mAU*s]	Height [mAU]	Area %
1	17.073	MM	0.3758	1026.17969	45.51066	50.3700
2	22.596	MM	0.5443	1011.10358	30.96145	49.6300

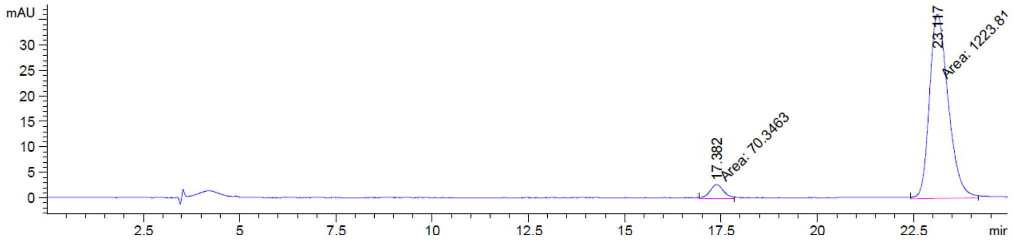

Peak #	RetTime [min]	Type	Width [min]	Area [mAU*s]	Height [mAU]	Area %
1	17.382	MM	0.4207	70.34632	2.78699	5.4357
2	23.117	MM	0.5646	1223.81226	36.12678	94.5643

(*S*)-1-Cyclohexyl-4,4,4-trifluoro-2-phenylbutan-1-one (3n)

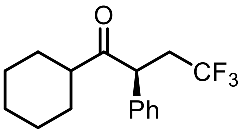

The title compound was synthesized according to the general procedure (**GP 7**) and was obtained after silica gel column chromatography (*n*-pentane : ethylacetate 60:1 to 40:1) as a colorless oil (67 % Yield, 19 mg). **R$_f$** = 0.40 (*n*-pentane : ethylacetate 60:1).

^{1}H NMR (400 MHz, Chloroform-*d*): δ = 7.36 – 7.26 (m, 3H), 7.22 – 7.18 (m, 2H), 4.12 (dd, *J* = 8.0, 5.2 Hz, 1H), 3.14 (dqd, *J* = 15.0, 10.8, 7.9 Hz, 1H), 2.45 – 2.23 (m, 2H), 1.96 – 1.86 (m, 1H), 1.83 – 1.73 (m, 1H), 1.68 – 1.57 (m, 2H), 1.42 – 1.33 (m, 2H), 1.30 – 0.99 (m, 4H) ppm.

^{13}C NMR (101 MHz, Chloroform-*d*): δ = 210.0, 137.0, 129.3, 128.3, 128.0, 126.4 (q, *J* = 277.1 Hz), 50.3 (q, *J* = 2.5 Hz), 49.8, 36.5 (q, *J* = 28.2 Hz), 29.3, 28.2, 26.0, 25.7, 25.2 ppm.

^{19}F NMR (376 MHz, Chloroform-*d*): δ = -65.38 (t, *J* = 10.8 Hz) ppm.

HRMS (Sicrit plasma/LTQ-Orbitrap): *m/z*: [M + H]$^+$ Calcd for $C_{16}H_{20}F_3O^+$ 285.1461; Found 285.1460.

IR (ATR): 2932, 2856, 1713, 1257, 1137, 700 cm^{-1}.

$[\alpha]_D^{20}$ = +16.4 (c = 0.5, $CHCl_3$).

Chiral HPLC: (Chiralpak IC, 0.3 % *i*PrOH/hexane, 1.0 mL/min, 210 nm): tR (major) 3.36 min, tR (minor) 3.67 min, 97:3 *er*.

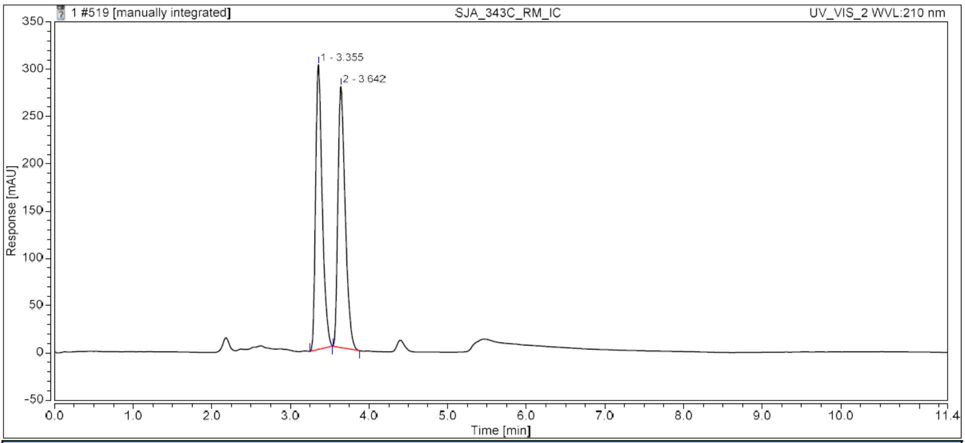

Integration Results							
No.	Peak Name	Retention Time min	Area mAU*min	Height mAU	Relative Area %	Relative Height %	Amount n.a.
1		3.355	29.274	301.167	49.76	52.15	n.a.
2		3.642	29.554	276.290	50.24	47.85	n.a.
Total:			58.828	577.458	100.00	100.00	

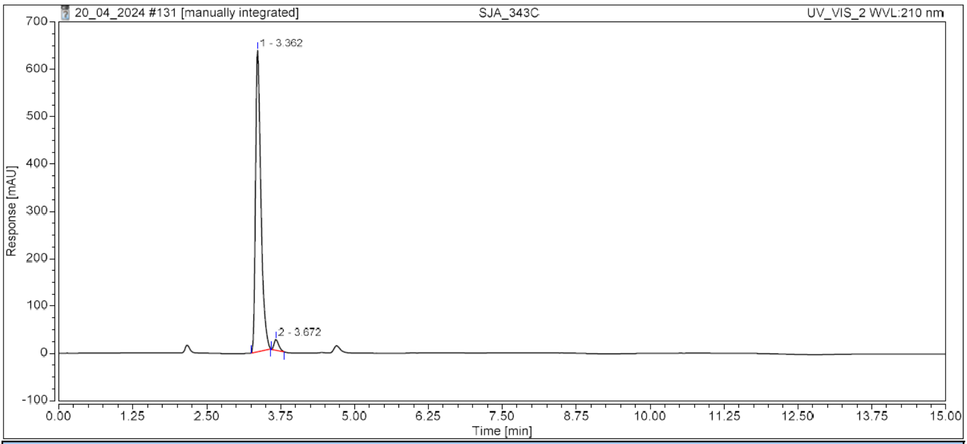

Integration Results							
No.	Peak Name	Retention Time min	Area mAU*min	Height mAU	Relative Area %	Relative Height %	Amount n.a.
1		3.362	67.568	636.473	96.97	96.55	n.a.
2		3.672	2.115	22.775	3.03	3.45	n.a.
Total:			69.683	659.248	100.00	100.00	

(*S*)-1-Cyclopropyl-4,4,4-trifluoro-2-phenylbutan-1-one (3o)

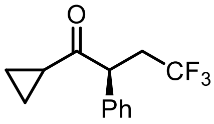

The title compound was synthesized according to the general procedure (**GP 7**) and was obtained after silica gel column chromatography (*n*-pentane : ethylacetate 30:1 to 20:1) as a pale orange oil (61 % Yield, 15 mg). $\mathbf{R_f}$ = 0.38(*n*-pentane : ethylacetate 50:1).

^{1}H NMR (400 MHz, Chloroform-*d*): δ = 7.39 – 7.28 (m, 3H), 7.25 – 7.20 (m, 2H), 4.13 (t, *J* = 6.6 Hz, 1H), 3.15 (dqd, *J* = 15.1, 11.0, 6.6 Hz, 1H), 2.38 (dqd, *J* = 15.1, 10.7, 6.6 Hz, 1H), 1.86 (tt, *J* = 7.8, 4.5 Hz, 1H), 1.09 – 0.94 (m, 2H), 0.92 – 0.84 (m, 1H), 0.77 – 0.69 (m, 1H) ppm.

^{13}C NMR (101 MHz, Chloroform-*d*): δ = 207.2, 137.3, 129.3, 128.4, 128.0, 126.6 (q, *J* = 277.0 Hz), 53.0 (q, *J* = 2.4 Hz), 36.0 (q, *J* = 28.3 Hz), 20.49, 12.1, 11.7 ppm.

^{19}F NMR (376 MHz, Chloroform-*d*): δ = -65.08 (t, *J* = 11.0 Hz) ppm.

HRMS (Sicrit plasma/LTQ-Orbitrap): *m/z*: $[M + H]^+$ Calcd for $C_{13}H_{14}F_3O^+$ 243.0991; Found 243.0991.

IR (ATR): 1701, 1493, 1455, 1253, 1123, 1040, 699 cm^{-1}.

$[\alpha]_D^{20}$ = +13.1 (c = 0.5, $CHCl_3$).

Chiral HPLC: (Chiralpak IC, 0.2 % *i*PrOH/hexane, 1.0 mL/min, 210 nm): tR (major) 10.43 min, tR (minor) 12.39 min, 95:5 *er*.

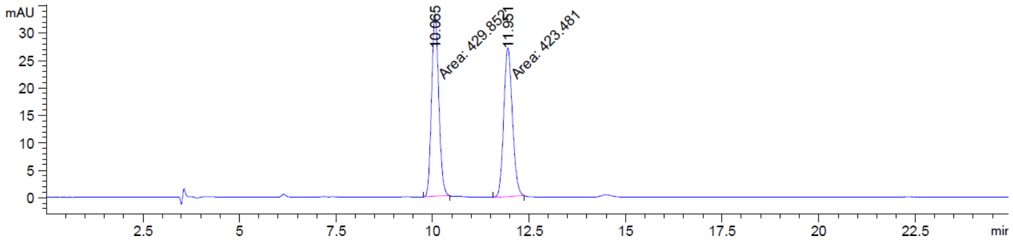

Peak #	RetTime [min]	Type	Width [min]	Area [mAU*s]	Height [mAU]	Area %
1	10.065	MM	0.2161	429.85239	33.15789	50.3733
2	11.951	MM	0.2604	423.48062	27.10389	49.6267

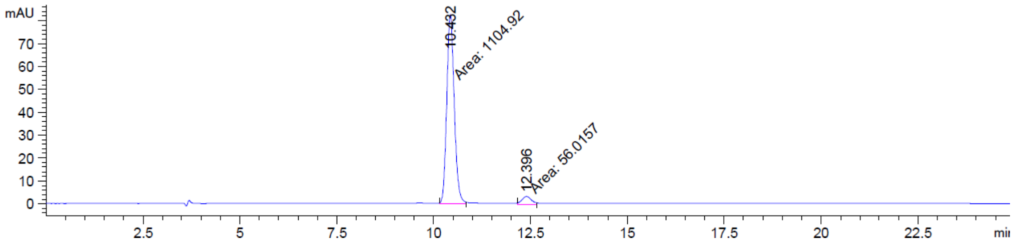

Peak #	RetTime [min]	Type	Width [min]	Area [mAU*s]	Height [mAU]	Area %
1	10.432	MM	0.2235	1104.92078	82.40814	95.1750
2	12.396	MM	0.2782	56.01573	3.35535	4.8250

(*S*)-4,4,4-Trifluoro-2-(4-methoxyphenyl)-1-phenylbutan-1-one (4a)

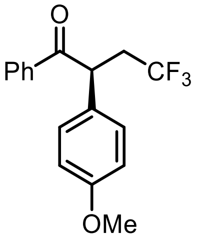

The title compound was synthesized according to the general procedure (**GP 7**) and was obtained after silica gel column chromatography (*n*-pentane : ethylacetate 30:1 to 20:1) as a colorless oil (72 % Yield, 22 mg). **R_f** = 0.26 (*n*-pentane : ethylacetate 30:1).

^{1}H NMR (400 MHz, Chloroform-*d*): δ = 7.99 – 7.90 (m, 2H), 7.53 – 7.47 (m, 1H), 7.43 – 7.37 (m, 2H), 7.24 – 7.19 (m, 2H), 6.86 – 6.81 (m, 2H), 4.86 (dd, *J* = 7.6, 5.6 Hz, 1H), 3.75 (s, 3H), 3.26 (dqd, *J* = 15.0, 10.8, 7.6 Hz, 1H), 2.52 (dqd, *J* = 15.0, 10.8, 5.6 Hz, 1H) ppm.

^{13}C NMR (101 MHz, Chloroform-*d*): δ = 197.0, 159.2, 135.8, 133.4, 129.4, 129.3, 128.9, 128.7, 126.5 (q, *J* = 277.2 Hz), 114.8, 55.3, 46.4 (q, *J* = 2.5 Hz), 37.5 (q, *J* = 28.0 Hz) ppm.

^{19}F NMR (376 MHz, Chloroform-*d*): δ = -65.05 (t, *J* = 11.0 Hz) ppm.

HRMS (ESI/QTOF) m/z: $[M + H]^+$ Calcd for $C_{17}H_{16}F_3O_2^+$ 309.1097; Found 309.1108.

IR(ATR): 2060, 1683, 1610, 1287, 1249, 974, 877 cm^{-1}.

$[\alpha]_D^{20}$ = +43.2 (c = 0.5, $CHCl_3$).

Chiral HPLC: (Chiralpak IB, 0.5 % *i*PrOH/hexane, 1.0 mL/min, 210 nm): tR (minor) 11.68 min, tR (major) 13.11 min, 95:5 *er*

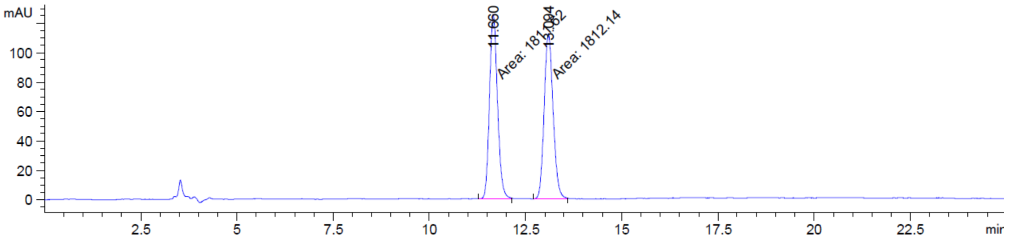

Peak #	RetTime [min]	Type	Width [min]	Area [mAU*s]	Height [mAU]	Area %
1	11.660	MM	0.2407	1811.62219	125.45358	49.9929
2	13.094	MM	0.2693	1812.13794	112.17067	50.0071

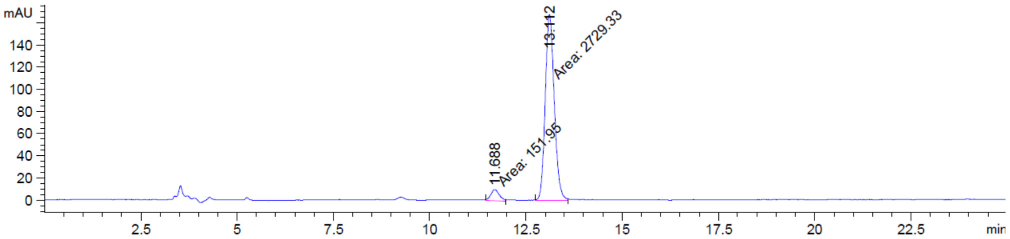

Peak #	RetTime [min]	Type	Width [min]	Area [mAU*s]	Height [mAU]	Area %
1	11.688	MM	0.2533	151.94998	9.99802	5.2737
2	13.112	MM	0.2714	2729.32983	167.63290	94.7263

(*S*)-2-([1,1'-Biphenyl]-4-yl)-4,4,4-trifluoro-1-phenylbutan-1-one (4b)

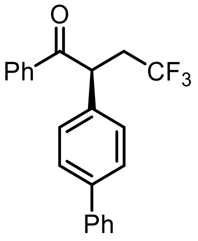

The title compound was synthesized according to the general procedure (**GP 7**) and was obtained after silica gel column chromatography (*n*-pentane : ethylacetate 30:1 to 20:1) as a colorless solid (80 % Yield, 28 mg). $\mathbf{R_f}$ = 0.28 (*n*-pentane : ethylacetate 30:1).

^{1}H NMR (400 MHz, Chloroform-*d*): δ = 7.94 – 7.89 (m, 2H), 7.48 – 7.40 (m, 5H), 7.37 – 7.27 (m, 6H), 7.27 – 7.22 (m, 1H), 4.88 (dd, *J* = 7.8, 5.4 Hz, 1H), 3.26 (dqd, *J* = 15.0, 10.8, 7.7 Hz, 1H), 2.50 (dqd, *J* = 15.0, 10.8, 5.4 Hz, 1H) ppm.

^{13}C NMR (101 MHz, Chloroform-*d*): δ = 196.7, 140.7, 140.2, 136.3, 135.7, 133.4, 128.88, 128.82, 128.7, 128.4, 128.0, 127.5, 127.0, 126.4 (q, *J* = 277.3 Hz), 46.8 (q, *J* = 2.5 Hz), 37.3 (q, *J* = 28.2 Hz) ppm.

^{19}F NMR (376 MHz, Chloroform-*d*): δ = -65.09 (t, *J* = 10.8 Hz) ppm.

HRMS (ESI/QTOF) m/z: $[M + Na]^+$ Calcd for $C_{22}H_{17}F_3NaO^+$ 377.1124; Found 377.1119.

IR (ATR): 1684, 1257, 1136, 1100, 689 cm^{-1}.

$[\alpha]_D^{20}$ = +23.2 (c = 1, $CHCl_3$).

Chiral HPLC: (Chiralpak IB, 1 % *i*PrOH/hexane, 1.0 mL/min, 210 nm): tR (major) 3.98 min, tR (minor) 4.03 min, 97:3 *er*.

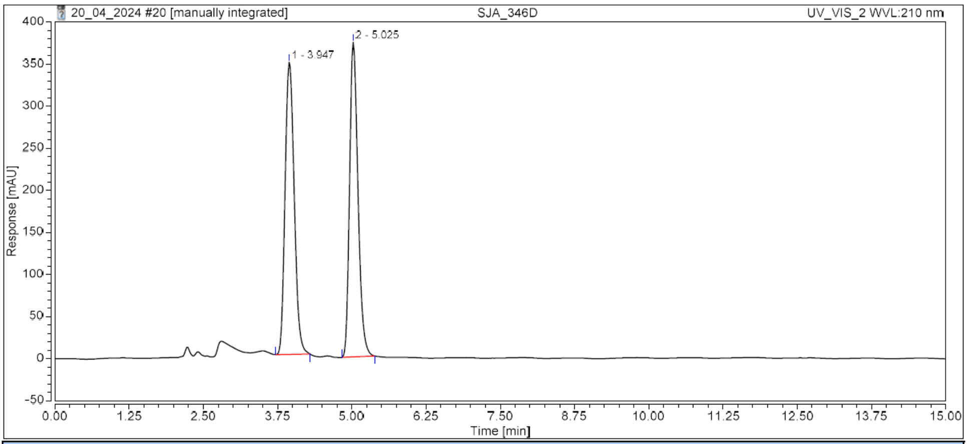

Integration Results							
No.	Peak Name	Retention Time min	Area mAU*min	Height mAU	Relative Area %	Relative Height %	Amount n.a.
1		3.947	61.414	347.131	49.87	48.13	n.a.
2		5.025	61.746	374.120	50.13	51.87	n.a.
Total:			123.160	721.251	100.00	100.00	

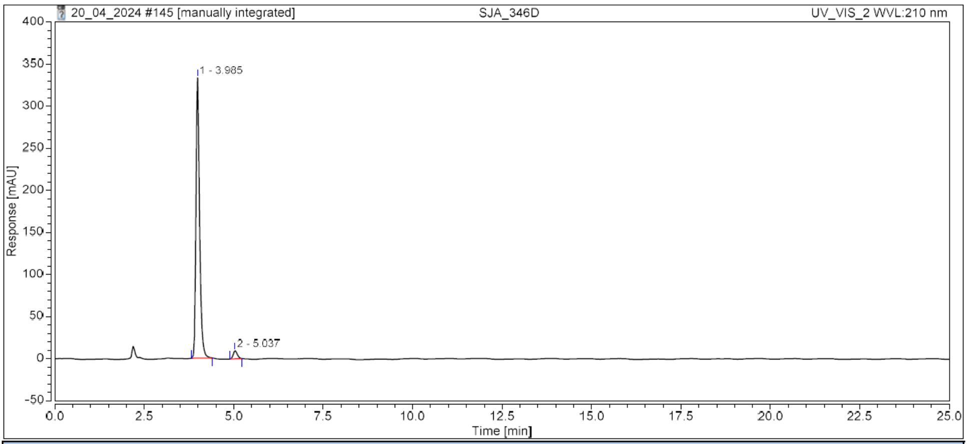

Integration Results							
No.	Peak Name	Retention Time min	Area mAU*min	Height mAU	Relative Area %	Relative Height %	Amount n.a.
1		3.985	39.717	333.093	97.00	97.21	n.a.
2		5.037	1.226	9.573	3.00	2.79	n.a.
Total:			40.944	342.665	100.00	100.00	

(*S*)-4-(4,4,4-Trifluoro-1-oxo-1-phenylbutan-2-yl)benzonitrile (4c)

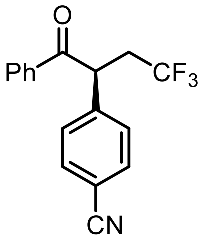

The title compound was synthesized according to the general procedure (**GP 7**) and was obtained after silica gel column chromatography (*n*-pentane : ethylacetate 20:1) as a colorless solid (62 % Yield, 19 mg). $\mathbf{R_f}$ = 0.22 (*n*-pentane : ethylacetate 20:1).

^{1}H NMR (400 MHz, Chloroform-*d*): δ = 7.95 – 7.89 (m, 2H), 7.64 – 7.60 (m, 2H), 7.59 – 7.53 (m, 1H), 7.47 – 7.41 (m, 4H), 4.97 (t, *J* = 6.7 Hz, 1H), 3.25 (dqd, *J* = 15.1, 10.6, 7.0 Hz, 1H), 2.59 (dqd, *J* = 15.1, 10.4, 6.4 Hz, 1H) ppm.

^{13}C NMR (101 MHz, Chloroform-*d*): δ = 195.9, 142.6, 135.2, 134.1, 133.2, 129.1, 129.0, 128.9, 126.2 (q, *J* = 277.2 Hz), 118.3, 112.2, 47.1 (d, *J* = 2.6 Hz), 37.3 (q, *J* = 28.6 Hz) ppm.

^{19}F NMR (376 MHz, Chloroform-*d*): δ = -64.88 (t, *J* = 10.6 Hz) ppm.

HRMS (ESI/QTOF): *m/z*: $[M + H]^+$ Calcd for $C_{17}H_{13}F_3NO^+$ 304.0944; Found 304.0939.

IR (ATR): 1685, 1607, 1258, 1139, 1056, 745 cm^{-1}.

$[\alpha]_D^{20}$ = +37.9 (c = 0.5, $CHCl_3$).

Chiral HPLC: (Chiralpak IB, 1 % *i*PrOH/hexane, 1.0 mL/min, 254 nm): tR (major) 9.24 min, tR (minor) 15.31 min, 92:8 *er*.

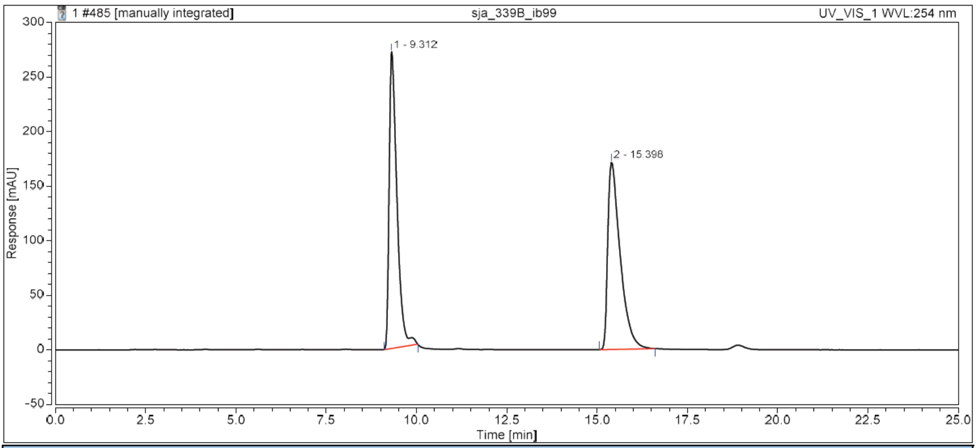

Integration Results							
No.	Peak Name	Retention Time min	Area mAU*min	Height mAU	Relative Area %	Relative Height %	Amount n.a.
1		9.312	68.145	271.851	49.99	61.28	n.a.
2		15.398	68.159	171.779	50.01	38.72	n.a.
Total:			136.304	443.630	100.00	100.00	

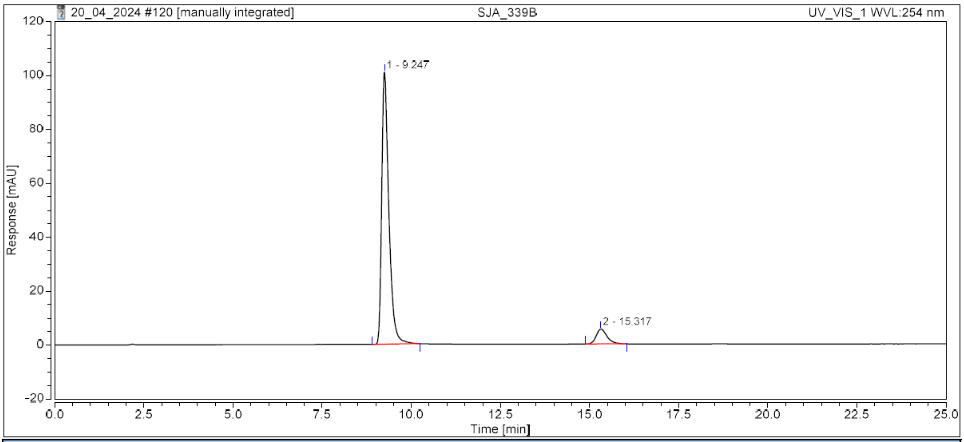

Integration Results							
No.	Peak Name	Retention Time min	Area mAU*min	Height mAU	Relative Area %	Relative Height %	Amount n.a.
1		9.247	23.269	100.957	92.37	94.82	n.a.
2		15.317	1.921	5.516	7.63	5.18	n.a.
Total:			25.190	106.473	100.00	100.00	

(*S*)-4,4,4-Trifluoro-1-phenyl-2-(*o*-tolyl)butan-1-one (4d)

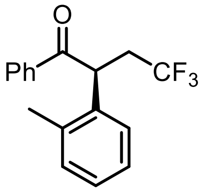

The title compound was synthesized according to the general procedure (**GP 7**) and was obtained after silica gel column chromatography (*n*-pentane : ethylacetate 50:1) as a colorless solid (69 % Yield, 20 mg). $\mathbf{R_f}$ = 0.38 (*n*-pentane : ethylacetate 50:1).

^{1}H NMR (400 MHz, Chloroform-*d*): δ = 7.85 – 7.80 (m, 2H), 7.52 – 7.46 (m, 1H), 7.41 – 7.35 (m, 2H), 7.25 – 7.21 (m, 1H), 7.15 (td, *J* = 7.2, 1.8 Hz, 1H), 7.12 – 7.04 (m, 2H), 5.03 (dd, *J* = 8.5, 4.0 Hz, 1H), 3.39 (dqd, *J* = 15.1, 10.9, 8.5 Hz, 1H), 2.55 (s, 3H), 2.34 (dqd, *J* = 15.0, 10.9, 4.1 Hz, 1H) ppm.

^{13}C NMR (101 MHz, Chloroform-*d*): δ = 197.4, 136.2, 136.1, 135.1, 133.3, 131.6, 128.8, 128.6, 127.9, 127.2, 127.1, 126.5 (q, *J* = 277.3 Hz), 43.6 (q, *J* = 2.4 Hz), 36.7 (q, *J* = 28.3 Hz), 19.7 ppm.

^{19}F NMR (376 MHz, Chloroform-*d*): δ = -65.67 (t, *J* = 11.0 Hz) ppm.

HRMS (ESI/QTOF) m/z: $[M + Na]^+$ Calcd for $C_{17}H_{15}F_3NaO^+$ 315.0967; Found 315.0962.

IR (ATR): 1684, 1306, 1258, 1138, 754, 690 cm^{-1}.

$[\alpha]_D^{20}$ = +33.6 (c = 0.5, $CHCl_3$).

Chiral HPLC: (Chiralpak IF, 0.5 % *i*PrOH/hexane, 1.0 mL/min, 210 nm): tR (major) 5.61 min, tR (major) 6.57 min, 96:4 *er*.

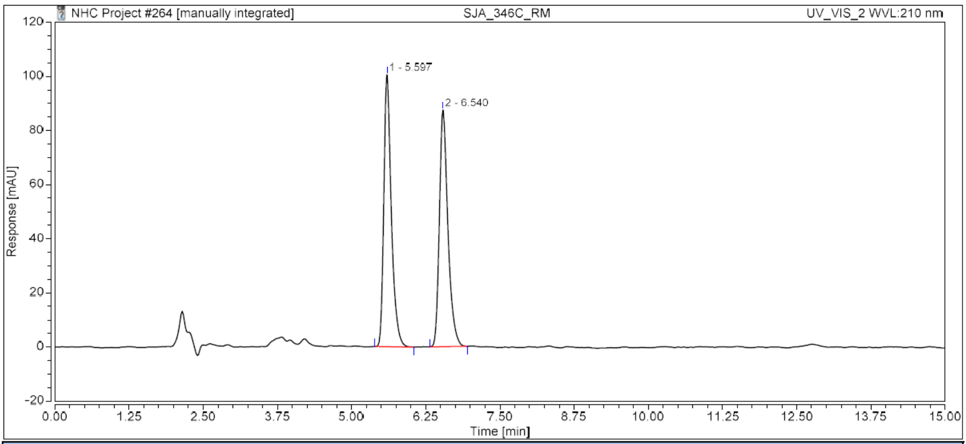

Integration Results

No.	Peak Name	Retention Time min	Area mAU*min	Height mAU	Relative Area %	Relative Height %	Amount n.a.
1		5.597	14.899	100.588	50.32	53.49	n.a.
2		6.540	14.708	87.471	49.68	46.51	n.a.
**Total:**			**29.608**	**188.059**	**100.00**	**100.00**	

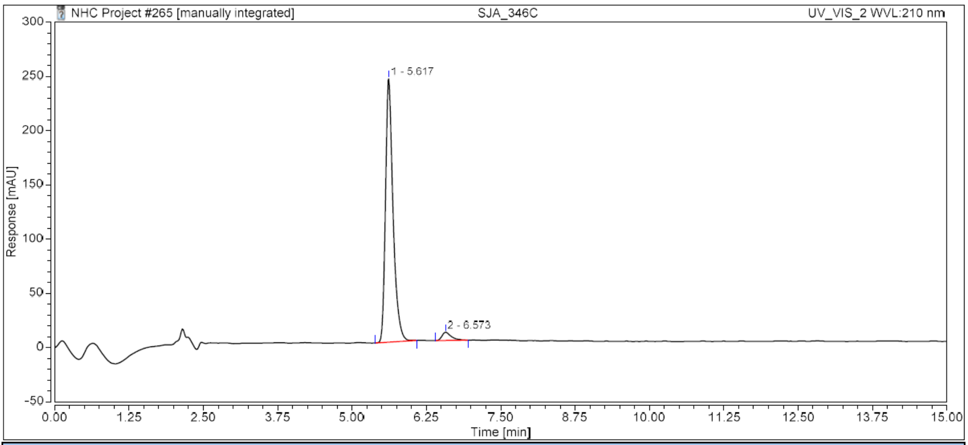

Integration Results							
No.	Peak Name	Retention Time min	Area mAU*min	Height mAU	Relative Area %	Relative Height %	Amount n.a.
1		5.617	36.107	242.904	96.34	96.97	n.a.
2		6.573	1.371	7.584	3.66	3.03	n.a.
Total:			37.478	250.488	100.00	100.00	

(*S*)-2-(1-Benzyl-1*H*-indol-3-yl)-4,4,4-trifluoro-1-phenylbutan-1-one (4e)

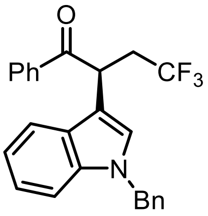

The title compound was synthesized according to the general procedure (**GP 7**) and was obtained after silica gel column chromatography (*n*-pentane : ethylacetate 20:1) as a colorless solid (52 % Yield, 21 mg). **R**$_f$ = 0.26 (*n*-pentane : ethylacetate 20:1).

^{1}H NMR (400 MHz, Chloroform-*d*): δ = 8.00 – 7.94 (m, 2H), 7.79 – 7.71 (m, 1H), 7.52 – 7.45 (m, 1H), 7.41 – 7.33 (m, 2H), 7.26 – 7.16 (m, 6H), 6.98 – 6.92 (m, 3H), 5.28 – 5.15 (m, 3H), 3.39 (dqd, *J* = 15.0, 10.9, 8.3 Hz, 1H), 2.65 (dqd, *J* = 15.6, 11.0, 4.6 Hz, 1H) ppm.

^{13}C NMR (101 MHz, Chloroform-*d*): δ = 196.9, 137.1, 137.0, 136.0, 133.2, 128.8, 128.7, 128.6, 127.8, 127.2, 126.6 (q, *J* = 277.3 Hz), 126.6, 126.4, 122.6, 120.2, 118.7, 111.5, 110.3, 50.2, 38.5 (q, *J* = 2.7 Hz), 36.8 (q, *J* = 27.6 Hz) ppm.

^{19}F NMR (376 MHz, Chloroform-*d*): δ = -65.52 (t, *J* = 10.8 Hz) ppm.

HRMS (ESI/QTOF): *m/z*: $[M + H]^+$ Calcd for $C_{25}H_{21}F_3NO^+$ 408.1570; Found 408.1565.

IR (ATR): 1682, 1459, 1333, 1257, 1137, 740 cm^{-1}.

$[\alpha]_D^{20}$ = +43.2 (c = 0.5, $CHCl_3$).

Chiral HPLC: (Chiralpak IC, 1 % *i*PrOH/hexane, 1.0 mL/min, 210 nm): tR (minor) 4.69 min, tR (major) 5.33 min, 98:2 *er*.

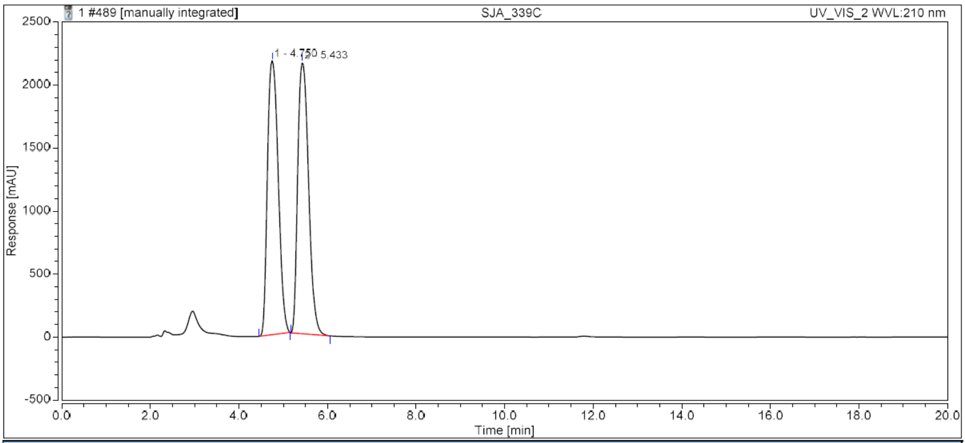

Integration Results							
No.	Peak Name	Retention Time<br>min	Area<br>mAU*min	Height<br>mAU	Relative Area<br>%	Relative Height<br>%	Amount<br>n.a.
1		4.750	611.150	2174.419	49.95	50.25	n.a.
2		5.433	612.401	2152.905	50.05	49.75	n.a.
**Total:**			**1223.551**	**4327.324**	**100.00**	**100.00**	

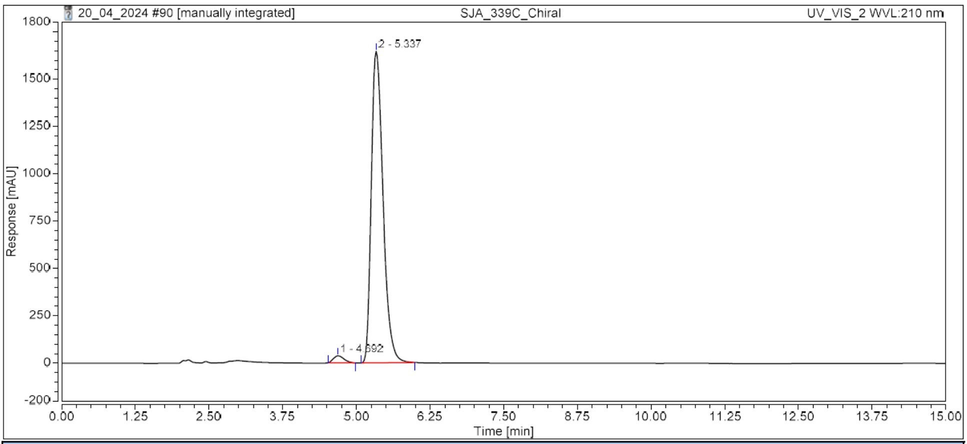

Integration Results							
No.	Peak Name	Retention Time<br>min	Area<br>mAU*min	Height<br>mAU	Relative Area<br>%	Relative Height<br>%	Amount<br>n.a.
1		4.692	7.507	36.676	1.94	2.18	n.a.
2		5.337	379.974	1645.164	98.06	97.82	n.a.
**Total:**			**387.481**	**1681.839**	**100.00**	**100.00**	

(*R*)-4,4,4-Trifluoro-1-phenyl-2-(thiophen-2-yl)butan-1-one (4f)

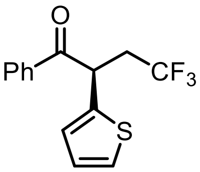

The title compound was synthesized according to the general procedure (**GP 7**) and was obtained after silica gel column chromatography (*n*-pentane : ethylacetate 30:1) as a colorless solid (72 % Yield, 20.5 mg). **R$_f$** = 0.24 (*n*-pentane : ethylacetate 30:1).

^{1}H NMR (400 MHz, Chloroform-*d*): δ = 8.04 – 7.95 (m, 2H), 7.59 – 7.53 (m, 1H), 7.45 (t, *J* = 7.6 Hz, 2H), 7.22 – 7.20 (m, 1H), 6.96 – 6.88 (m, 2H), 5.21 (dd, *J* = 8.3, 4.9 Hz, 1H), 3.43 – 3.25 (m, 1H), 2.72 – 2.55 (m, 1H) ppm.

^{13}C NMR (101 MHz, Chloroform-*d*): δ = 195.6, 139.3, 135.4, 133.7, 129.0, 128.9, 127.5, 126.5, 126.1 (q, *J* = 277.4 Hz), 125.9, 41.7 (q, *J* = 2.8 Hz), 38.1 (q, *J* = 28.4 Hz) ppm.

^{19}F NMR (376 MHz, Chloroform-*d*): δ = -65.33 (t, *J* = 10.7 Hz) ppm.

HRMS (ESI/QTOF): *m/z*: $[M + Na]^+$ Calcd for $C_{14}H_{11}F_3NaOS^+$ 307.0375; Found 307.0382.

IR (ATR): 1686, 1252, 1174, 1050, 689 cm^{-1}.

$[\alpha]_D^{20}$ = +29.2 (c = 0.6, $CHCl_3$).

Chiral HPLC: (Chiralpak IB, 1 % *i*PrOH/hexane, 1.0 mL/min, 254 nm): tR (major) 3.40 min, tR (minor) 4.23 min, 96:4 *er*.

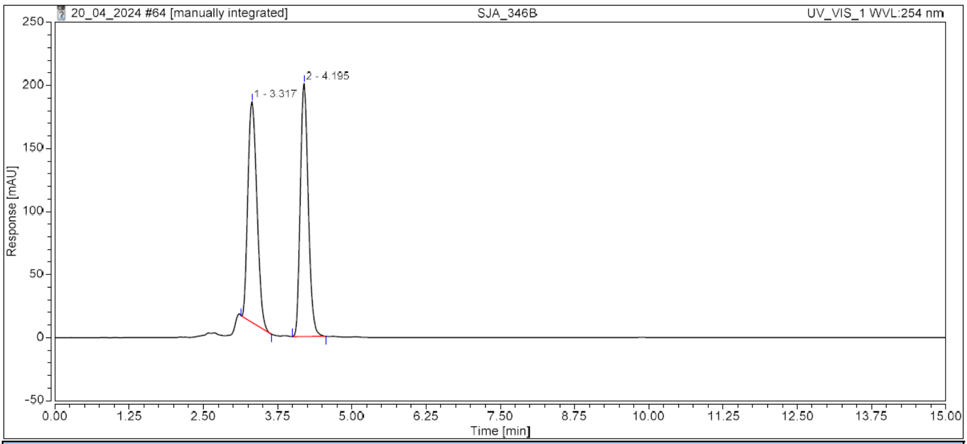

Integration Results							
No.	Peak Name	Retention Time min	Area mAU*min	Height mAU	Relative Area %	Relative Height %	Amount n.a.
1		3.317	30.667	175.097	49.84	46.60	n.a.
2		4.195	30.870	200.643	50.16	53.40	n.a.
**Total:**			**61.538**	**375.740**	**100.00**	**100.00**	

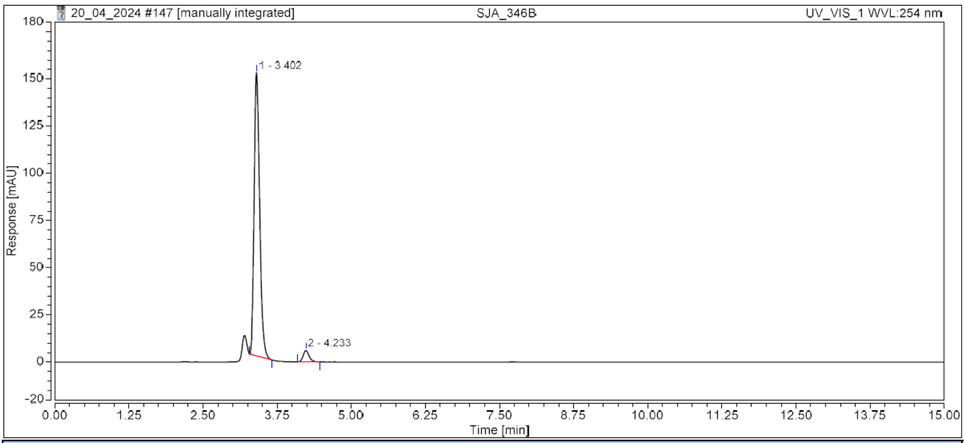

Integration Results							
No.	Peak Name	Retention Time min	Area mAU*min	Height mAU	Relative Area %	Relative Height %	Amount n.a.
1		3.402	16.079	150.083	95.93	96.14	n.a.
2		4.233	0.682	6.030	4.07	3.86	n.a.
**Total:**			**16.761**	**156.113**	**100.00**	**100.00**	

(*S*)-4,4,4-Trifluoro-1-phenyl-2-(pyridin-3-yl)butan-1-one (4g)

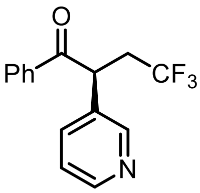

The title compound was synthesized according to the general procedure (**GP 7**) and was obtained after silica gel column chromatography (*n*-pentane : ethylacetate 20:1 to 9:1) as a colorless solid (71 % Yield, 20 mg). $\mathbf{R_f}$ = 0.28 (*n*-pentane : ethylacetate 9:1).

^{1}H NMR (400 MHz, Chloroform-*d*): δ = 8.64 (dd, *J* = 2.5, 0.8 Hz, 1H), 8.51 (dd, *J* = 4.8, 1.6 Hz, 1H), 7.98 – 7.91 (m, 2H), 7.62 (ddd, *J* = 8.0, 2.4, 1.6 Hz, 1H), 7.57 – 7.52 (m, 1H), 7.46 – 7.41 (m, 2H), 7.26

– 7.22 (m, 1H), 4.96 (t, *J* = 6.7 Hz, 1H), 3.27 (dqd, *J* = 15.1, 10.6, 7.3 Hz, 1H), 2.59 (dqd, *J* = 15.1, 10.5, 6.2 Hz, 1H) ppm.

^{13}C NMR (101 MHz, Chloroform-*d*): δ = 196.2, 149.9, 149.5, 135.28, 135.22, 134.0, 133.3, 129.0, 128.9, 126.2 (q, *J* = 277.2 Hz), 124.2, 44.5 (q, *J* = 2.5 Hz), 37.3 (q, *J* = 28.5 Hz) ppm.

^{19}F NMR (376 MHz, Chloroform-*d*): δ = -64.91 (t, *J* = 10.4 Hz) ppm.

HRMS (ESI/QTOF): *m/z*: $[M + H]^+$ Calcd for $C_{15}H_{13}F_3NO^+$ 280.0944; Found 280.0945

IR (ATR): 1685, 1479, 1254, 1141, 1110, 708 cm^{-1}.

$[\alpha]_D^{20}$ = +35.4 (c = 0.5, $CHCl_3$).

Chiral HPLC: (Chiralpak IC, 5 % *i*PrOH/hexane, 1.0 mL/min, 210 nm): tR (minor) 8.77 min, tR (major) 10.10 min, 94:6 *er*.

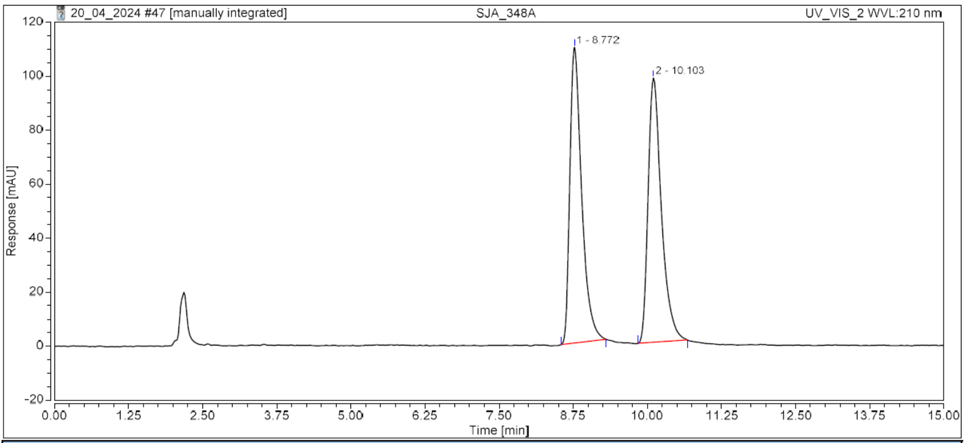

Integration Results

No.	Peak Name	Retention Time min	Area mAU*min	Height mAU	Relative Area %	Relative Height %	Amount n.a.
1		8.772	25.190	109.572	49.77	52.81	n.a.
2		10.103	25.423	97.894	50.23	47.19	n.a.
Total:			50.614	207.466	100.00	100.00	

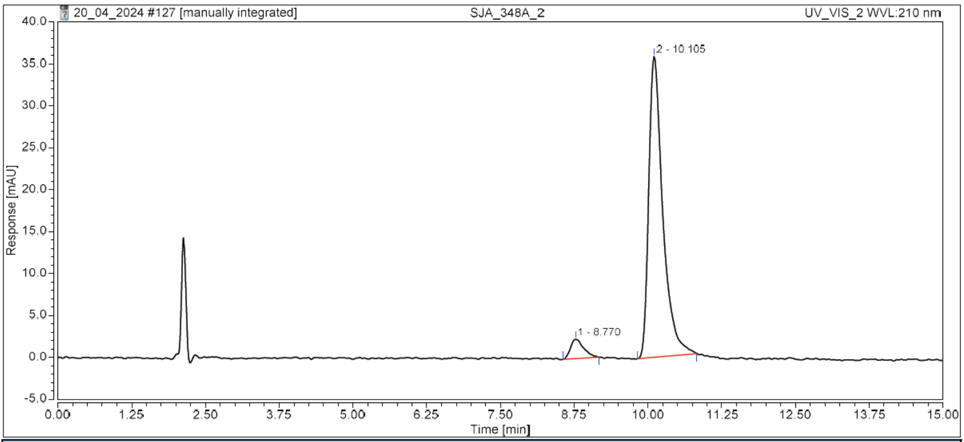

Integration Results

No.	Peak Name	Retention Time min	Area mAU*min	Height mAU	Relative Area %	Relative Height %	Amount n.a.
1		8.770	0.594	2.289	5.67	6.00	n.a.
2		10.105	9.876	35.883	94.33	94.00	n.a.
Total:			10.470	38.171	100.00	100.00	

(*R*)-4,4,4-Trifluoro-1-phenyl-2-(phenylthio)butan-1-one (4h)

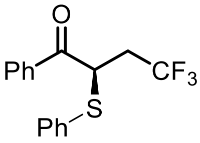

The title compound was synthesized according to the general procedure (**GP 7**) and was obtained after silica gel column chromatography (*n*-pentane : ethylacetate 30:1 to 20:1) as a colorless solid (57% Yield, 18 mg). **R**$_f$ = 0.34 (*n*-pentane : ethylacetate 30:1).

^{1}H NMR (400 MHz, Chloroform-*d*): δ = 7.98 – 7.93 (m, 2H), 7.64 – 7.57 (m, 1H), 7.52 – 7.45 (m, 2H), 7.41 – 7.28 (m, 5H), 4.71 (dd, *J* = 8.7, 4.3 Hz, 1H), 3.08 (dqd, *J* = 15.1, 10.5, 8.7 Hz, 1H), 2.63 (dqd, *J* = 14.9, 10.5, 4.3 Hz, 1H) ppm.

^{13}C NMR (101 MHz, Chloroform-*d*): δ = 192.6, 135.4, 135.2, 133.7, 129.9, 129.7, 129.4, 128.9, 128.8, 125.9 (d, *J* = 277.8 Hz), 43.9 (q, *J* = 2.4 Hz), 35.5 (q, *J* = 28.6 Hz) ppm.

^{19}F NMR (376 MHz, Chloroform-*d*): δ = -64.85 (t, *J* = 10.5 Hz) ppm.

HRMS (ESI/QTOF) m/z: $[M + H]^+$ Calcd for $C_{16}H_{14}F_3OS^+$ 311.0712; Found 311.0718.

IR (ATR): 1682, 12581168, 1137, 1065 cm^{-1}.

$[\alpha]_D^{20}$ = +13.4 (c = 0.5, $CHCl_3$).

Chiral HPLC: (Chiralpak IG, 0.2 % *i*PrOH/hexane, 1.0 mL/min, 254 nm): tR (minor) 19.76 min, tR (major) 22.93 min, 94:6 *er.*

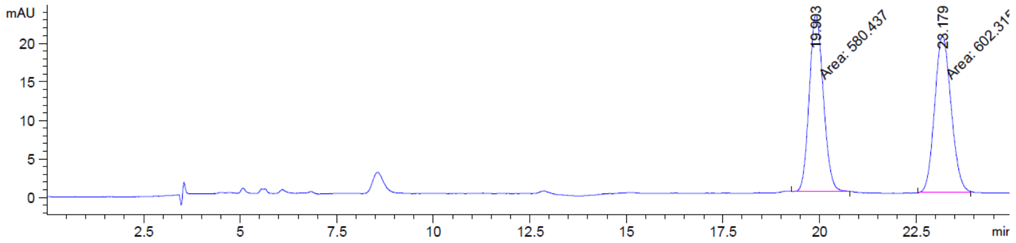

Peak #	RetTime [min]	Type	Width [min]	Area [mAU*s]	Height [mAU]	Area %
1	19.903	MM	0.4216	580.43713	22.94644	49.0751
2	23.179	MM	0.4942	602.31543	20.31406	50.9249

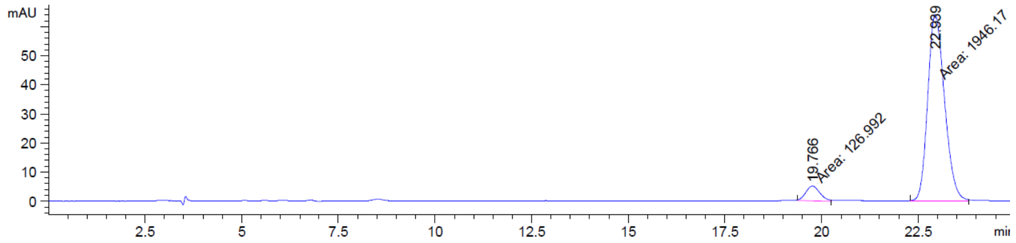

Peak #	RetTime [min]	Type	Width [min]	Area [mAU*s]	Height [mAU]	Area %
1	19.766	MM	0.4196	126.99155	5.04395	6.1255
2	22.939	MM	0.5062	1946.17358	64.08261	93.8745

(*R*)-2-(4,4,4-Trifluoro-1-oxo-1-phenylbutan-2-yl)isoindoline-1,3-dione (4i)

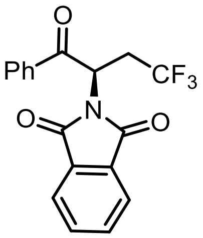

The title compound was synthesized according to the general procedure (**GP 7**) and was obtained after silica gel column chromatography (*n*-pentane : ethylacetate 20:1 to 9:1) as a colorless solid (64% Yield, 22 mg). $\mathbf{R_f}$ = 0.22 (*n*-pentane : ethylacetate 9:1).

^{1}**H NMR** (400 MHz, Chloroform-*d*): δ = 7.84 – 7.79 (m, 4H), 7.73 – 7.70 (m, 2H), 7.54 – 7.48 (m, 1H), 7.44 – 7.37 (m, 2H), 5.92 – 5.84 (m, 1H), 3.21 – 3.07 (m, 2H) ppm.

^{13}**C NMR** (101 MHz, Chloroform-*d*): δ = 193.5, 167.0, 134.6, 134.3, 133.7, 131.5, 129.0, 128.2, 126.3 (q, *J* = 276.6 Hz), 123.9, 49.3 (q, *J* = 2.6 Hz), 32.1 (q, *J* = 29.1 Hz) ppm.

^{19}**F NMR** (376 MHz, Chloroform-*d*): δ = -64.96 (t, *J* = 10.7 Hz) ppm.

HRMS (ESI/QTOF) m/z: $[M + H]^+$ Calcd for $C_{18}H_{13}F_3NO_3^+$ 348.0842; Found 348.0849.

IR (ATR): 1777, 1720, 1381, 1299, 1143, 767 cm^{-1}.

$[\alpha]_D^{20}$ = +15.4 (c = 0.5, $CHCl_3$).

Chiral HPLC: (Chiralpak IC, 1 % *i*PrOH/hexane, 1.0 mL/min, 210 nm): tR (major) 13.02 min, tR (minor) 15.08 min, 93:7 *er*.

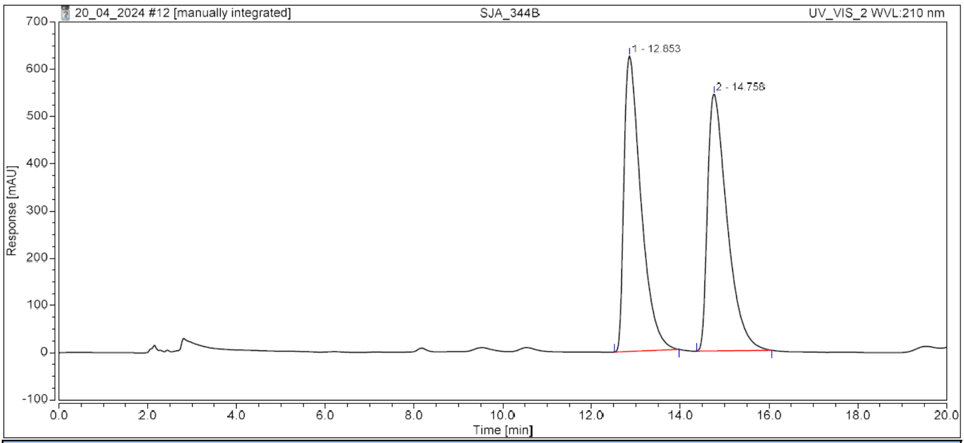

Integration Results

No.	Peak Name	Retention Time min	Area mAU*min	Height mAU	Relative Area %	Relative Height %	Amount n.a.
1		12.853	277.678	625.808	50.01	53.50	n.a.
2		14.758	277.544	544.029	49.99	46.50	n.a.
**Total:**			**555.222**	**1169.837**	**100.00**	**100.00**	

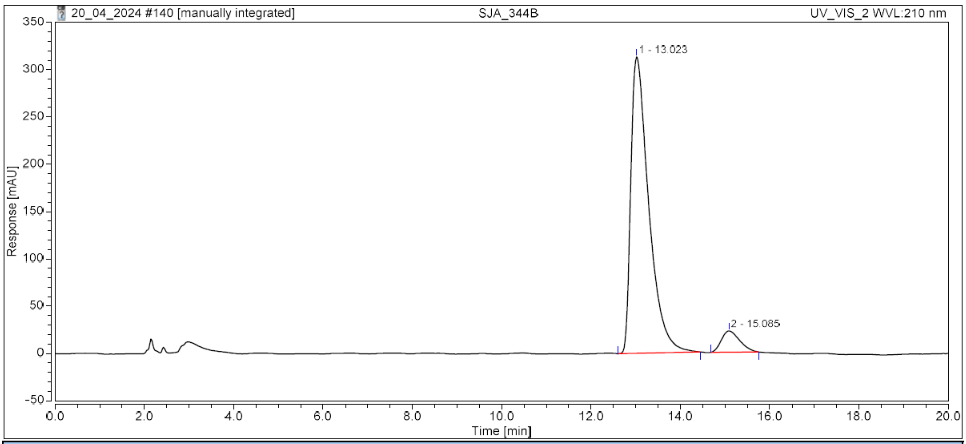

Integration Results

No.	Peak Name	Retention Time min	Area mAU*min	Height mAU	Relative Area %	Relative Height %	Amount n.a.
1		13.023	145.350	313.455	93.13	93.28	n.a.
2		15.085	10.719	22.575	6.87	6.72	n.a.
**Total:**			**156.069**	**336.029**	**100.00**	**100.00**	

(*R*)-4,4-Dimethyl-1-phenyl-2-(2,2,2-trifluoroethyl)pentan-1-one (4j)

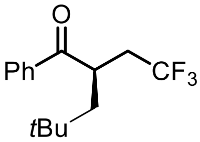

The title compound was synthesized according to the general procedure (**GP 7**) and was obtained after silica gel column chromatography (*n*-pentane : ethylacetate 80:1) as a colorless solid (69% Yield, 19 mg). **R$_f$** = 0.36 (*n*-pentane : ethylacetate 80:1).

^{1}H NMR (400 MHz, Chloroform-*d*): δ = 8.01 – 7.97 (m, 2H), 7.63 – 7.57 (m, 1H), 7.53 – 7.47 (m, 2H), 3.87 – 3.79 (m, 1H), 2.71 (dqd, *J* = 15.0, 10.8, 8.0 Hz, 1H), 2.27 (dqd, *J* = 15.5, 10.9, 4.8 Hz, 1H), 1.94 (dd, *J* = 14.2, 7.2 Hz, 1H), 1.43 (dd, *J* = 14.2, 4.5 Hz, 1H), 0.88 (s, 9H) ppm.

^{13}C NMR (101 MHz, Chloroform-*d*): δ = 201.9, 136.2, 133.5, 129.0, 128.5, 126.5 (q, *J* = 277.3 Hz), 46.1, 37.7 (q, *J* = 28.1 Hz), 36.0 (q, *J* = 2.1 Hz), 31.3, 29.9 ppm.

^{19}F NMR (376 MHz, Chloroform-*d*): δ = -65.13 (t, *J* = 10.5 Hz) ppm.

HRMS (ESI/QTOF): *m/z*: $[M + Na]^+$ Calcd for $C_{15}H_{19}F_3NaO^+$ 295.1280; Found 295.1276.

IR (ATR): 1686, 1476, 1303, 1141, 1105, 713 cm^{-1}.

$[\alpha]_D^{20}$ = +21.2 (c = 0.5, $CHCl_3$).

Chiral HPLC: (Chiralpak IC, 0.1 % *i*PrOH/hexane, 1.0 mL/min, 210 nm): tR (major) 5.20 min, tR (minor) 6.15 min, 95:5 *er*.

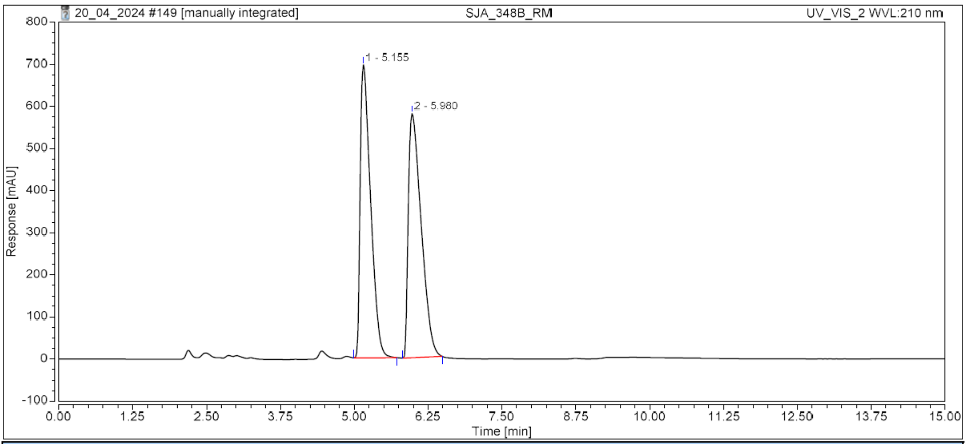

Integration Results

No.	Peak Name	Retention Time min	Area mAU*min	Height mAU	Relative Area %	Relative Height %	Amount n.a.
1		5.155	142.225	695.972	49.36	54.53	n.a.
2		5.980	145.900	580.363	50.64	45.47	n.a.
**Total:**			**288.125**	**1276.335**	**100.00**	**100.00**	

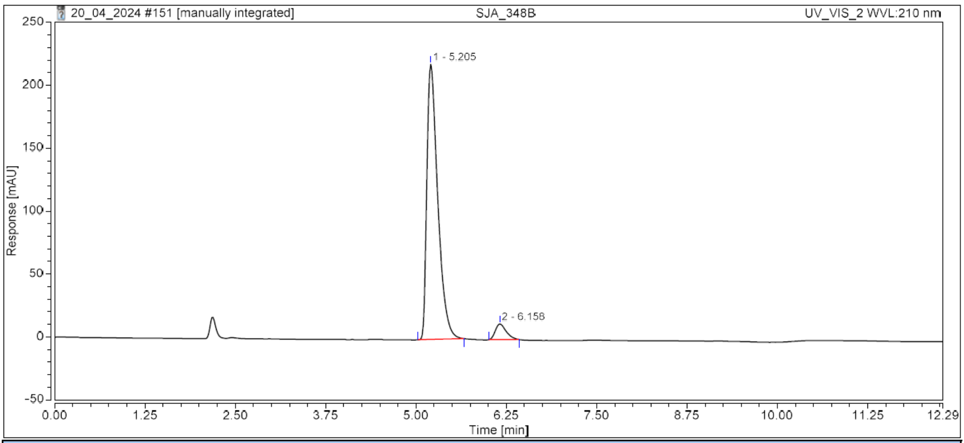

Integration Results							
No.	Peak Name	Retention Time min	Area mAU*min	Height mAU	Relative Area %	Relative Height %	Amount n.a.
1		5.205	38.045	218.640	94.60	94.61	n.a.
2		6.158	2.172	12.461	5.40	5.39	n.a.
Total:			40.216	231.101	100.00	100.00	

(*S*)-2-Cyclohexyl-4,4,4-trifluoro-1-phenylbutan-1-one (4k)

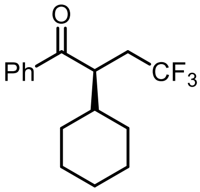

The title compound was synthesized according to the general procedure (**GP 7**) and was obtained after silica gel column chromatography (*n*-pentane : ethylacetate 80:1) as a colorless solid (46% Yield, 13 mg). $\mathbf{R_f}$ = 0.38 (*n*-pentane : ethylacetate 60:1).

^{1}H NMR (400 MHz, Chloroform-*d*): δ = 7.99 – 7.92 (m, 2H), 7.63 – 7.56 (m, 1H), 7.53 – 7.44 (m, 2H), 3.62 (ddd, *J* = 10.6, 5.1, 1.9 Hz, 1H), 3.02 – 2.84 (m, 1H), 2.34 – 2.20 (m, 1H), 1.75 – 1.59 (m, 6H), 1.28 – 1.03 (m, 4H), 0.97 – 0.85 (m, 1H) ppm.

^{13}C NMR (101 MHz, Chloroform-*d*): δ = 201.0, 137.1, 133.2, 128.7, 128.2, 127.0 (q, *J* = 276.8 Hz), 45.0 (q, *J* = 2.2 Hz), 40.7, 32.4 (q, *J* = 28.3 Hz), 31.2, 29.2, 26.3, 26.2, 26.0 ppm.

^{19}F NMR (376 MHz, Chloroform-*d*): δ = -65.55 (t, *J* = 11.1 Hz) ppm.

HRMS (ESI/QTOF): m/z: $[M + Na]^+$ Calcd for $C_{16}H_{19}F_3NaO^+$ 307.1280; Found 307.1273.

IR (ATR): 2929, 1683, 1598, 1257, 1151, 712 cm^{-1}.

$[\alpha]_D^{20}$ = +27.6 (c = 0.5, $CHCl_3$).

Chiral HPLC: (Chiralpak IC, 0.2 % *i*PrOH/hexane, 1.0 mL/min, 254 nm): tR (major) 6.78 min, tR (minor) 7.26 min, 95.3:4.7 *er*.

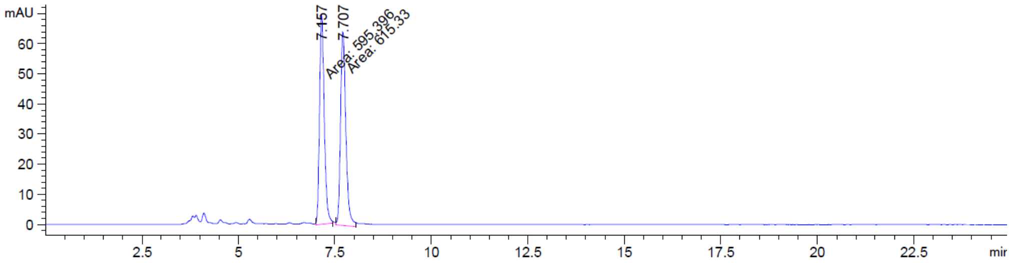

Peak #	RetTime [min]	Type	Width [min]	Area [mAU*s]	Height [mAU]	Area %
1	7.157	MM	0.1428	595.39636	69.51085	49.1768
2	7.707	MM	0.1591	615.33026	64.47378	50.8232

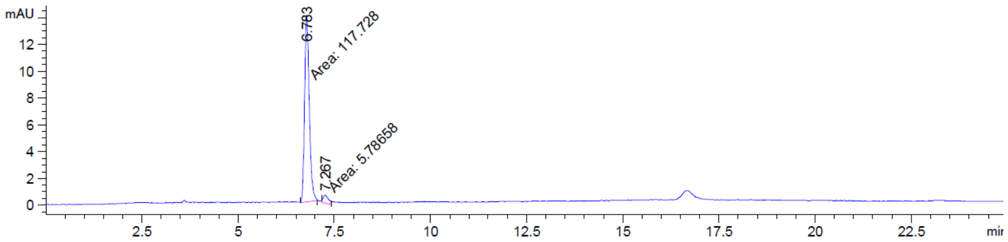

Peak #	RetTime [min]	Type	Width [min]	Area [mAU*s]	Height [mAU]	Area %
1	6.783	MM	0.1408	117.72830	13.93753	95.3151
2	7.267	MM	0.1590	5.78658	6.06660e-1	4.6849

(*S*)-4,4,5,5,6,6,6-Heptafluoro-1,2-diphenylhexan-1-one (4l)

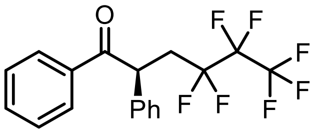

The title compound was synthesized according to the general procedure (**GP 7**) and was obtained after silica gel column chromatography (*n*-pentane : ethylacetate 80:1) as a colorless solid (69% Yield, 26 mg). $\mathbf{R_f}$ = 0.38 (*n*-pentane : ethylacetate 80:1).

^{1}H NMR (400 MHz, Chloroform-*d*): δ = 8.02 – 7.94 (m, 2H), 7.55 – 7.48 (m, 1H), 7.45 – 7.38 (m, 2H), 7.35 – 7.29 (m, 4H), 7.28 – 7.21 (m, 1H), 5.04 (dd, *J* = 8.4, 4.2 Hz, 1H), 3.54 – 3.35 (m, 1H), 2.54 – 2.34 (m, 1H) ppm.

^{13}C NMR (101 MHz, Chloroform-*d*): δ = 196.8, 137.8, 135.7, 133.5, 129.5, 128.9, 128.8, 128.1, 128.0, 123.3 – 105.0 (m), 45.6 (t, *J* = 2.2 Hz), 34.2 (t, *J* = 20.8 Hz) ppm.

^{19}F NMR (376 MHz, Chloroform-*d*): δ = -80.93 (t, *J* = 9.6 Hz), -112.73 – -115.67 (m), -128.24 (dd, *J* = 15.1, 4.9 Hz) ppm.

HRMS (ESI/QTOF): *m/z*: $[M + H]^+$ Calcd for $C_{18}H_{14}F_7O^+$ 379.0927; Found 379.0921.

IR (ATR): 1687, 1449, 1225, 1115, 1075, 698 cm^{-1}.

$[\alpha]_D^{20}$ = 121.2 (c = 1.0, $CHCl_3$).

Chiral HPLC: (Chiralpak IB, 0.1 % *i*PrOH/hexane, 1.0 mL/min, 254 nm): tR (major) 9.73 min, tR (minor) 12.63 min, 96:4 *er*.

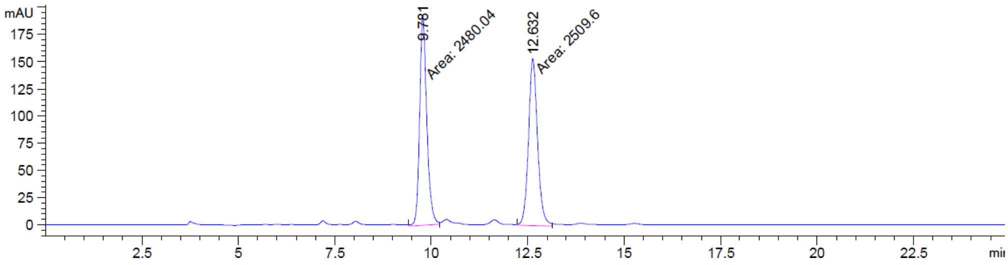

Peak #	RetTime [min]	Type	Width [min]	Area [mAU*s]	Height [mAU]	Area %
1	9.781	MM	0.2144	2480.04297	192.78847	49.7038
2	12.632	MM	0.2722	2509.59814	153.64198	50.2962

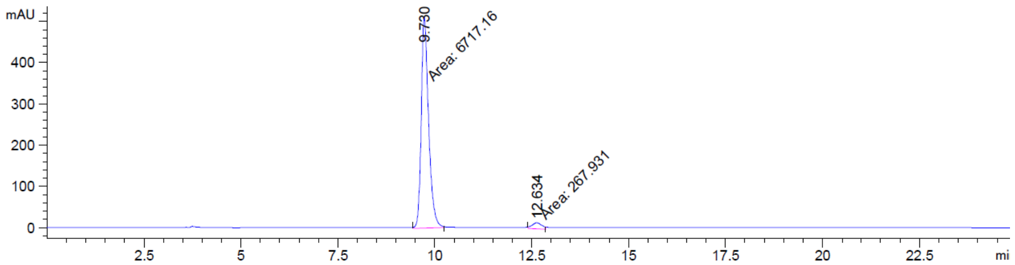

Peak #	RetTime [min]	Type	Width [min]	Area [mAU*s]	Height [mAU]	Area %
1	9.730	MM	0.2185	6717.15576	512.27185	96.1642
2	12.634	MM	0.2991	267.93091	14.93021	3.8358

(*S*)-4,4,5,5,6,6,7,7,7-Nonafluoro-1,2-diphenylheptan-1-one (4m)

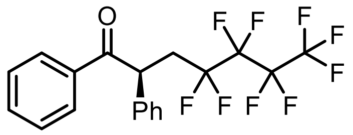

The title compound was synthesized according to the general procedure (**GP 7**) and was obtained after silica gel column chromatography (*n*-pentane : ethylacetate 80:1) as a colorless solid (62% Yield, 26.5 mg). **R$_f$** = 0.40 (*n*-pentane : ethylacetate 80:1).

^{1}H NMR (400 MHz, Chloroform-*d*): δ = 8.00 – 7.96 (m, 2H), 7.56 – 7.48 (m, 1H), 7.45 – 7.39 (m, 2H), 7.35 – 7.31 (m, 3H), 7.29 – 7.19 (m, 1H), 5.05 (dd, *J* = 8.4, 4.2 Hz, 1H), 3.56 – 3.37 (m, 1H), 2.55 – 2.36 (m, 1H) ppm.

^{13}C NMR (101 MHz, Chloroform-*d*): δ = 196.8, 137.8, 135.7, 133.5, 129.5, 128.9, 128.8, 128.2, 128.0, 122.95 – 105.33 (m), 45.7, 34.4 (t, *J* = 20.8 Hz) ppm.

^{19}F NMR (376 MHz, Chloroform-*d*): δ = -81.47 – -81.58 (m), -112.09 – -114.91 (m), -124.77 – -125.00 (m), -126.30 – -126.50 (m) ppm.

HRMS (ESI/QTOF) m/z: [M + H]$^+$ Calcd for $C_{19}H_{14}F_9O^+$ 429.0895; Found 429.0900.

IR (ATR): 1687, 1218, 1133, 1103, 715 cm^{-1}.

$[\alpha]_D^{20}$ = +79.8 (c = 0.5, $CHCl_3$).

Chiral HPLC: (Chiralpak IB, 0.1 % *i*PrOH/hexane, 1.0 mL/min, 254 nm): tR (major) 6.37 min, tR (minor) 7.39 min, 95.5:4.5 *er*.

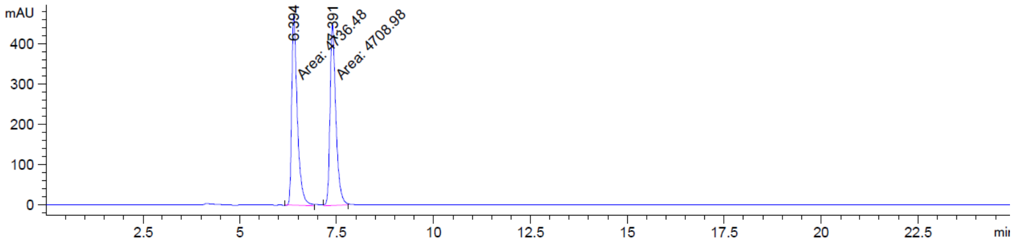

Peak #	RetTime [min]	Type	Width [min]	Area [mAU*s]	Height [mAU]	Area %
1	6.394	MM	0.1667	4736.48438	473.43192	50.1456
2	7.391	MM	0.1751	4708.98438	448.31842	49.8544

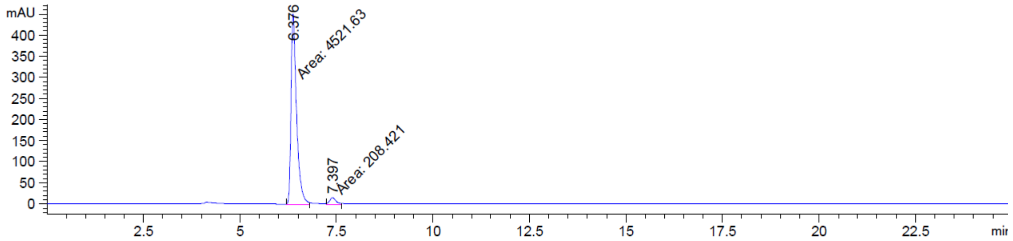

Peak #	RetTime [min]	Type	Width [min]	Area [mAU*s]	Height [mAU]	Area %
1	6.376	MM	0.1664	4521.63379	453.00137	95.5937
2	7.397	MM	0.2028	208.42079	17.13265	4.4063

Isopropyl (*S*)-2-methyl-2-(4-(4-(4,4,4-trifluoro-1-oxo-1-phenylbutan-2-yl)benzoyl)phenoxy)propanoate (6a)

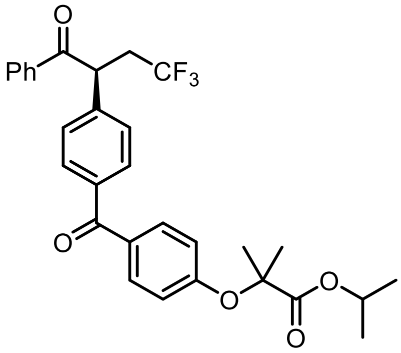

The title compound was synthesized according to the general procedure (**GP 7**) and was obtained after silica gel column chromatography (*n*-pentane : ethylacetate 9:1) as a colorless oil (60% Yield, 31.5 mg). $\mathbf{R_f}$ = 0.24 (*n*-pentane : ethylacetate 9:1).

^{1}H NMR (400 MHz, Chloroform-*d*): δ = 8.00 – 7.93 (m, 2H), 7.73 – 7.67 (m, 4H), 7.57 – 7.52 (m, 1H), 7.48 – 7.37 (m, 4H), 6.87 – 6.81 (m, 2H), 5.12 – 5.02(m, 1H), 5.00 (dd, *J* = 7.4, 5.7 Hz, 1H), 3.32 (dqd, *J* = 15.1, 10.7, 7.4 Hz, 1H), 2.60 (dqd, *J* = 15.0, 10.6, 5.7 Hz, 1H), 1.65 (s, 6H), 1.19 (d, *J* = 6.3 Hz, 6H) ppm.

^{13}C NMR (101 MHz, Chloroform-*d*): δ = 196.3, 194.8, 173.2, 159.8, 141.4, 137.8, 135.5, 133.8, 132.1, 130.8, 130.3, 128.99, 128.96, 128.1, 126.4 (q, *J* = 277.2 Hz), 117.3, 79.5, 69.4, 47.2 (q, *J* = 2.5 Hz), 37.4 (q, *J* = 28.4 Hz), 25.5, 25.4, 21.6 ppm.

^{19}F NMR (376 MHz, Chloroform-*d*): δ = -65.00 (t, *J* = 10.7 Hz) ppm.

HRMS (ESI/QTOF): *m/z*: $[M + Na]^+$ Calcd for $C_{30}H_{29}F_3NaO_5^+$ 549.1859; Found 549.1870.

IR (ATR): 1730, 1686, 1654, 1599, 1283, 1257, 1144, 929 cm^{-1}.

$[\boldsymbol{\alpha}]_{\boldsymbol{D}}^{\mathbf{20}}$ = +79.8 (c = 0.5, $CHCl_3$).

Chiral HPLC: (Chiralpak IB, 1 % *i*PrOH/hexane, 1.0 mL/min, 254 nm): tR (major) 11.11 min, tR (minor) 15.61 min, 91:9 *er*.

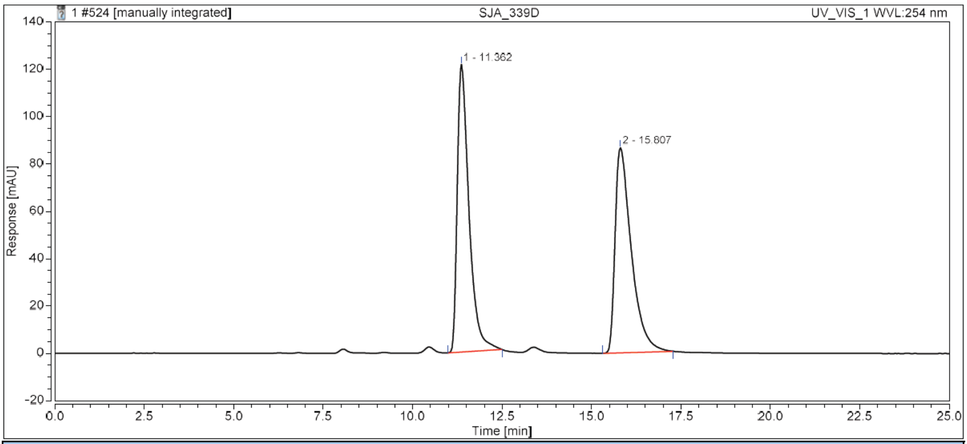

Integration Results							
No.	Peak Name	Retention Time min	Area mAU*min	Height mAU	Relative Area %	Relative Height %	Amount n.a.
1		11.362	46.182	121.539	50.37	58.38	n.a.
2		15.807	45.495	86.634	49.63	41.62	n.a.
**Total:**			**91.677**	**208.173**	**100.00**	**100.00**	

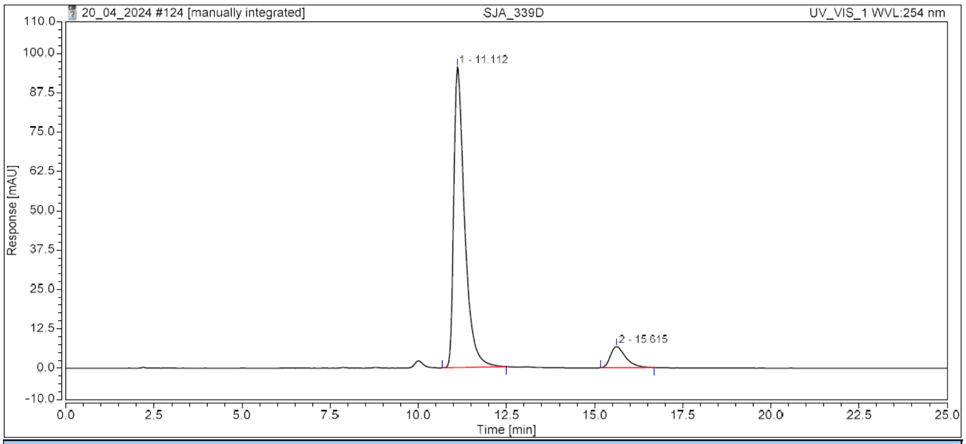

Integration Results							
No.	Peak Name	Retention Time min	Area mAU*min	Height mAU	Relative Area %	Relative Height %	Amount n.a.
1		11.112	34.210	95.584	91.02	93.40	n.a.
2		15.615	3.376	6.750	8.98	6.60	n.a.
**Total:**			**37.586**	**102.334**	**100.00**	**100.00**	

(8*R*,9*S*,13*S*,14*S*)-13-methyl-3-((*S*)-4,4,4-trifluoro-1-oxo-1-phenylbutan-2-yl)-6,7,8,9,11,12,13,14,15,16-decahydro-17*H*-cyclopenta[a]phenanthren-17-one (6b)

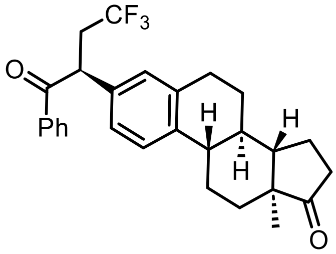

The title compound was synthesized according to the general procedure (**GP 7**) and was obtained after silica gel column chromatography (*n*-pentane : ethylacetate 9:1 to 4:1) as a pale yellow oil (56% Yield, 25.5 mg). $\mathbf{R_f}$ = 0.32 (*n*-pentane : ethylacetate 4:1).

^{1}**H NMR** (400 MHz, Chloroform-*d*): δ = 8.01 – 7.96 (m, 2H), 7.55 – 7.48 (m, 1H), 7.46 – 7.38 (m, 2H), 7.22 (dd, *J* = 8.4, 2.7 Hz, 1H), 7.12 – 7.05 (m, 1H), 7.00 (dd, *J* = 5.0, 2.0 Hz, 1H), 4.85 (dd, *J* = 8.2, 4.9 Hz, 1H), 3.43 – 3.19 (m, 1H), 2.90 – 2.81 (m, 2H), 2.58 – 2.43 (m, 2H), 2.39 – 2.32 (m, 1H), 2.28 – 2.19 (m, 1H), 2.18 – 1.89 (m, 4H), 1.67 – 1.33 (m, 6H), 0.88 (d, *J* = 2.4 Hz, 3H).

^{13}C NMR (101 MHz, Chloroform-*d*): δ = 220.7, 196.8 (d, *J* = 2.6 Hz), 139.4, 137.6 (d, *J* = 1.9 Hz), 135.8, 134.7, 133.3, 128.8, 128.6, 128.2 (d, *J* = 3.1 Hz), 126.4 (q, *J* = 277.3 Hz), 126.3 (d, *J* = 1.5 Hz), 125.5 (d, *J* = 2.3 Hz), 50.5, 47.9, 46.5 (p, *J* = 2.5 Hz), 44.2, 37.9, 37.4 (qd, *J* = 28.1, 3.7 Hz), 35.8, 31.5, 29.3 (d, *J* = 4.2 Hz), 26.3 (d, *J* = 1.4 Hz), 25.5, 21.5, 13.8 ppm.

^{19}F NMR (376 MHz, Chloroform-*d*): δ = -65.26 (t, *J* = 10.5 Hz) ppm.

HRMS (ESI/QTOF): *m/z*: $[M + Na]^+$ Calcd for $C_{28}H_{29}F_3NaO_2^+$ 477.2012; Found 477.2014.

IR (ATR): 1735, 1684, 1260, 1105, 1083, 713 cm^{-1}.

$[\alpha]_D^{20}$ = +134.2 (c = 0.5, $CHCl_3$).

Chiral HPLC: (Chiralpak IB, 3 % *i*PrOH/hexane, 1.0 mL/min, 210 nm): tR (major) 7.11 min, tR (minor) 8.57 min, 96:4 *dr*.

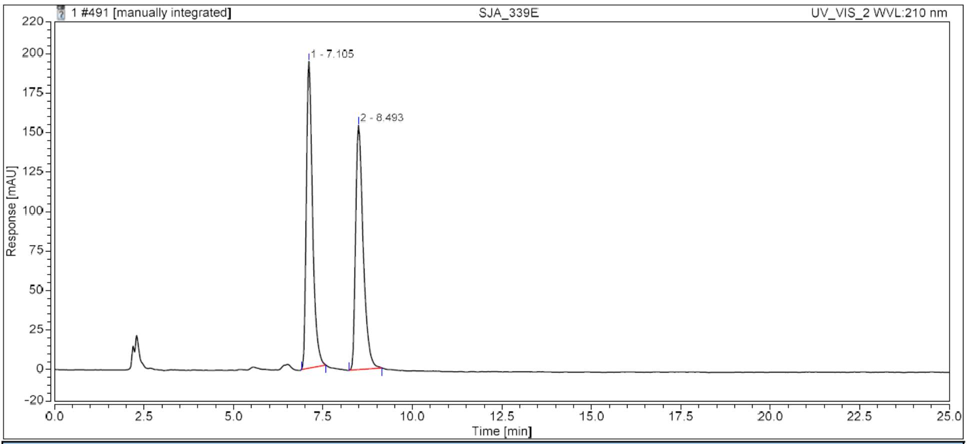

Integration Results

No.	Peak Name	Retention Time<br>min	Area<br>mAU*min	Height<br>mAU	Relative Area<br>%	Relative Height<br>%	Amount<br>n.a.
1		7.105	38.719	194.195	50.55	55.65	n.a.
2		8.493	37.875	154.777	49.45	44.35	n.a.
**Total:**			**76.594**	**348.972**	**100.00**	**100.00**	

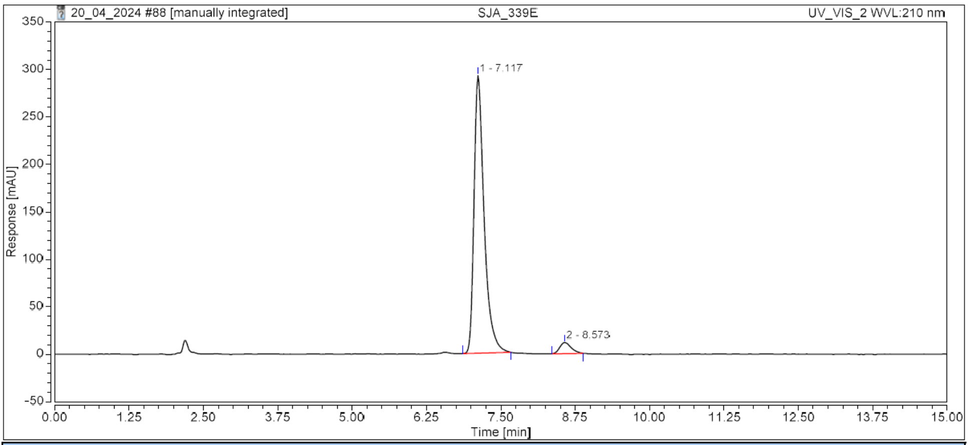

Integration Results

No.	Peak Name	Retention Time<br>min	Area<br>mAU*min	Height<br>mAU	Relative Area<br>%	Relative Height<br>%	Amount<br>n.a.
1		7.117	57.786	292.365	95.71	96.18	n.a.
2		8.573	2.591	11.623	4.29	3.82	n.a.
**Total:**			**60.377**	**303.989**	**100.00**	**100.00**	

(*S*)-2-((*R*)-2,8-Dimethyl-2-((4*R*,8*R*)-4,8,12-trimethyltridecyl)chroman-6-yl)-4,4,4-trifluoro-1-phenylbutan-1-one (6c)

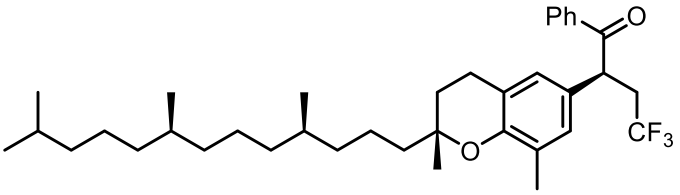

The title compound was synthesized according to the general procedure (**GP 7**) and was obtained after silica gel column chromatography (*n*-pentane : ethylacetate 20:1) as a colorless oil (69% Yield, 40.5 mg). $\mathbf{R_f}$ = 0.28 (*n*-pentane : ethylacetate 30:1).

^{1}H NMR (400 MHz, Chloroform-*d*): δ = 8.01 – 7.96 (m, 2H), 7.54 – 7.48 (m, 1H), 7.45 – 7.39 (m, 2H), 6.89 – 6.75 (m, 2H), 4.76 (dd, *J* = 8.6, 4.4 Hz, 1H), 3.31 (dqd, *J* = 14.9, 10.8, 8.5 Hz, 1H), 2.75 – 2.61 (m, 2H), 2.54 – 2.35 (m, 1H), 2.10 (s, 3H), 1.81 – 1.64 (m, 2H), 1.58 – 1.48 (m, 3H), 1.46 – 0.98 (m, 21H), 0.90 – 0.81 (m, 12H) ppm.

^{13}C NMR (101 MHz, Chloroform-*d*): δ = 197.1, 151.7, 136.0, 133.1, 128.8, 128.6, 127.8 (d, *J* = 4.1 Hz), 127.2, 127.1, 126.4 (q, *J* = 277.4 Hz), 126.2 (d, *J* = 3.8 Hz), 121.2, 76.2, 46.2 (q, *J* = 2.6 Hz), 40.3 (d, *J* = 2.1 Hz), 39.3, 37.66 (q, *J* = 27.8 Hz), 37.46, 37.42, 37.3, 32.8, 32.6, 30.9 (d, *J* = 1.4 Hz), 28.0, 24.8, 24.4, 24.2 (d, *J* = 1.4 Hz), 22.7, 22.6, 22.2, 20.9, 19.7, 19.6, 16.1 ppm.

^{19}F NMR (376 MHz, Chloroform-*d*): δ = -65.37 (t, *J* = 10.9 Hz) ppm.

HRMS (ESI/QTOF): *m/z*: $[M + Na]^+$ Calcd for $C_{37}H_{53}F_3NaO_2^+$ 609.3890; Found 609.3885.

IR (ATR): 1962, 1685, 1448. 1259, 1136, 1106, 712 cm^{-1}.

$[\alpha]_D^{20}$ = +17.2 (c = 0.5, $CHCl_3$).

Chiral HPLC: (Chiralpak IB, 0.5 % *i*PrOH/hexane, 1.0 mL/min, 230 nm): tR (major) 4.12 min, tR (minor) 4.68 min, 97:3 *dr*.

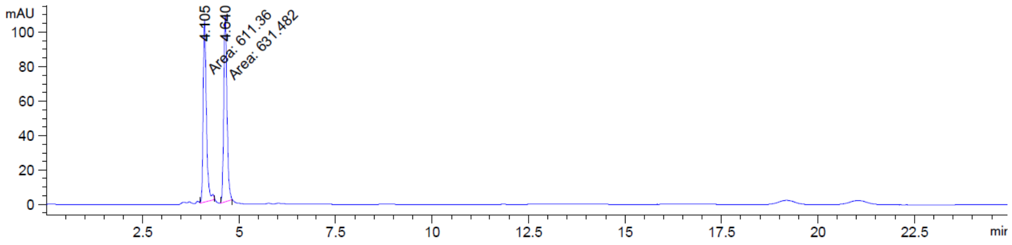

Peak #	RetTime [min]	Type	Width [min]	Area [mAU*s]	Height [mAU]	Area %
1	4.105	MM	0.0957	611.35956	106.49433	49.1904
2	4.640	MM	0.0967	631.48248	108.83195	50.8096

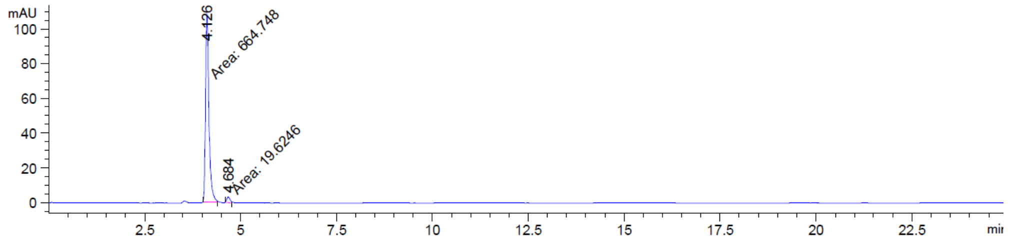

Peak #	RetTime [min]	Type	Width [min]	Area [mAU*s]	Height [mAU]	Area %
1	4.126	MM	0.1024	664.74780	108.23608	97.1325
2	4.684	MM	0.0973	19.62462	3.36138	2.8675

Scale up experiment

In a nitrogen-filled glove box, an oven-dried 50 mL screw cap reaction tube was charged with chiral thiazolium salt **NHC7** (90 mg, 0.1 mmol, 10 mol%). Then, 10 mL of dry and degassed methyl *tert*-butyl ether (TBME) was added. Subsequently, aldehyde **1d** (246 mg, 1.5 mmol, 1.5 eq.), styrene **2a** (104 mg, 1 mmol, 1.0 eq.), Togni reagent I **F1** (660 mg, 2 mmol, 2 eq.), and Cs_2CO_3 (81.5 mg, 0.25 mmol, 25 mol%) were added to the reaction tube, which was then screwed tightly. The reaction tube was removed from the glove box and stirred at 40 °C for 36 hours. The reaction mixture was then filtered through a short pad of silica gel (2 cm), and the solvent was evaporated under reduced pressure. The crude product mixture was purified by silica gel column chromatography using *n*-pentane : ethylacetate (30:1) mixture as eluent to obtain enantioenriched ketone **4d** as colourless solid in 61% yield (205 mg, 92:8 er).

Control experiments

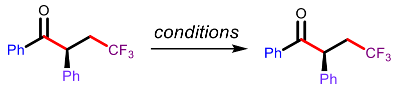

3a, 96:4 er
or
(±)-**3a**

3a

Results

	conditions	er 3a
with 96:4 er **3a**	25 mol% Cs_2CO_3 in TBME (0.1 M), at 40 °C, 16 h	**95.5:4.5**
	10 mol% **NHC7** + 25 mol% Cs_2CO_3 in TBME (0.1 M), at 40 °C, 16 h	**96:4**
	10 mol% **NHC7** in TBME (0.1 M), at 40 40 °C, 16 h	**96:4**
(±)-**3a**	10 mol% **NHC7** + 25 mol% Cs_2CO_3 in TBME (0.1 M), at 40 °C, 16 h	**50:50**

Inside a nitrogen-filled glove box, a 2 mL screw-cap vial was charged with either 92% enantiopure (S)-3a or (±)-3a (6.95 mg, 0.025 mmol) and treated under the conditions mentioned in the table above, with the results also noted in the same table.

X-ray Crystallography data of 3d

Methyl (*S*)-4-(4,4,4-trifluoro-2-phenylbutanoyl)benzoate (3d)

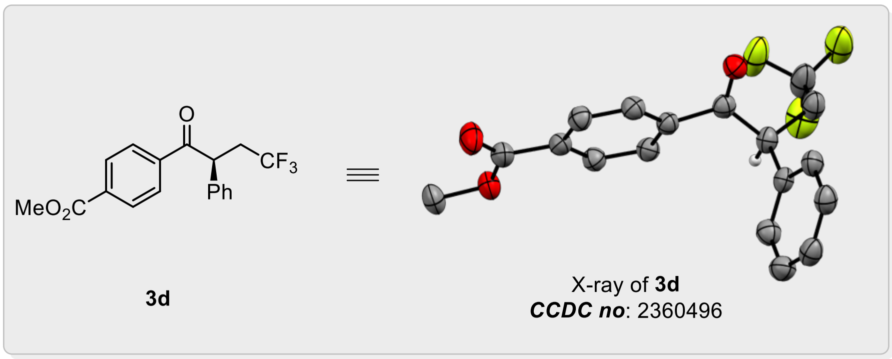

Experimental. Single colorless needle-shaped crystals of **3d** were used as supplied. A suitable crystal with dimensions 0.60 × 0.04 × 0.04 mm^3 was selected and mounted on a XtaLAB Synergy R, DW system, HyPix-Arc 150 diffractometer. The crystal was kept at a steady T = 139.99(10) K during data collection. The structure was solved with the olex2.solve 1.5 (Bourhis et al., 2015) solution program using iterative methods and by using Olex2 1.5 (Dolomanov et al., 2009)[11,12] as the graphical interface. The model was refined with ShelXL 2019/3 (Sheldrick, 2015)[13] using full matrix least squares minimisation on $\boldsymbol{F^2}$.

Crystal Data. $C_{18}H_{15}F_3O_3$, M_r = 336.30, orthorhombic, $P2_12_12_1$ (No. 19), a = 5.67450(11) Å, b = 15.6592(3) Å, c = 17.9793(3) Å, $\alpha = \beta = \gamma = 90^\circ$, V = 1597.61(5) Å^3, T = 139.99(10) K, Z = 4, Z' = 1, μ(Cu K_α) = 1.010, 17635 reflections measured, 3239 unique (R_{int} = 0.0377) which were used in all calculations. The final wR_2 was 0.0834 (all data) and R_1 was 0.0329 (I≥2 σ(I)).

**Compound**	**3d**
Formula	$C_{18}H_{15}F_3O_3$
$D_{calc.}$/ g cm^{-3}	1.398
μ/mm^{-1}	1.010
Formula Weight	336.30
Colour	colourless
Shape	needle
Size/mm^3	0.60×0.04×0.04
T/K	139.99(10)
Crystal System	orthorhombic
Flack Parameter	0.05(7)
Hooft Parameter	0.05(7)
Space Group	$P2_12_12_1$
a/Å	5.67450(11)
b/Å	15.6592(3)
c/Å	17.9793(3)
α/°	90
β/°	90
γ/°	90
V/Å^3	1597.61(5)
Z	4
Z'	1
Wavelength/Å	1.54184
Radiation type	Cu K_α
Θ_{min}/°	3.743
Θ_{max}/°	75.800
Measured Refl's.	17635
Indep't Refl's	3239
Refl's I≥2 σ(I)	2859
$R_{\rm int}$	0.0377
Parameters	278
Restraints	0
Largest Peak	0.177
Deepest Hole	-0.181
GooF	1.047
wR_2 (all data)	0.0834
wR_2	0.0808
R_1 (all data)	0.0387
R_1	0.0329
CCDC number	**2360496**

Structure Quality Indicators

**Reflections:**	d min (CuKα) 2Θ=151.6° 0.80	I/σ(I) 38.2	Rint m=5.47 3.77%	Full 135.4° 98% to 151.6° 100
**Refinement:**	Shift 0.000	Max Peak 0.2	Min Peak -0.2	GooF 1.047
	Hooft .05(7)			

A colourless needle-shaped crystal with dimensions 0.60 × 0.04 × 0.04 mm^3 was mounted. Data were collected using a XtaLAB Synergy R, DW system, HyPix-Arc 150 diffractometer operating at T = 139.99(10) K.

Data were measured using ω scans with Cu K_α radiation. The diffraction pattern was indexed and the total number of runs and images was based on the strategy calculation from the program CrysAlisPro system (CCD 43.121a 64-bit (release 24-04-2024)). The maximum resolution that was achieved was Θ = 75.800° (0.80 Å).

The unit cell was refined using CrysAlisPro 1.171.43.124a (Rigaku OD, 2024) on 6810 reflections, 39% of the observed reflections.

Data reduction, scaling and absorption corrections were performed using CrysAlisPro 1.171.43.124a (Rigaku OD, 2024).[14] The final completeness is 100.00 % out to 75.800° in Θ. An analytical absorption correction was performed using CrysAlisPro 1.171.43.124a (Rigaku Oxford Diffraction, 2024). The analytical numeric absorption correction was done using a multifaceted crystal model based on expressions derived by R.C. Clark & J.S. Reid. (Clark, R. C. & Reid, J. S. (1995). Acta Cryst. A51, 887-897). The empirical absorption correction was done using spherical harmonics, implemented in SCALE3 ABSPACK scaling algorithm. The absorption coefficient μ of this crystal is 1.010 mm^{-1} at this wavelength (λ = 1.54184Å) and the minimum and maximum transmissions are 0.743 and 0.968.

The structure was solved and the space group $P2_12_12_1$ (# 19) determined by the olex2.solve 1.5 (Bourhis et al., 2015) structure solution program using iterative methods and refined by full matrix least squares minimisation on $\boldsymbol{F}^2$ using version 2019/3 of ShelXL (Sheldrick, 2015). All non-hydrogen atoms were refined anisotropically. Hydrogen atom positions were calculated geometrically and refined freely.

There is a single formula unit in the asymmetric unit, which is represented by the reported sum formula. In other words: Z is 4 and Z' is 1. The moiety formula is C18 H15 F3 O3.

The Flack parameter was refined to 0.05(7). Determination of absolute structure using Bayesian statistics on Bijvoet differences using the Olex2 results in 0.05(7). The chiral atoms in this structure are: C1(S). Note: The Flack parameter is used to determine chirality of the crystal studied, the value should be near 0, a value of 1 means that the stereochemistry is wrong and the model should be inverted. A value of 0.5 means that the crystal consists of a racemic mixture of the two enantiomers.

Ortep-represention of (*S*)-**3d** (thermal ellipsoids set at 50% probability). CCDC **2360496** contains the crystallographic data for (*S*)-**3d**. These data can be obtained free of charge from The Cambridge Crystallographic Data Centre via www.ccdc.cam.ac.uk/data_request/cif.

Datablock: sja339-1

Bond precision:	C-C = 0.0032 A		Wavelength=1.54184
Cell:	a=5.67450(11)	b=15.6592(3)	c=17.9793(3)
	alpha=90	beta=90	gamma=90
Temperature:	140 K		

	Calculated	Reported
Volume	1597.61(5)	1597.61(5)
Space group	P 21 21 21	P 21 21 21
Hall group	P 2ac 2ab	P 2ac 2ab
Moiety formula	C18 H15 F3 O3	C18 H15 F3 O3
Sum formula	C18 H15 F3 O3	C18 H15 F3 O3
Mr	336.30	336.30
Dx,g cm-3	1.398	1.398
Z	4	4
Mu (mm-1)	1.010	1.010
F000	696.0	696.0
F000'	698.69	
h,k,lmax	7,19,22	6,19,22
Nref	3323[1942]	3239
Tmin,Tmax	0.953,0.964	0.743,0.968
Tmin'	0.546	

Correction method= # Reported T Limits: Tmin=0.743 Tmax=0.968
AbsCorr = ANALYTICAL

Data completeness= 1.67/0.97 Theta(max)= 75.800

R(reflections)= 0.0329(2859) wR2(reflections)= 0.0834(3239)

S = 1.047 Npar= 278

Computational Details.

Due to the potential presence of numerous energetically low-lying conformations for each of the reactants, transition states, and products, we ran CREST[15] simulations on each species (employing constraints on the transition states when needed). From these simulations, a set of the ten most diverse structures lying within 15.0 kcal/mol of the global minima were extracted using marc, a conformer clustering algorithm.[16] The geometries of all of these species were then optimized in the gas phase using the PBE0[17,18] density functional with Grimme's -D3(BJ) dispersion correction[19,20] along [*i.e.*, PBE0-D3(BJ)] with the def2-SVP basis set[21] using Gaussian16[22] and those species having the lowest energies retained for further consideration. For the radical species, as well as for the transition states that involve a radical-radical reaction to form the new C-C bond, optimizations were performed using the unrestricted open-shell wavefunction, including the keyword "guess=mix" which destroys a-b and spatial symmetries and facilitates the optimization of open-shell singlet state structures (for the TS optimizations). Single point energies using the SMD solvation model[23] for diethylether (as a surrogate for tert-butyl methyl ether) were then determined for the selected species at the PBE0-D3(BJ)/def2-TZVP level on the optimized PBE0-D3(BJ)/def2-SVP geometries. Each species was characterized as either minima (zero imaginary frequencies) or transition states (one imaginary frequency) by examining vibrational frequencies on the optimized structures. Free energy corrections were determined at 313.15 K using Goodvibes.[24,25] Final free energies for each species/conformer were obtained by summing the PBE0-D3(BJ)/def2-TZVP electronic energies and the enthalpy/entropy corrections from the PBE0-D3(BJ)/def2-SVP frequency analysis. The lowest energy conformers from each of the respective pathways were used to determine effective DG values via Boltzmann weighting at 313.15K at which point a computed enantioselectivity ratio was established from the theoretical kinetic constants: $k_{S/R} = \exp\left(\frac{-\Delta G^{TS}_{eff,S/R}}{kT}\right)$ as $er_{S/R} = 100 \times \frac{k_{S/R}}{k_S + k_R}$.

Table S7. Relative free energies of the transition state structures. Values in kcal/mol.

**Structure**	**ΔΔG‡ (relative to lowest energy enantiomer)**
TS1_si-si (leading to the *S*-product)	4.63
TS2_re-re (leading to the *R*-product)	3.02
TS3_si-re (leading to the *R*-product)	0.54
TS4_re-si (leading to the *S*-product)	0.00

Table S8. Electronic energies, free energy corrections, single point electronic energies, and total free energies of key species. Values in hartree.

	**PBE0-D3(BJ)/def2-SVP Electronic Energy**	**PBE0-D3(BJ)/def2-SVP Free Energy Correction**	**PBE0-D3(BJ)/def2-TZVP// PBE0-D3(BJ)/def2-SVP Electronic Energy**	**Total Free Energy**
NHC7	-2772.857967	1.036756	-2775.544367	-2774.507611
Ketyl Radical7	-3117.298112	1.132407	-3120.308042	-3119.175635
TS1_si-si	-3763.406442	1.278091	-3767.176195	-3765.898104
TS2_re-re	-3763.401675	1.276506	-3767.177169	-3765.900663
TS3_si-re	-3763.406389	1.276185	-3767.180810	-3765.904625
TS4_re-si	-3763.408578	1.276914	-3767.182397	-3765.905483

NMR spectra

2,6-Bis(1-(3,5-dimethylphenyl)vinyl)-4-(trifluoromethyl)aniline (9c)

^{1}H NMR (400 MHz, Chloroform-*d*):

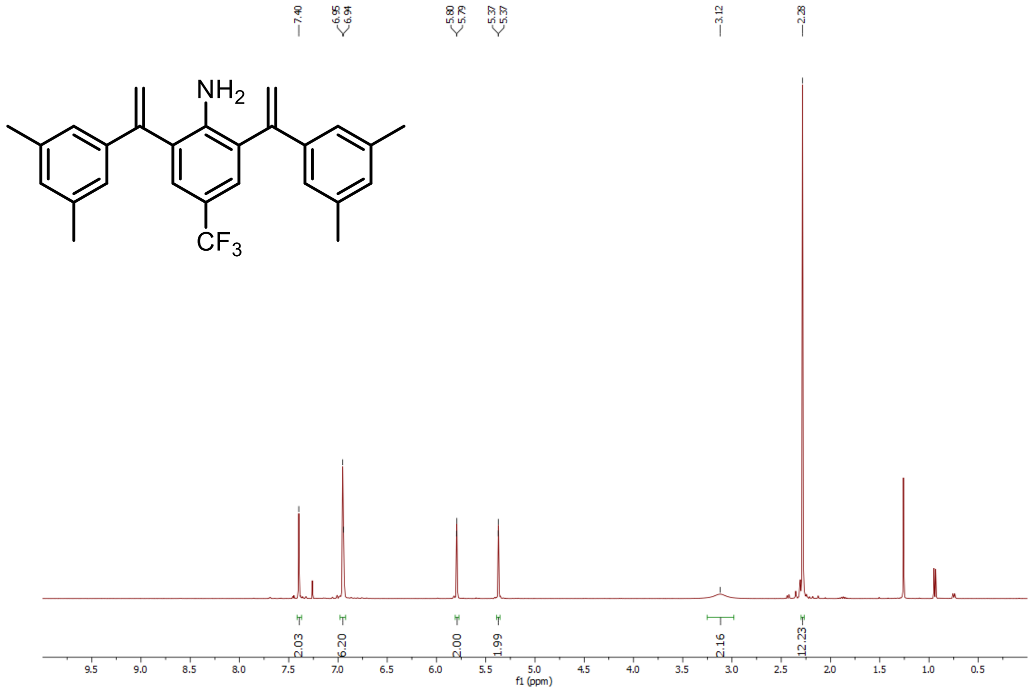

^{13}C NMR (101 MHz, Chloroform-*d*):

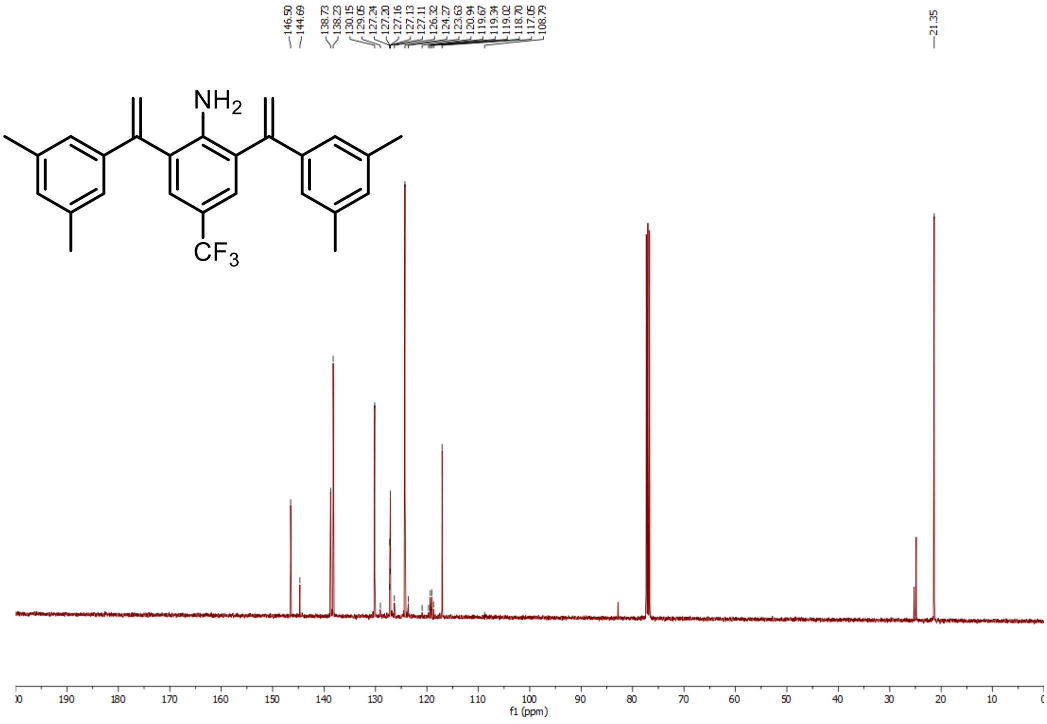

^{19}**F NMR** (376 MHz, Chloroform-*d*):

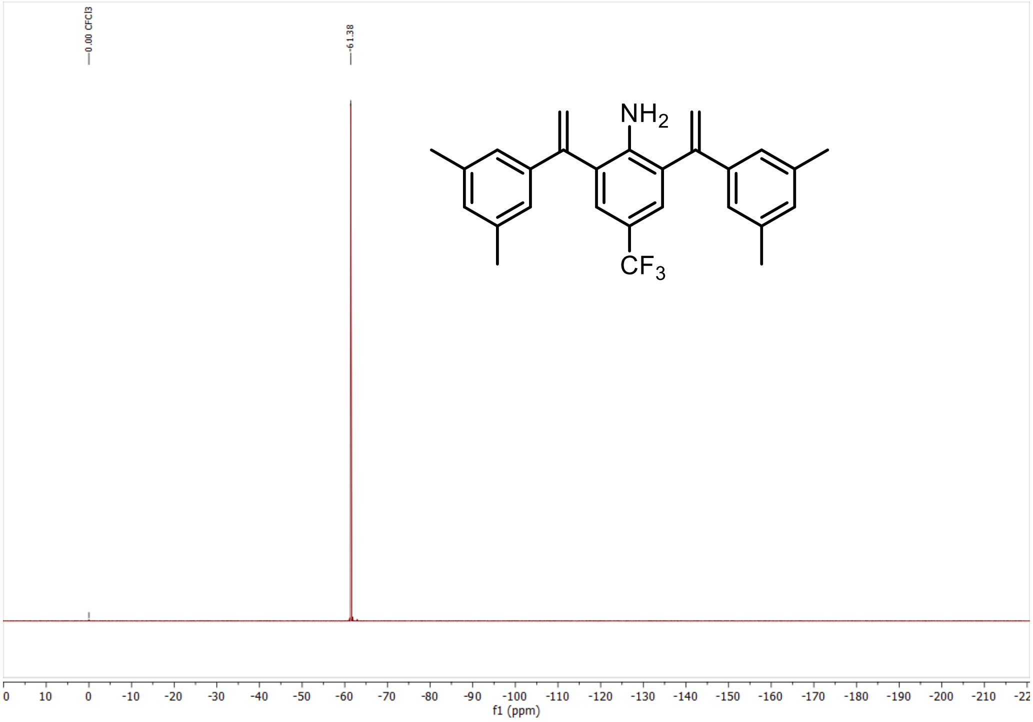

2,6-Bis(1-(3,5-di-*tert*-butylphenyl)vinyl)-4-(trifluoromethyl)aniline (9d)

^{1}H NMR (400 MHz, Chloroform-*d*):

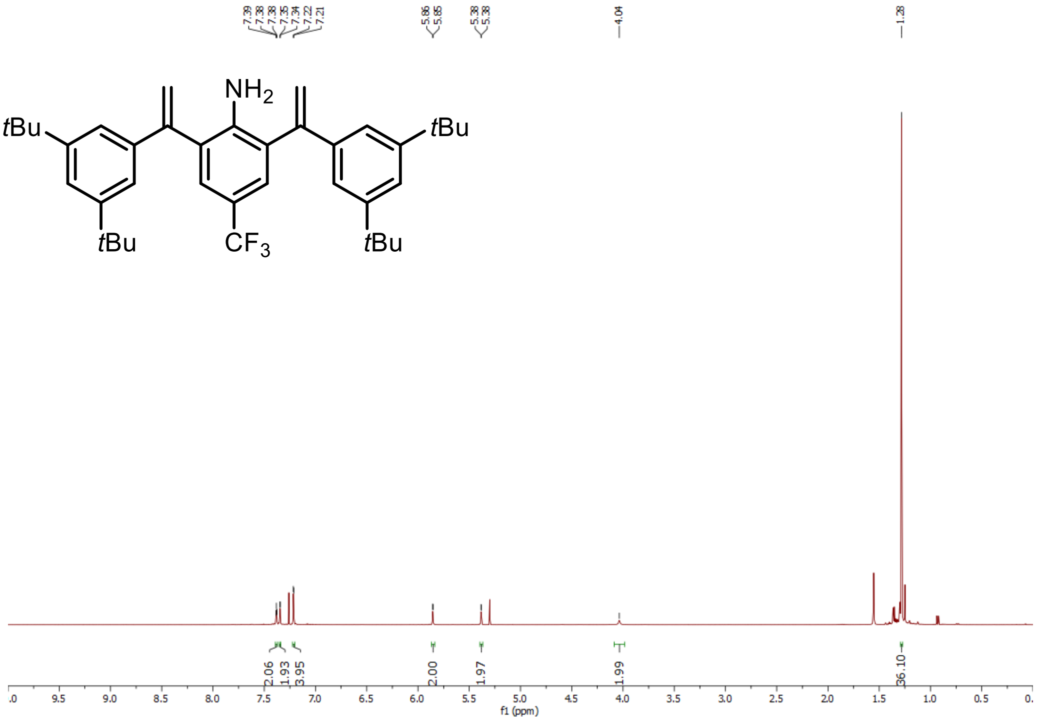

^{13}C NMR (101 MHz, Chloroform-*d*):

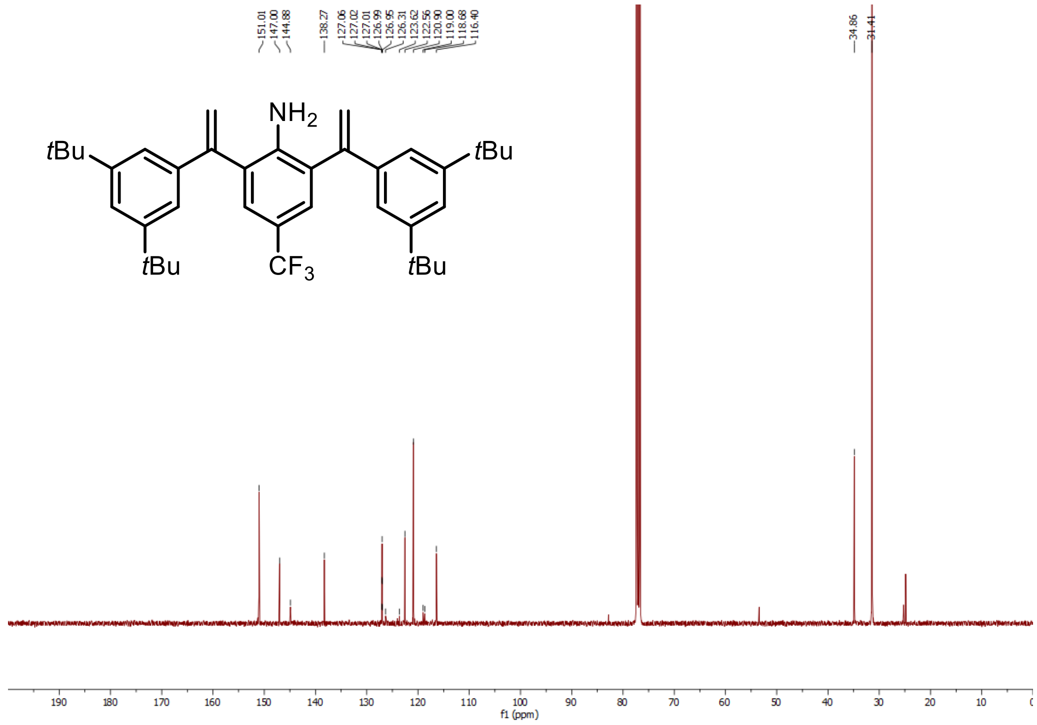

^{19}F NMR (376 MHz, Chloroform-*d*):

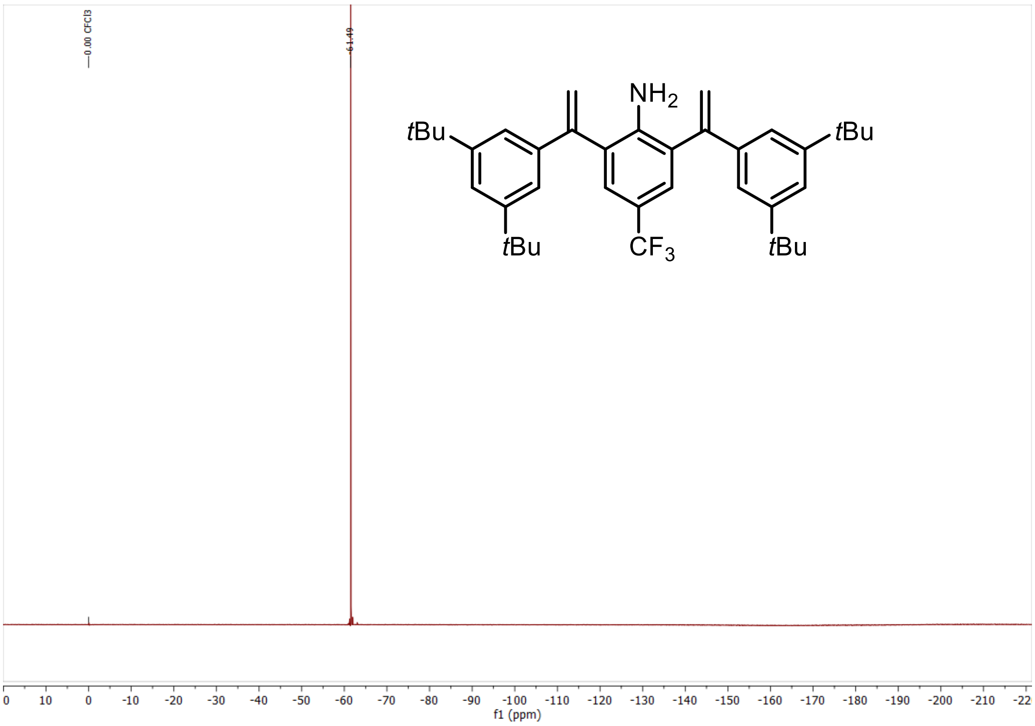

2,6-Bis((*R*)-1-(3,5-dimethylphenyl)ethyl)-4-(trifluoromethyl)aniline (10e)

^{1}H NMR (400 MHz, Chloroform-*d*):

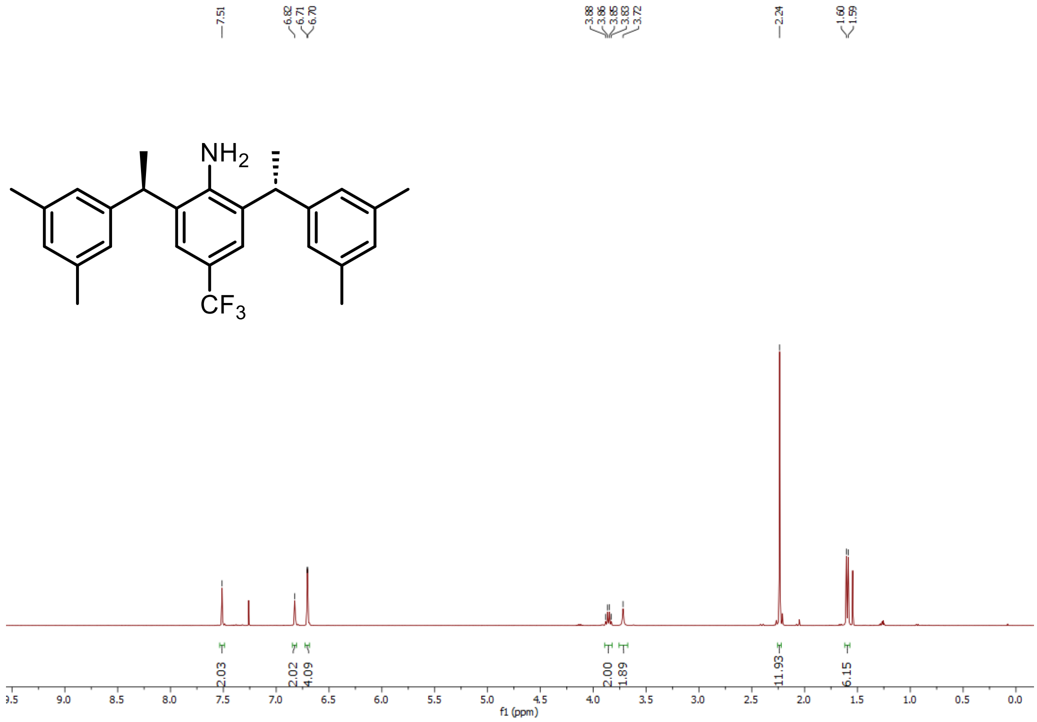

^{13}C NMR (101 MHz, Chloroform-*d*):

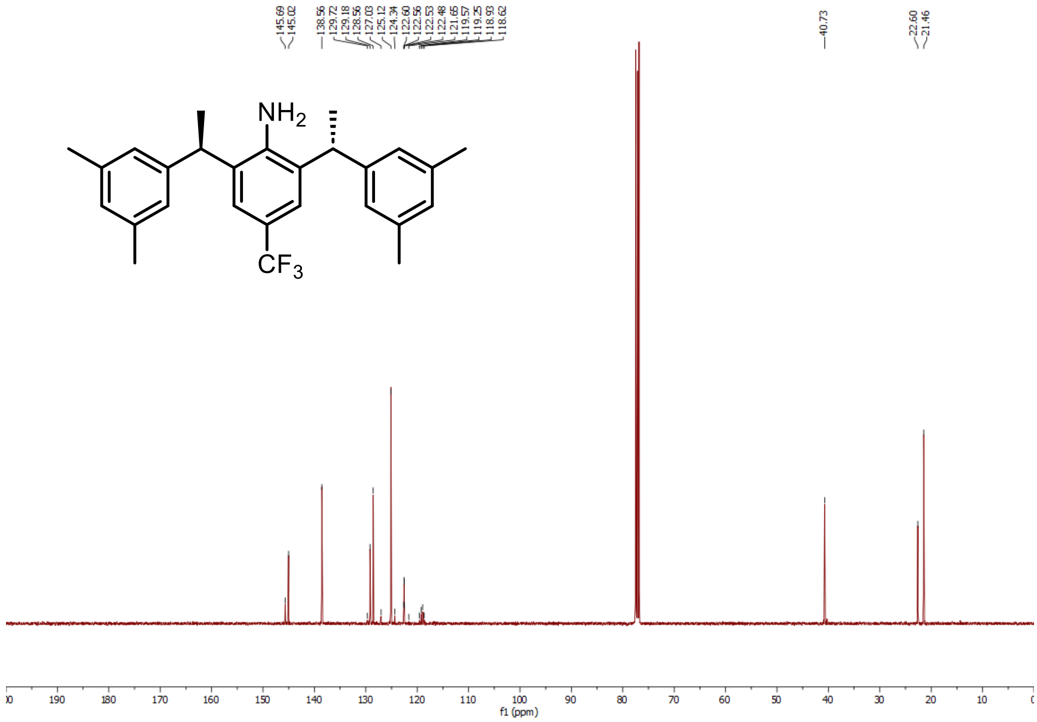

^{19}F NMR (376 MHz, Chloroform-*d*):

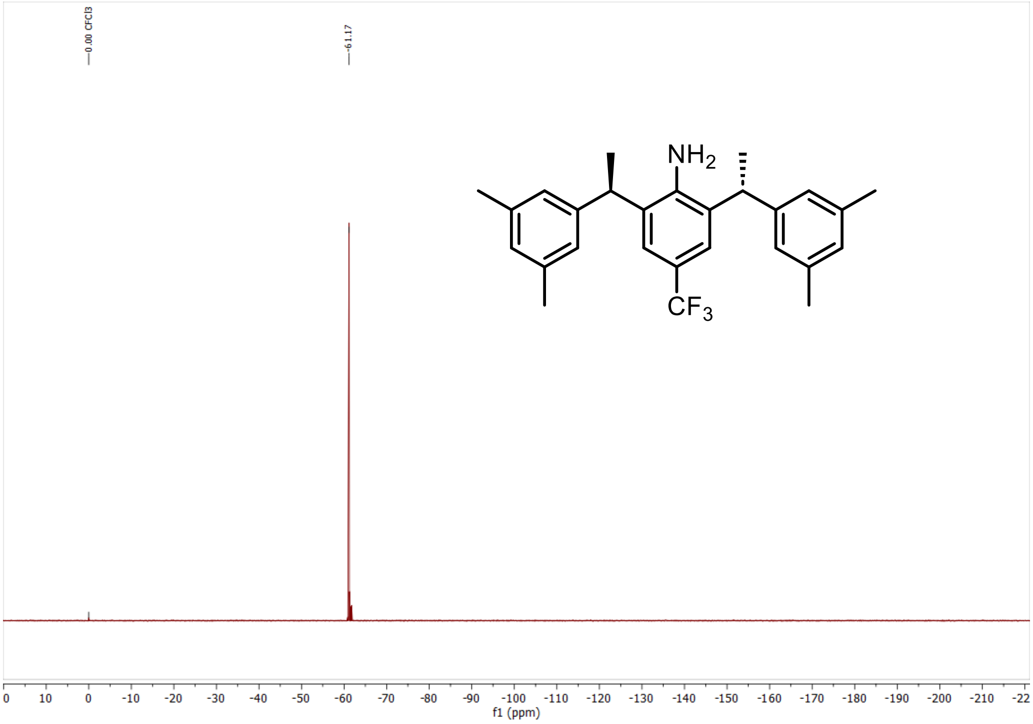

2,6-Bis((*R*)-1-(3,5-di-*tert*-butylphenyl)ethyl)-4-(trifluoromethyl)aniline (10f)

^{1}H NMR (400 MHz, Chloroform-*d*):

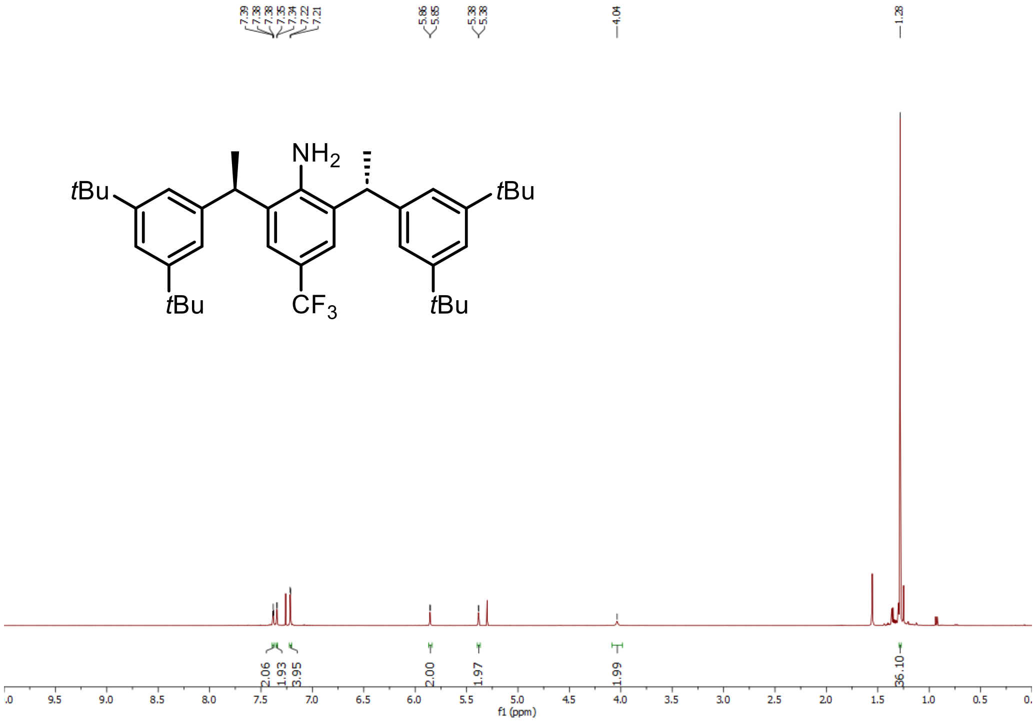

^{13}C NMR (101 MHz, Chloroform-*d*):

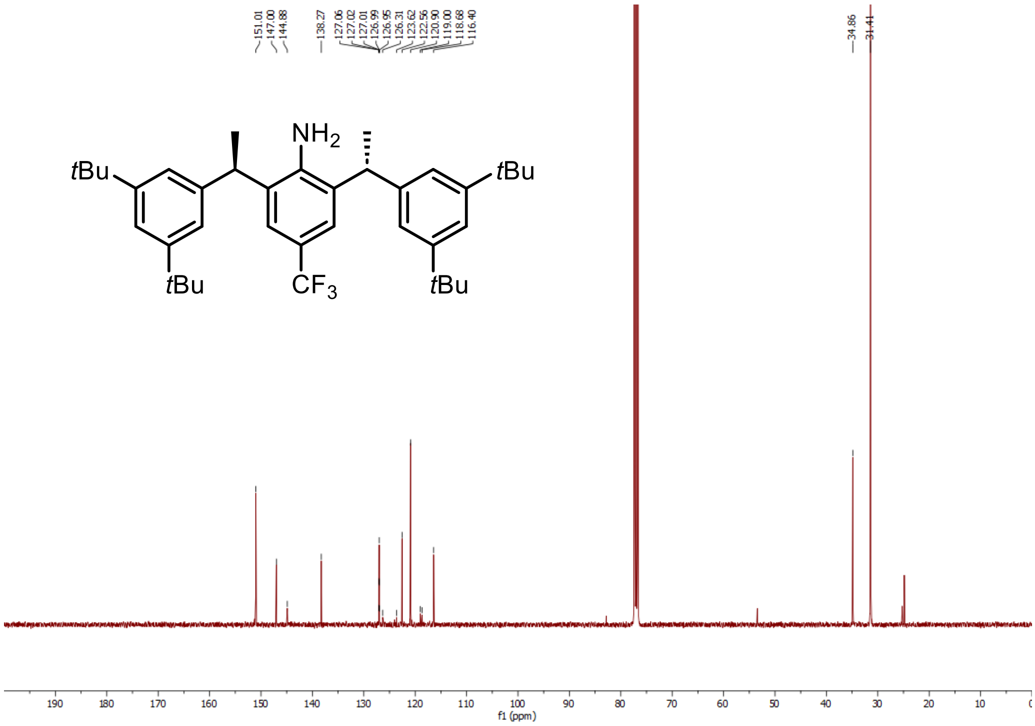

^{19}F NMR (376 MHz, Chloroform-*d*):

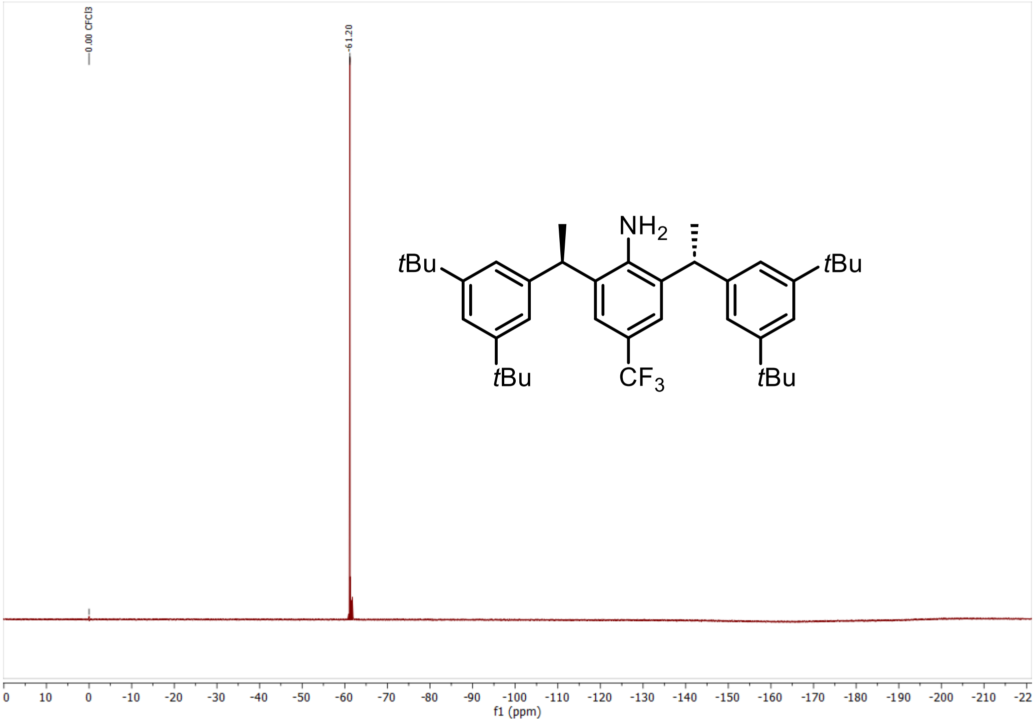

3-(2,6-Bis((*R*)-1-phenylethyl)phenyl)-5,6,7,8-tetrahydro-4*H*-cyclohepta[*d*]thiazol-3-ium perchlorate (NHC1)

^{1}H NMR (400 MHz, Chloroform-*d*):

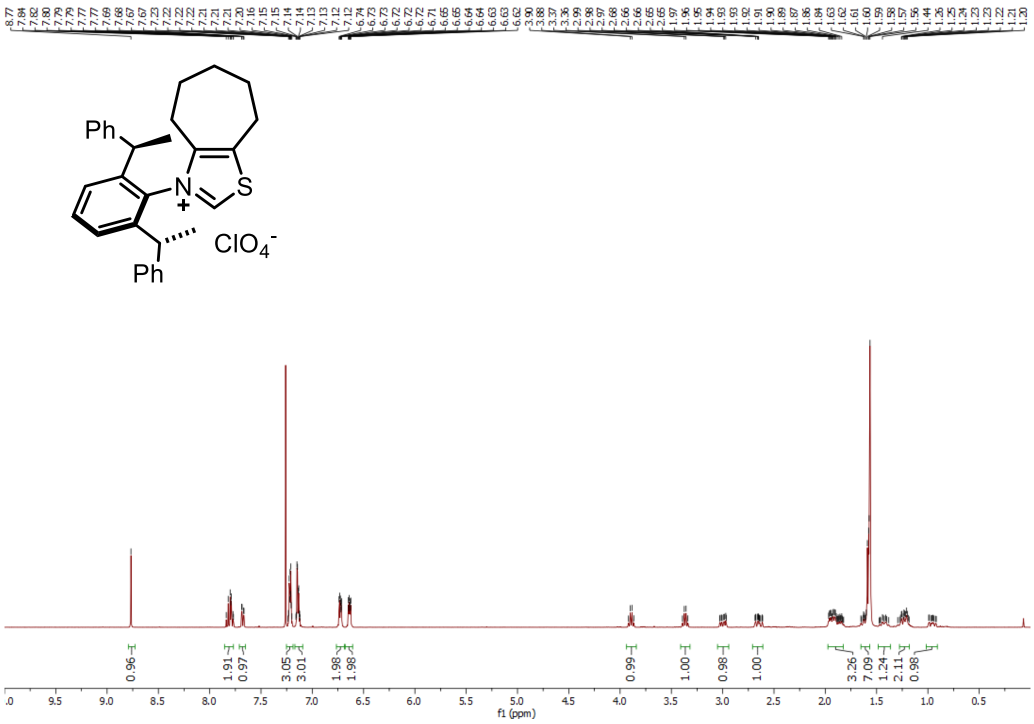

^{13}C NMR (101 MHz, Chloroform-*d*):

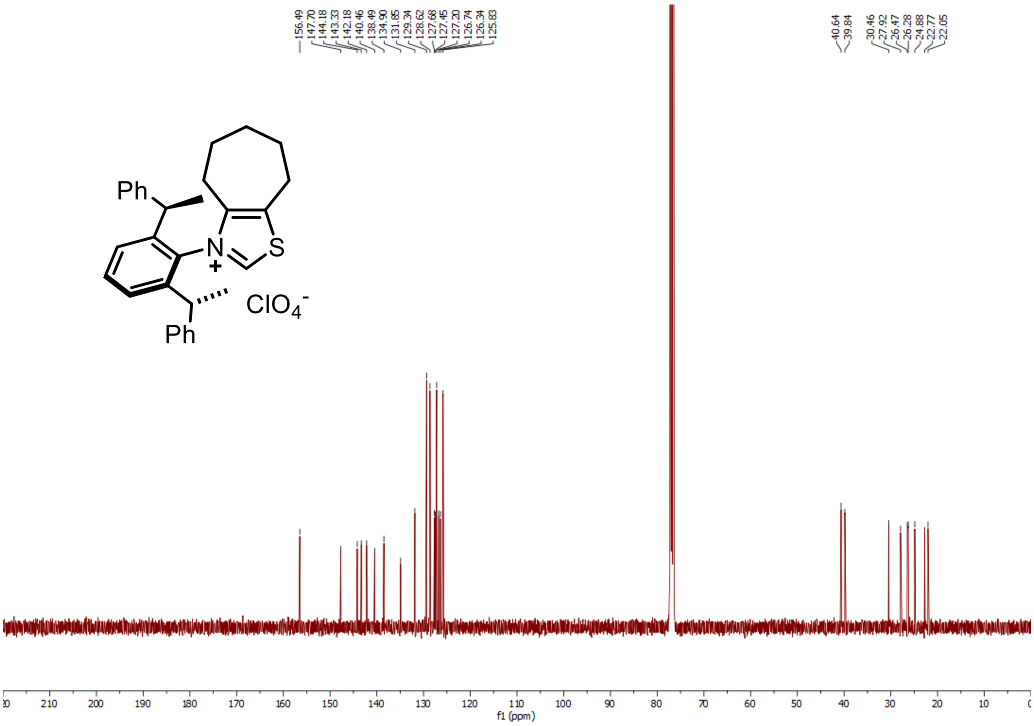

3-(4-Methoxy-2,6-bis((*R*)-1-phenylethyl)phenyl)-5,6,7,8-tetrahydro-4*H*-cyclohepta[*d*]thiazol-3-ium perchlorate (NHC2)

^{1}H NMR (400 MHz, Chloroform-*d*):

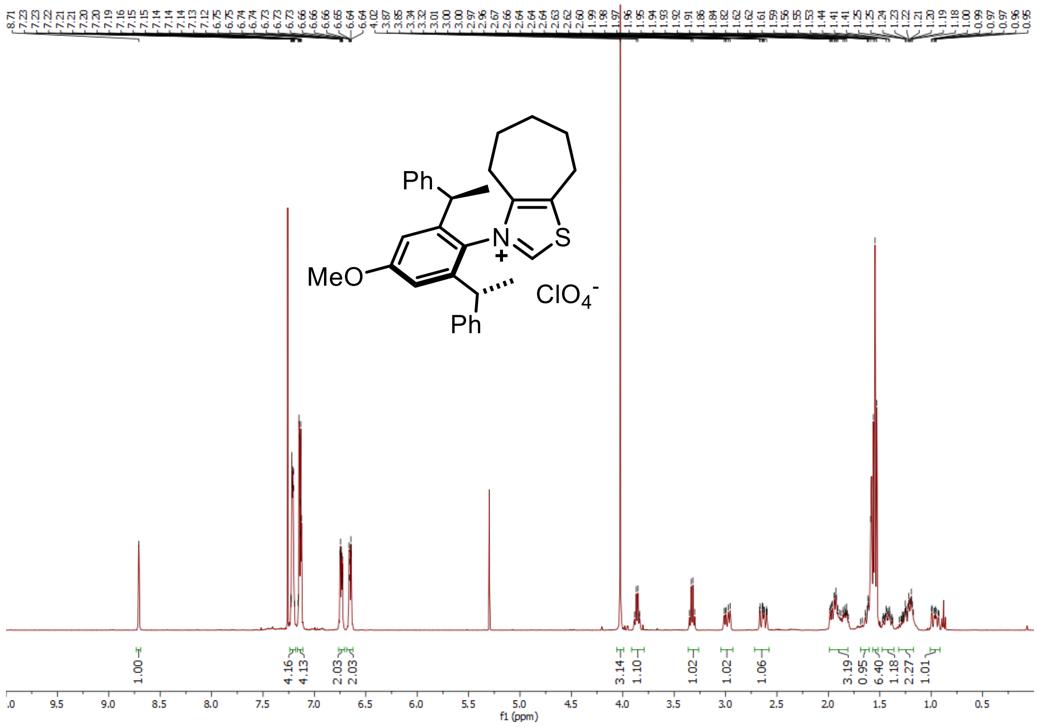

^{13}C NMR (101 MHz, Chloroform-*d*):

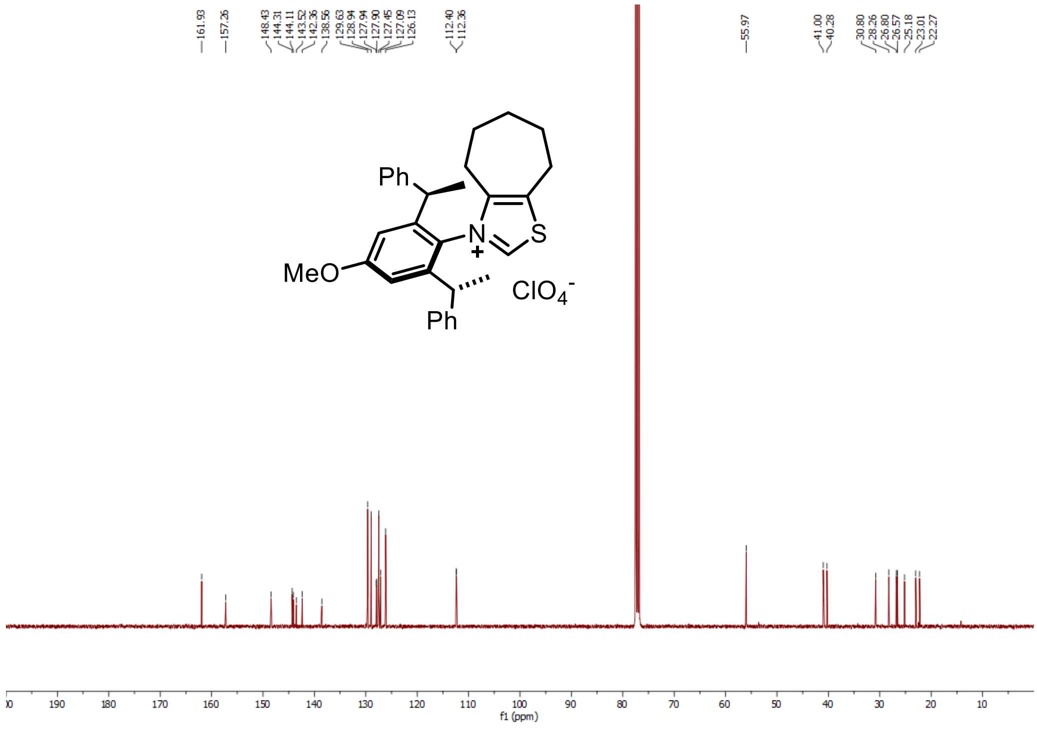

3-(2,6-Bis((*R*)-1-phenylethyl)-4-(trifluoromethyl)phenyl)-5,6,7,8-tetrahydro-*4H*-cyclohepta[*d*]thiazol-3-ium perchlorate (NHC3)

^{1}H NMR (400 MHz, Chloroform-*d*):

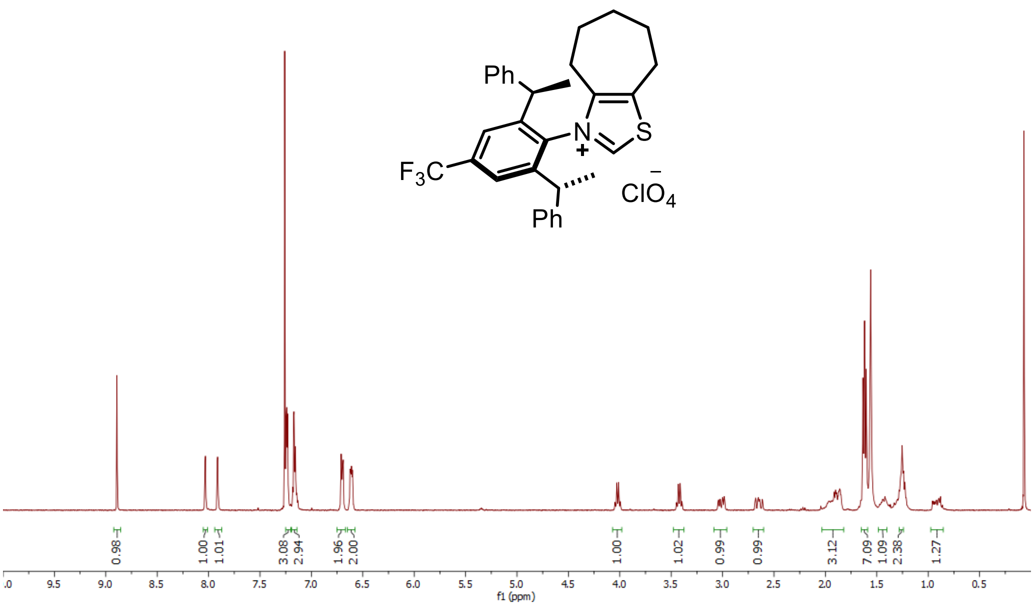

^{13}C NMR (101 MHz, Chloroform-*d*):

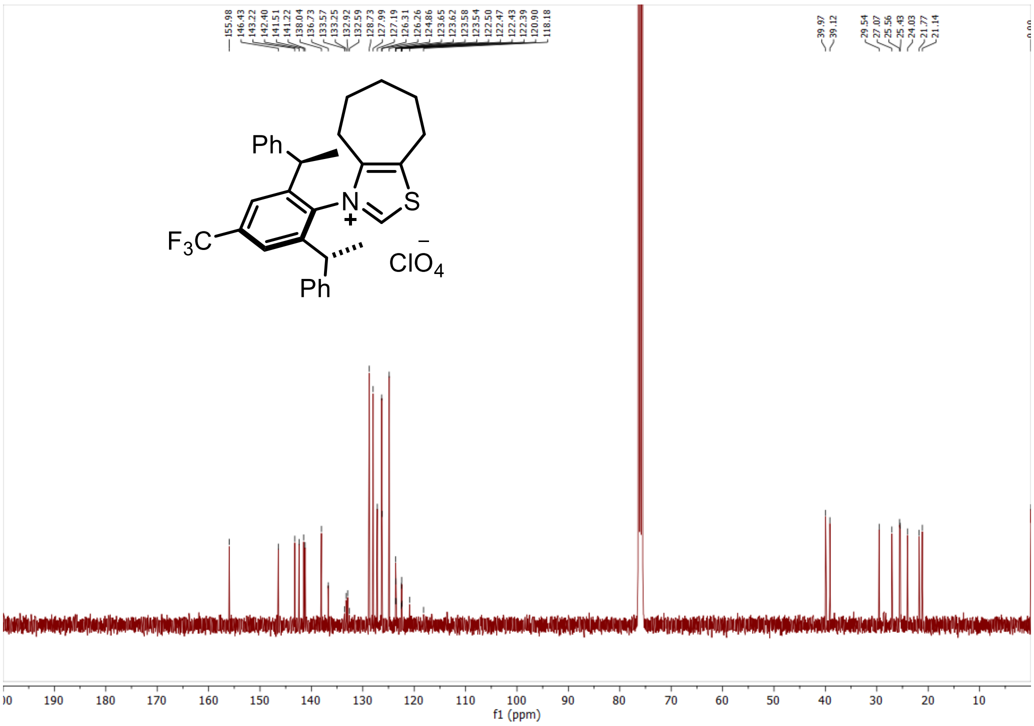

^{19}F NMR (376 MHz, Chloroform-*d*):

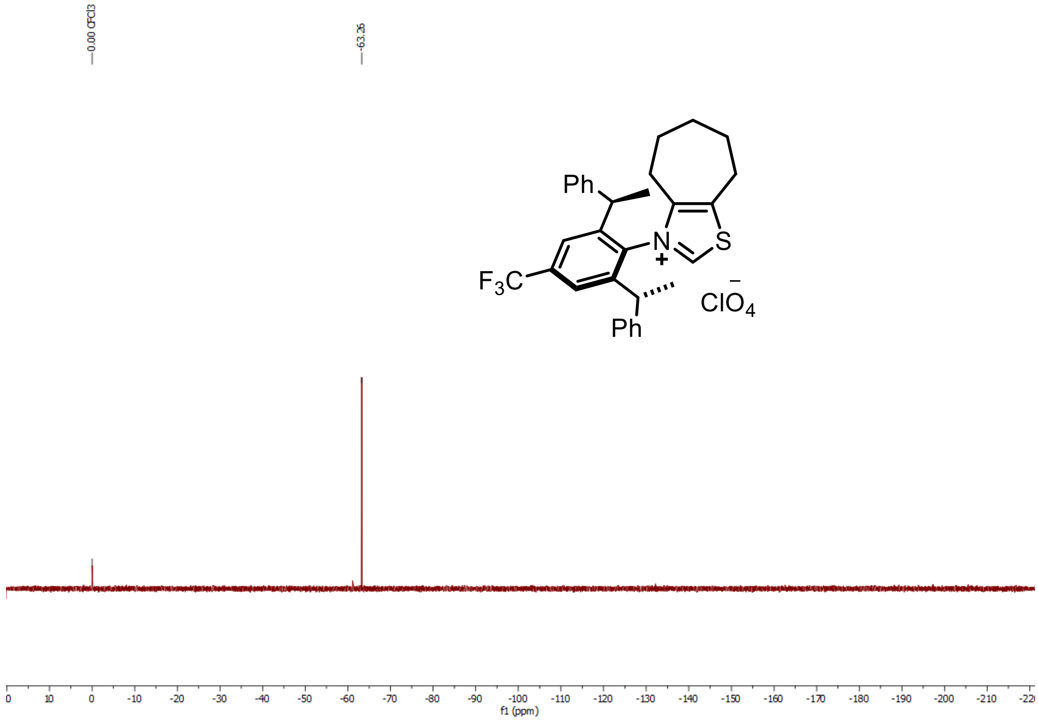

3-(2,6-Bis((*R*)-1-phenylethyl)phenyl)-4,5,6,7,8,9-hexahydrocycloocta[*d*]thiazol-3-ium perchlorate (NHC4)

^{1}H NMR (400 MHz, Chloroform-*d*):

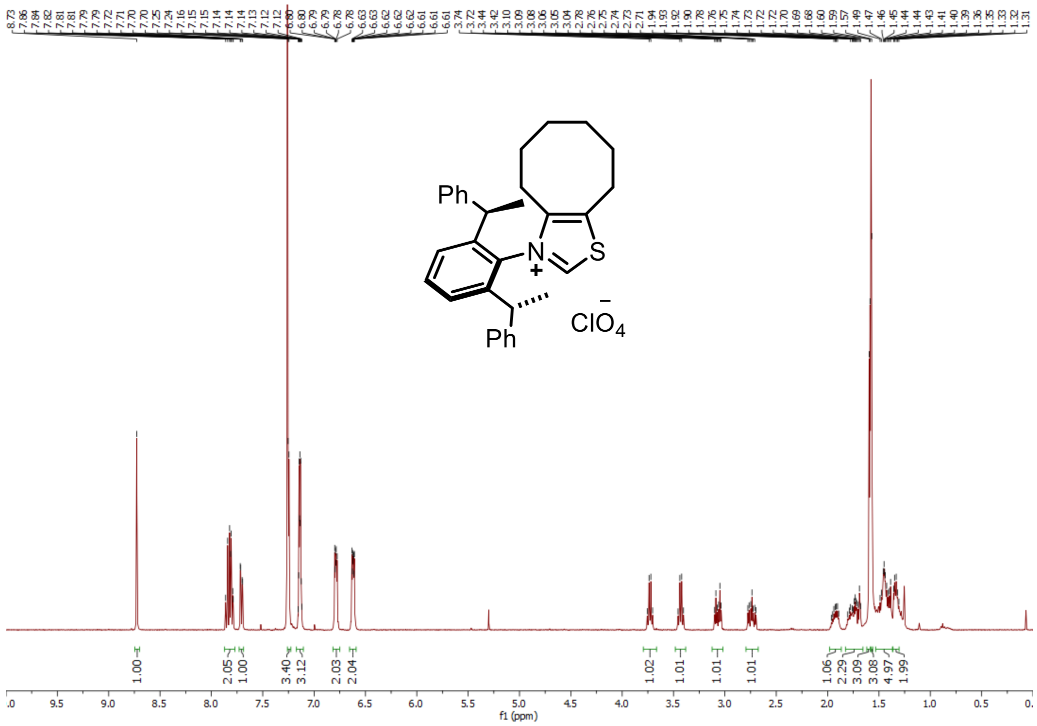

^{13}C NMR (101 MHz, $CDCl_3$):

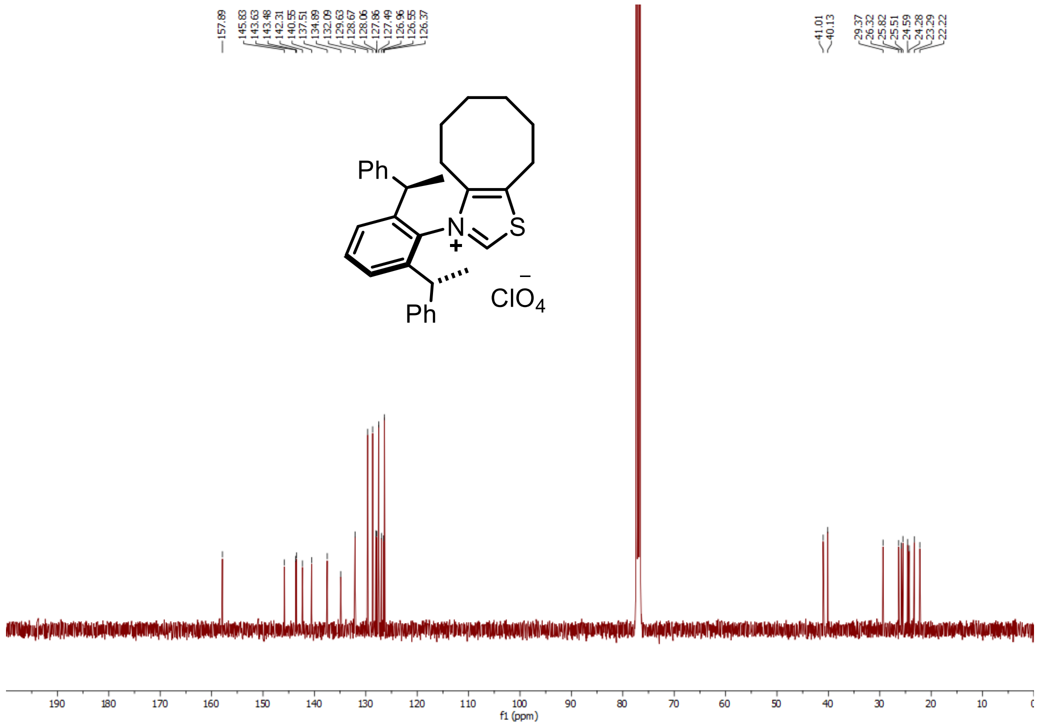

3-(2,6-Bis((*R*)-1-phenylethyl)-4-(trifluoromethyl)phenyl)-4,5,6,7,8,9,10,11,12,13-decahydrocyclododeca[d]thiazol-3-ium perchlorate (NHC5)

^{1}H NMR (400 MHz, Chloroform-*d*):

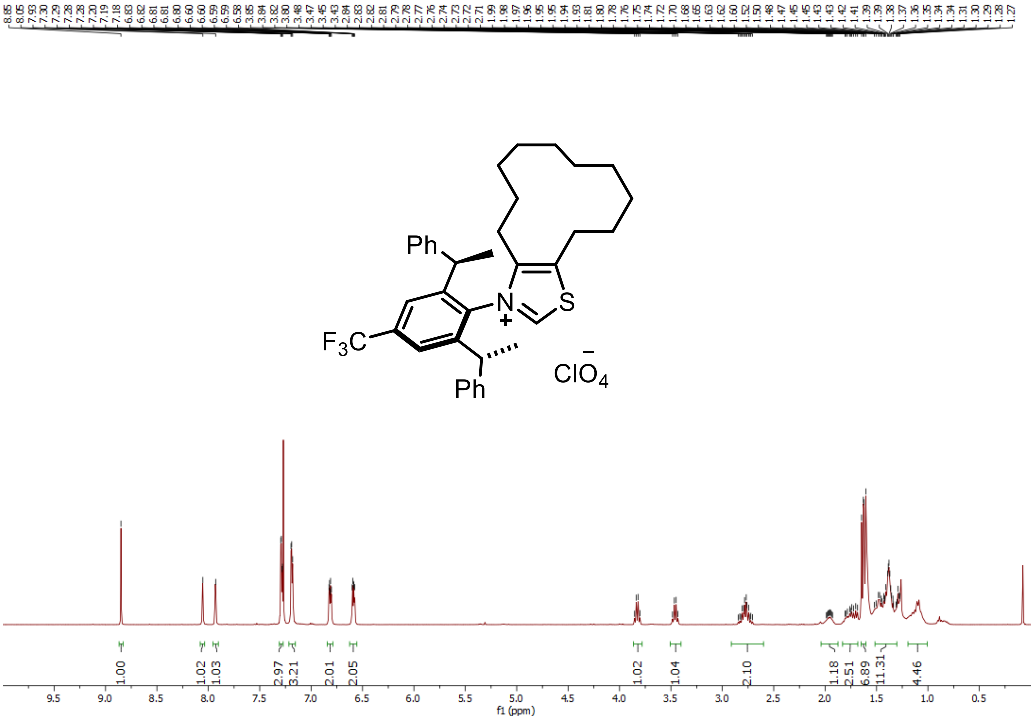

^{13}C NMR (101 MHz, Chloroform-*d*):

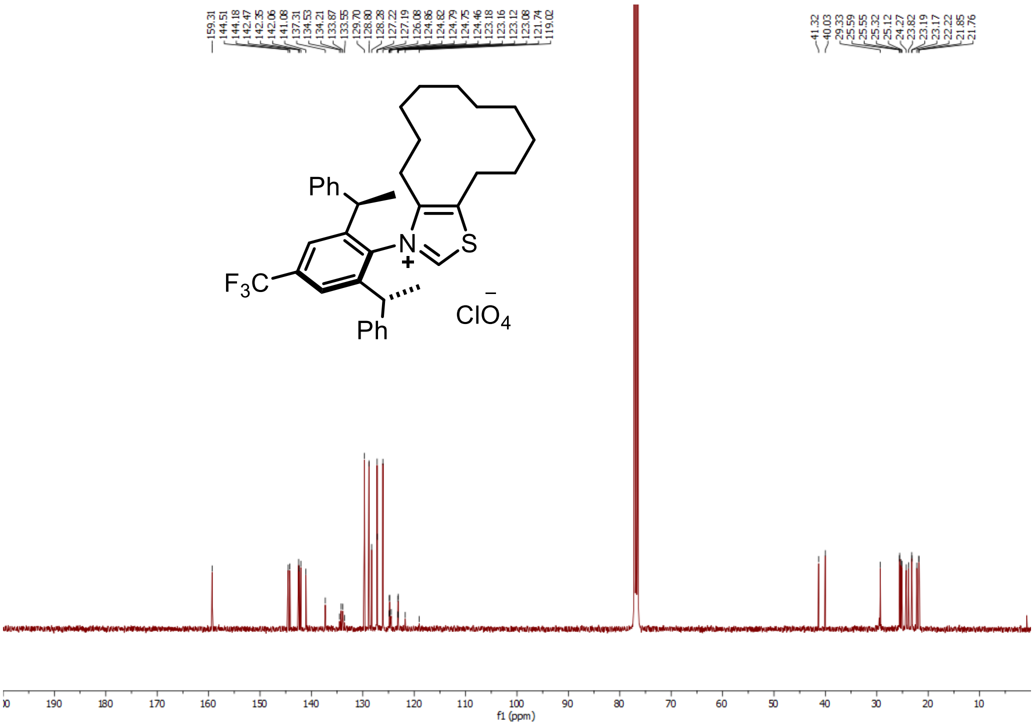

^{19}F NMR (376 MHz, Chloroform-*d*):

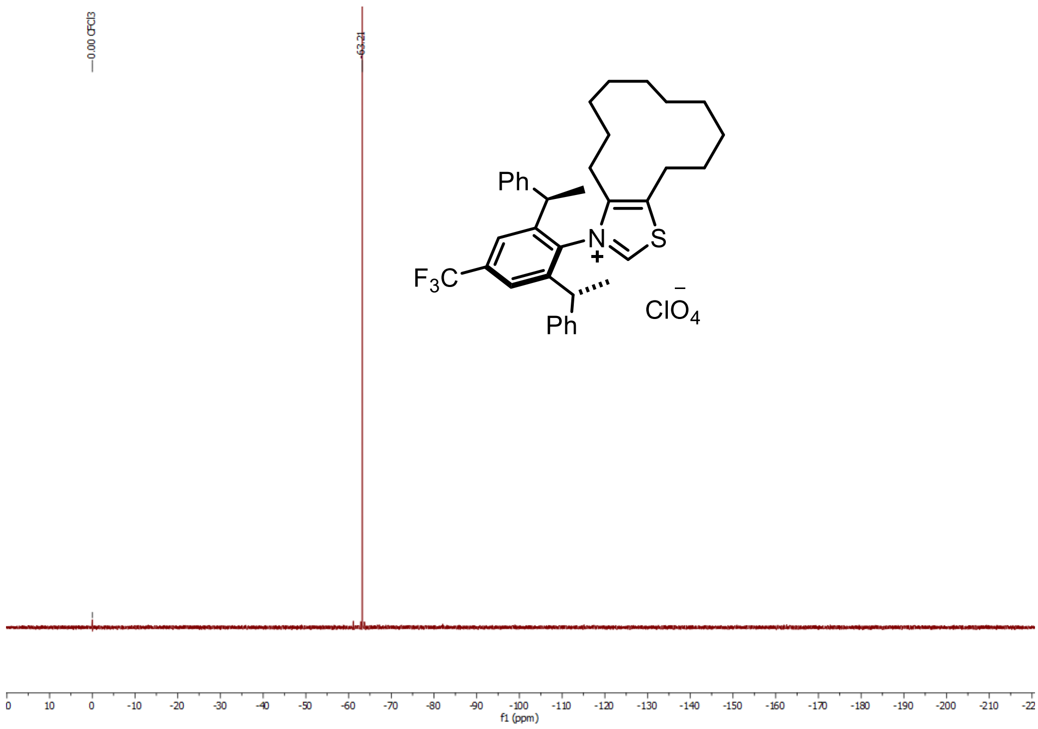

3-(2,6-Bis((*R*)-1-(3,5-dimethylphenyl)ethyl)-4-(trifluoromethyl)phenyl)-4,5,6,7,8,9,10,11,12,13-decahydrocyclododeca[d]thiazol-3-ium perchlorate (NHC6)

^{1}H NMR (400 MHz, Chloroform-*d*):

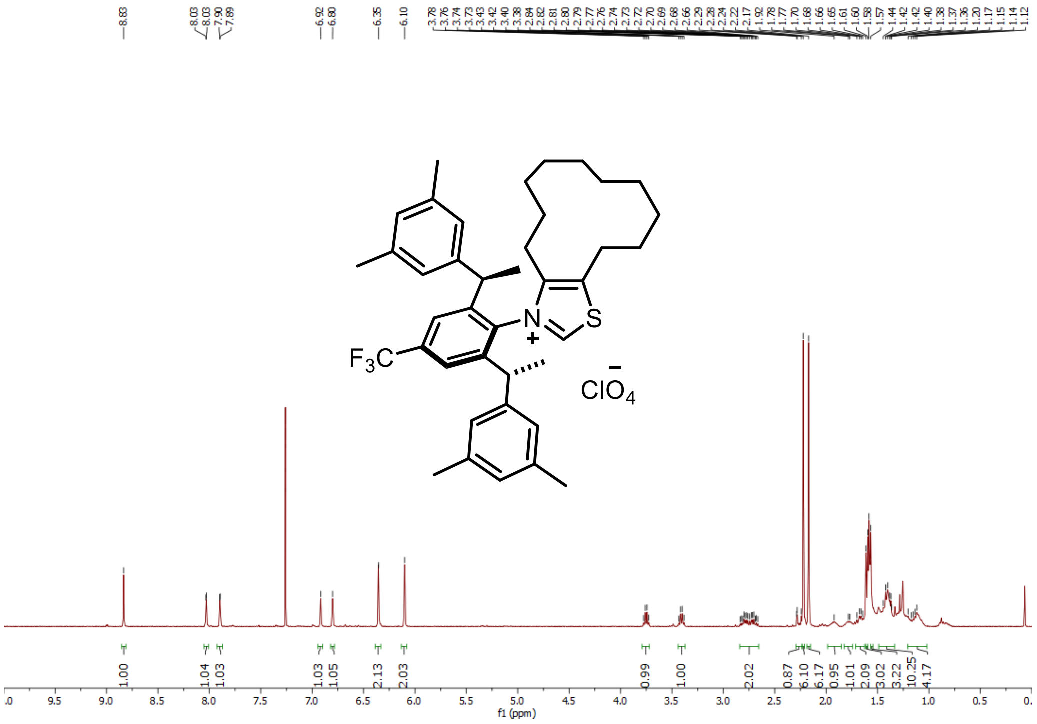

^{13}C NMR (101 MHz, Chloroform-*d*):

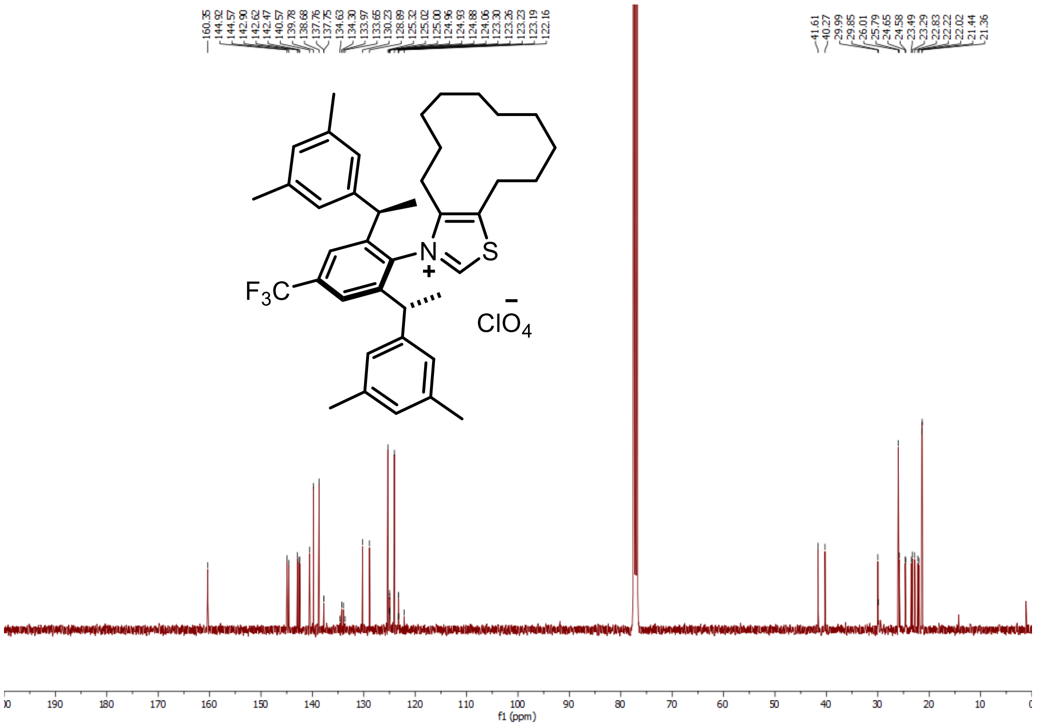

^{19}F NMR (376 MHz, Chloroform-*d*):

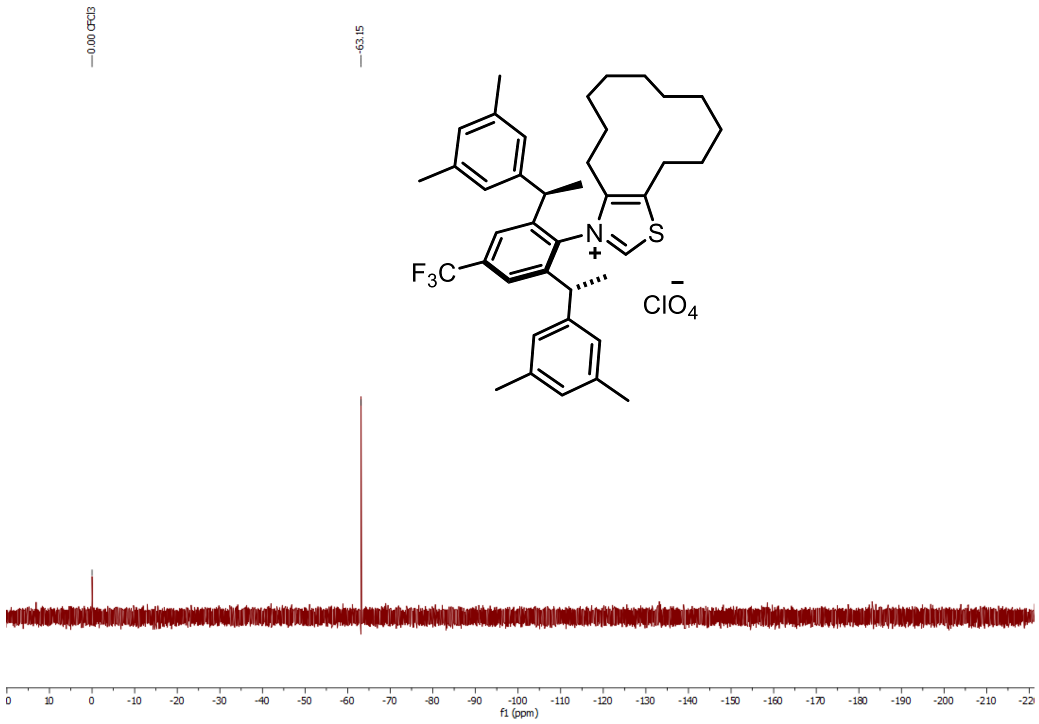

3-(2,6-Bis((*R*)-1-(3,5-di-*tert*-butylphenyl)ethyl)-4-(trifluoromethyl)phenyl)-4,5,6,7,8,9,10,11,12,13-decahydrocyclododeca[*d*]thiazol-3-ium perchlorate (NHC7)

^{1}H NMR (400 MHz, Chloroform-*d*):

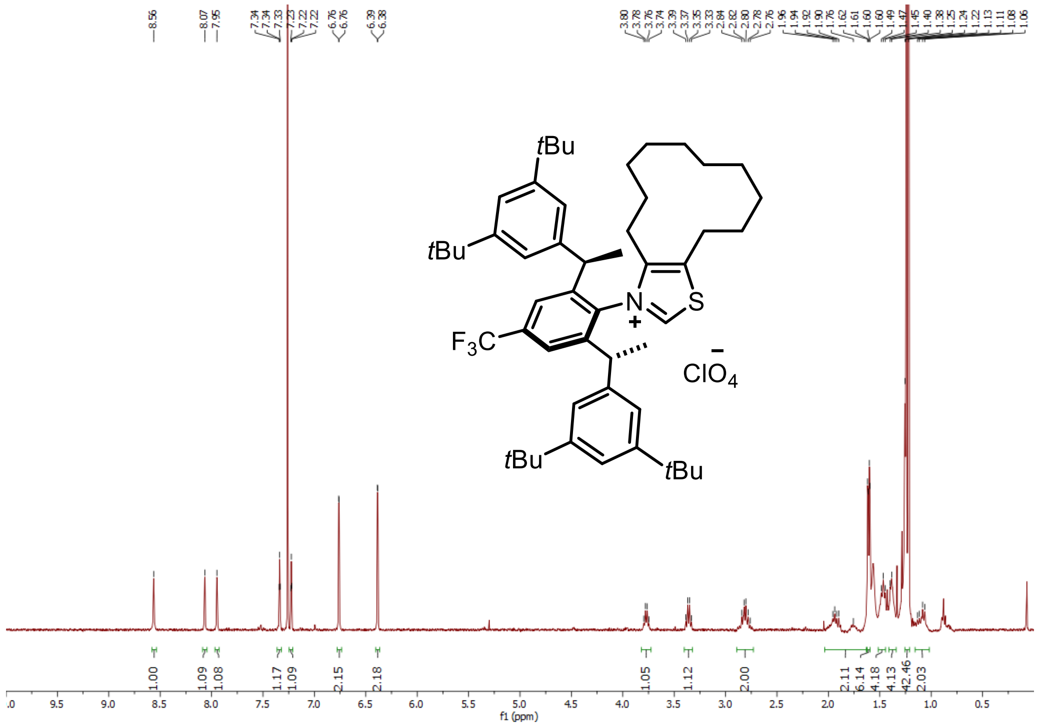

^{13}C NMR (101 MHz, Chloroform-*d*):

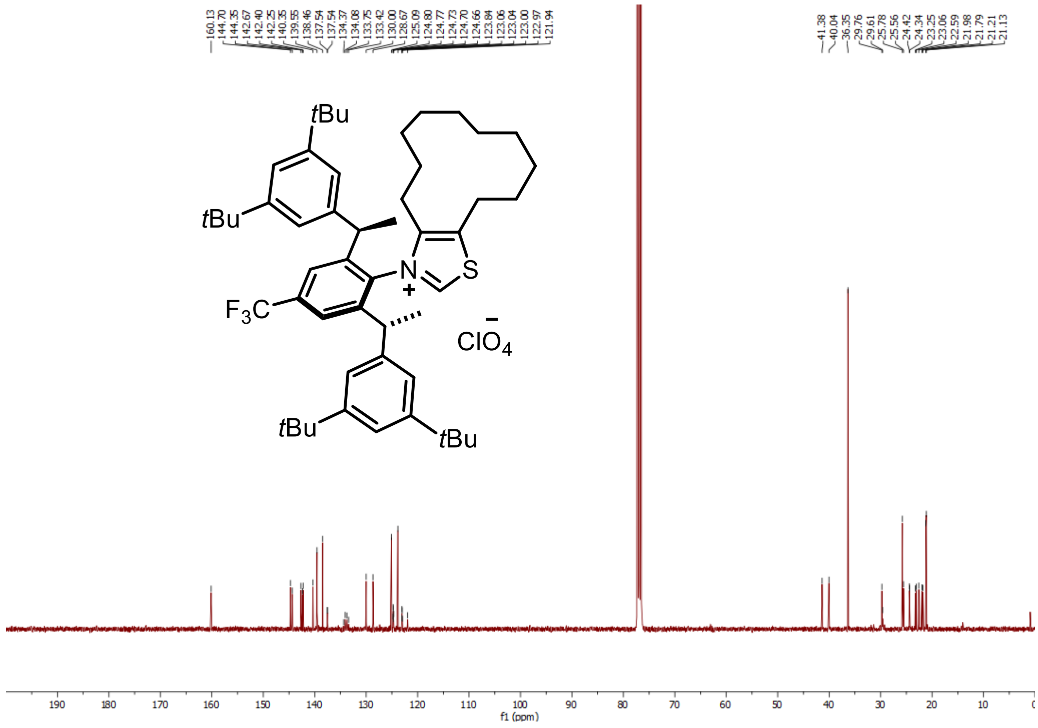

^{19}F NMR (376 MHz, Chloroform-*d*):

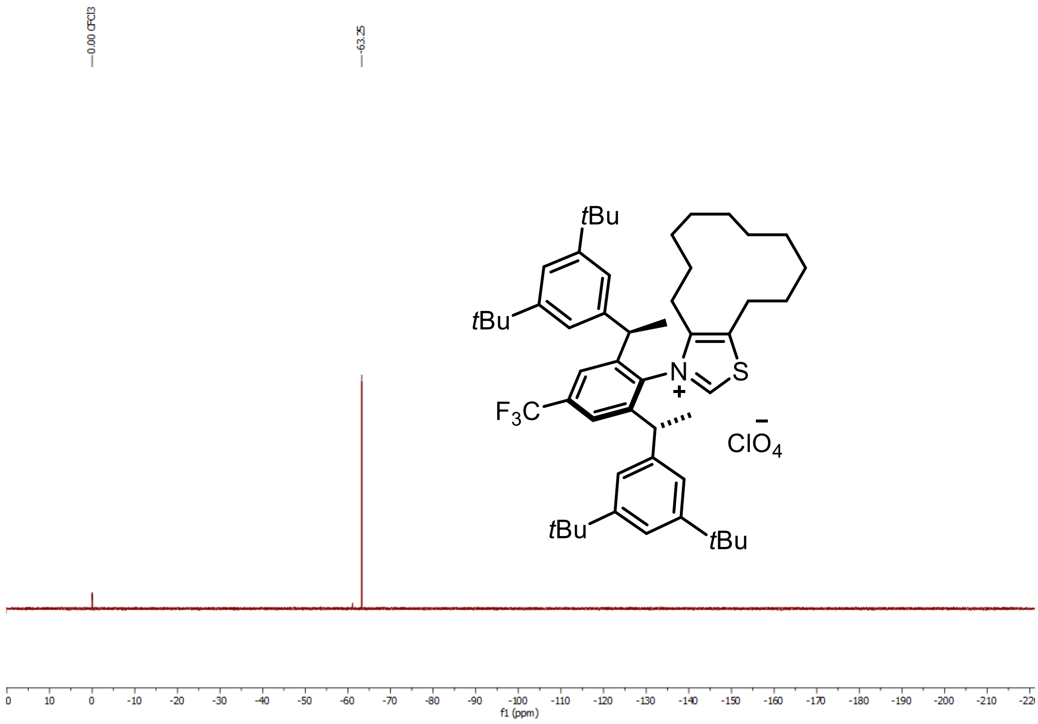

3-(4-Methyl-2,6-bis((*R*)-1-phenylethyl)phenyl)-5,6,7,8-tetrahydro-4H-cyclohepta[*d*]thiazol-3-ium perchlorate (NHC10)

^{1}H NMR (400 MHz, Chloroform-*d*):

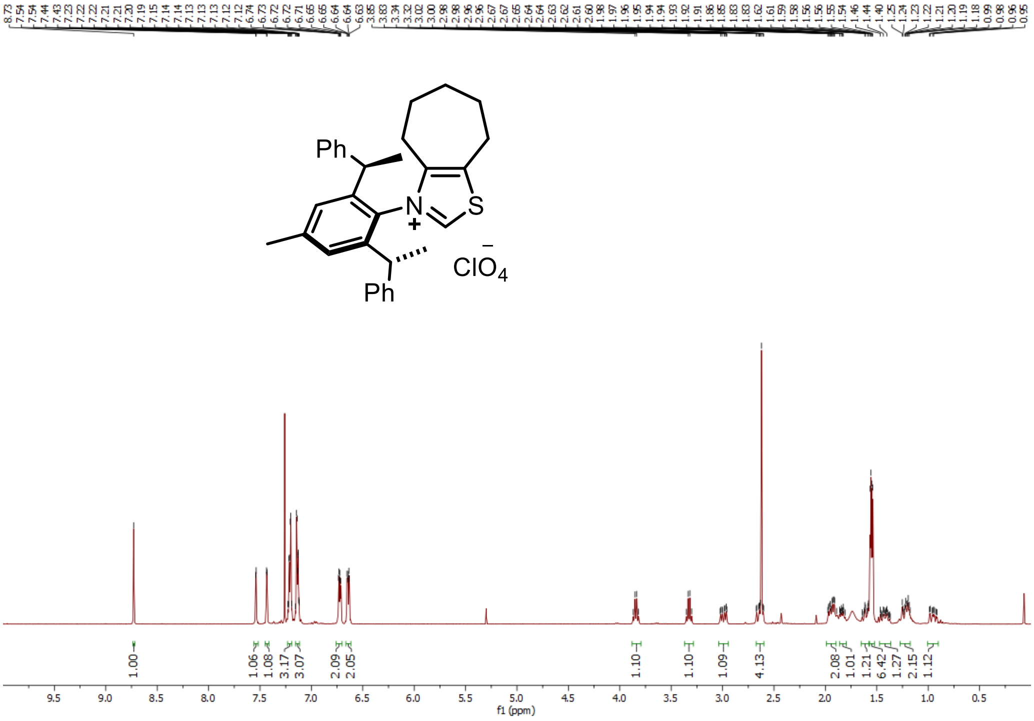

^{13}C NMR (101 MHz, Chloroform-*d*):

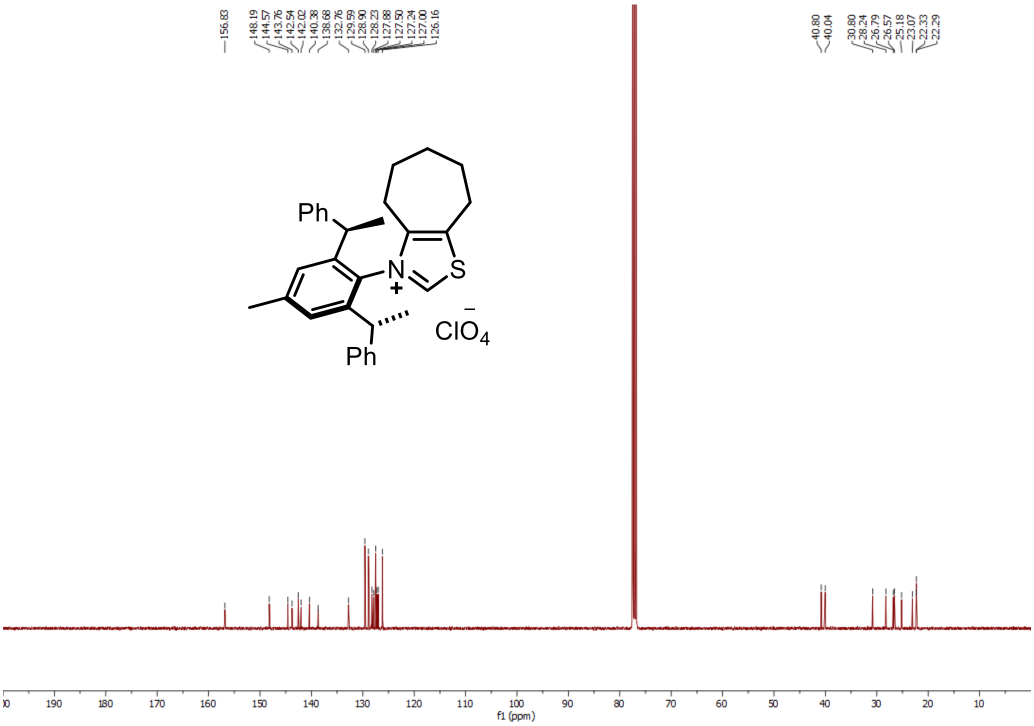

3-(2,6-Bis((*R*)-1-(3,5-dimethylphenyl)ethyl)phenyl)-5,6,7,8-tetrahydro-4*H*-cyclohepta[d]thiazol-3-ium perchlorate (NHC11)

^{1}H NMR (400 MHz, Chloroform-*d*):

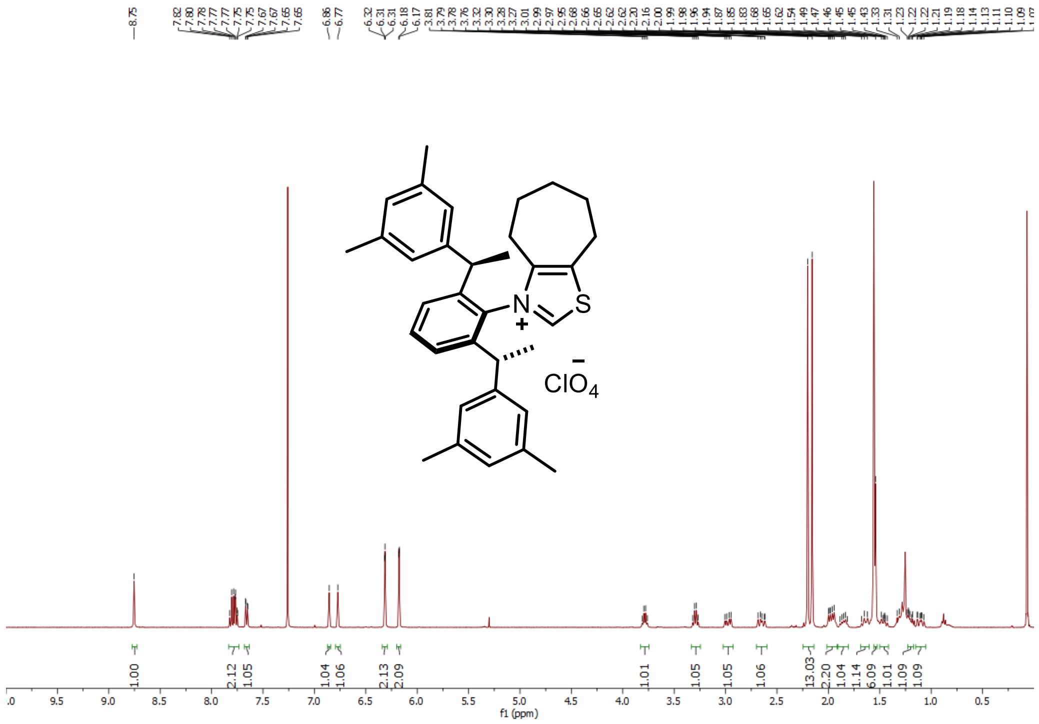

^{13}C NMR (101 MHz, Chloroform-*d*):

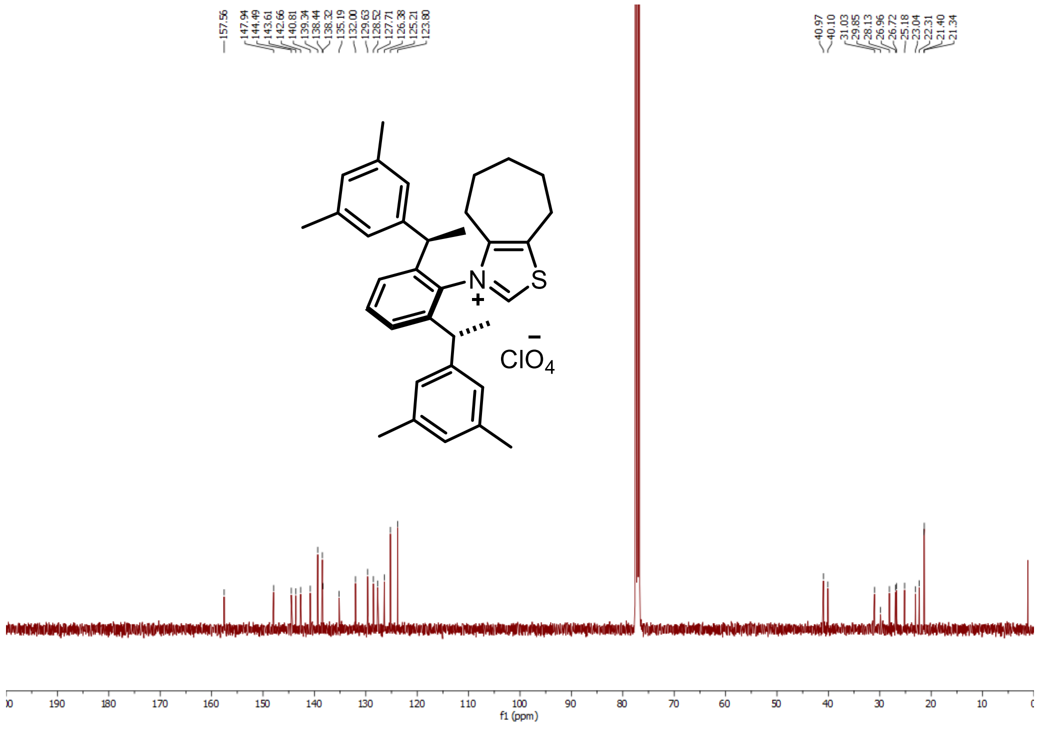

3-(4-Methyl-2,6-bis((*R*)-1-phenylethyl)phenyl)-4,5,6,7,8,9,10,11,12,13-decahydrocyclododeca[d]thiazol-3-ium perchlorate (NHC12)

^{1}H NMR (400 MHz, Chloroform-*d*):

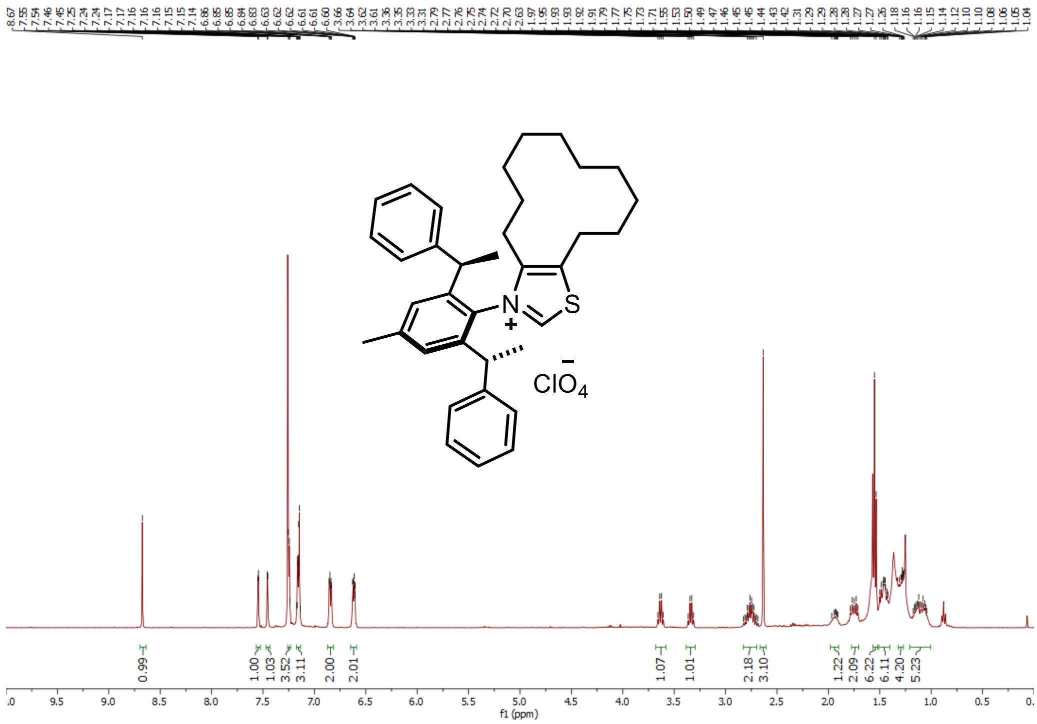

^{13}C NMR (101 MHz, Chloroform-*d*):

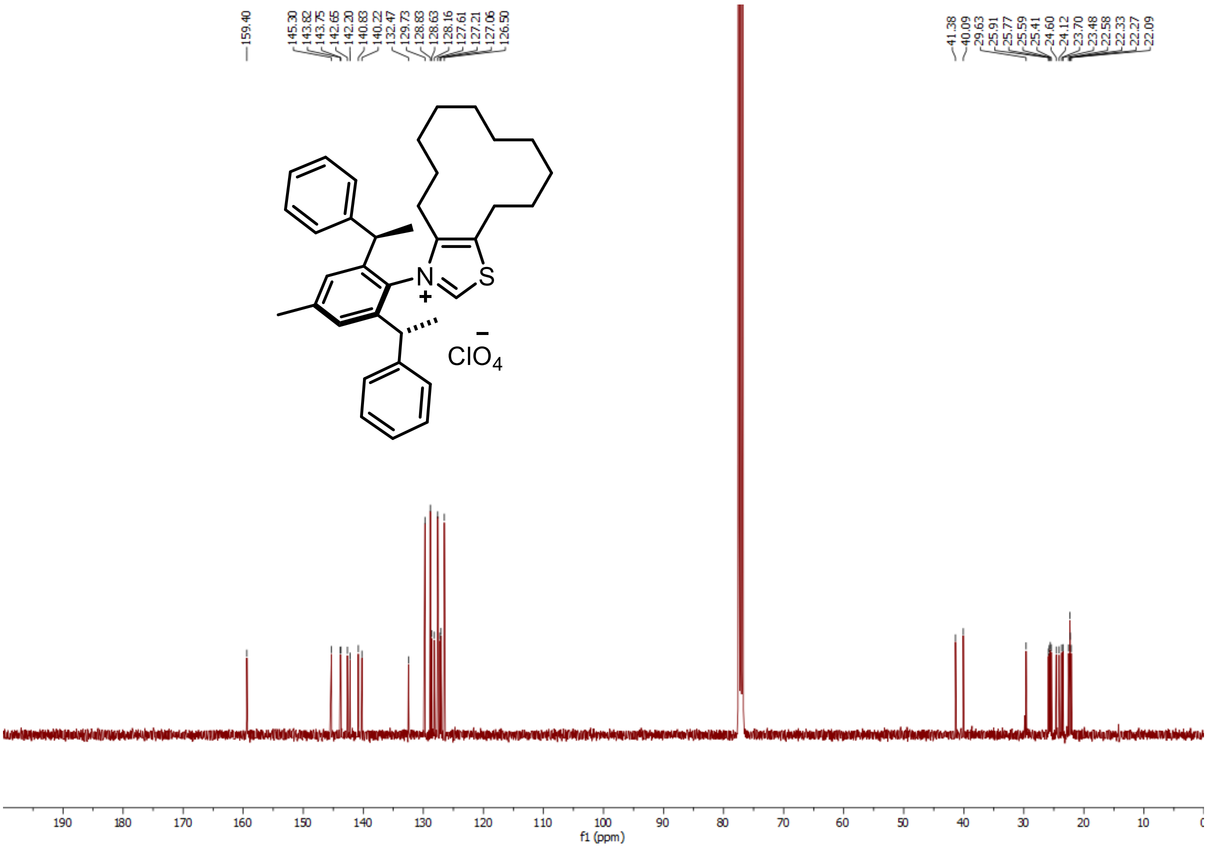

Isopropyl 2-methyl-2-(4-(4-vinylbenzoyl)phenoxy)propanoate (5a)

^{1}H NMR (400 MHz, Chloroform-*d*):

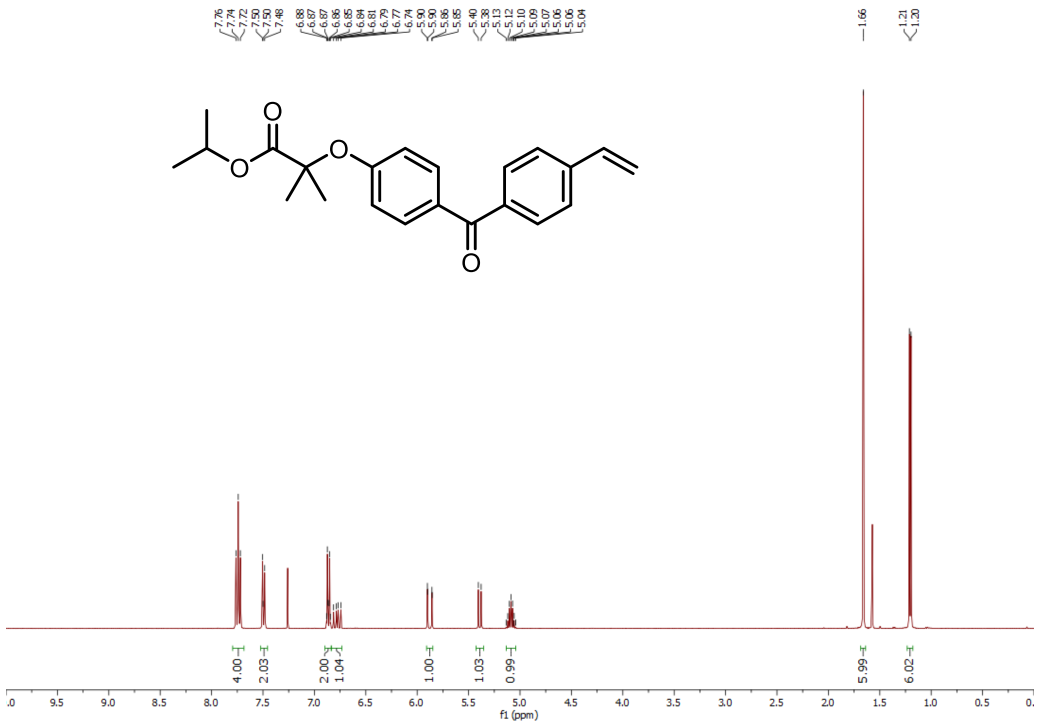

(8*R*,9*S*,13*S*,14*S*)-13-Methyl-3-vinyl-6,7,8,9,11,12,13,14,15,16-decahydro-17*H*-cyclopenta[*a*]phenanthren-17-one (5b)

^{1}H NMR (400 MHz, Chloroform-*d*):

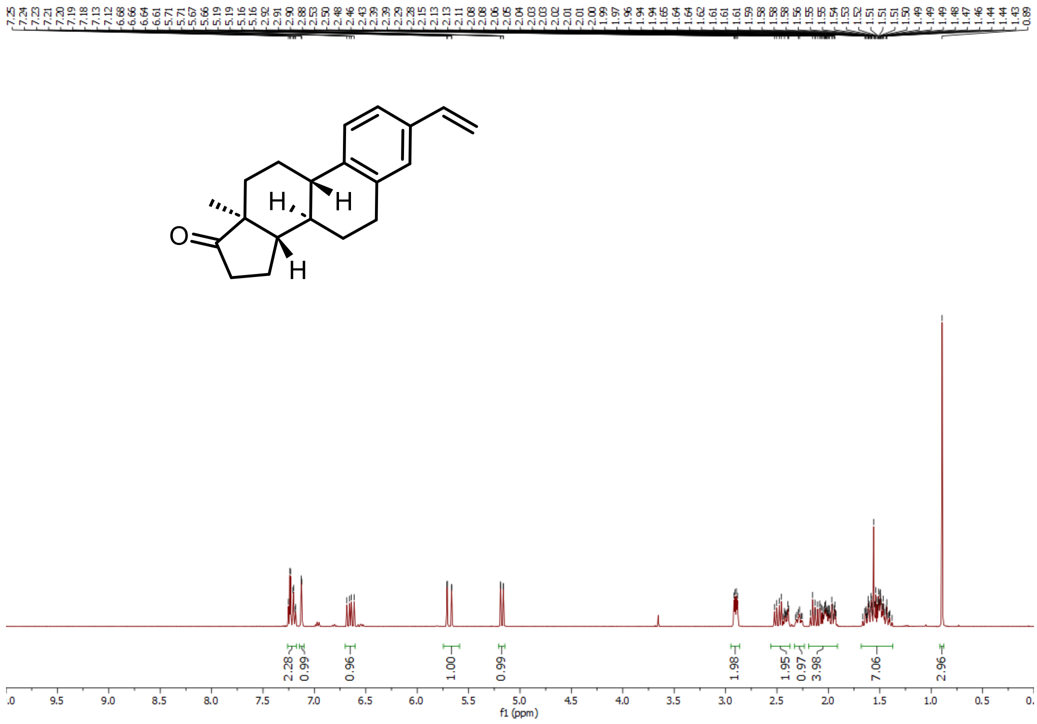

(*R*)-2,8-Dimethyl-2-((4*R*,8*R*)-4,8,12-trimethyltridecyl)-6-vinylchromane (5c)

^{1}H NMR (400 MHz, Chloroform-*d*):

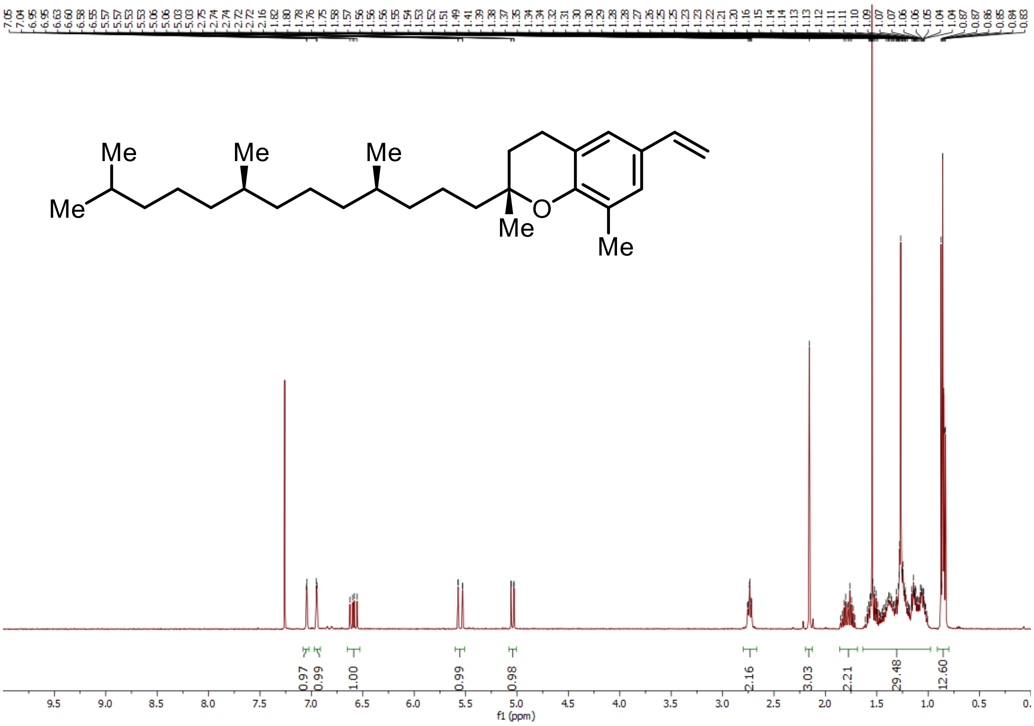

(*S*)-4,4,4-Trifluoro-1,2-diphenylbutan-1-one (3a)

^{1}H NMR (400 MHz, Chloroform-*d*):

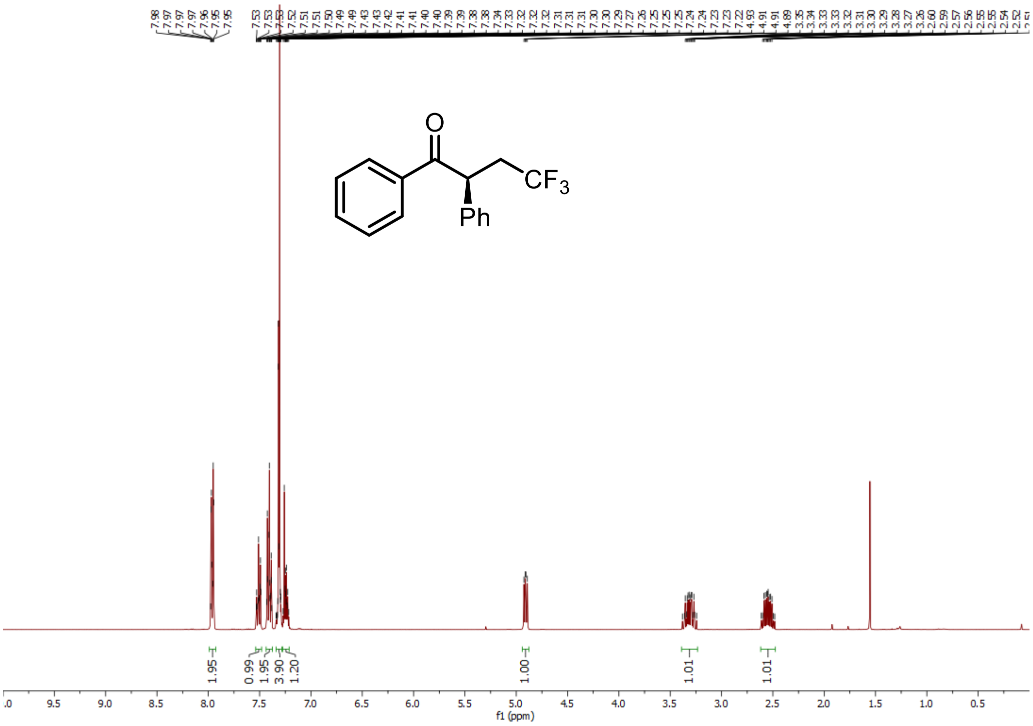

^{13}C NMR (101 MHz, Chloroform-*d*):

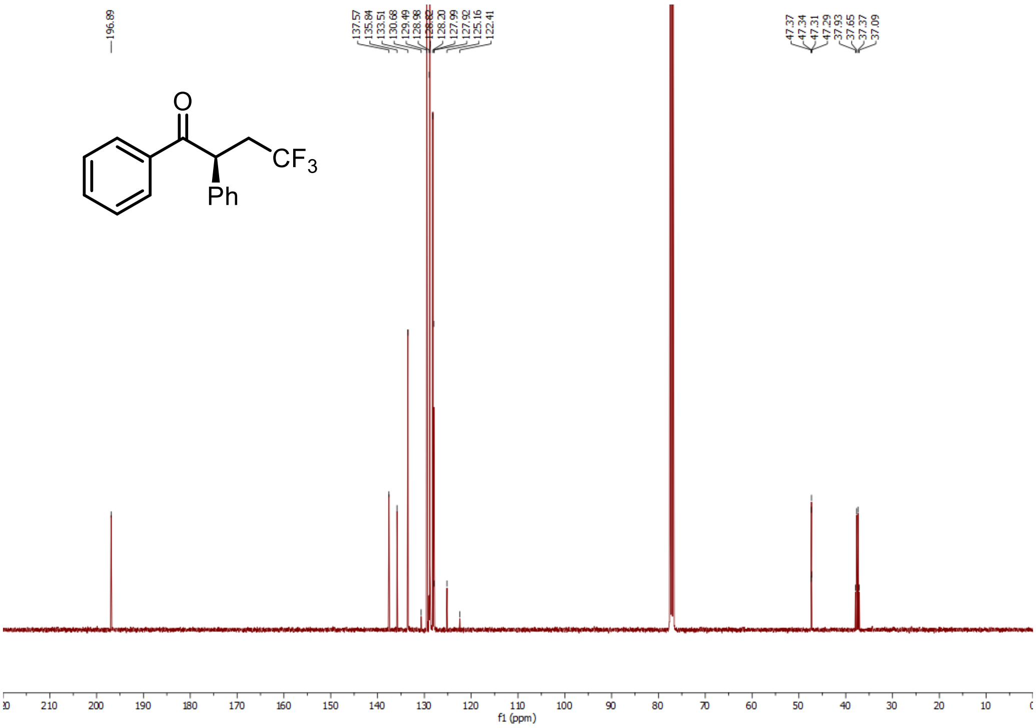

^{19}F NMR (376 MHz, Chloroform-*d*):

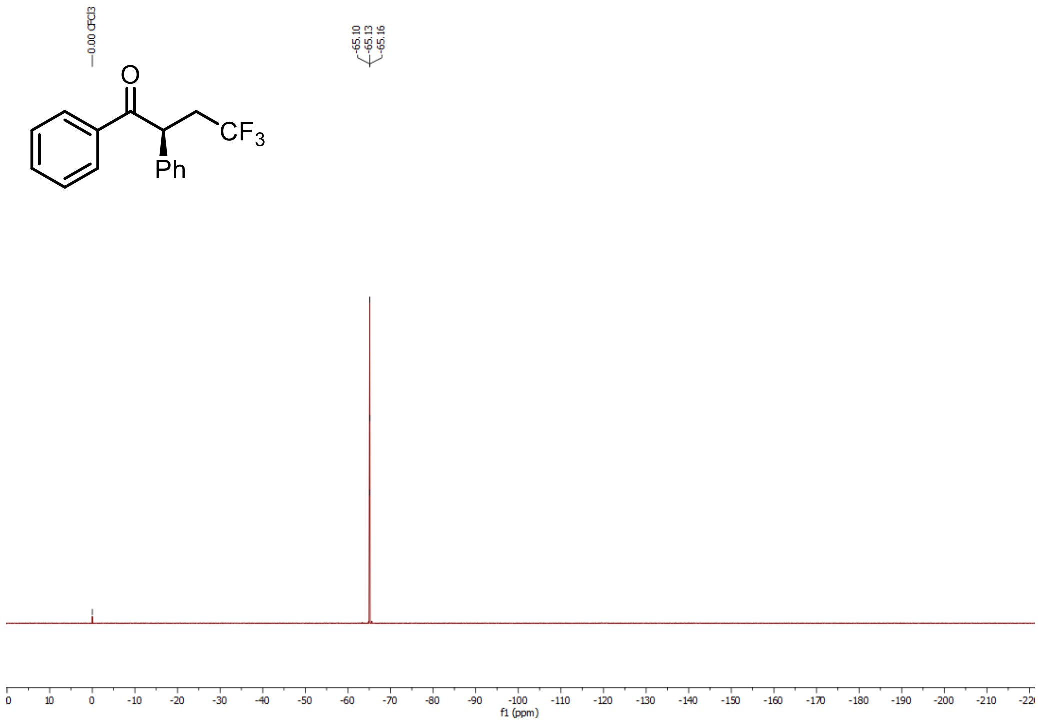

(*S*)-4,4,4-Trifluoro-1-(4-methoxyphenyl)-2-phenylbutan-1-one (3b)

^{1}H NMR (400 MHz, Chloroform-*d*):

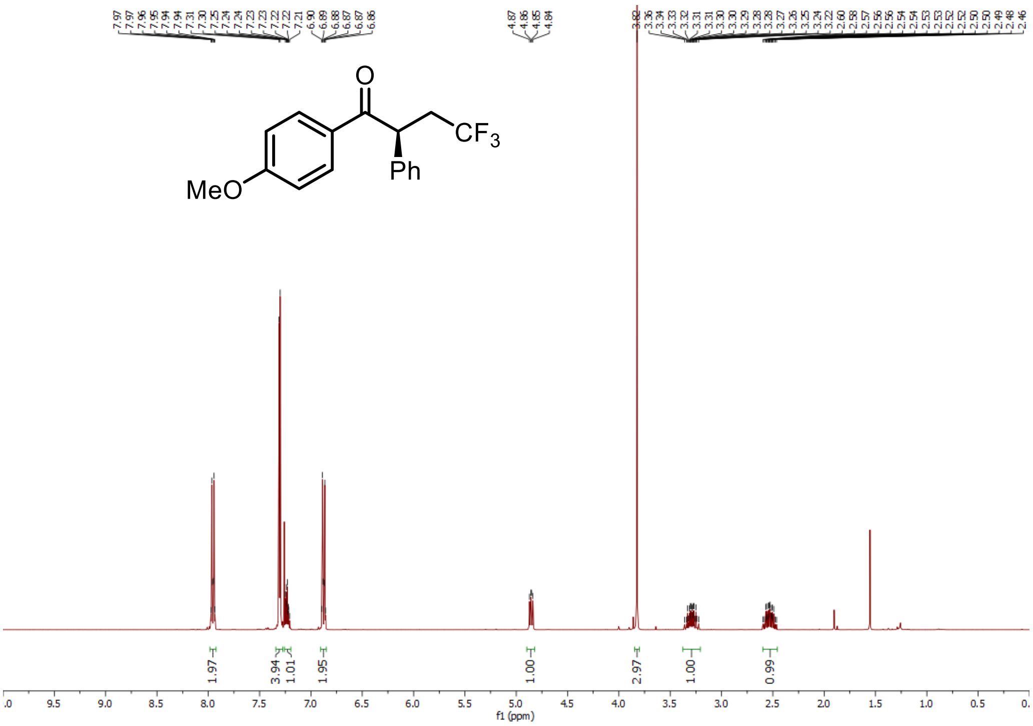

^{13}C NMR (101 MHz, Chloroform-*d*):

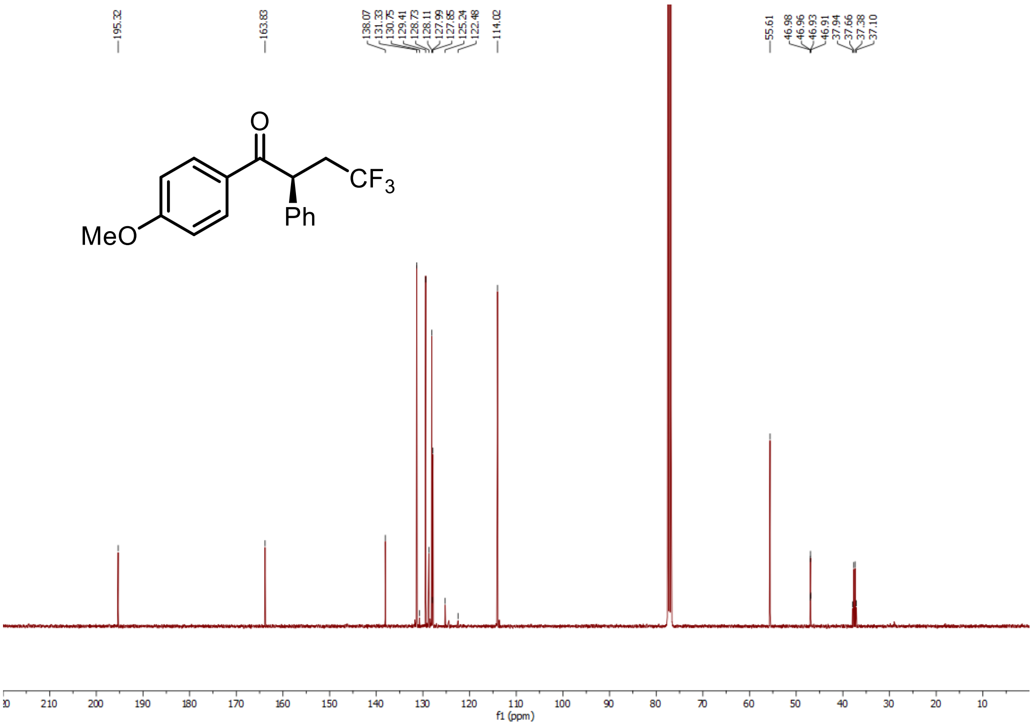

^{19}F NMR (376 MHz, Chloroform-*d*):

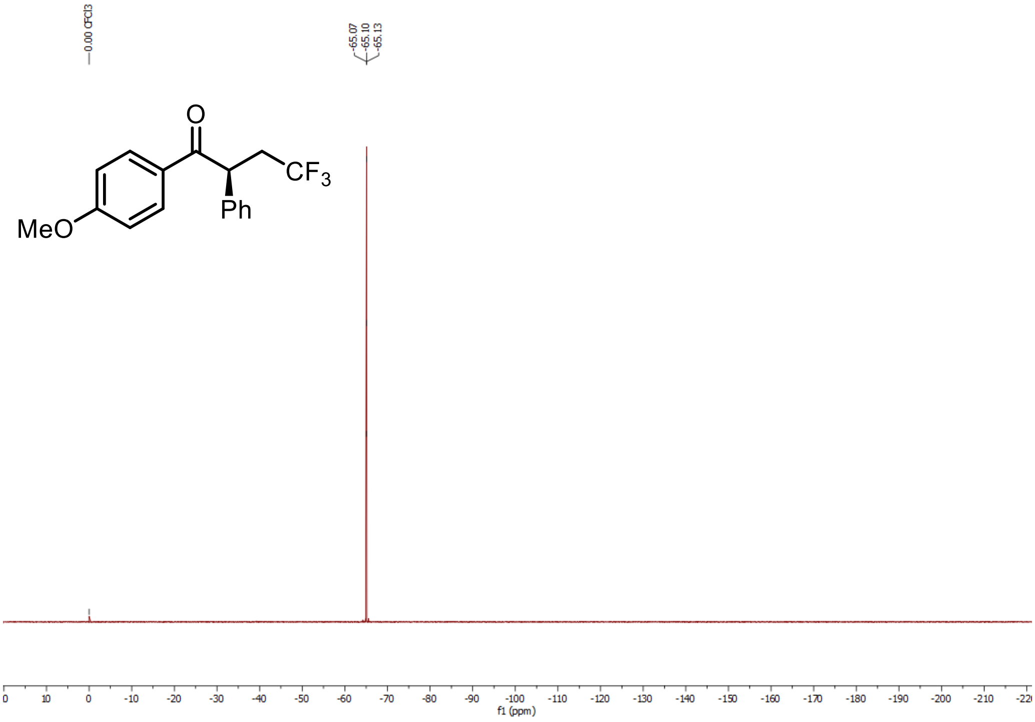

(*S*)-4,4,4-Trifluoro-1-(4-nitrophenyl)-2-phenylbutan-1-one (3c)

^{1}H NMR (400 MHz, Chloroform-*d*):

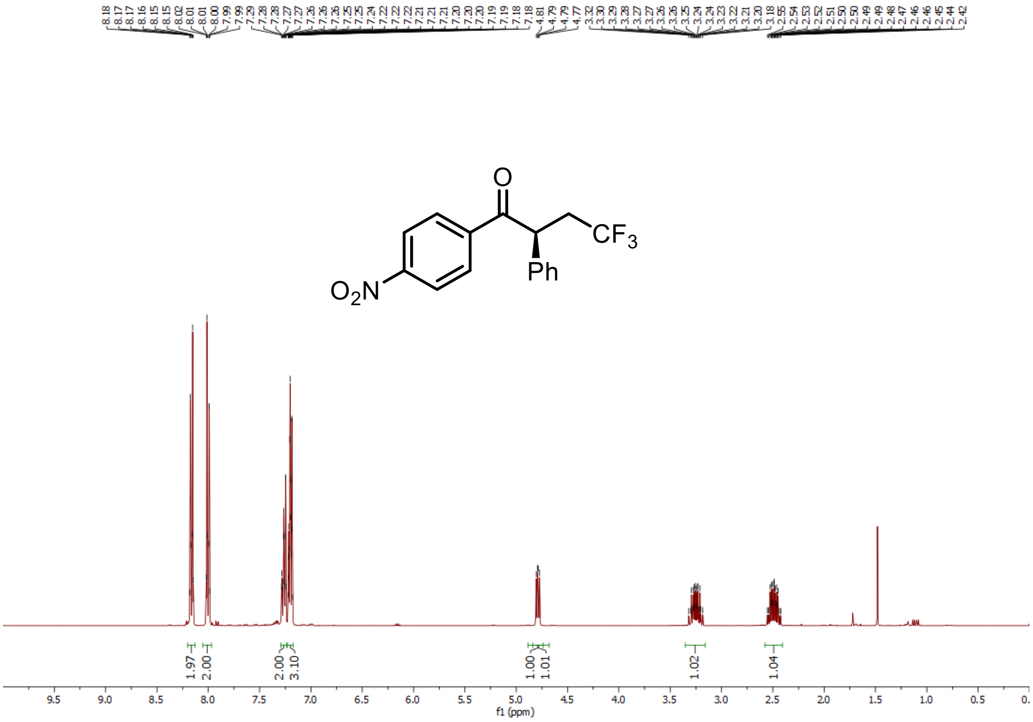

^{13}C NMR (101 MHz, Chloroform-*d*):

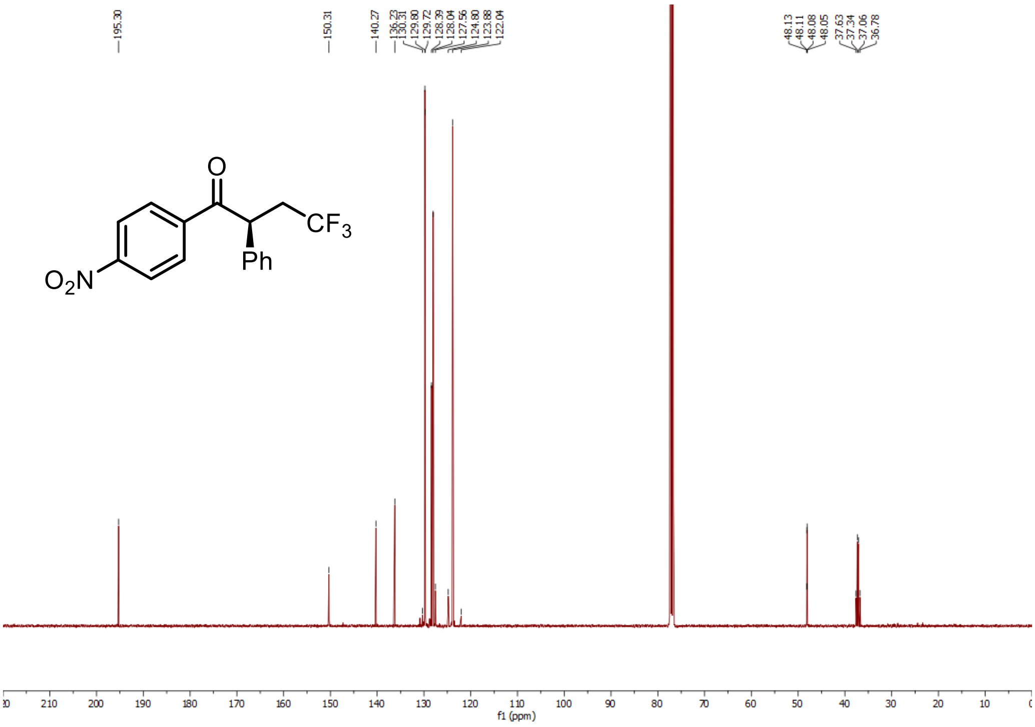

^{19}F NMR (376 MHz, Chloroform-*d*):

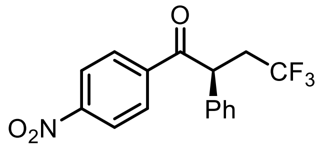

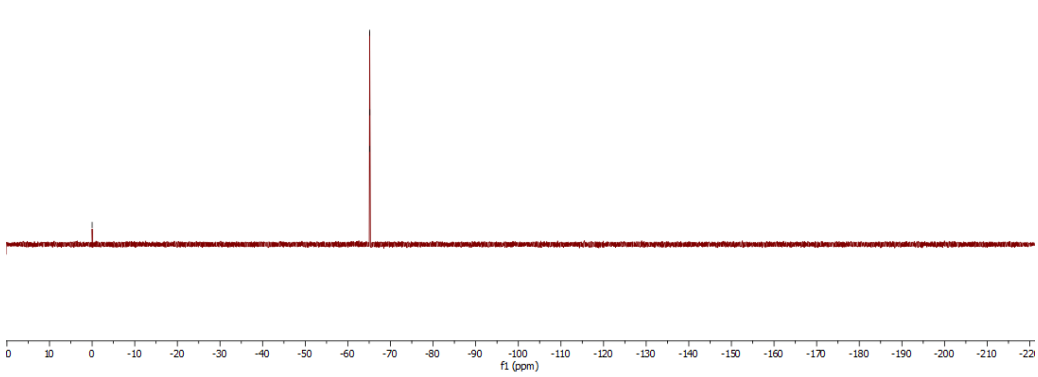

Methyl (*S*)-4-(4,4,4-trifluoro-2-phenylbutanoyl)benzoate (3d)

^{1}H NMR (400 MHz, Chloroform-*d*):

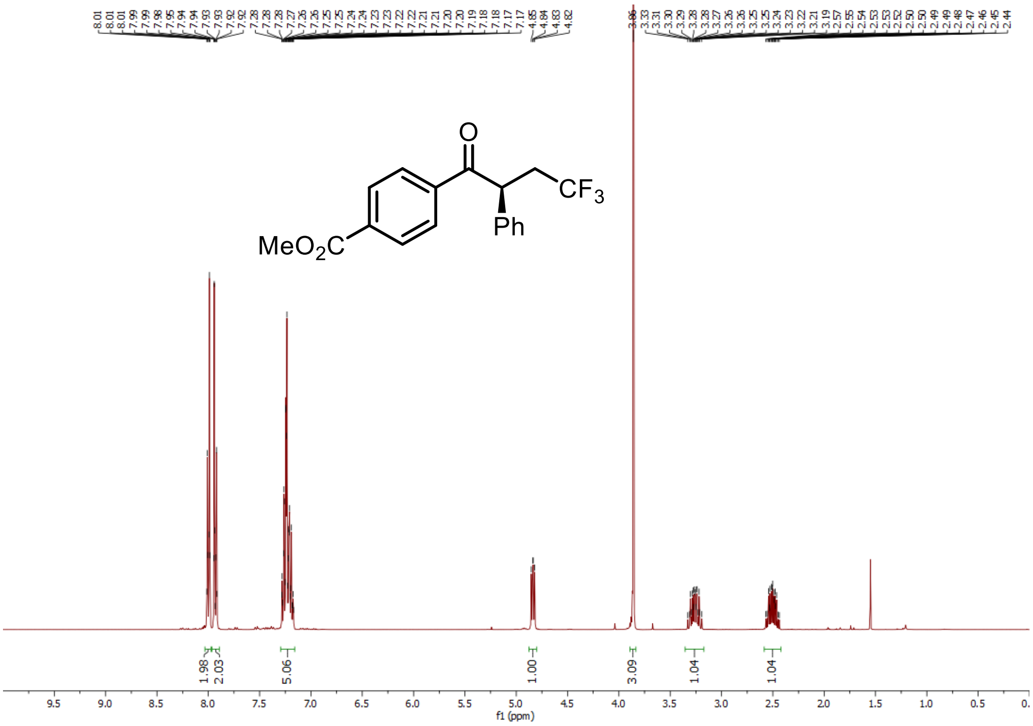

^{13}C NMR (101 MHz, Chloroform-*d*):

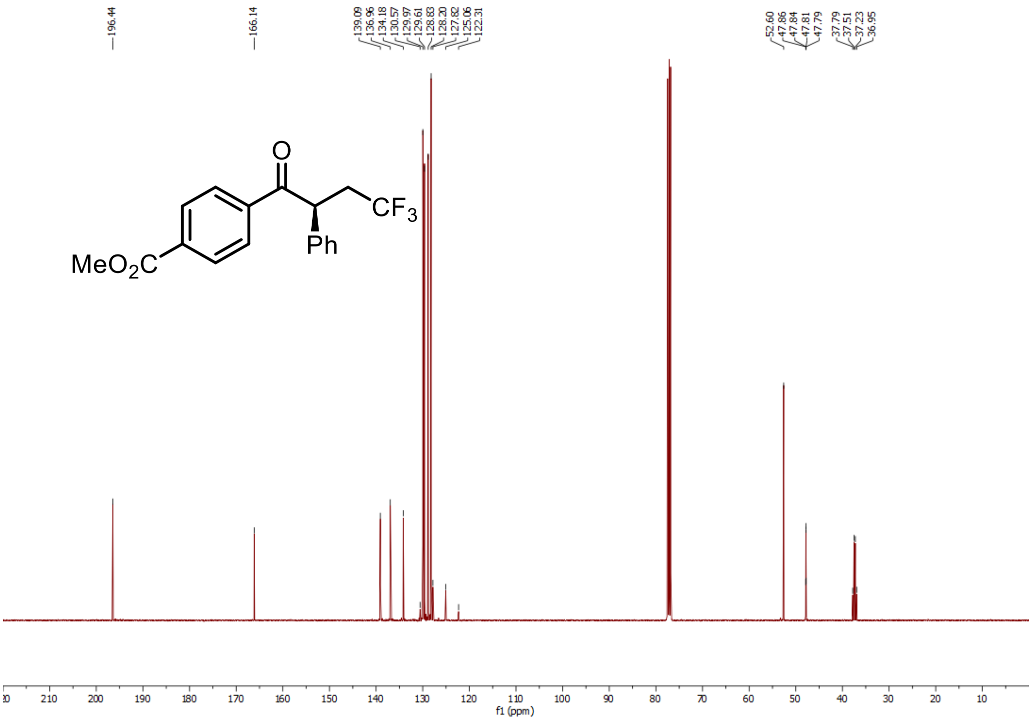

^{19}F NMR (376 MHz, Chloroform-*d*):

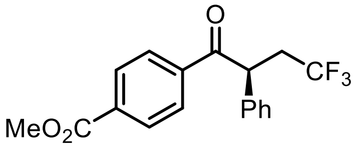

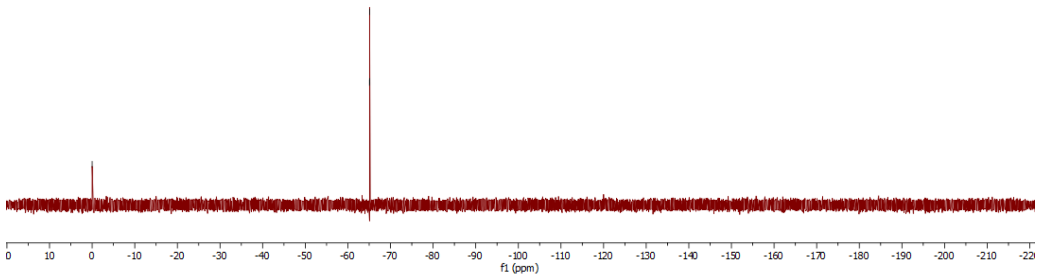

(*S*)-4,4,4-Trifluoro-1-(3-methoxyphenyl)-2-phenylbutan-1-one (3e)

^{1}H NMR (400 MHz, Chloroform-*d*):

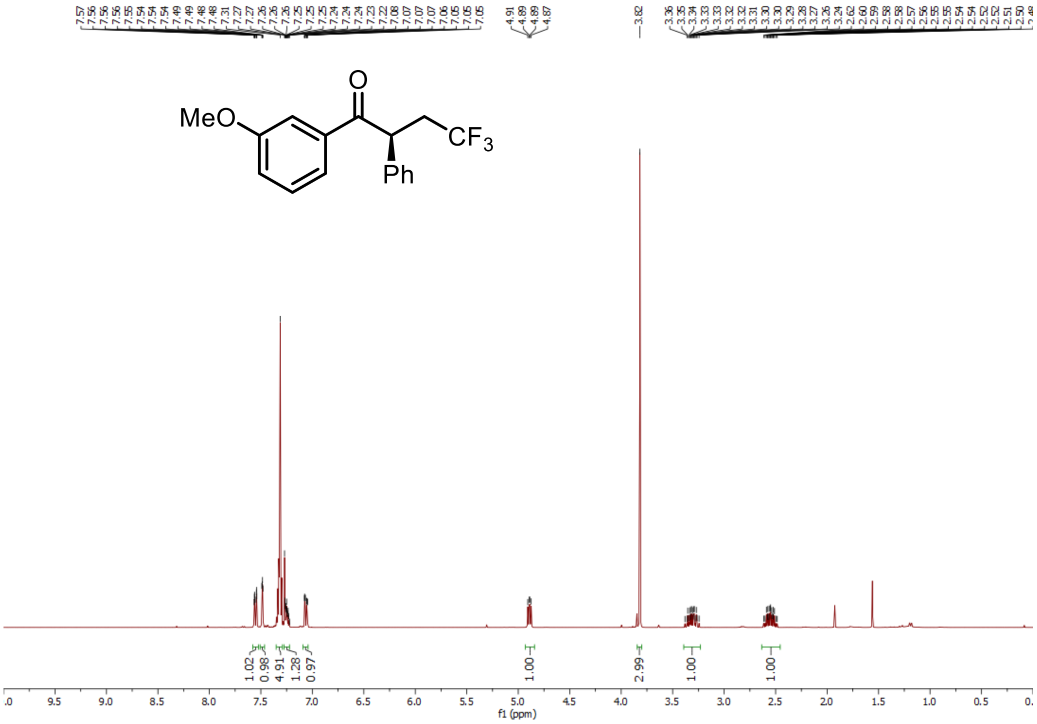

^{13}C NMR (101 MHz, Chloroform-*d*):

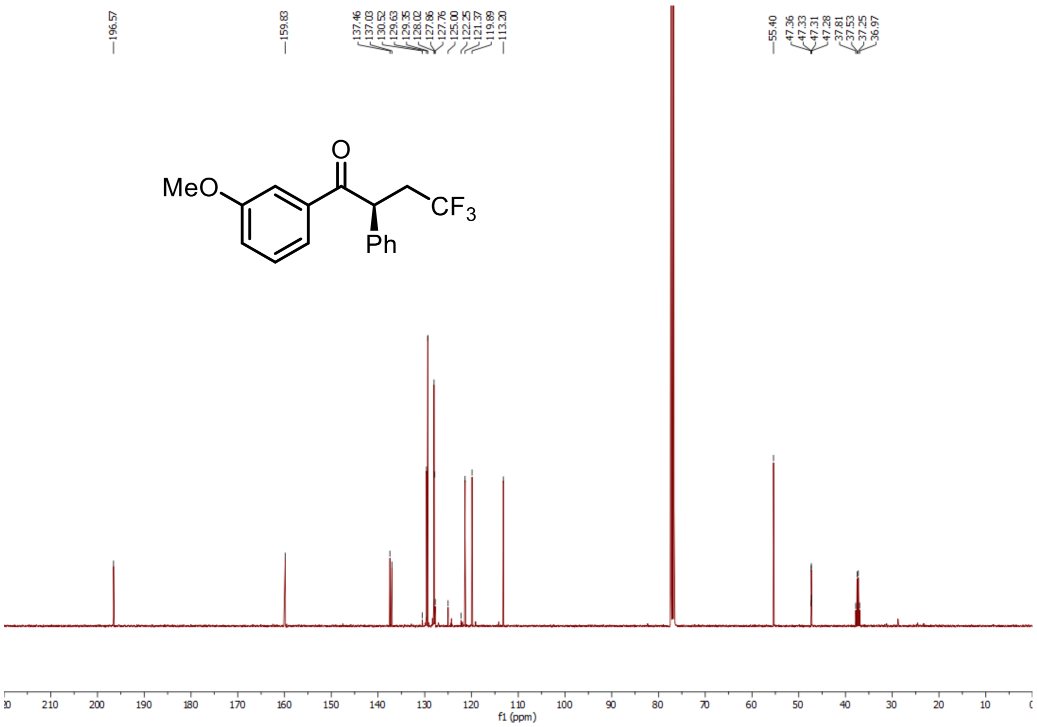

^{19}F NMR (376 MHz, Chloroform-*d*):

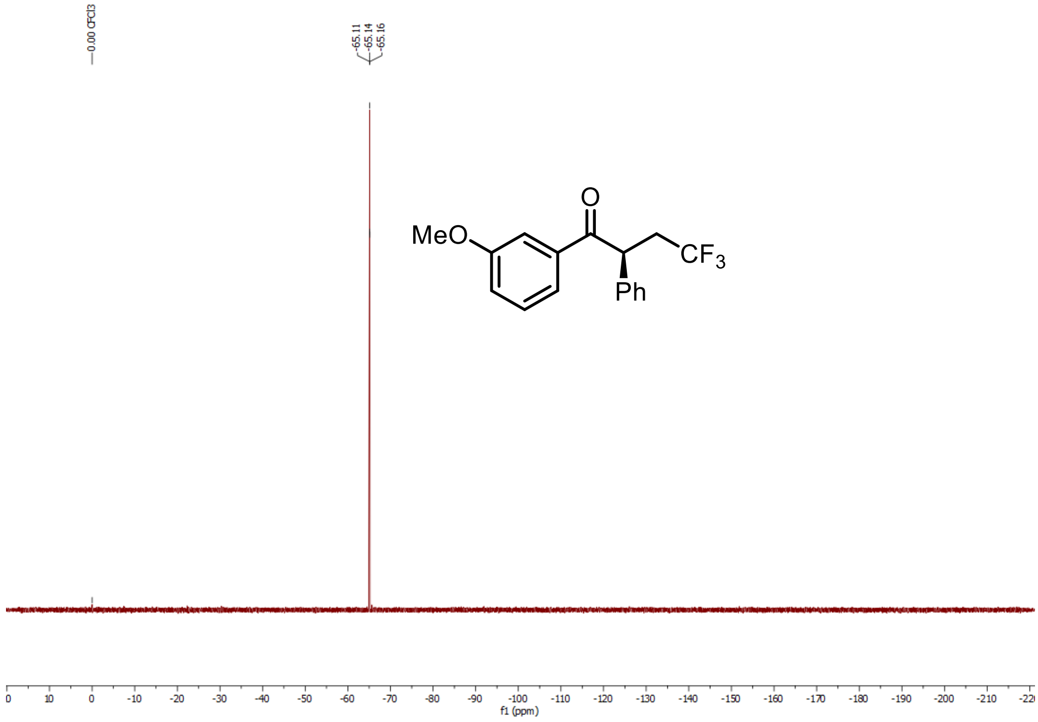

(*S*)-4,4,4-Trifluoro-1-(2-fluorophenyl)-2-phenylbutan-1-one (3f)

^{1}H NMR (400 MHz, Chloroform-*d*):

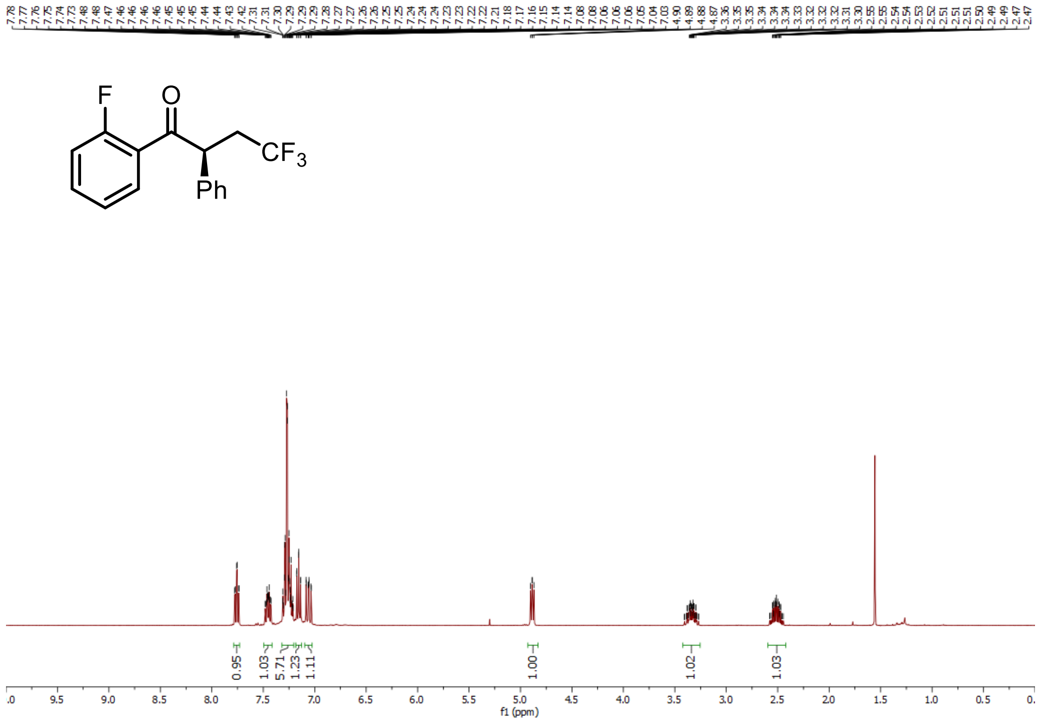

^{13}C NMR (101 MHz, Chloroform-*d*)

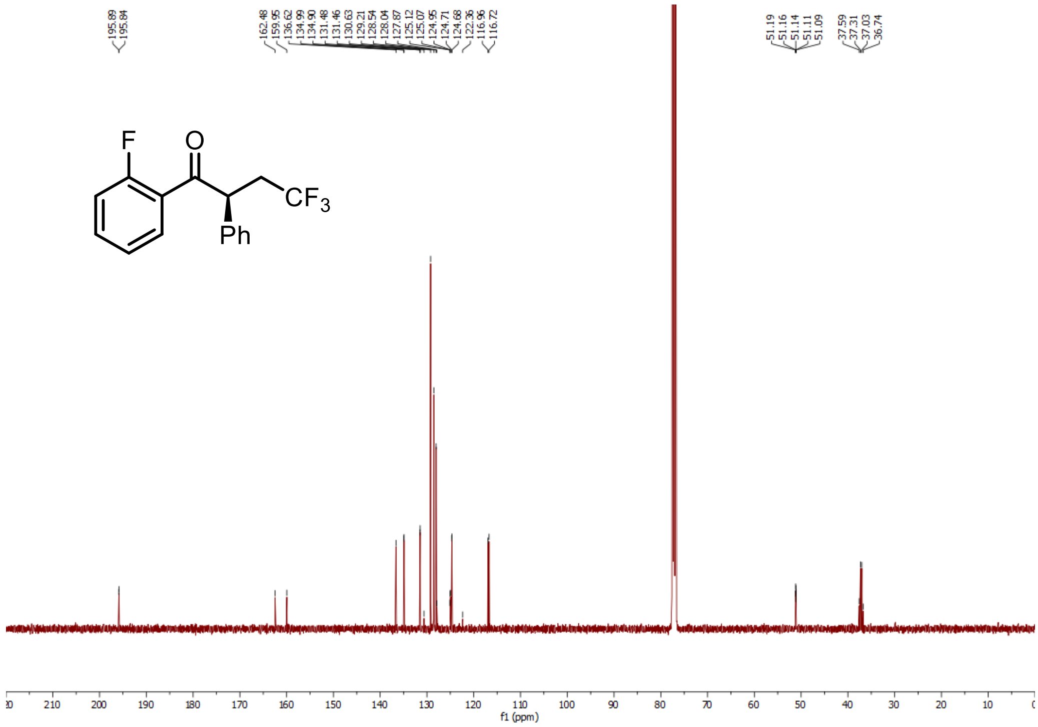

^{19}F NMR (376 MHz, Chloroform-*d*):

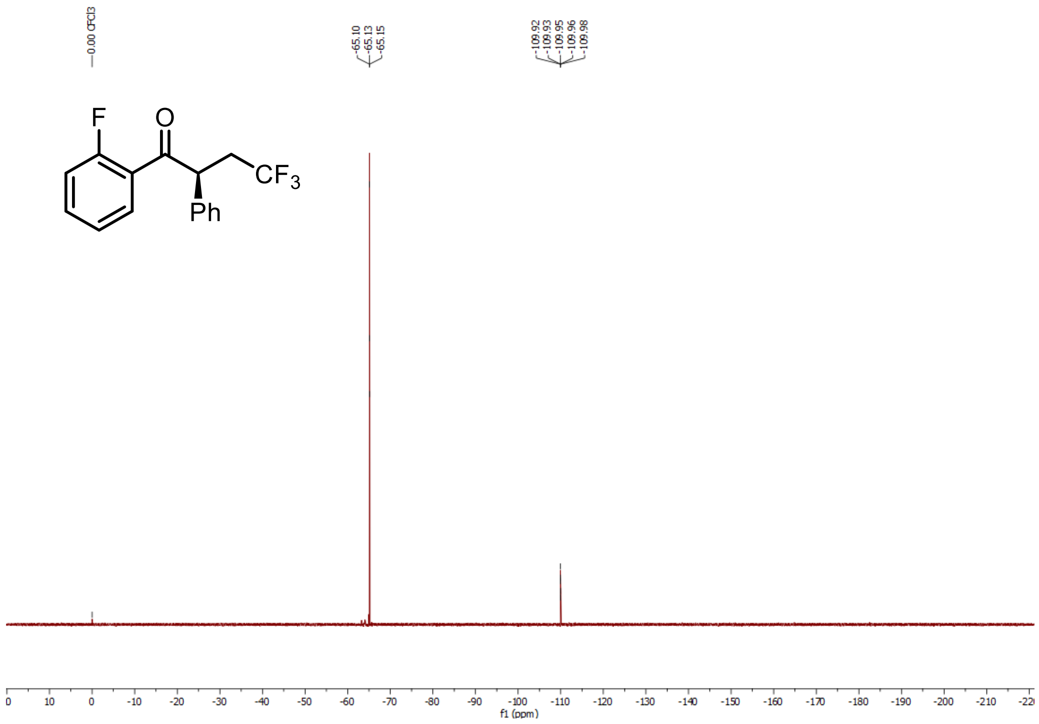

(*S*)-4,4,4-Trifluoro-1-(2-hydroxyphenyl)-2-phenylbutan-1-one (3g)

^{1}H NMR (400 MHz, Chloroform-*d*):

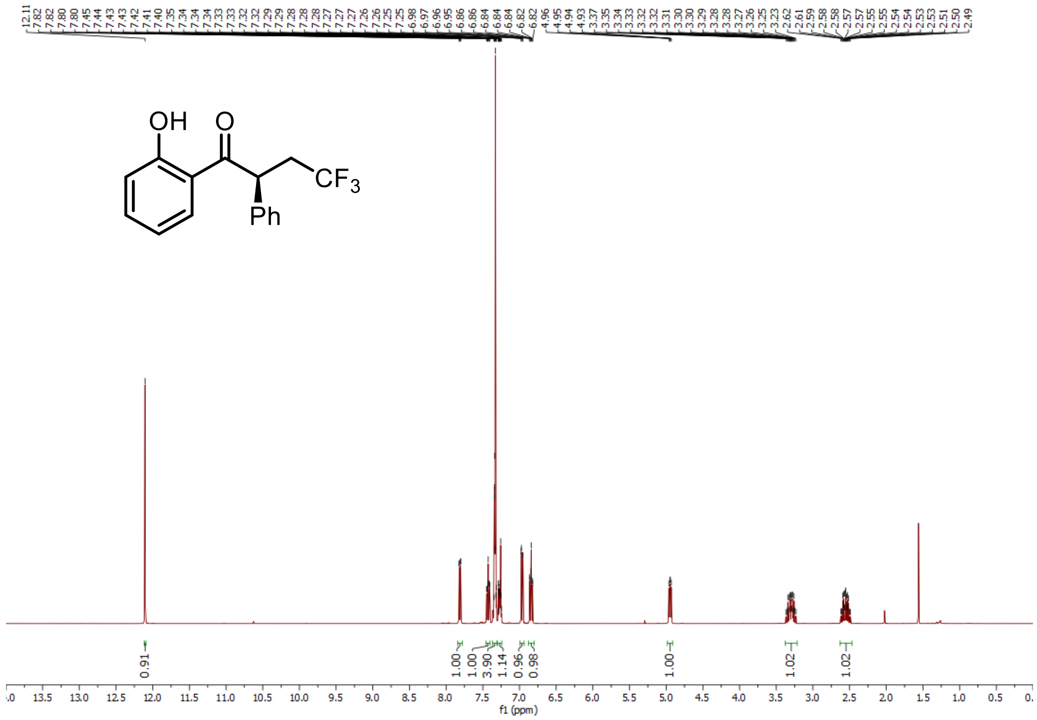

^{13}C NMR (101 MHz, Chloroform-*d*):

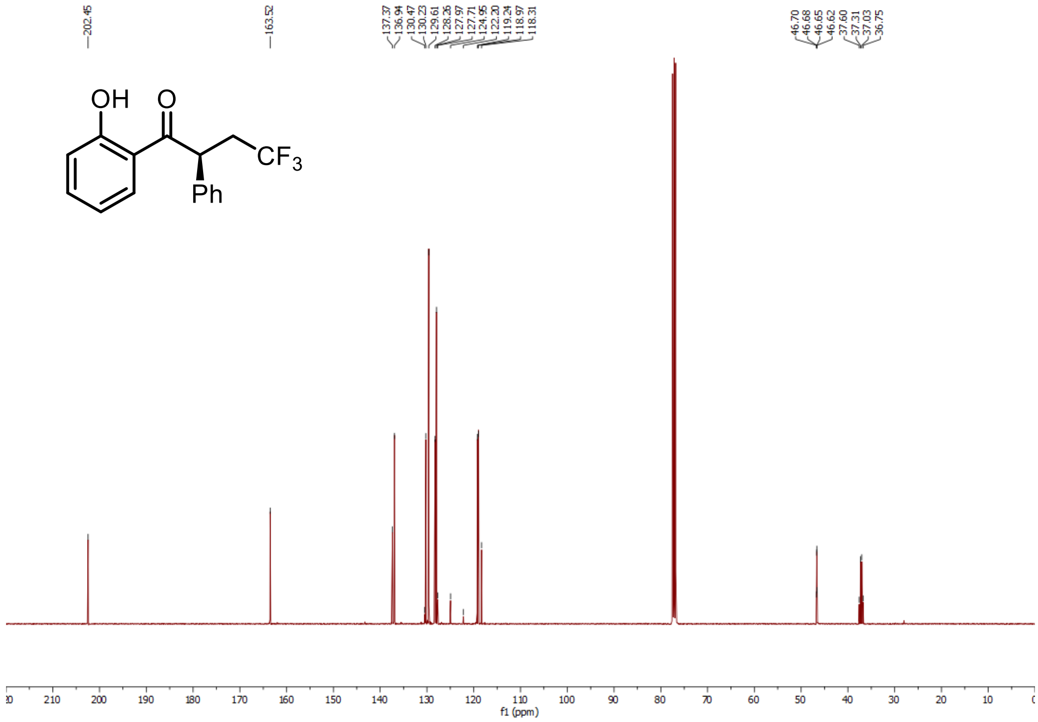

¹⁹F NMR (376 MHz, Chloroform-*d*):

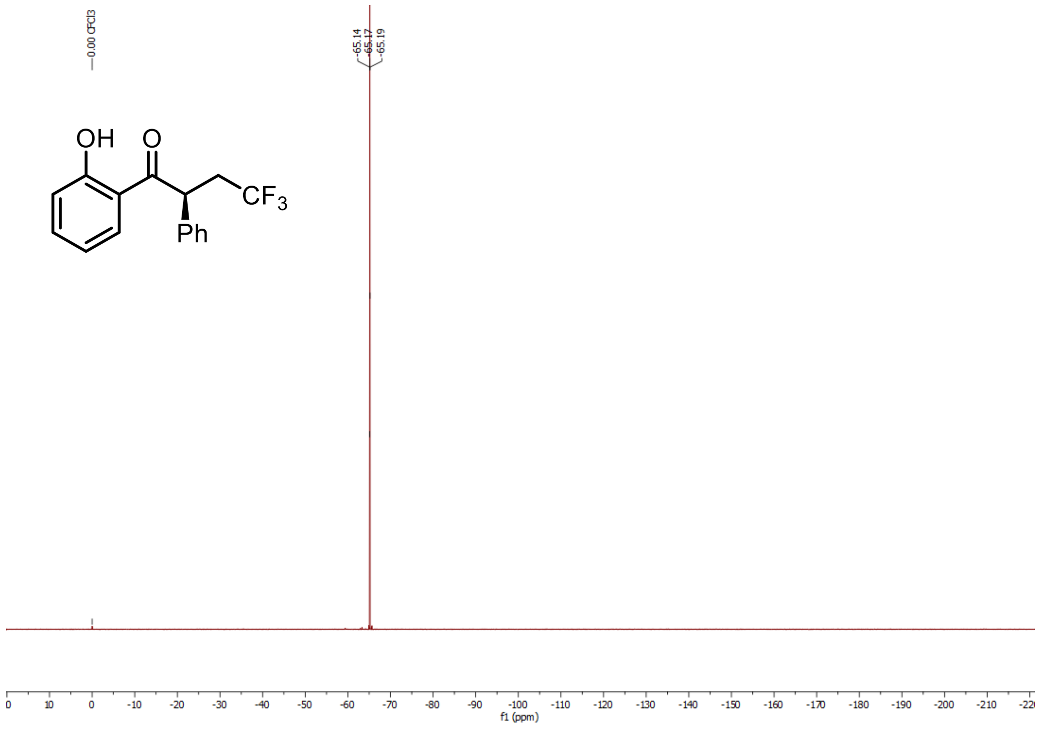

(*S*)-4,4,4-Trifluoro-1-(naphthalen-2-yl)-2-phenylbutan-1-one (3h)

^{1}H NMR (400 MHz, Chloroform-*d*):

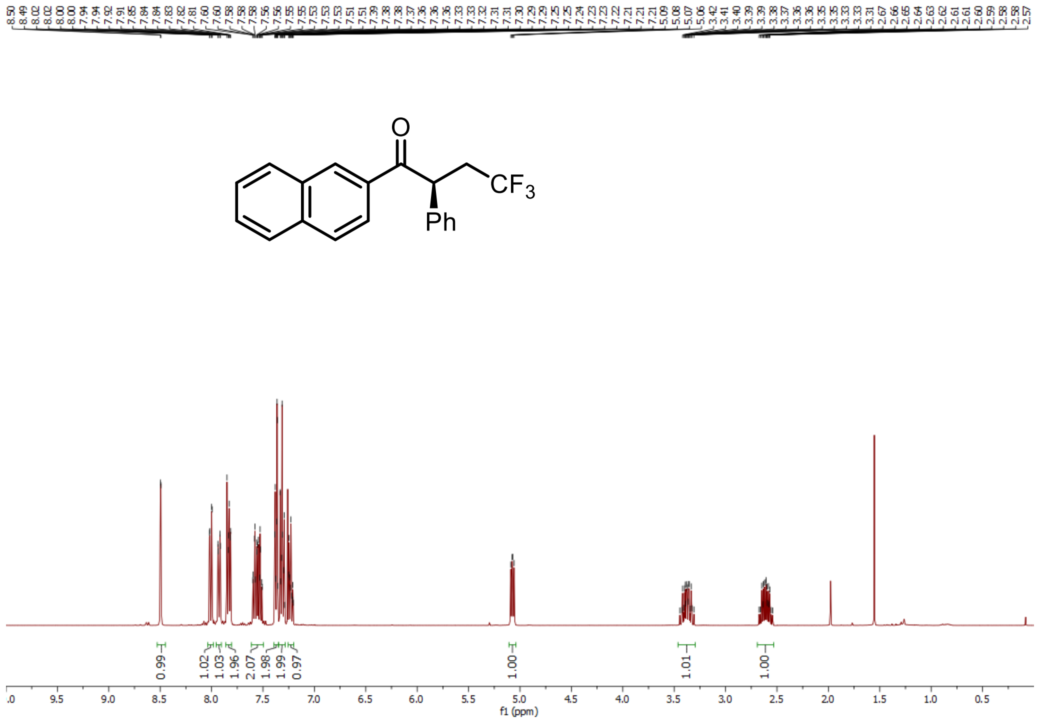

^{13}C NMR (101 MHz, Chloroform-*d*):

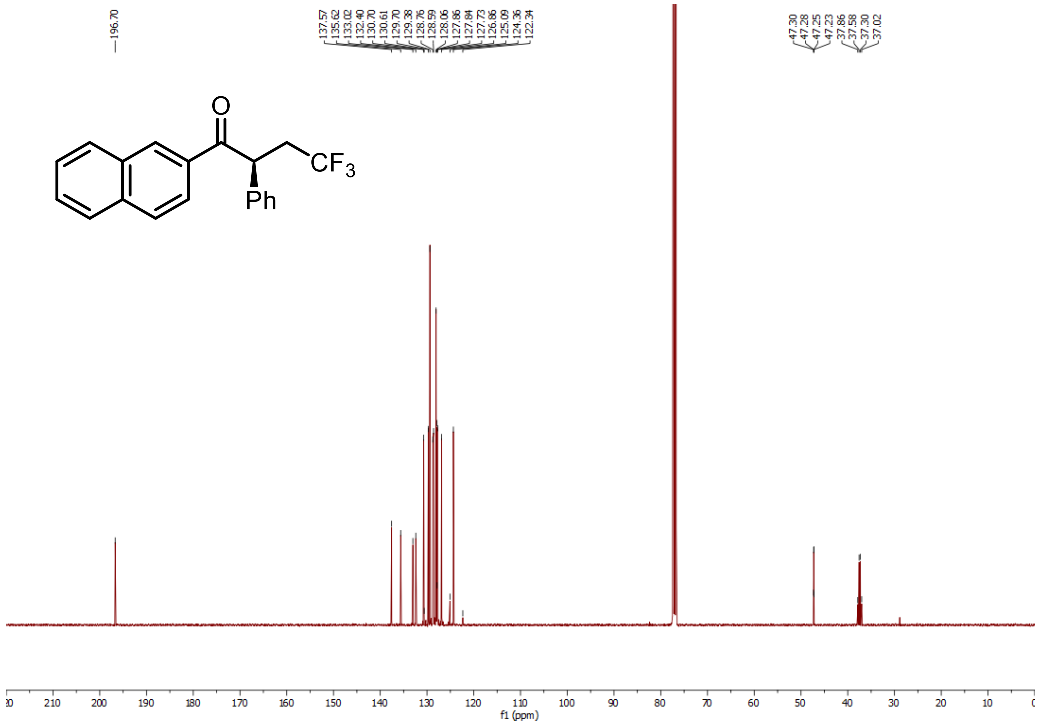

^{19}F NMR (376 MHz, Chloroform-*d*):

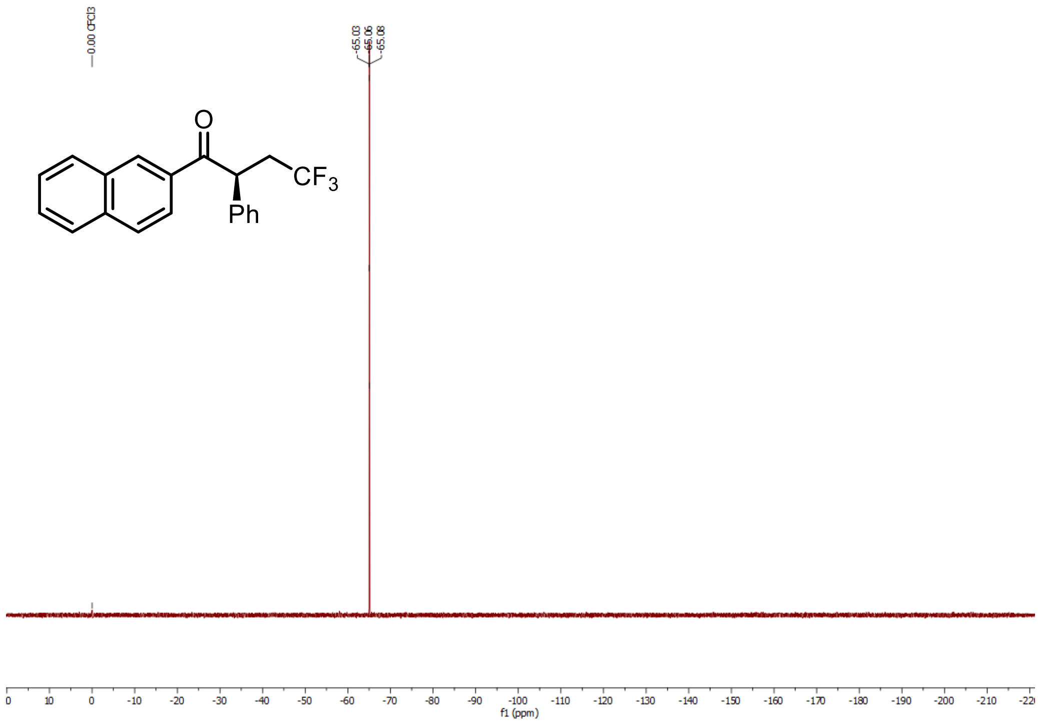

(*S*)-4,4,4-Trifluoro-2-phenyl-1-(pyridin-3-yl)butan-1-one (3i)

[1]**H NMR** (400 MHz, Chloroform-*d*):

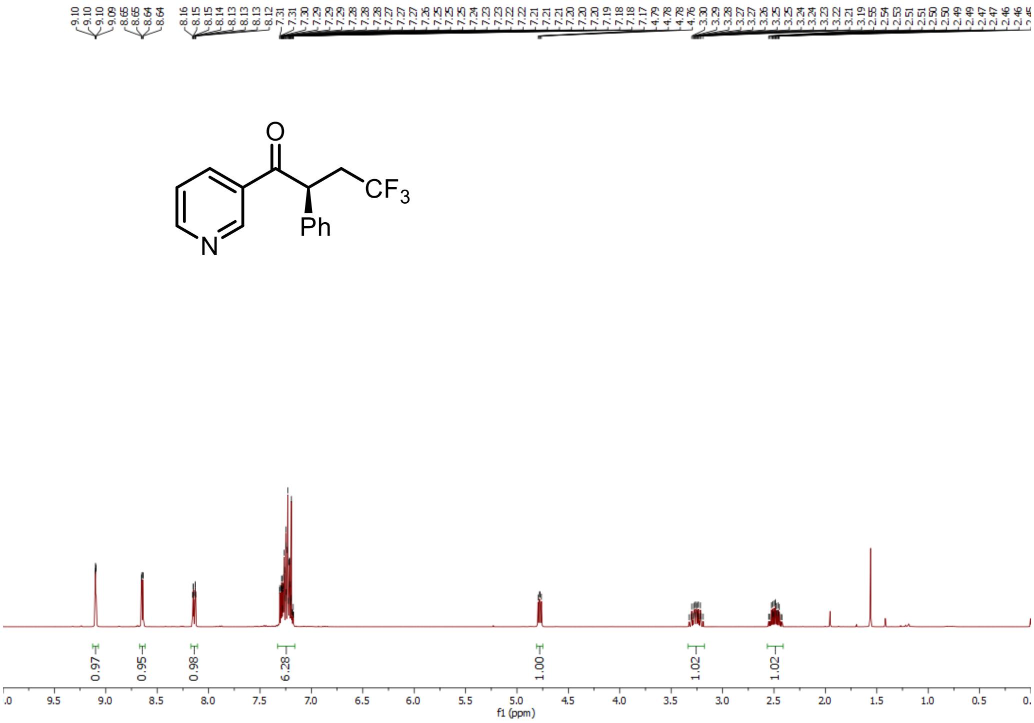

[13]**C NMR** (101 MHz, Chloroform-*d*):

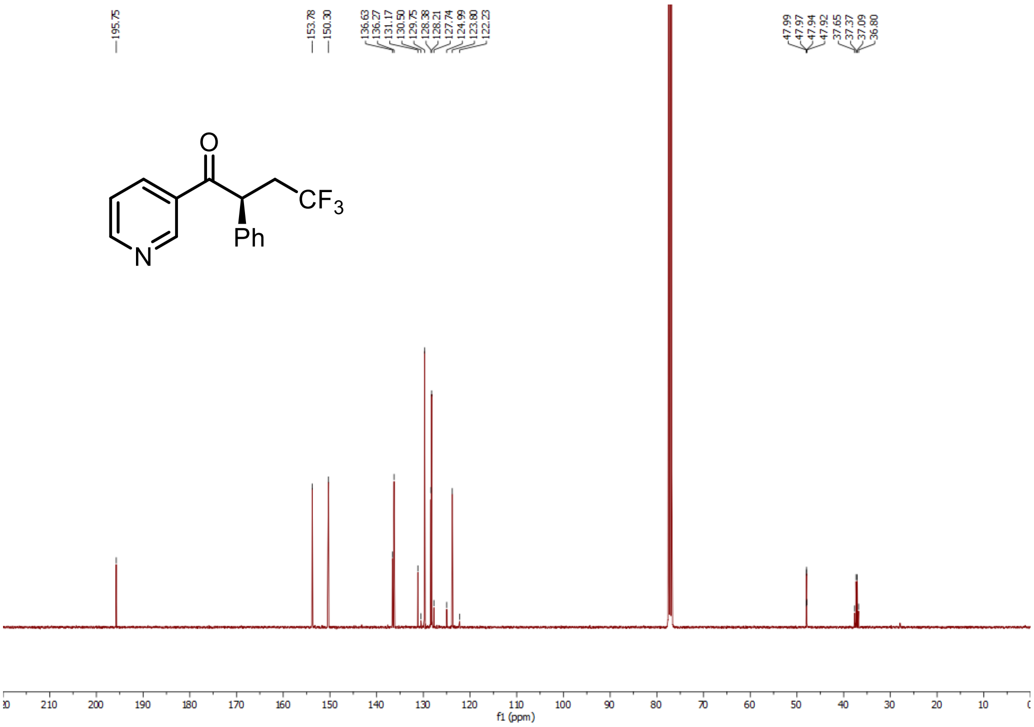

^{19}F NMR (376 MHz, Chloroform-*d*):

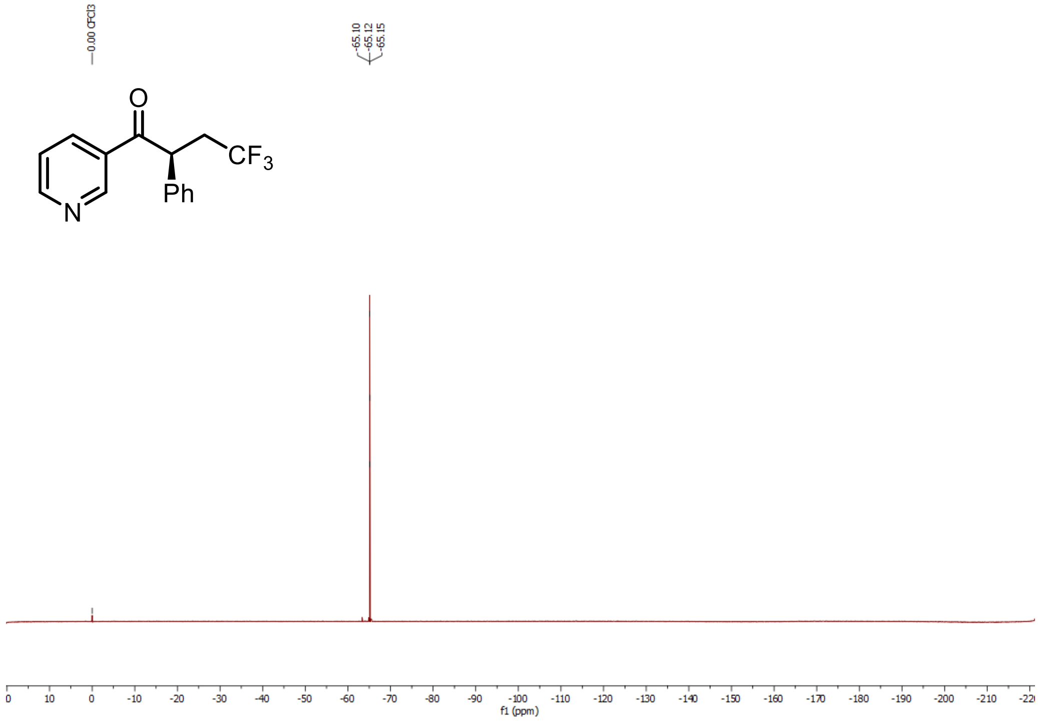

(*S*)-1-(9-Ethyl-9*H*-carbazol-3-yl)-4,4,4-trifluoro-2-phenylbutan-1-one (3j)

^{1}H NMR (400 MHz, Chloroform-*d*):

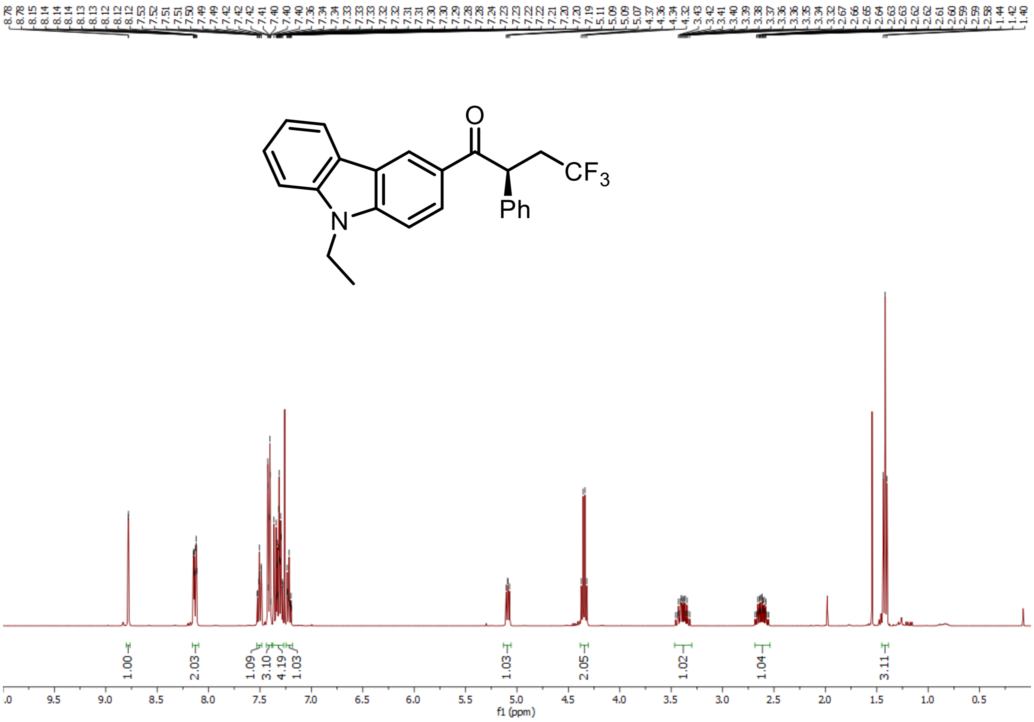

^{13}C NMR (101 MHz, Chloroform-*d*):

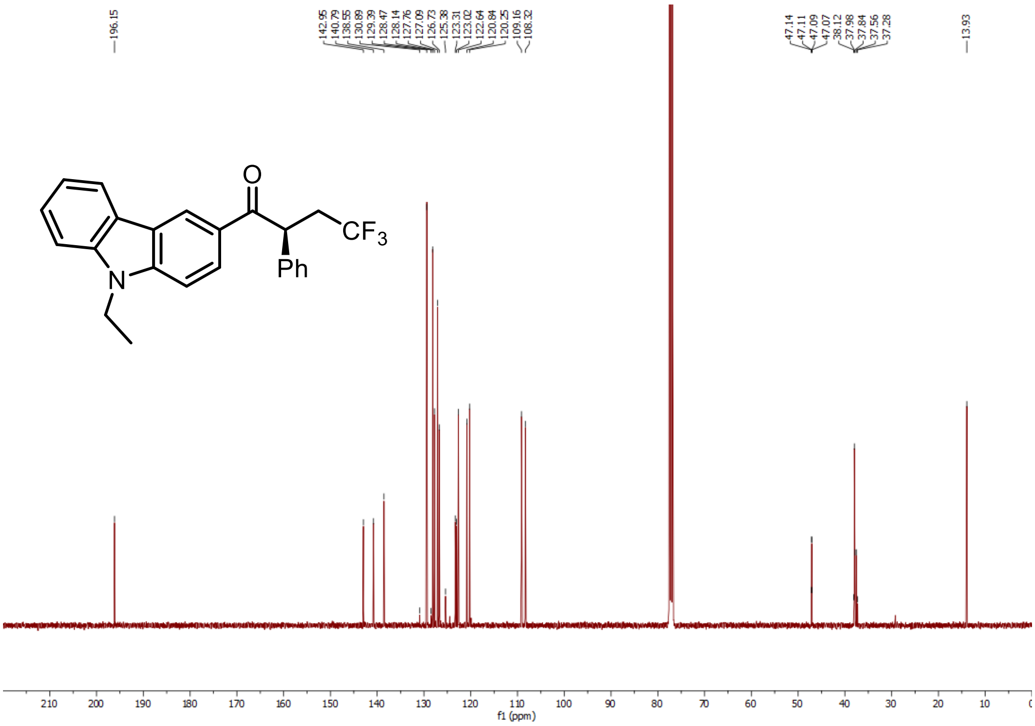

^{19}F NMR (376 MHz, Chloroform-*d*):

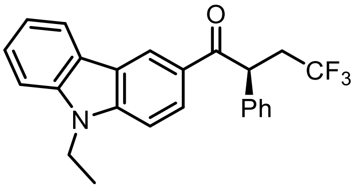

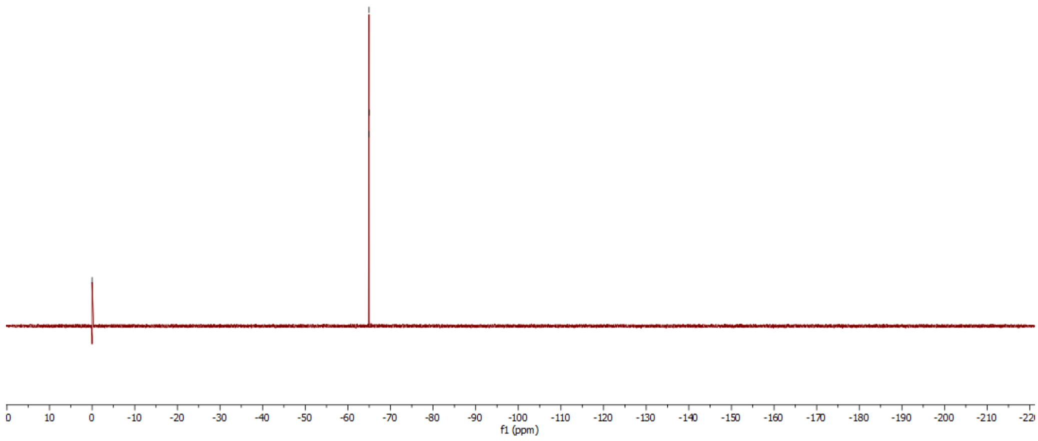

(*S*)-4,4,4-trifluoro-1-(furan-2-yl)-2-phenylbutan-1-one (3k)

^{1}H NMR (400 MHz, Chloroform-*d*):

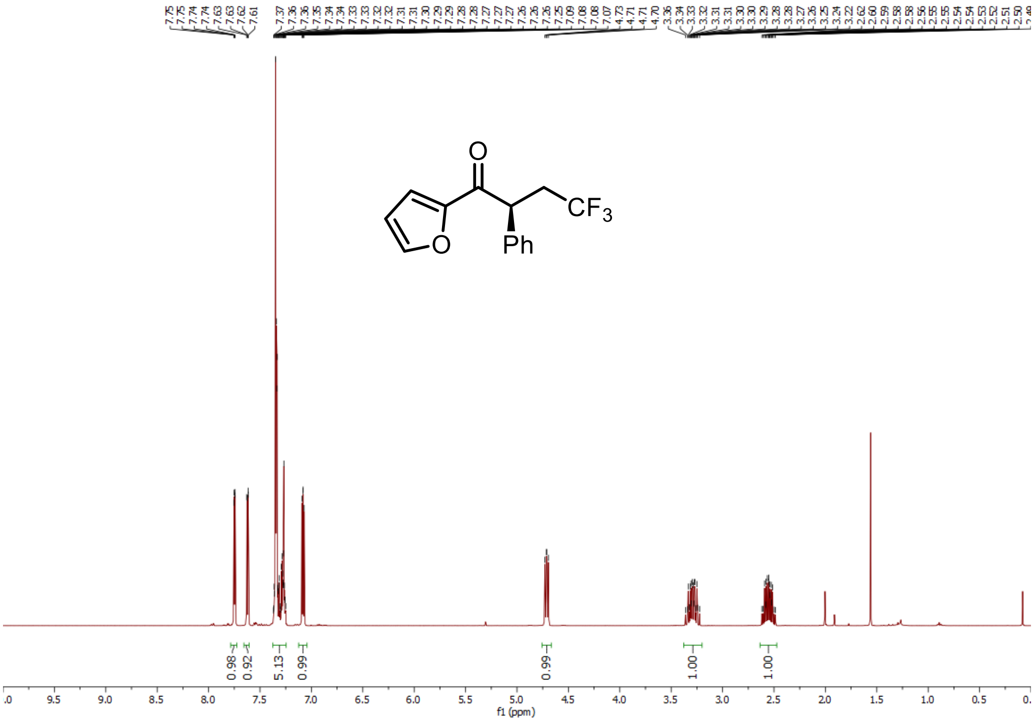

^{13}C NMR (101 MHz, Chloroform-*d*):

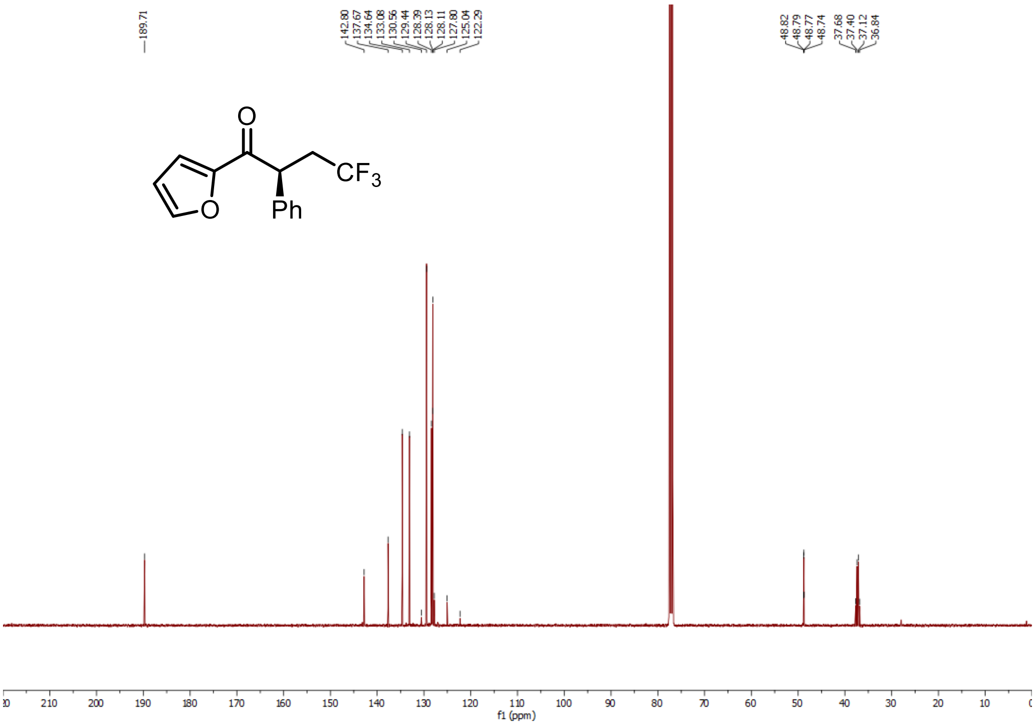

^{19}F NMR (376 MHz, Chloroform-*d*):

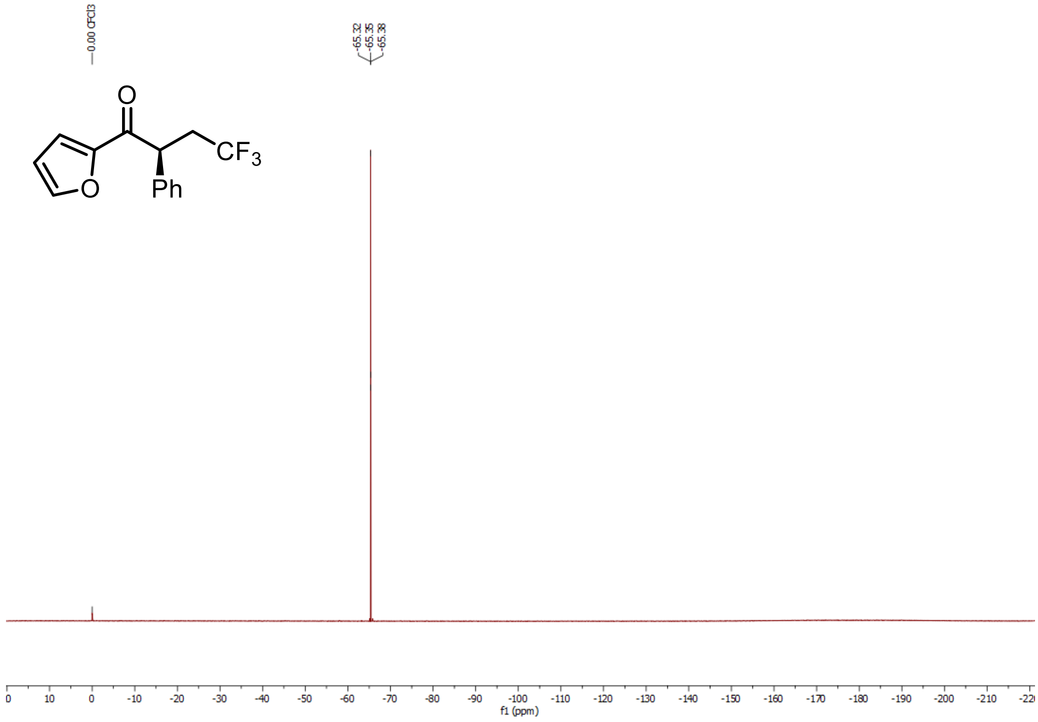

(*S*)-4,4,4-Trifluoro-2-phenyl-1-(thiophen-2-yl)butan-1-one (3l)

^{1}H NMR (400 MHz, Chloroform-*d*):

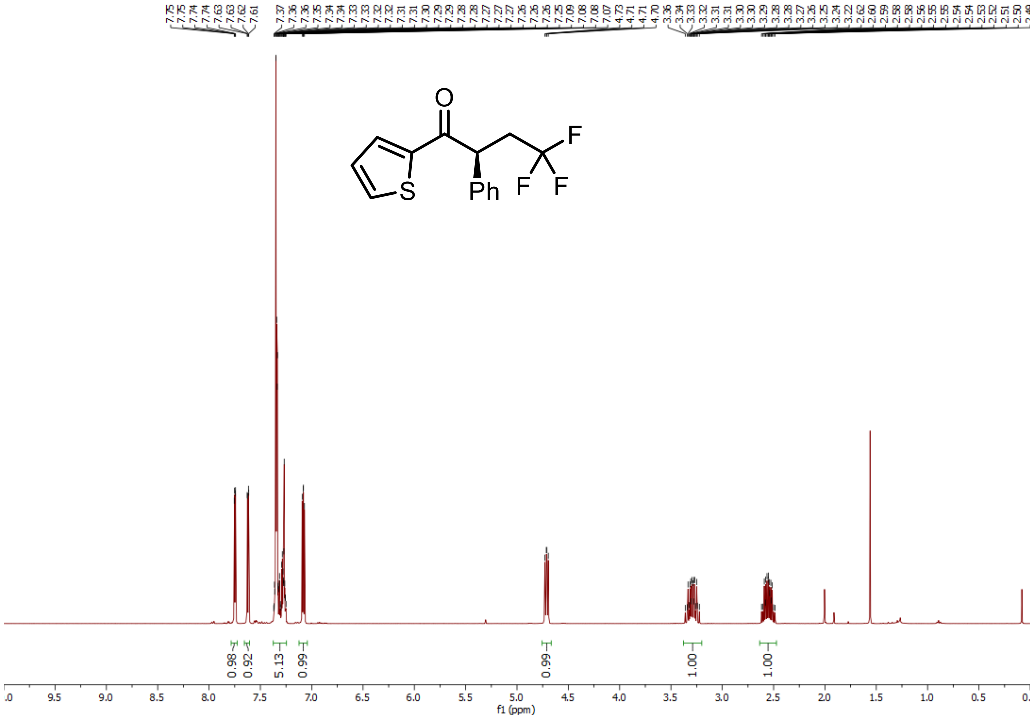

^{13}C NMR (101 MHz, Chloroform-*d*):

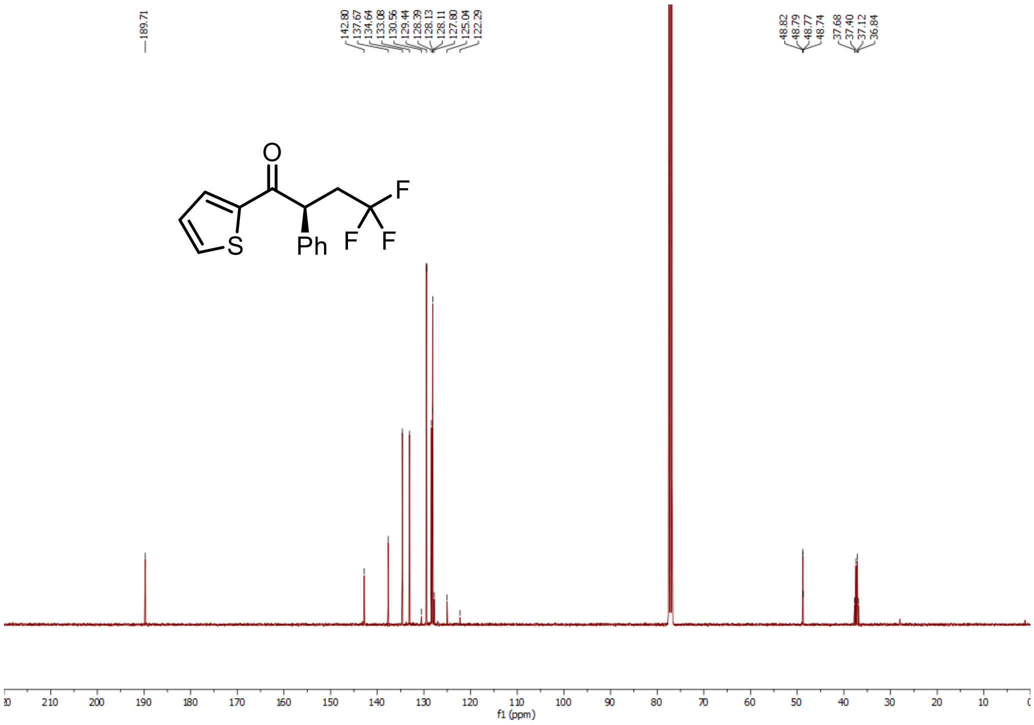

^{19}F NMR (376 MHz, Chloroform-*d*):

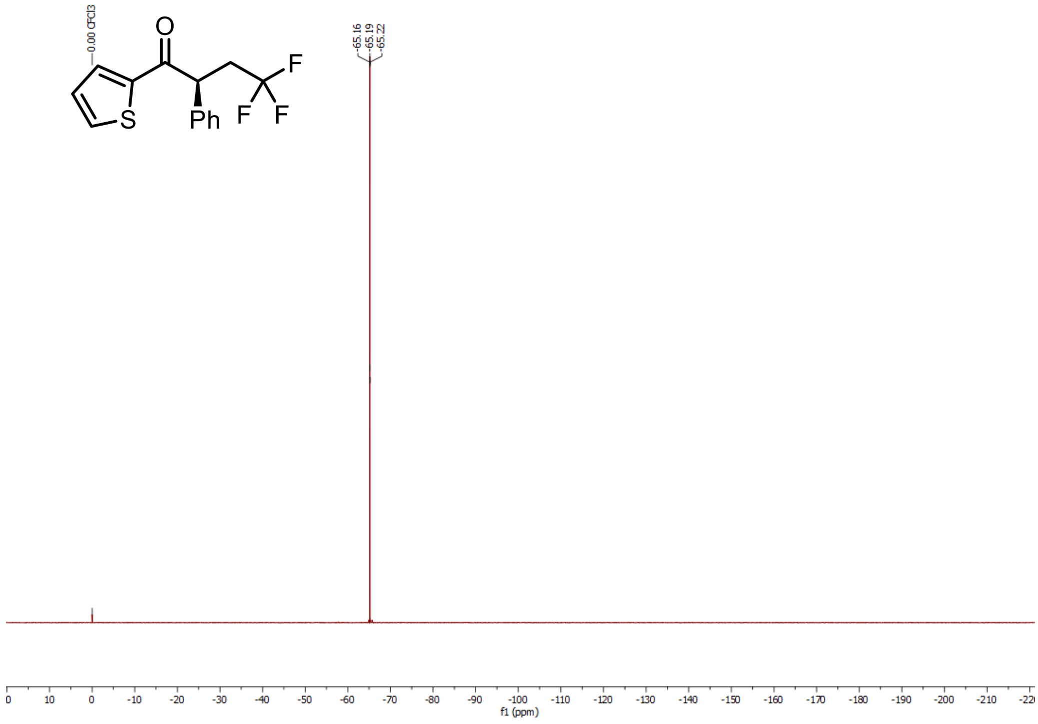

(*S*)-6,6,6-Trifluoro-4-(4-methoxyphenyl)hexan-3-one (3m)

^{1}H NMR (400 MHz, Chloroform-*d*):

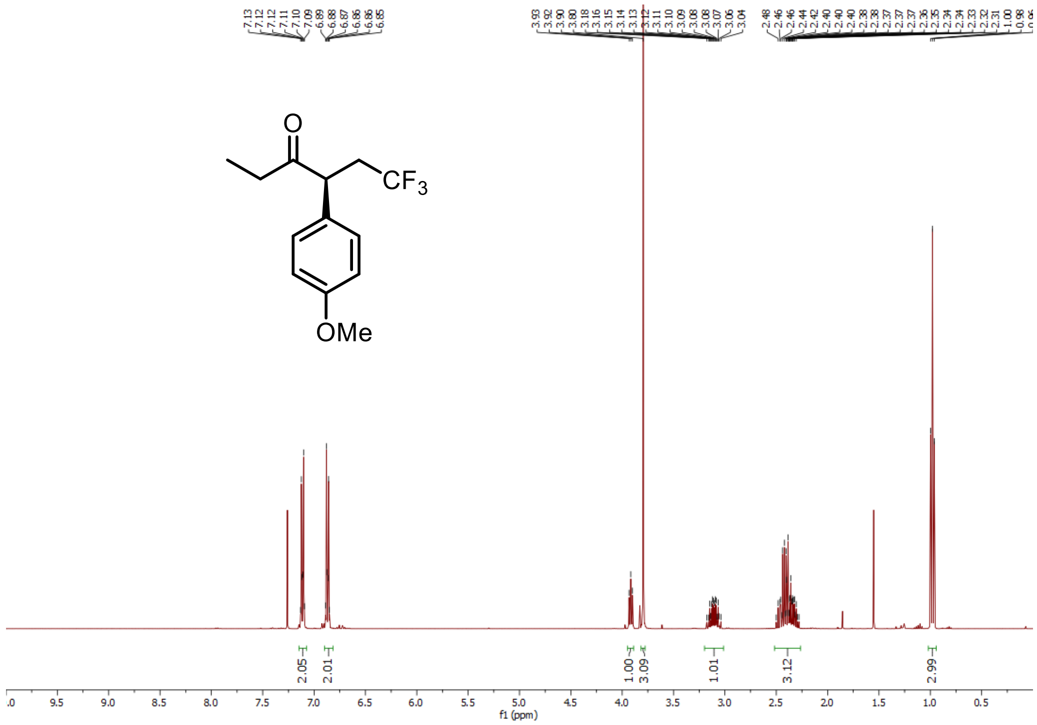

^{13}C NMR (101 MHz, Chloroform-*d*):

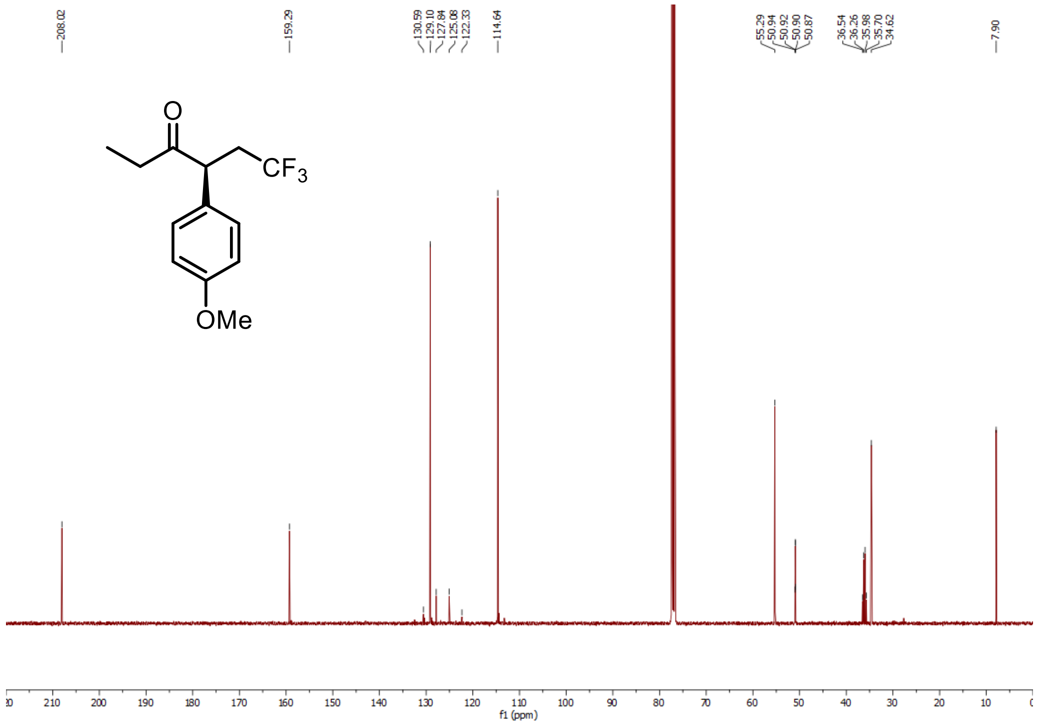

^{19}F NMR (376 MHz, Chloroform-*d*):

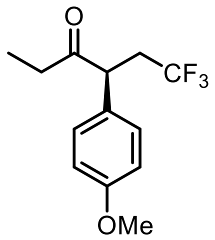

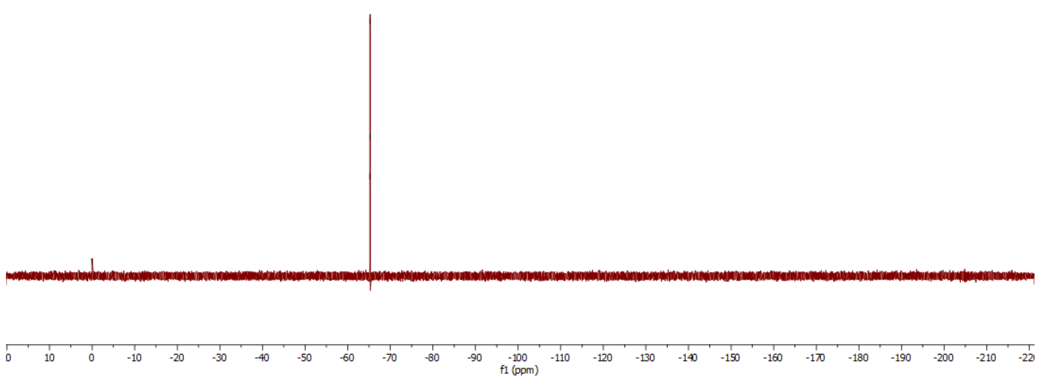

(*S*)-1-Cyclohexyl-4,4,4-trifluoro-2-phenylbutan-1-one (3n)

^{1}H NMR (400 MHz, Chloroform-*d*):

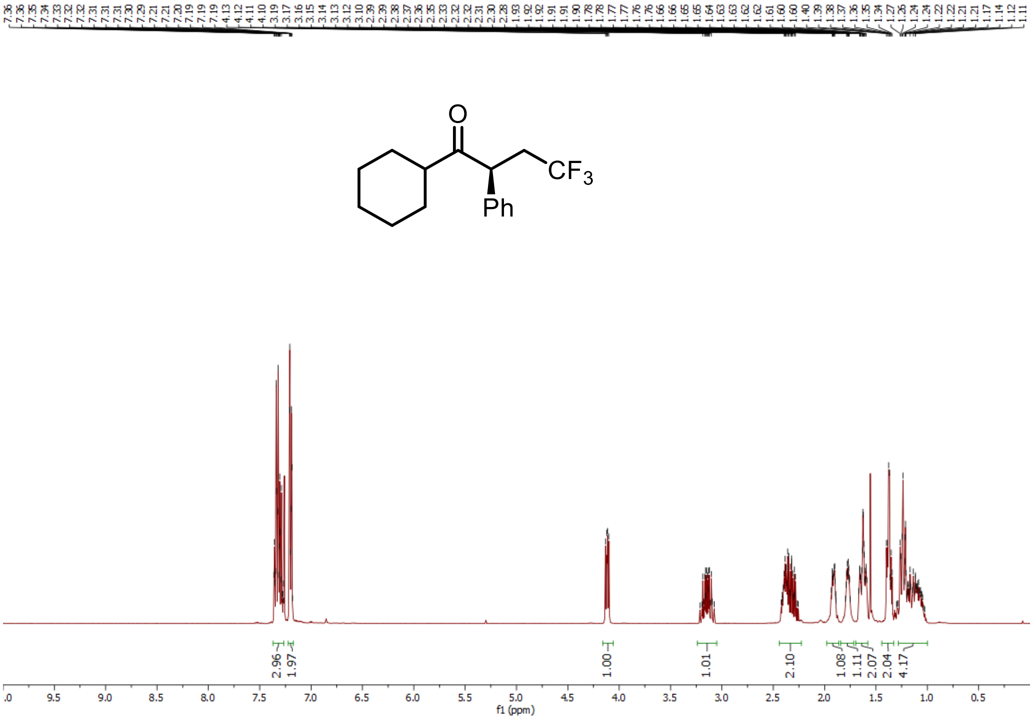

^{13}C NMR (101 MHz, Chloroform-*d*):

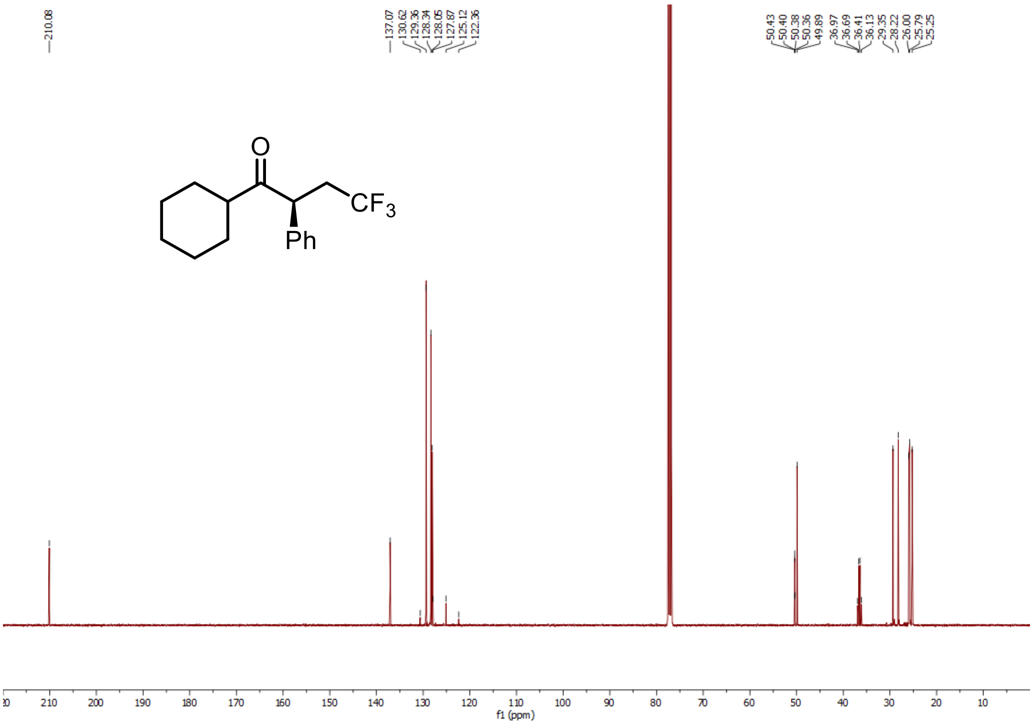

^{19}F NMR (376 MHz, Chloroform-*d*):

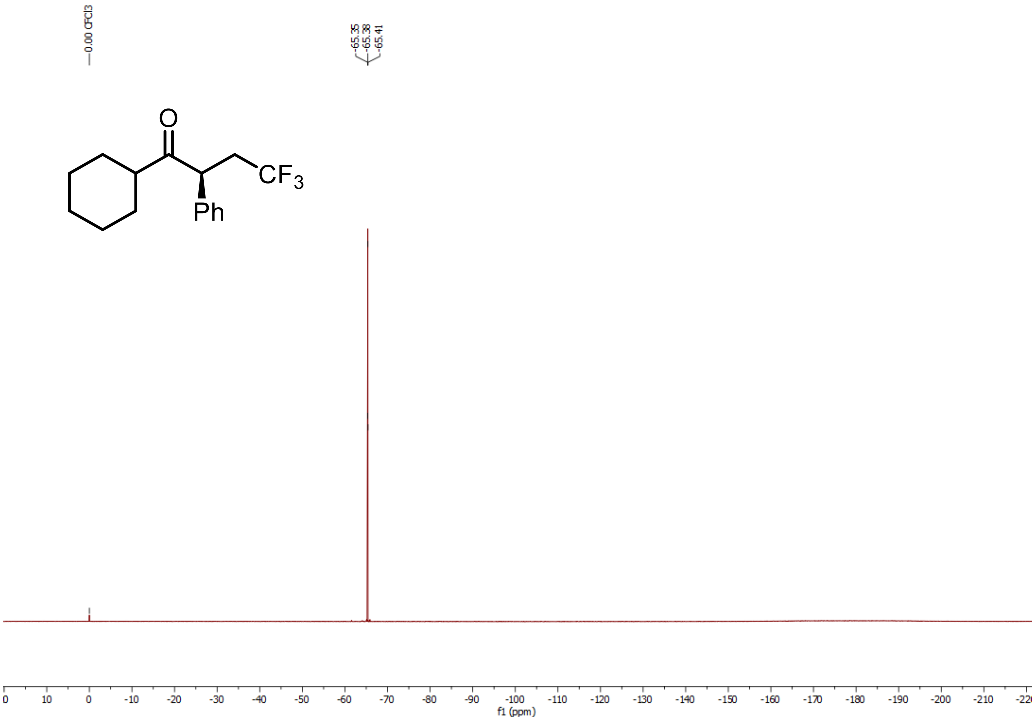

(*S*)-1-Cyclopropyl-4,4,4-trifluoro-2-phenylbutan-1-one (3o)

^{1}H NMR (400 MHz, Chloroform-*d*):

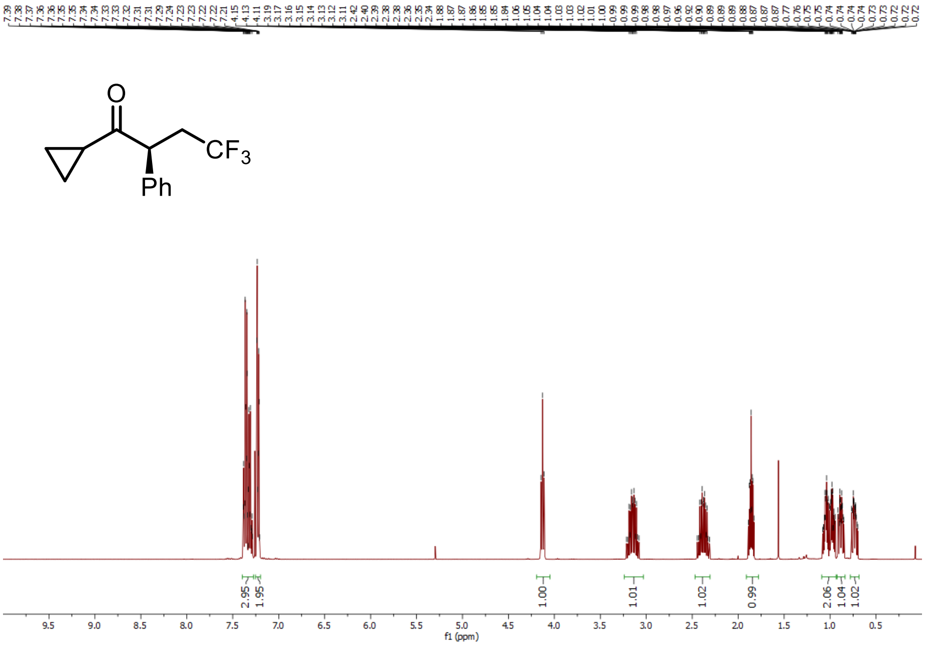

^{13}C NMR (101 MHz, Chloroform-*d*):

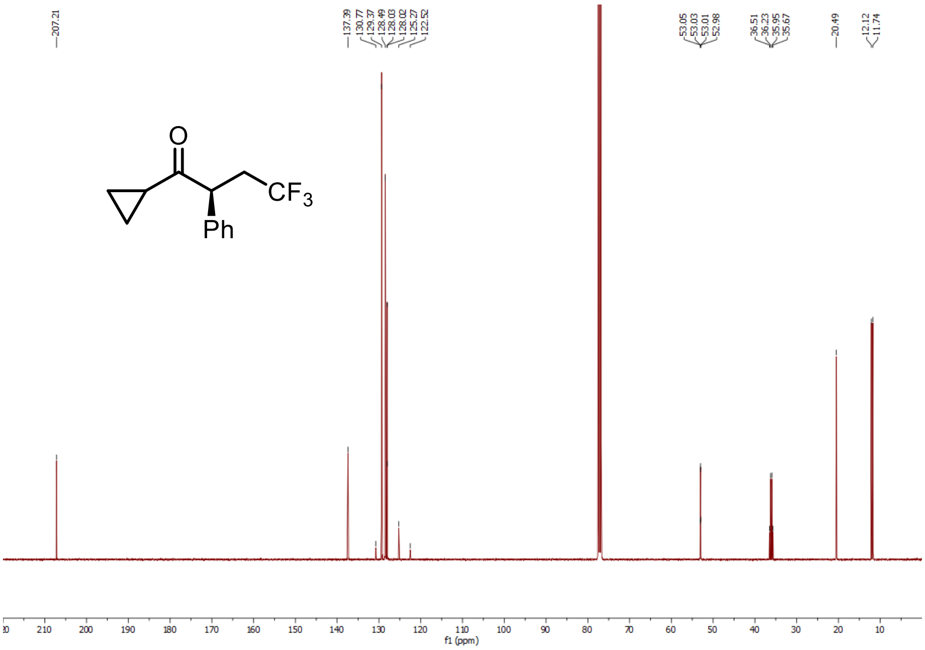

^{19}F NMR (376 MHz, Chloroform-*d*):

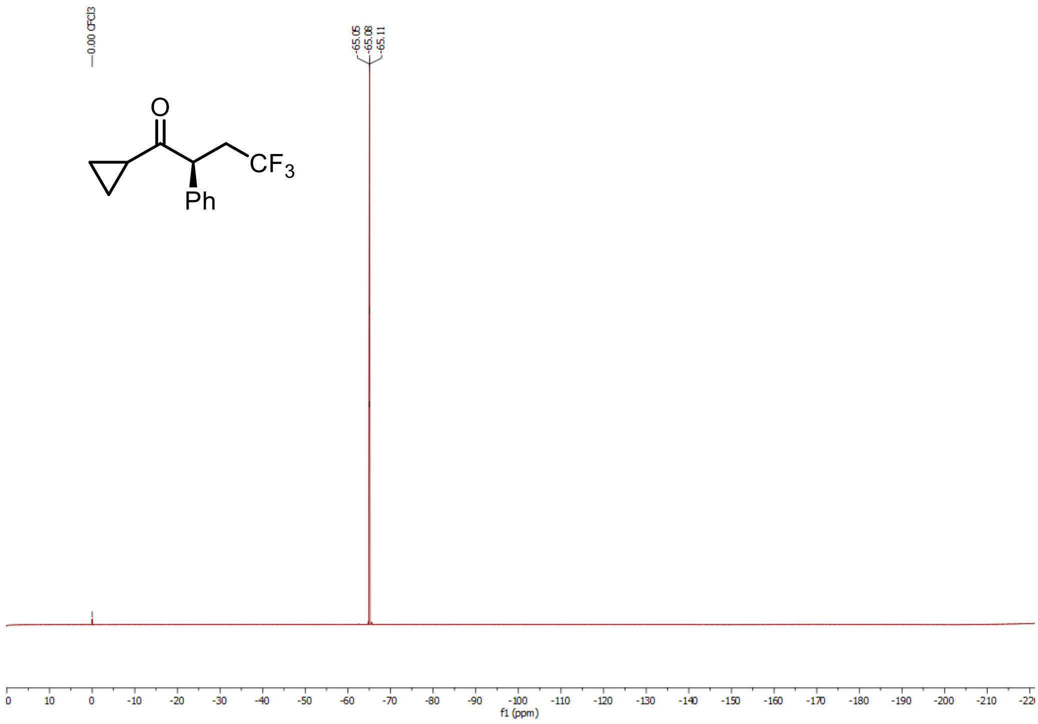

(*S*)-4,4,4-Trifluoro-2-(4-methoxyphenyl)-1-phenylbutan-1-one (4a)

^{1}H NMR (400 MHz, Chloroform-*d*):

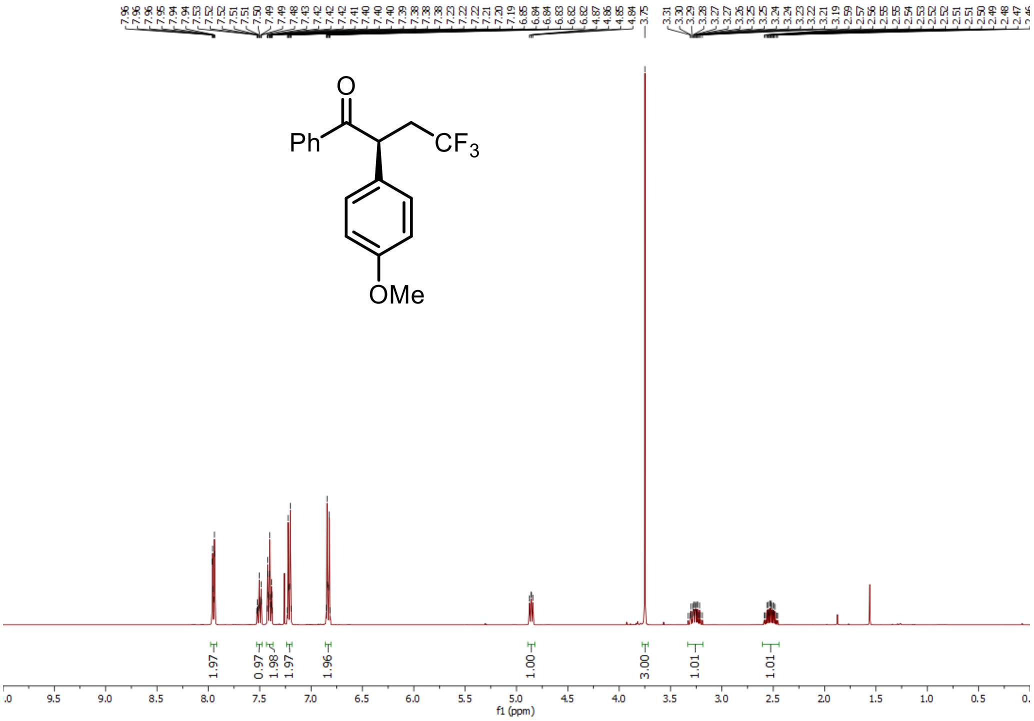

^{13}C NMR (101 MHz, Chloroform-*d*):

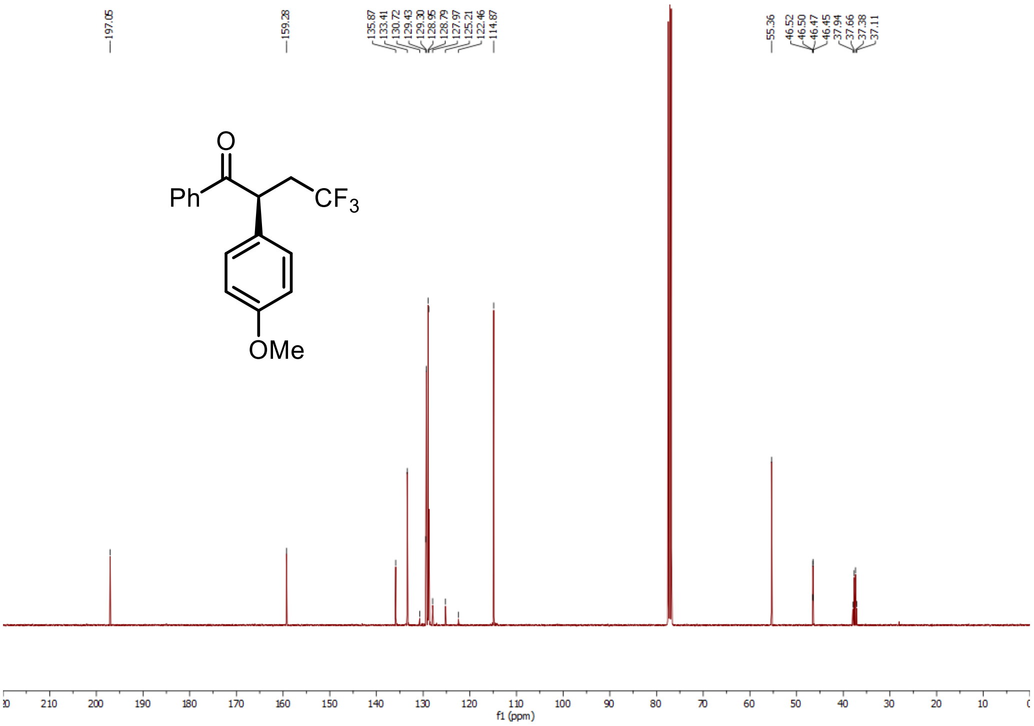

[19]**F NMR** (376 MHz, Chloroform-*d*):

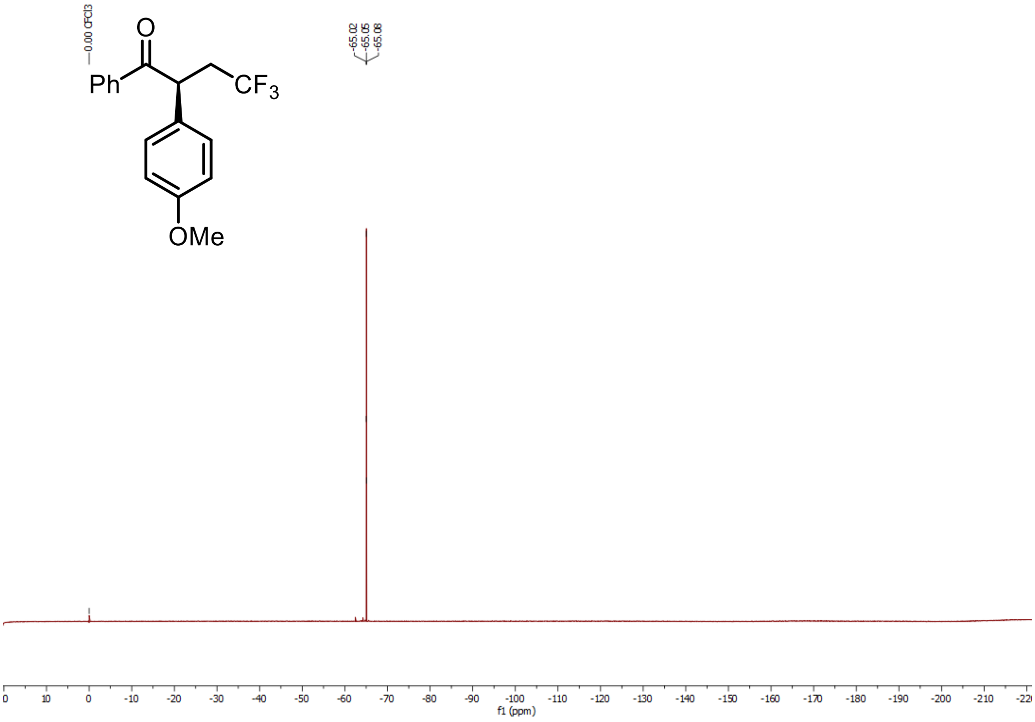

(*S*)-2-([1,1'-Biphenyl]-4-yl)-4,4,4-trifluoro-1-phenylbutan-1-one (4b)

^{1}H NMR (400 MHz, Chloroform-*d*):

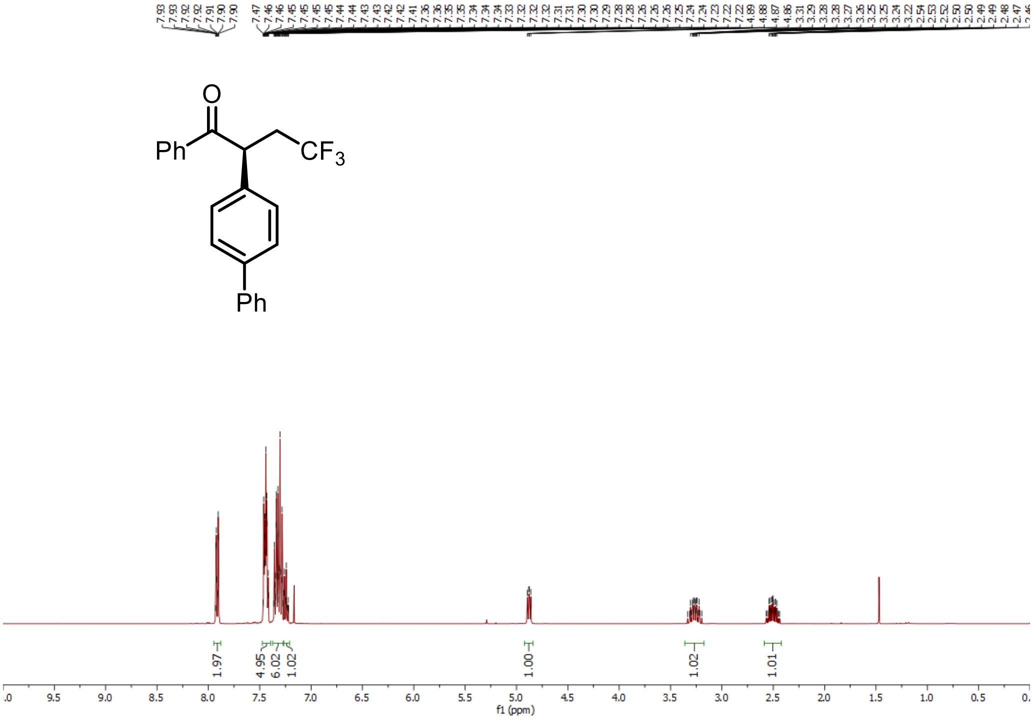

^{13}C NMR (101 MHz, Chloroform-*d*):

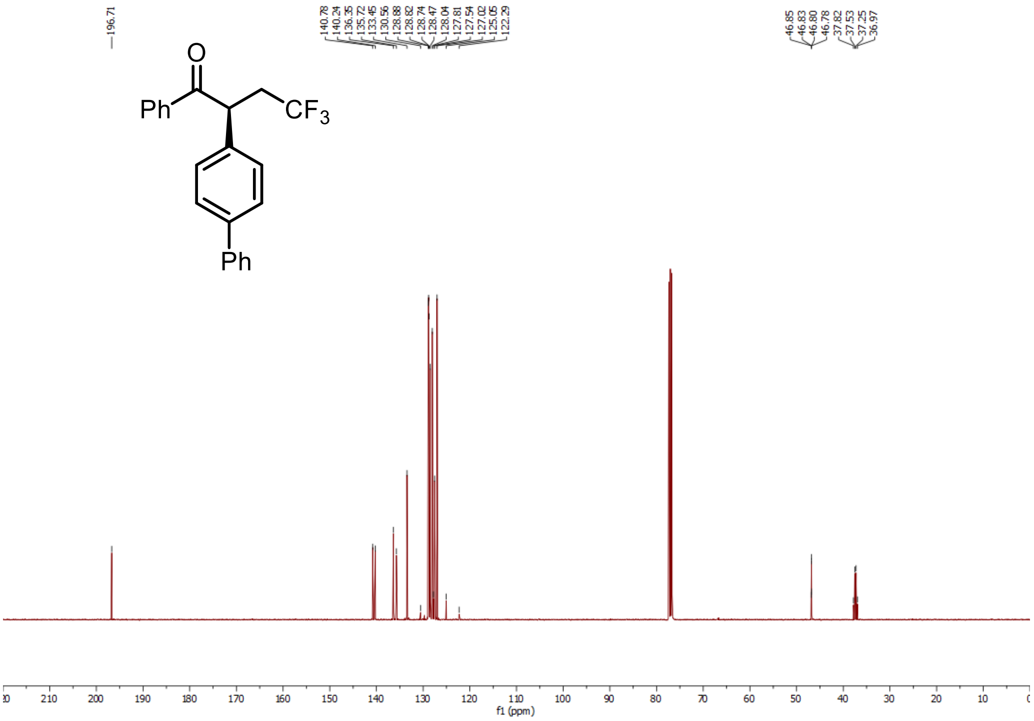

^{19}F NMR (376 MHz, Chloroform-*d*):

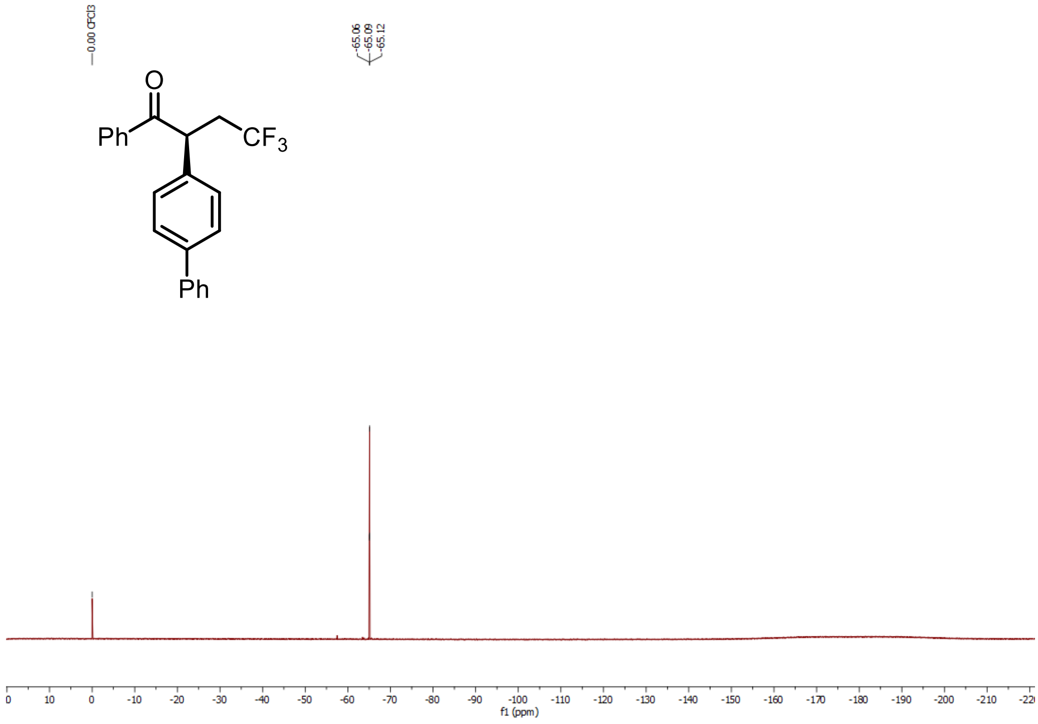

(*S*)-4-(4,4,4-Trifluoro-1-oxo-1-phenylbutan-2-yl)benzonitrile (4c)

^{1}H NMR (400 MHz, Chloroform-*d*):

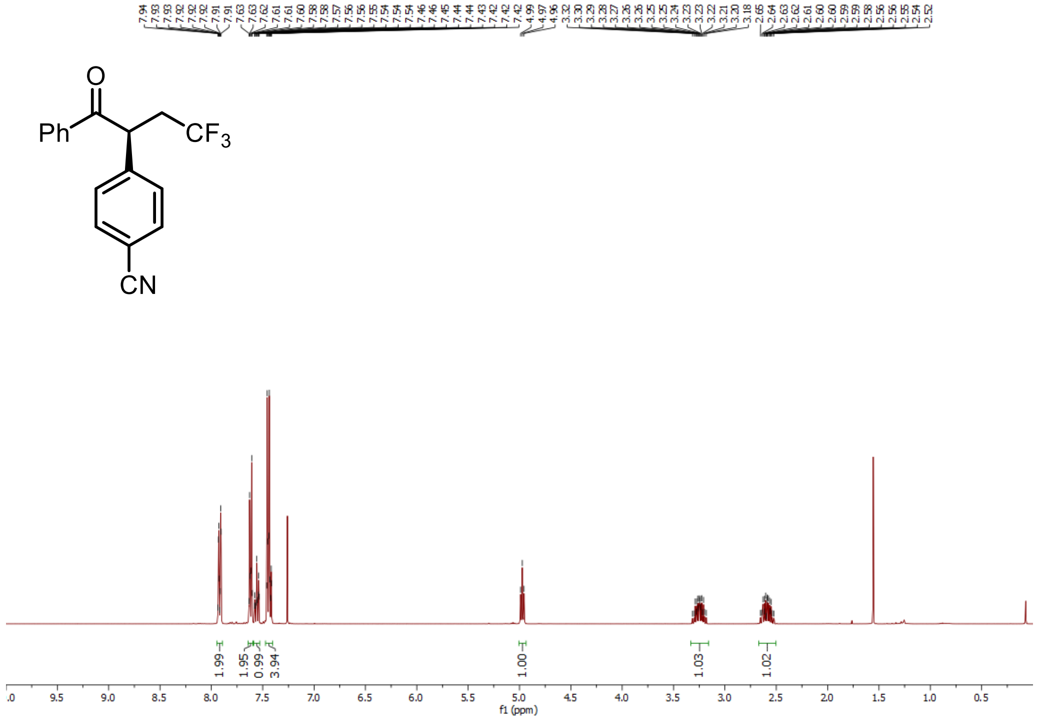

^{13}C NMR (101 MHz, Chloroform-*d*):

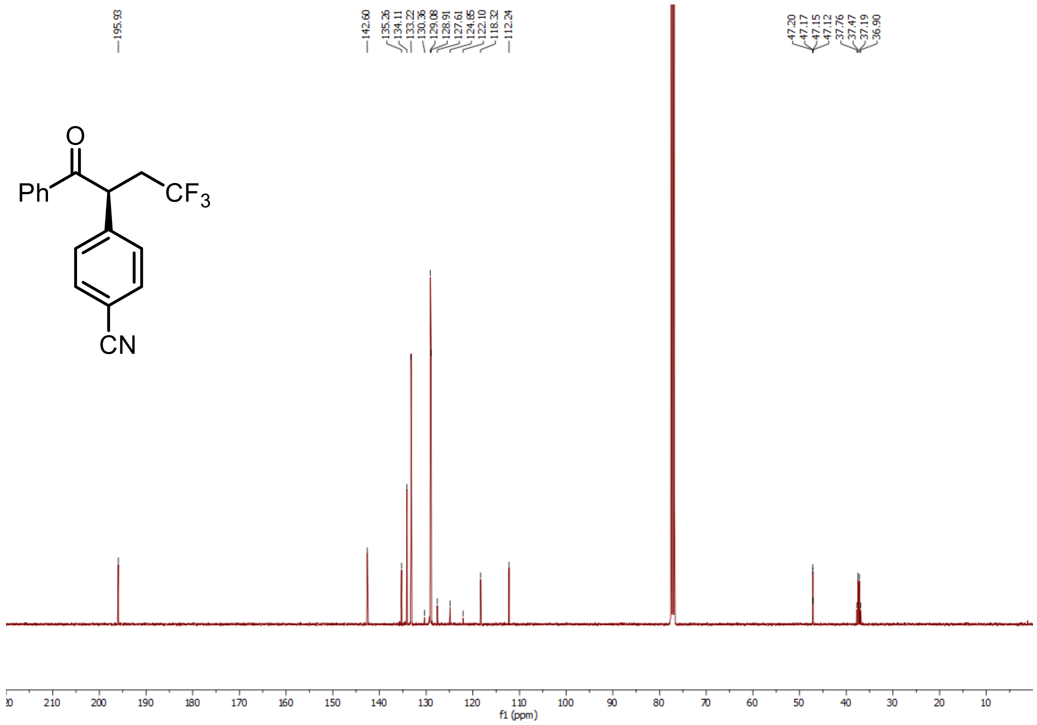

^{19}F NMR (376 MHz, Chloroform-*d*):

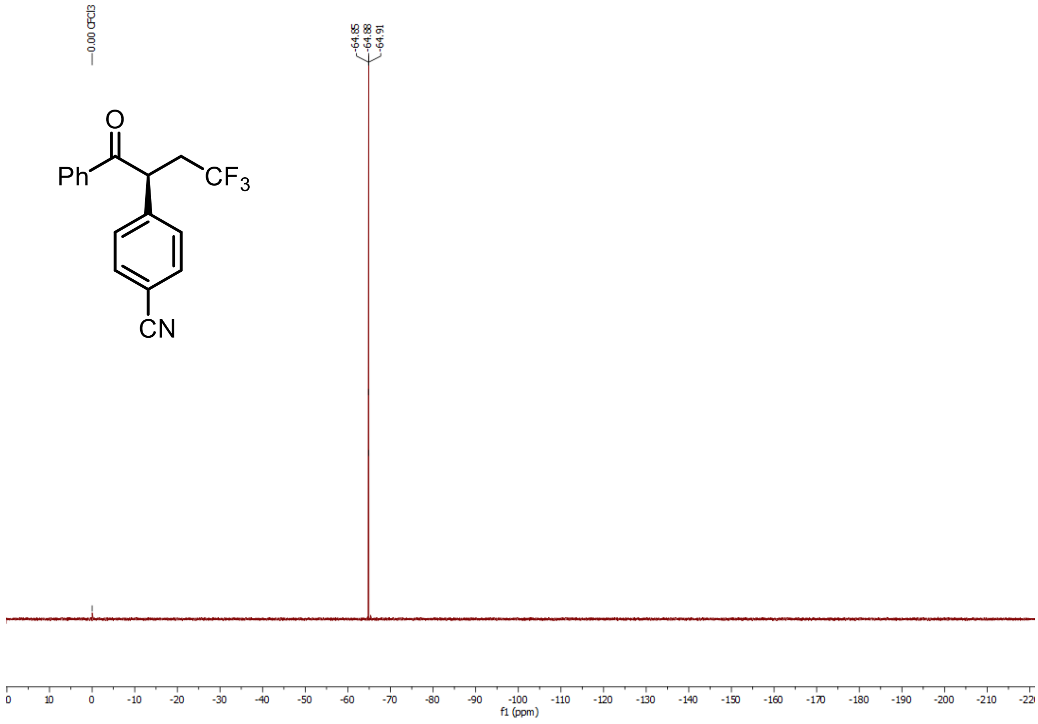

(*S*)-4,4,4-Trifluoro-1-phenyl-2-(*o*-tolyl)butan-1-one (4d)

^{1}H NMR (400 MHz, Chloroform-*d*):

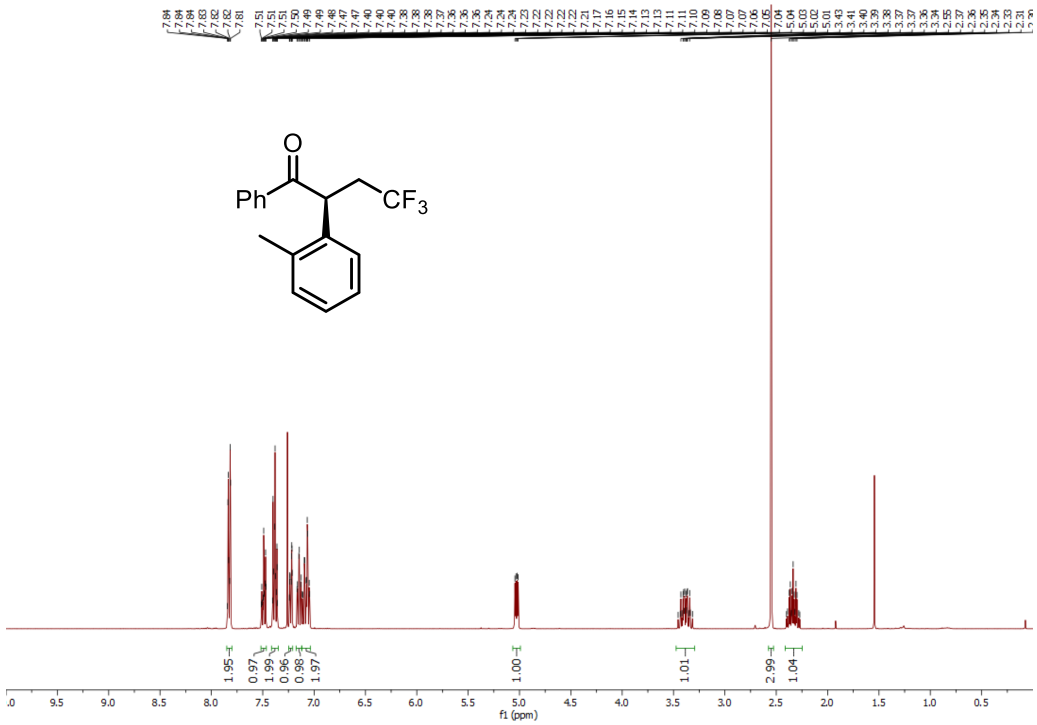

^{13}C NMR (101 MHz, Chloroform-*d*):

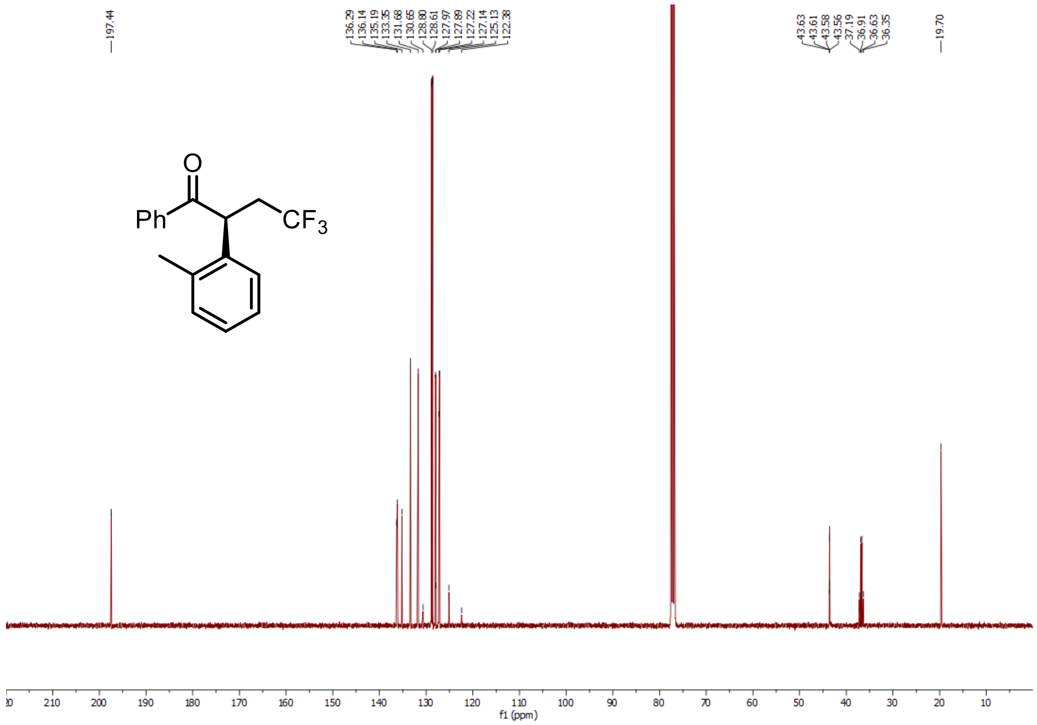

^{19}F NMR (376 MHz, Chloroform-*d*):

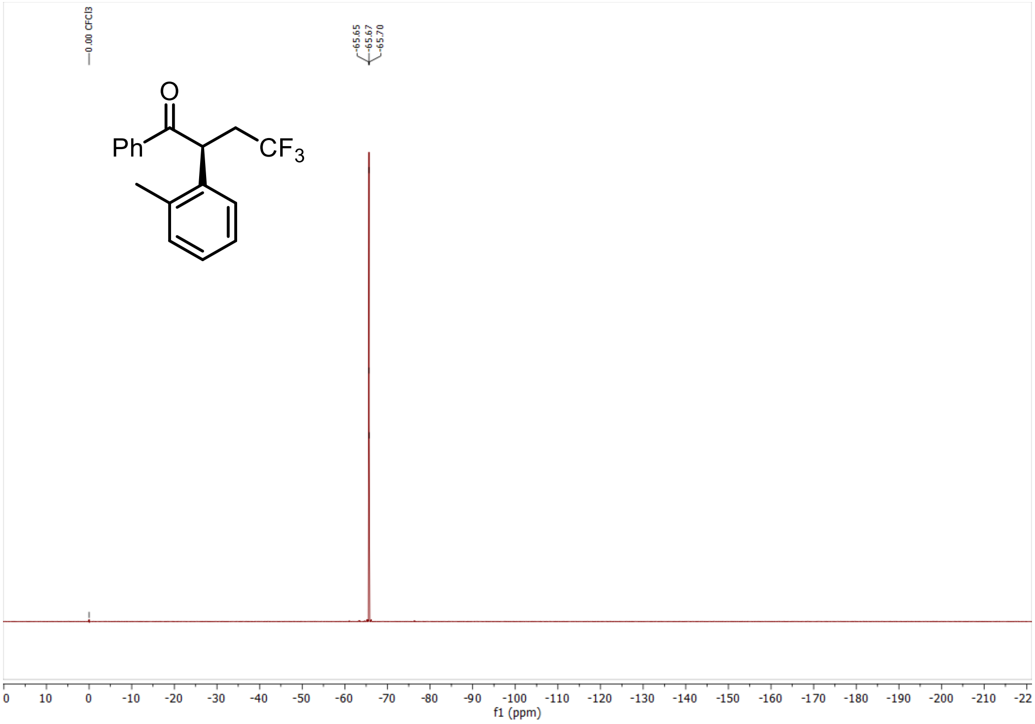

(*S*)-2-(1-Benzyl-1*H*-indol-3-yl)-4,4,4-trifluoro-1-phenylbutan-1-one (4e)

^{1}H NMR (400 MHz, Chloroform-*d*):

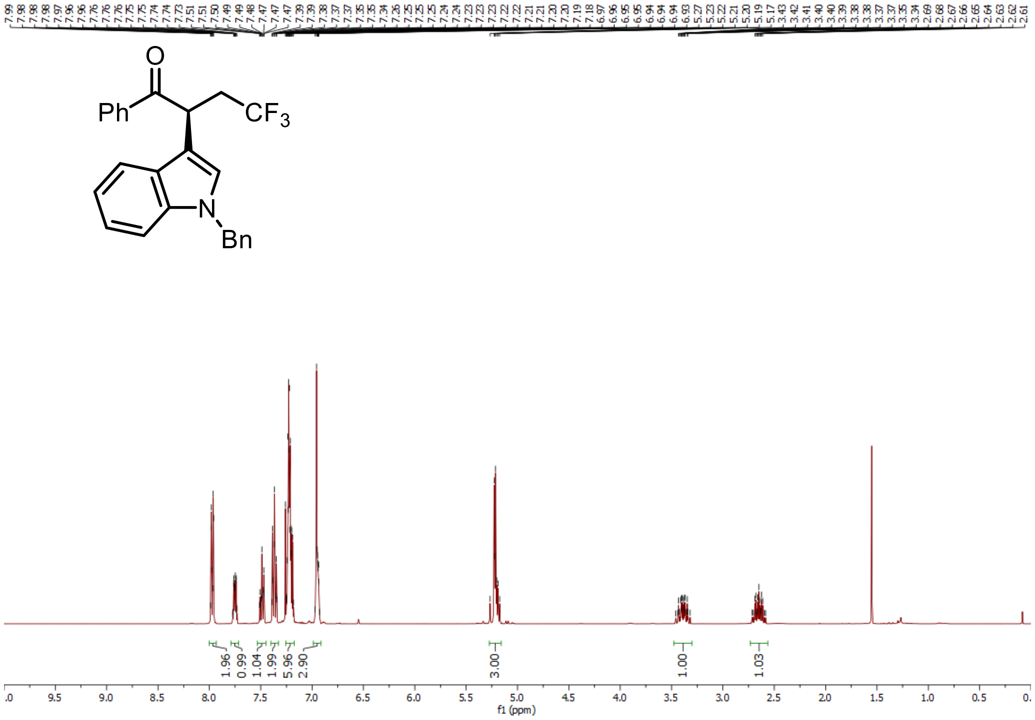

^{13}C NMR (101 MHz, Chloroform-*d*):

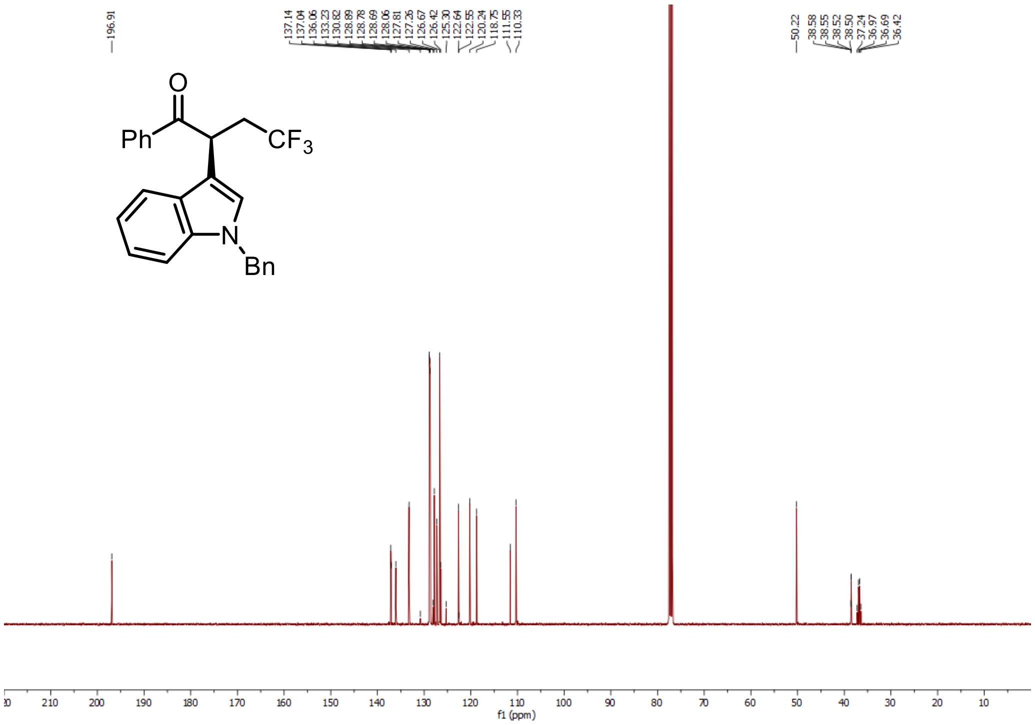

^{19}F NMR (376 MHz, Chloroform-*d*):

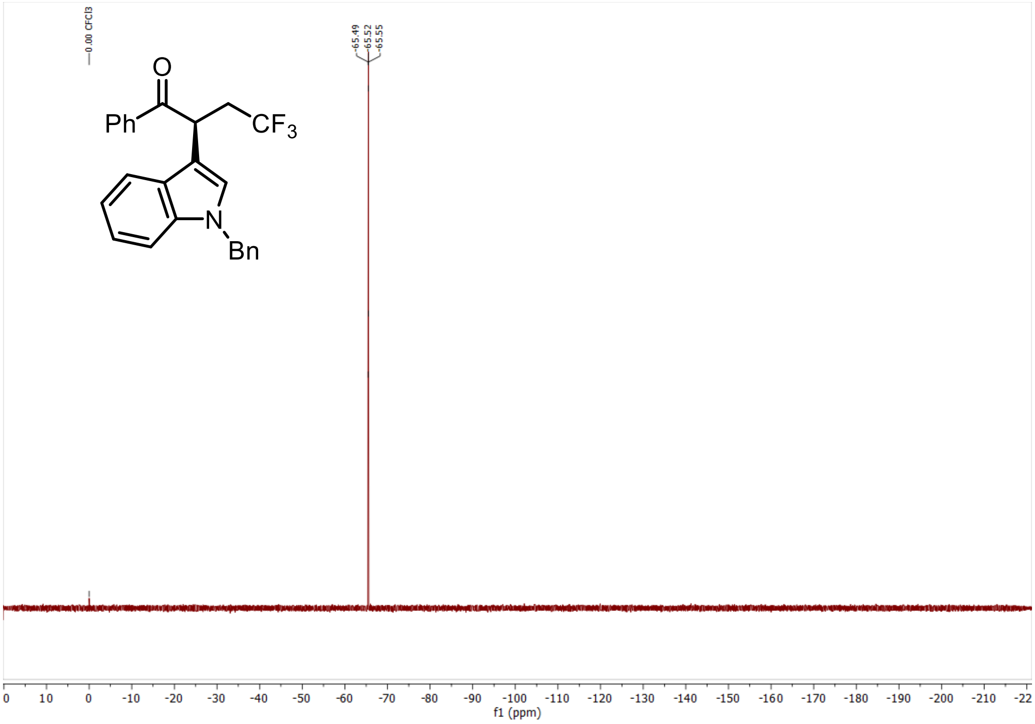

(*R*)-4,4,4-Trifluoro-1-phenyl-2-(thiophen-2-yl)butan-1-one (4f)

^{1}H NMR (400 MHz, Chloroform-*d*):

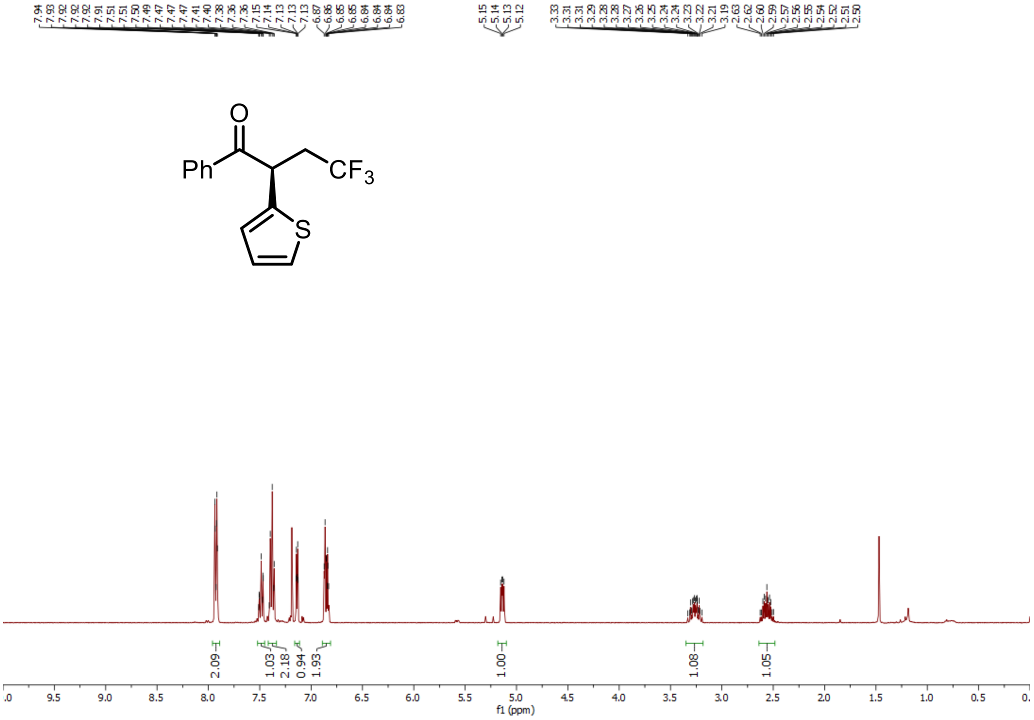

^{13}C NMR (101 MHz, Chloroform-*d*):

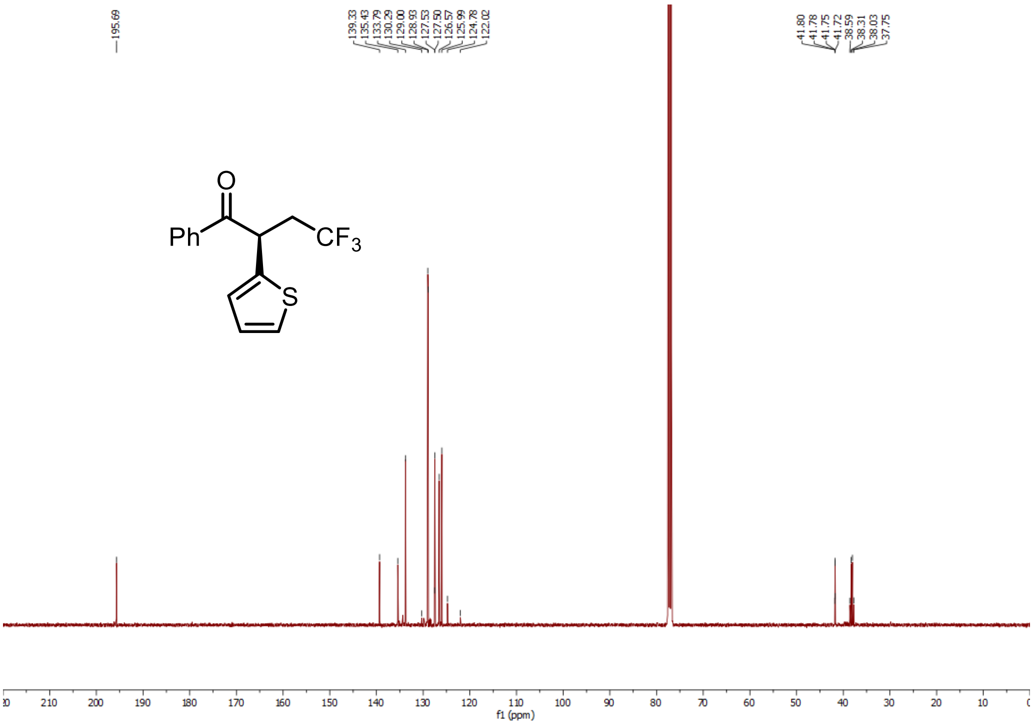

^{19}F NMR (376 MHz, Chloroform-*d*):

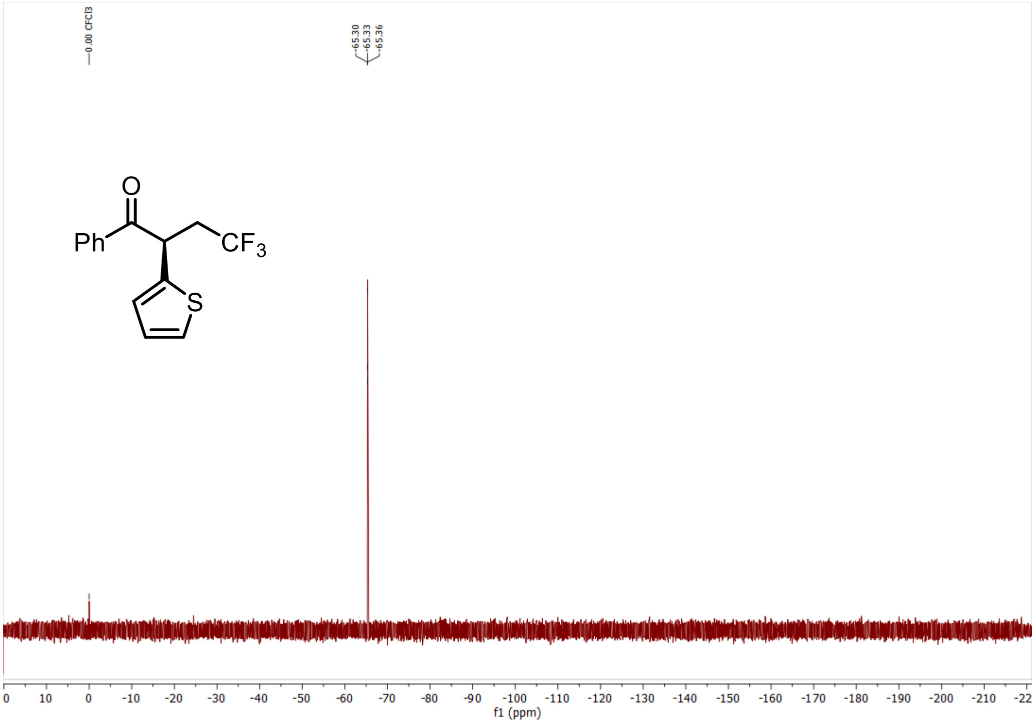

(*S*)-4,4,4-Trifluoro-1-phenyl-2-(pyridin-3-yl)butan-1-one (4g)

^{1}H NMR (400 MHz, Chloroform-*d*):

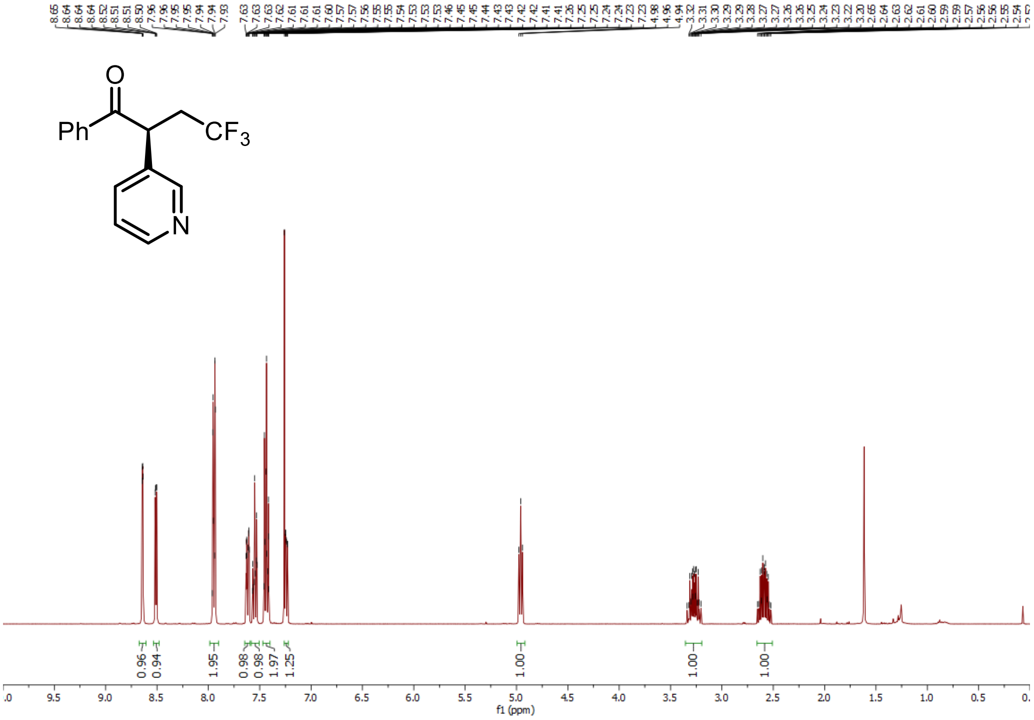

^{13}C NMR (101 MHz, Chloroform-*d*):

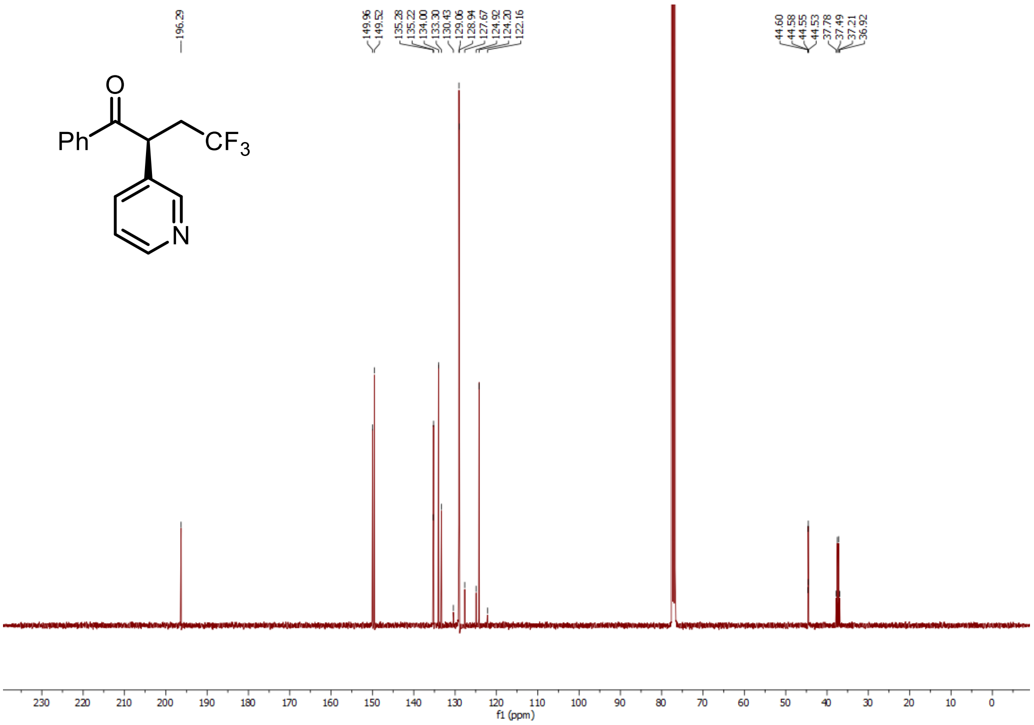

^{19}F NMR (376 MHz, Chloroform-*d*):

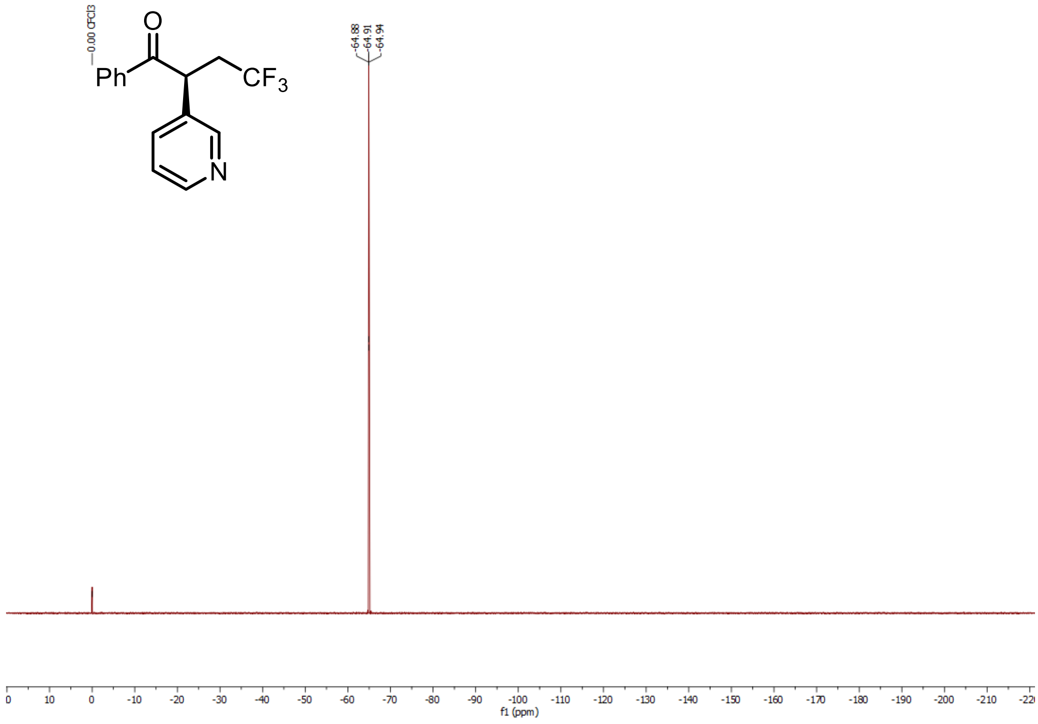

(*R*)-4,4,4-Trifluoro-1-phenyl-2-(phenylthio)butan-1-one (4h)

^{1}H NMR (400 MHz, Chloroform-*d*):

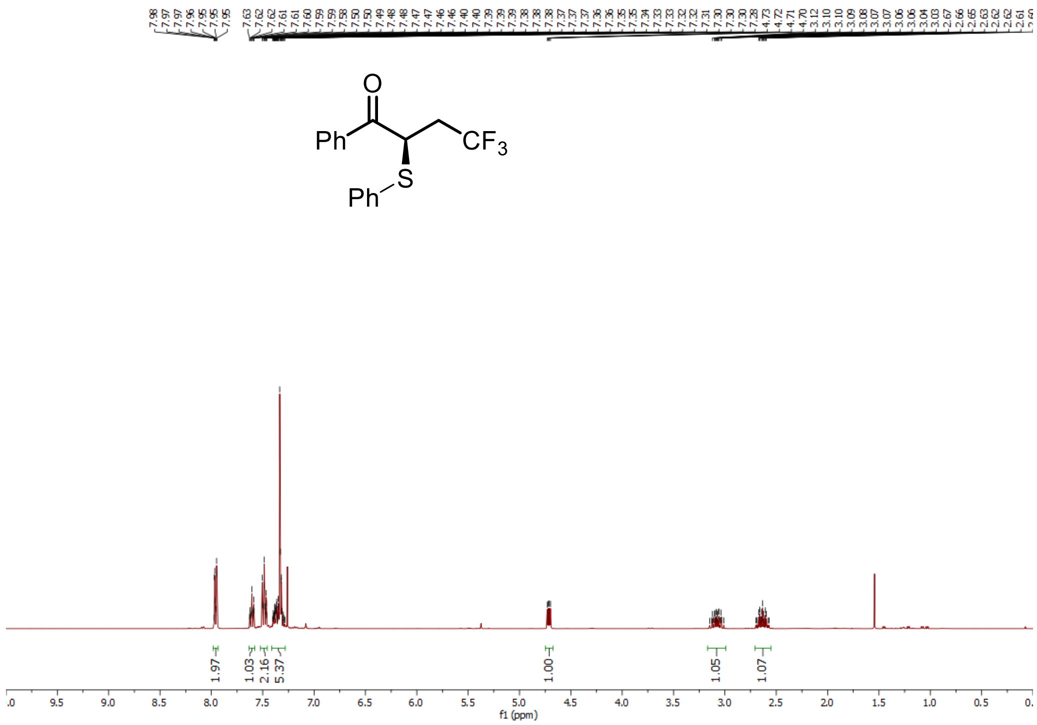

^{13}C NMR (101 MHz, Chloroform-*d*):

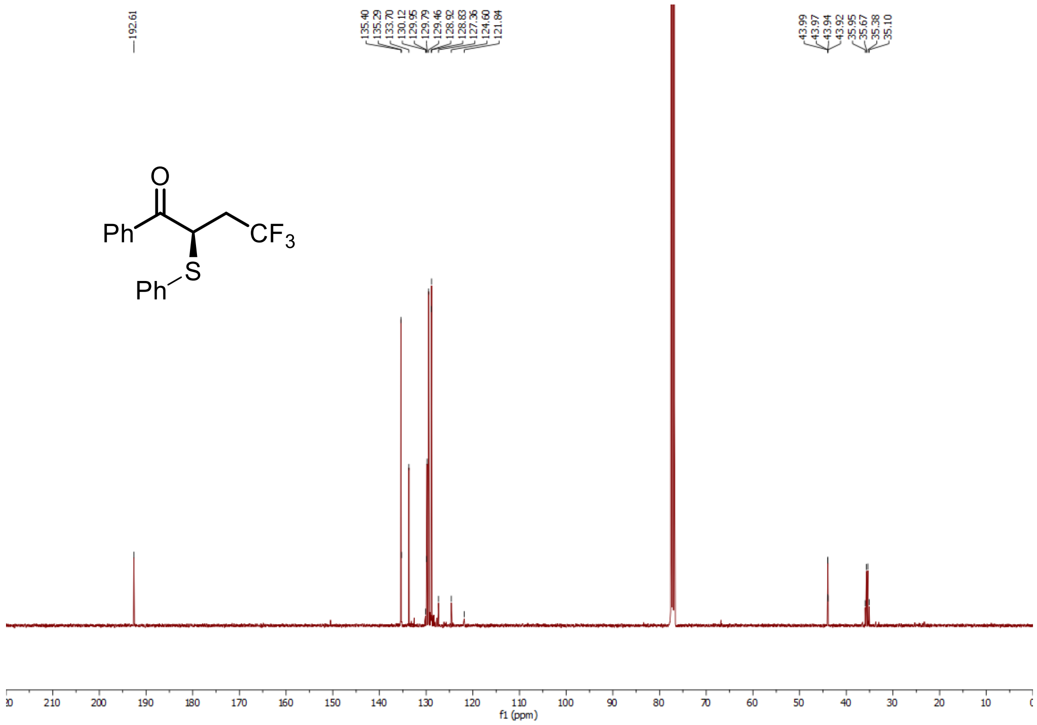

^{19}F NMR (376 MHz, Chloroform-*d*):

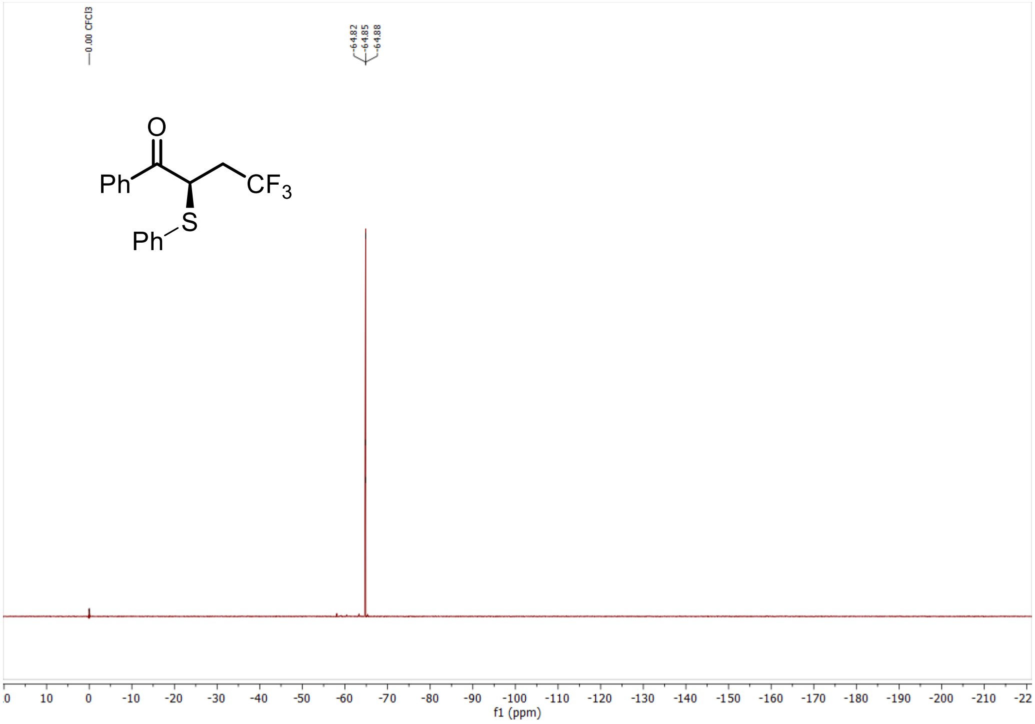

(*R*)-2-(4,4,4-Trifluoro-1-oxo-1-phenylbutan-2-yl)isoindoline-1,3-dione (4i)

^{1}H NMR (400 MHz, Chloroform-*d*):

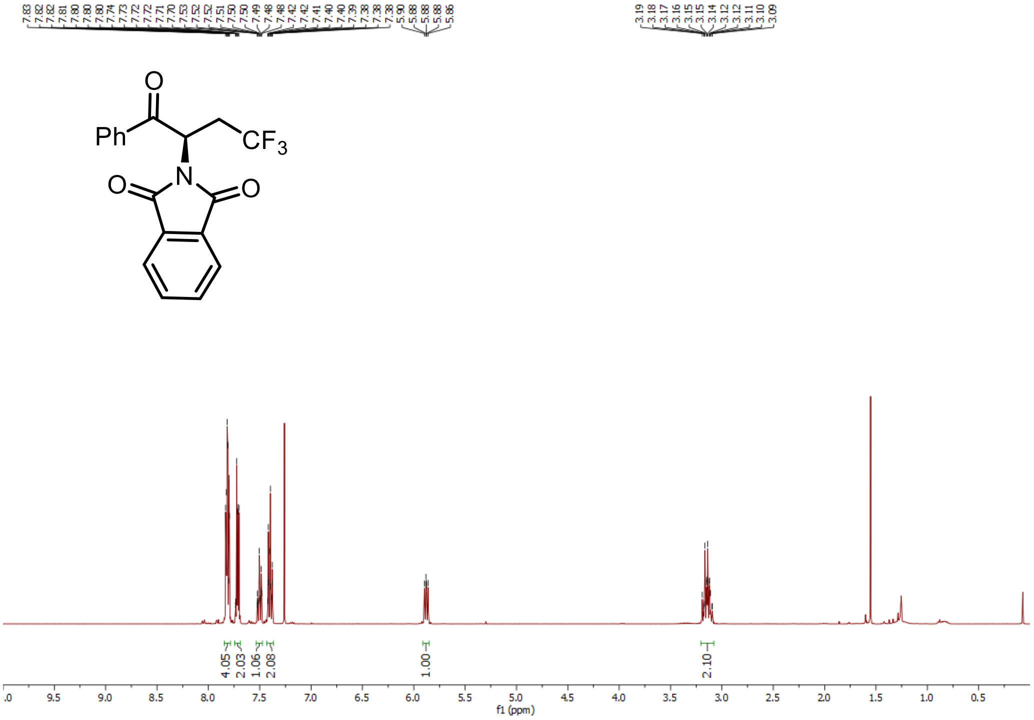

^{13}C NMR (101 MHz, Chloroform-*d*):

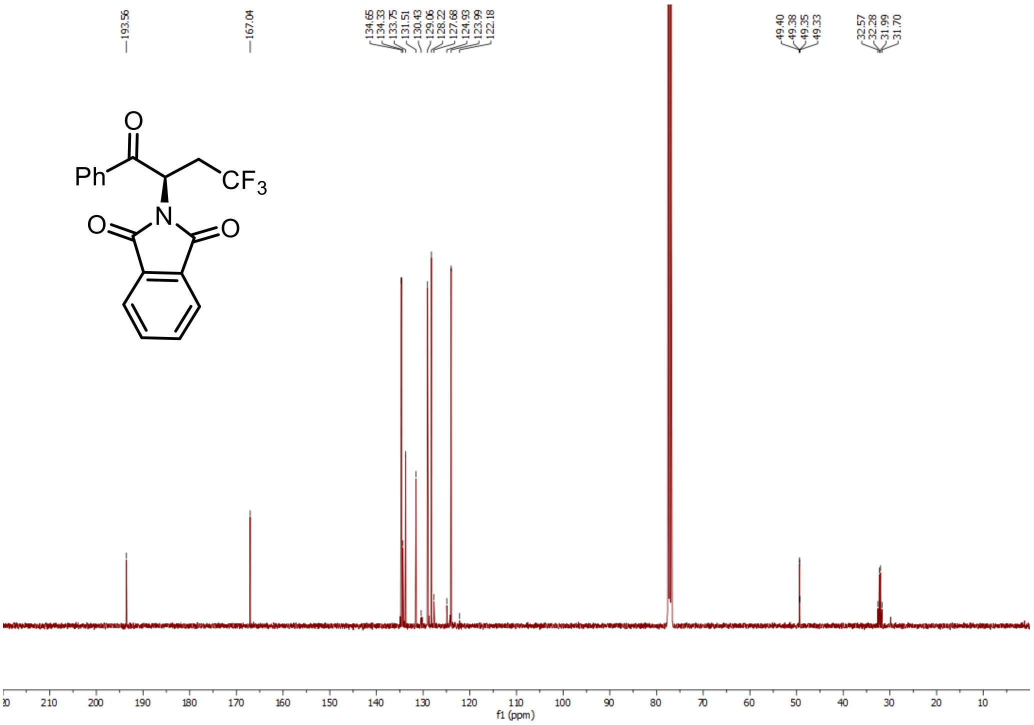

^{19}F NMR (376 MHz, Chloroform-*d*):

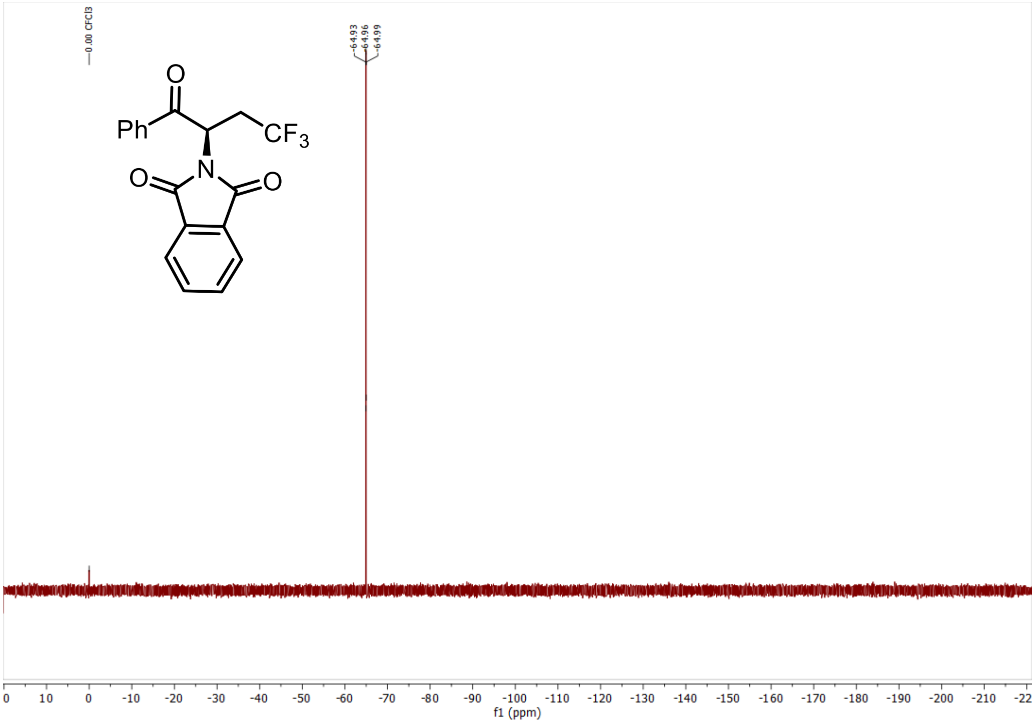

(*R*)-4,4-Dimethyl-1-phenyl-2-(2,2,2-trifluoroethyl)pentan-1-one (4j)

^{1}H NMR (400 MHz, Chloroform-*d*):

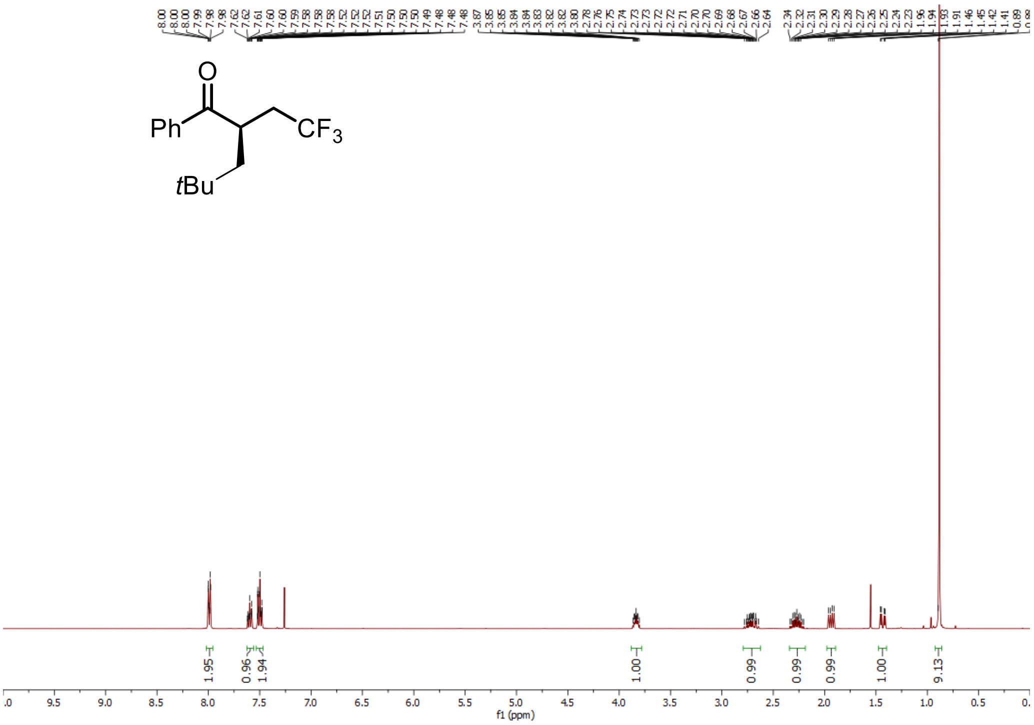

^{13}C NMR (101 MHz, Chloroform-*d*):

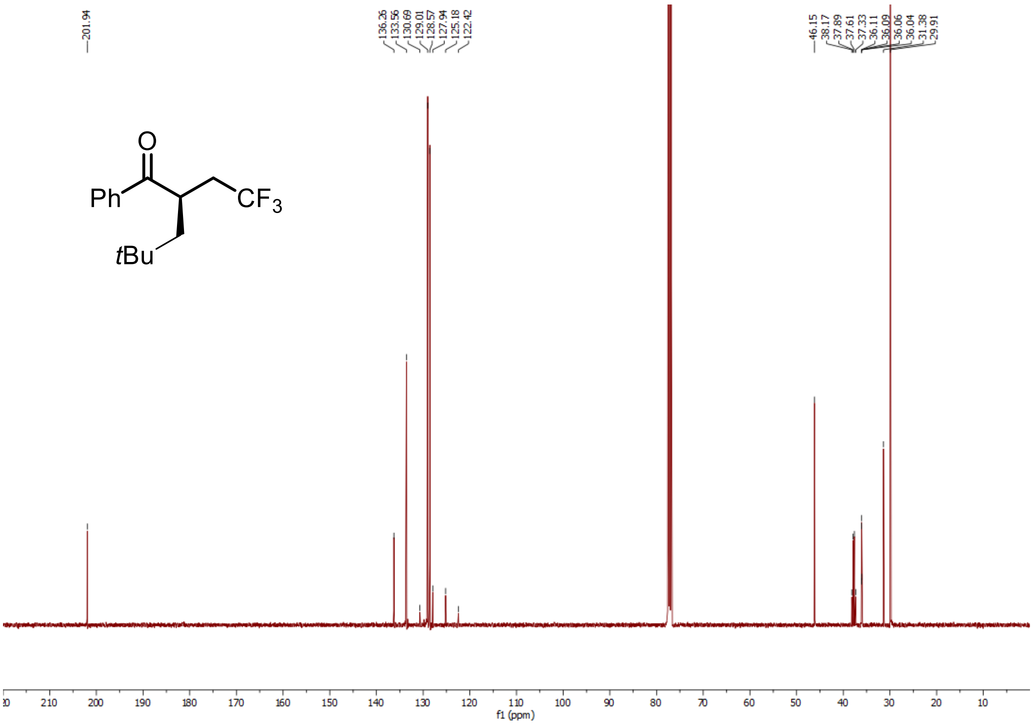

^{19}F NMR (376 MHz, Chloroform-*d*):

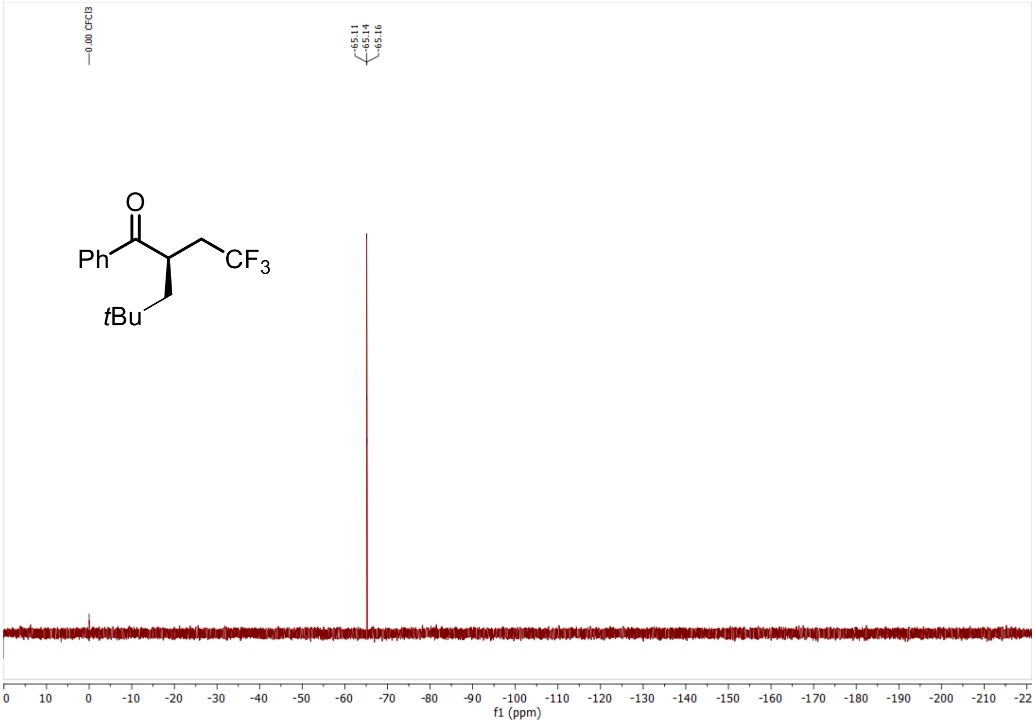

(*S*)-2-Cyclohexyl-4,4,4-trifluoro-1-phenylbutan-1-one (4k)

^{1}H NMR (400 MHz, Chloroform-*d*):

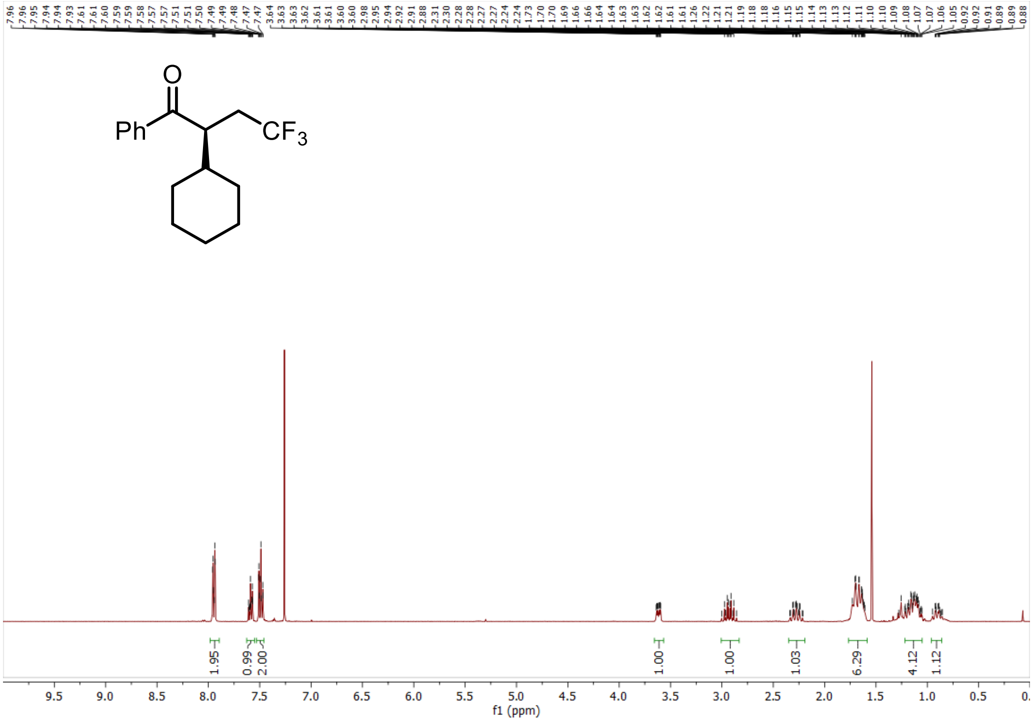

^{13}C NMR (101 MHz, Chloroform-*d*):

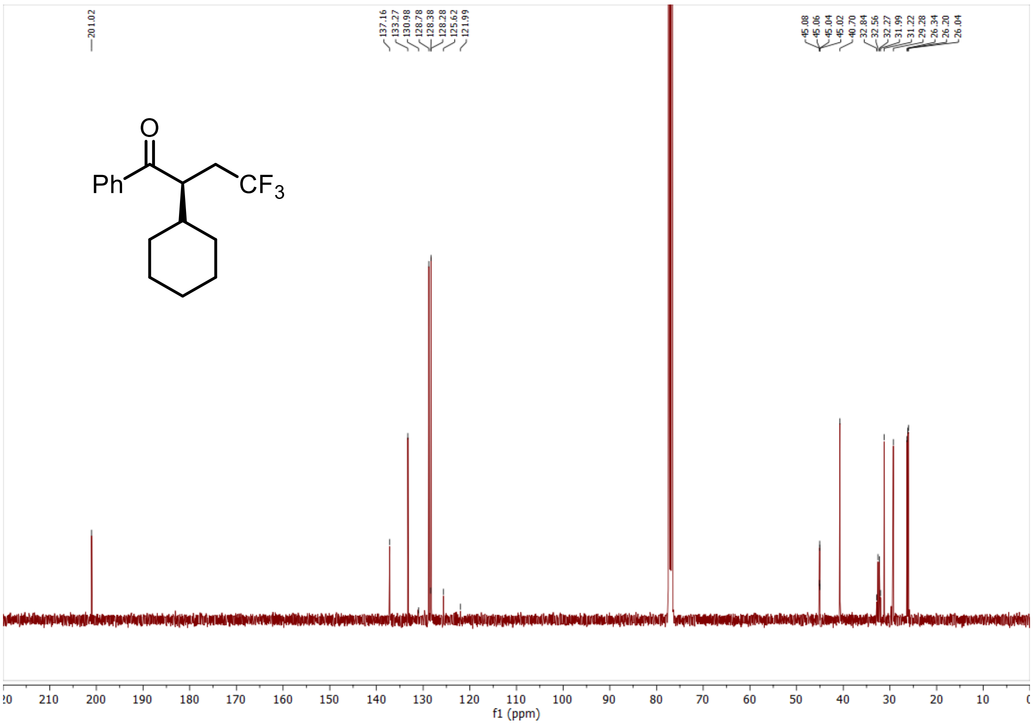

^{19}F NMR (376 MHz, Chloroform-*d*):

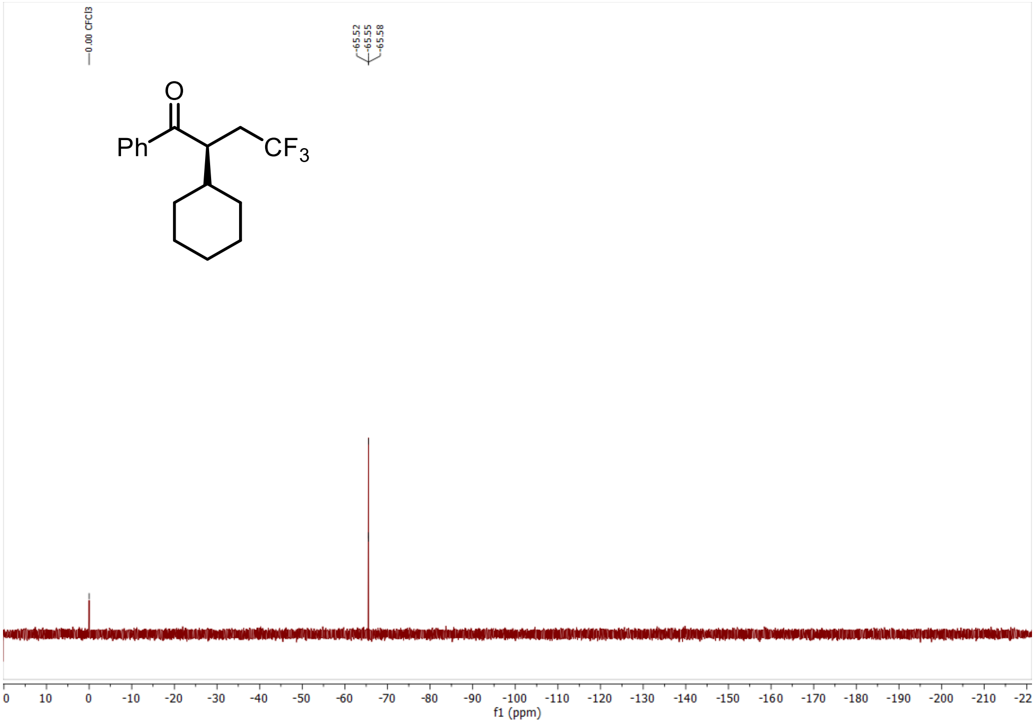

(*S*)-4,4,5,5,6,6,6-Heptafluoro-1,2-diphenylhexan-1-one (4l)

^{1}H NMR (400 MHz, Chloroform-*d*):

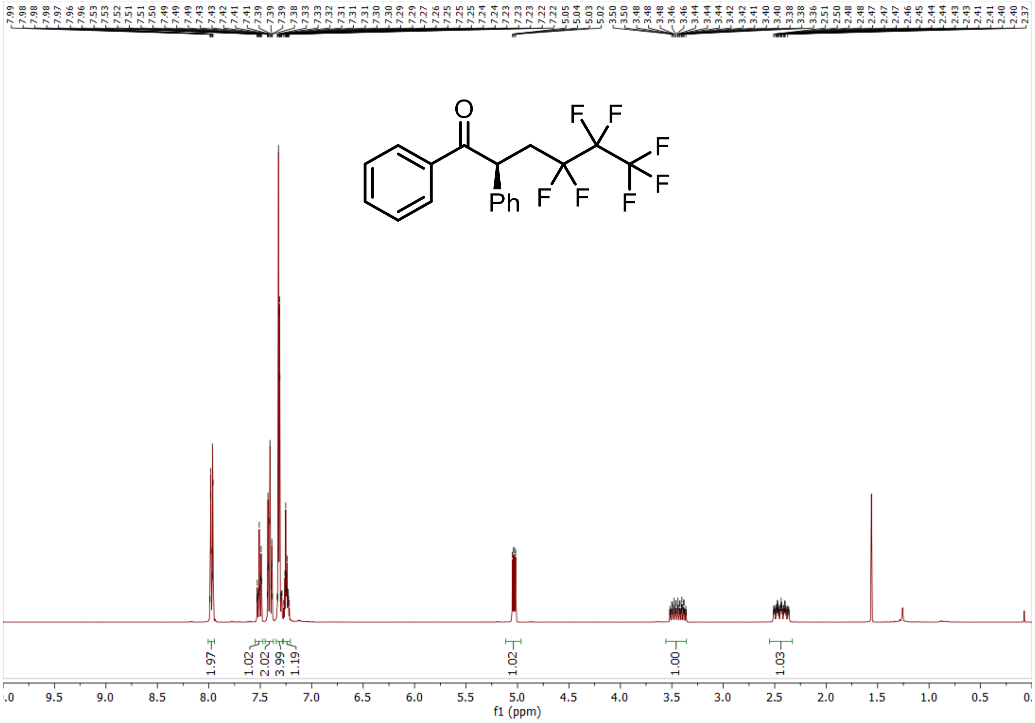

^{13}C NMR (101 MHz, Chloroform-*d*):

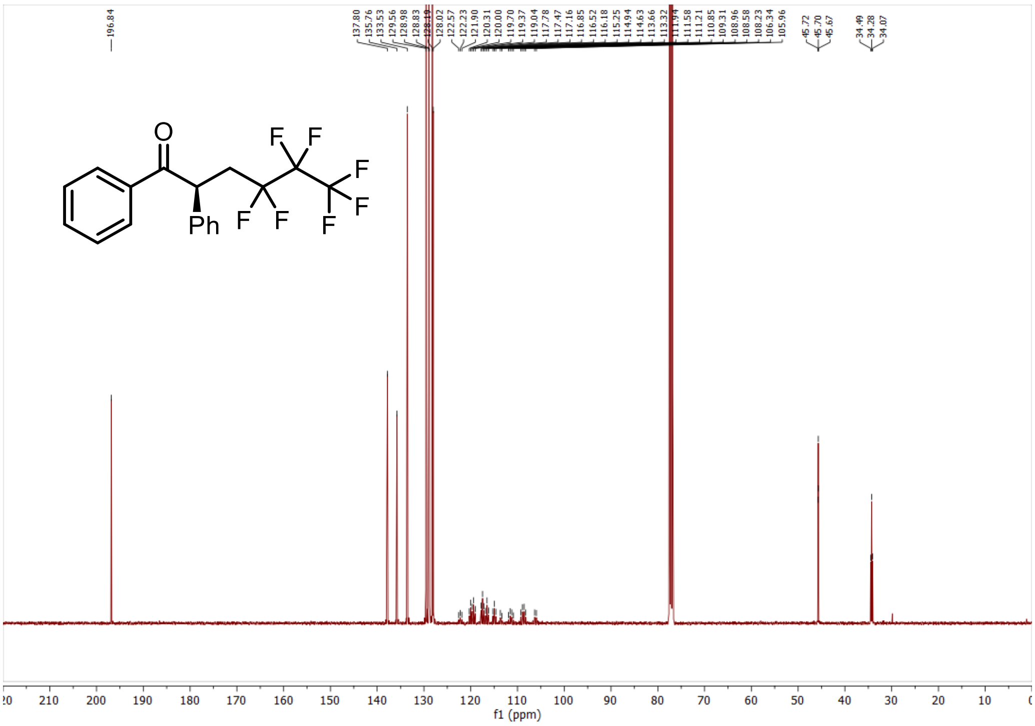

^{19}F NMR (376 MHz, Chloroform-*d*):

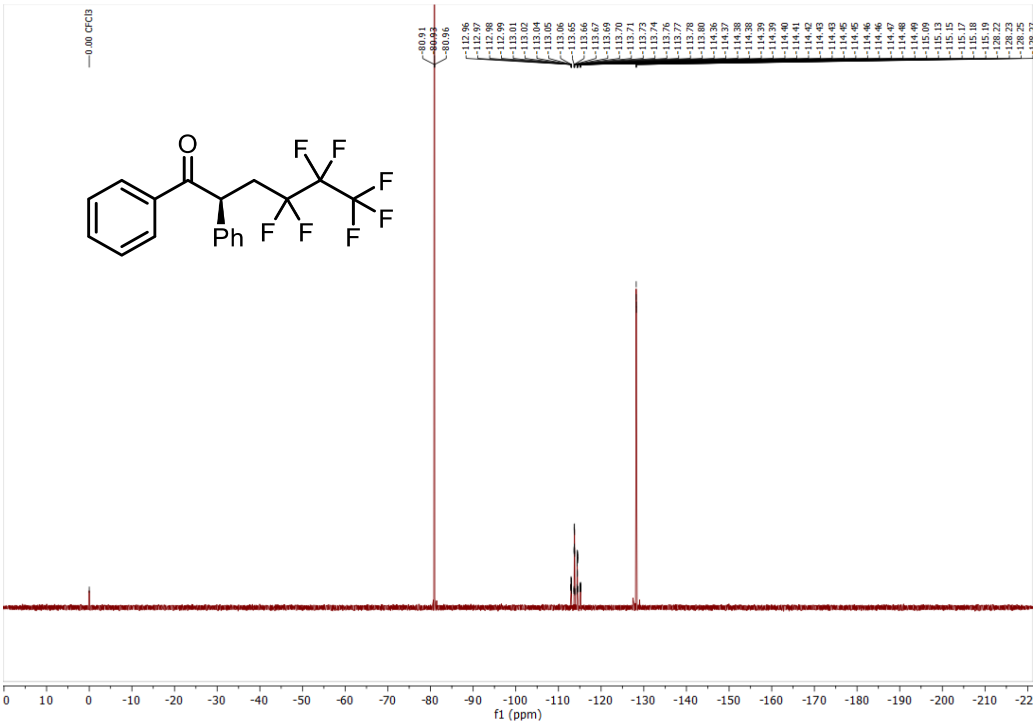

(*S*)-4,4,5,5,6,6,7,7,7-Nonafluoro-1,2-diphenylheptan-1-one (4m)

^{1}H NMR (400 MHz, Chloroform-*d*):

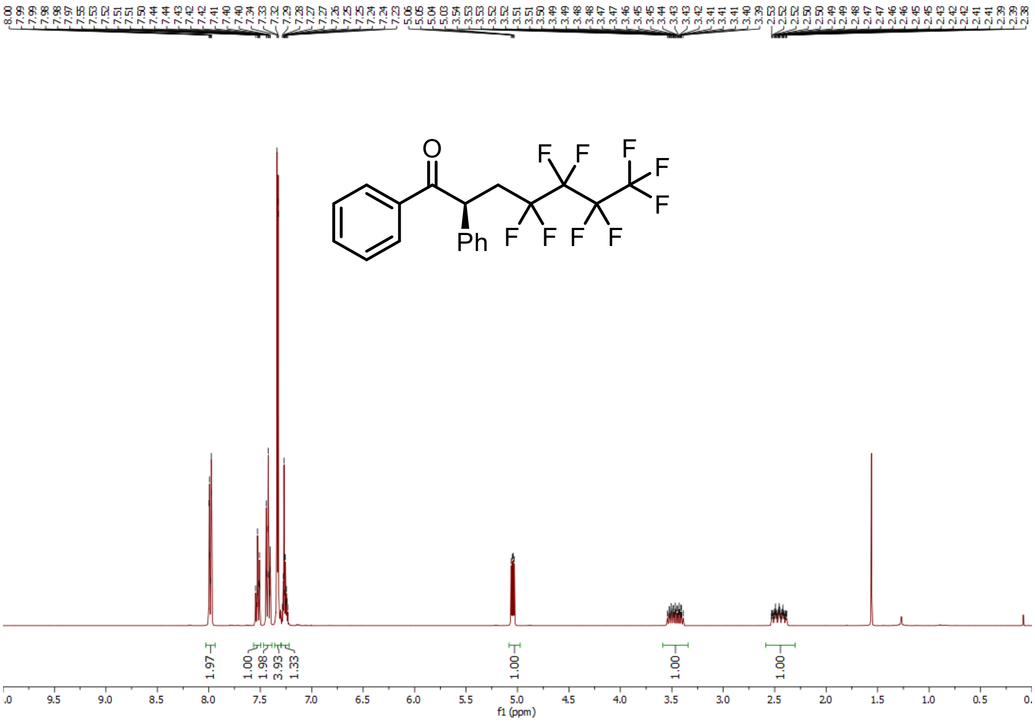

^{13}C NMR (101 MHz, Chloroform-*d*):

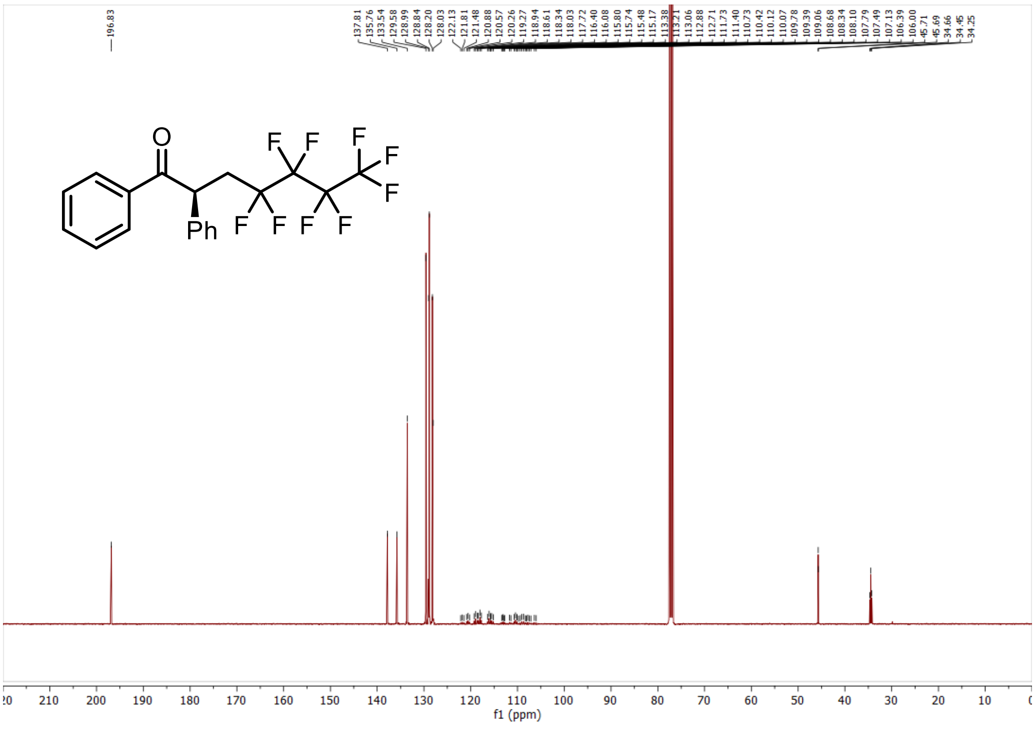

^{19}F NMR (376 MHz, Chloroform-*d*):

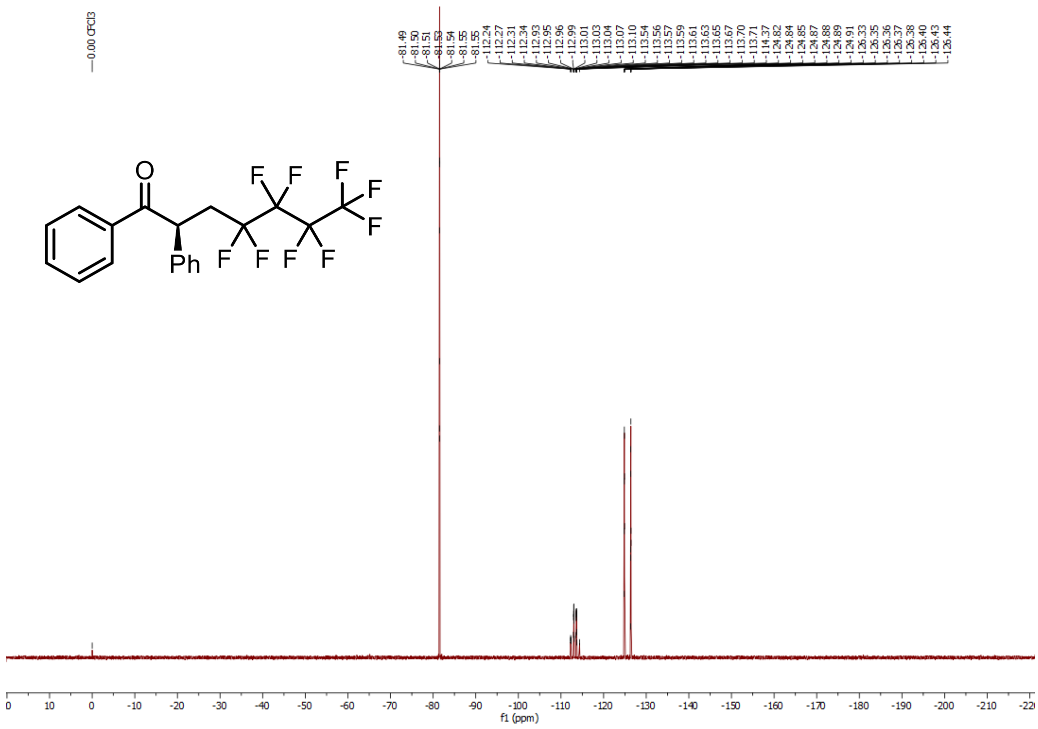

Isopropyl (*S*)-2-methyl-2-(4-(4-(4,4,4-trifluoro-1-oxo-1-phenylbutan-2-yl)benzoyl)phenoxy)propanoate (6a)

^{1}H NMR (400 MHz, Chloroform-*d*):

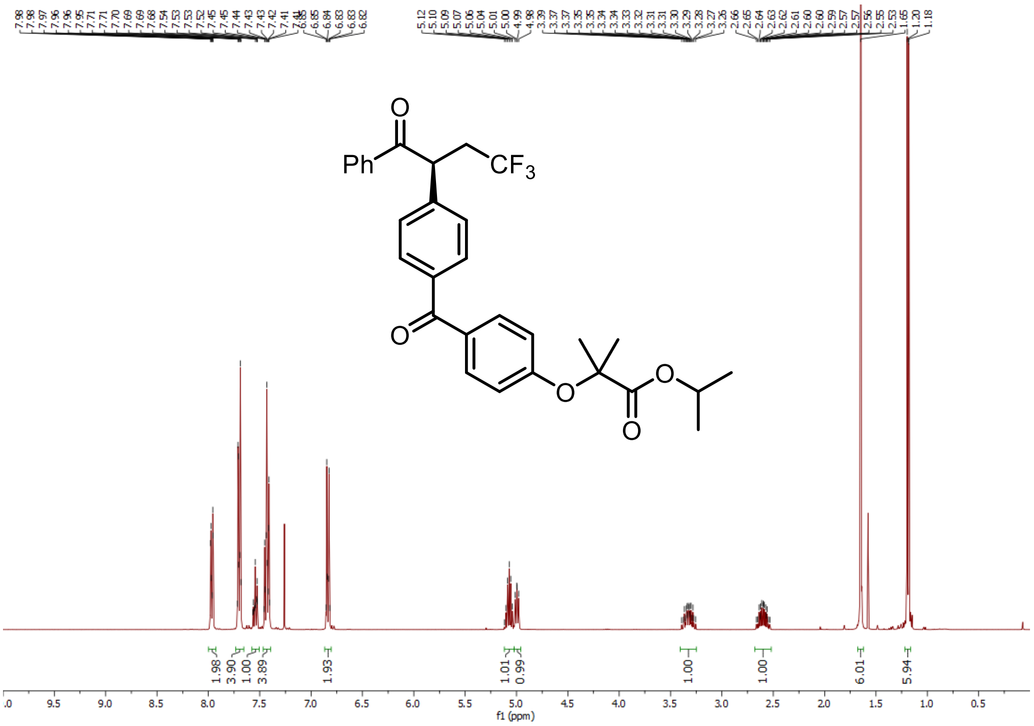

^{13}C NMR (101 MHz, Chloroform-*d*):

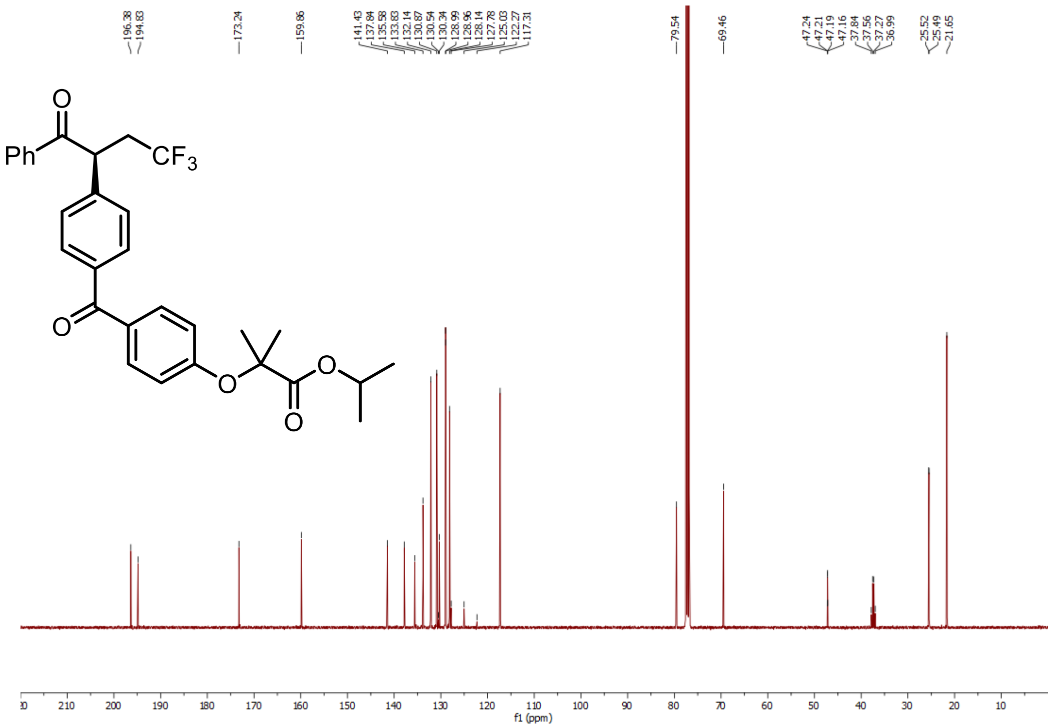

^{19}F NMR (376 MHz, Chloroform-*d*):

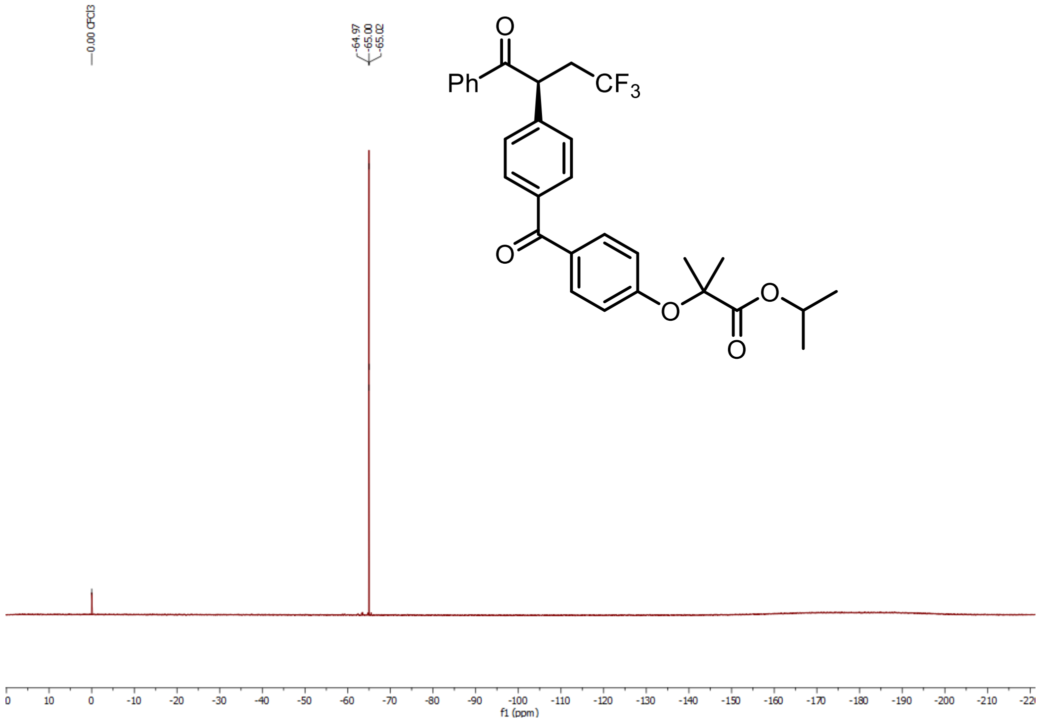

(8*R*,9*S*,13*S*,14*S*)-13-Methyl-3-((*S*)-4,4,4-trifluoro-1-oxo-1-phenylbutan-2-yl)-6,7,8,9,11,12,13,14,15,16-decahydro-17*H*-cyclopenta[a]phenanthren-17-one (6b)

^{1}H NMR (400 MHz, Chloroform-*d*):

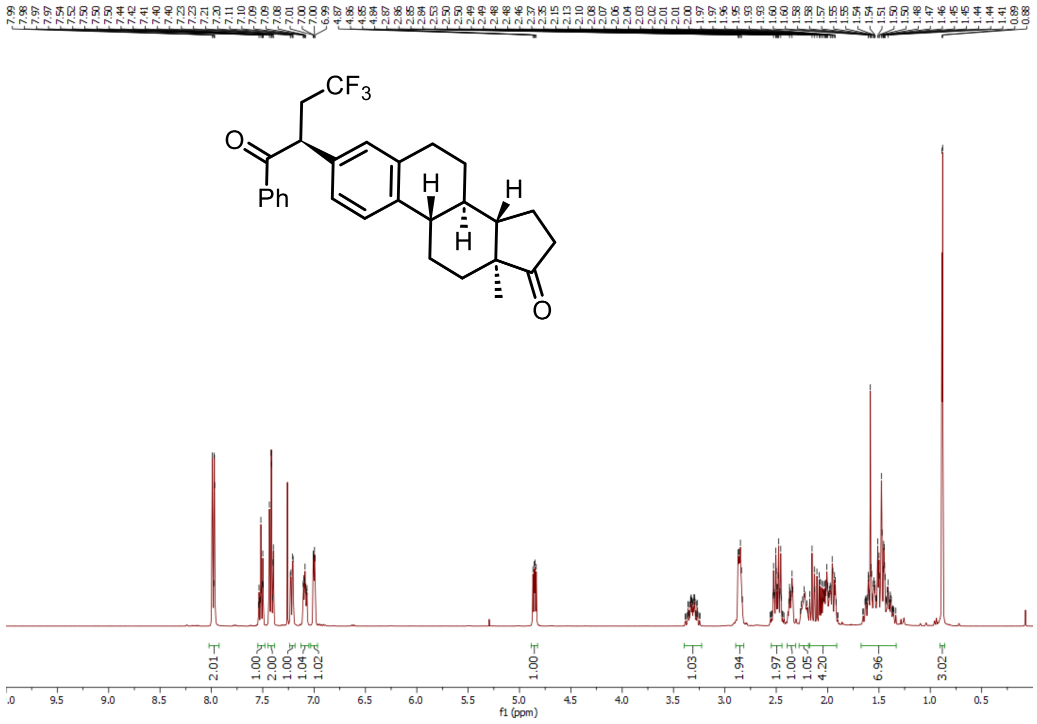

^{13}C NMR (101 MHz, Chloroform-*d*):

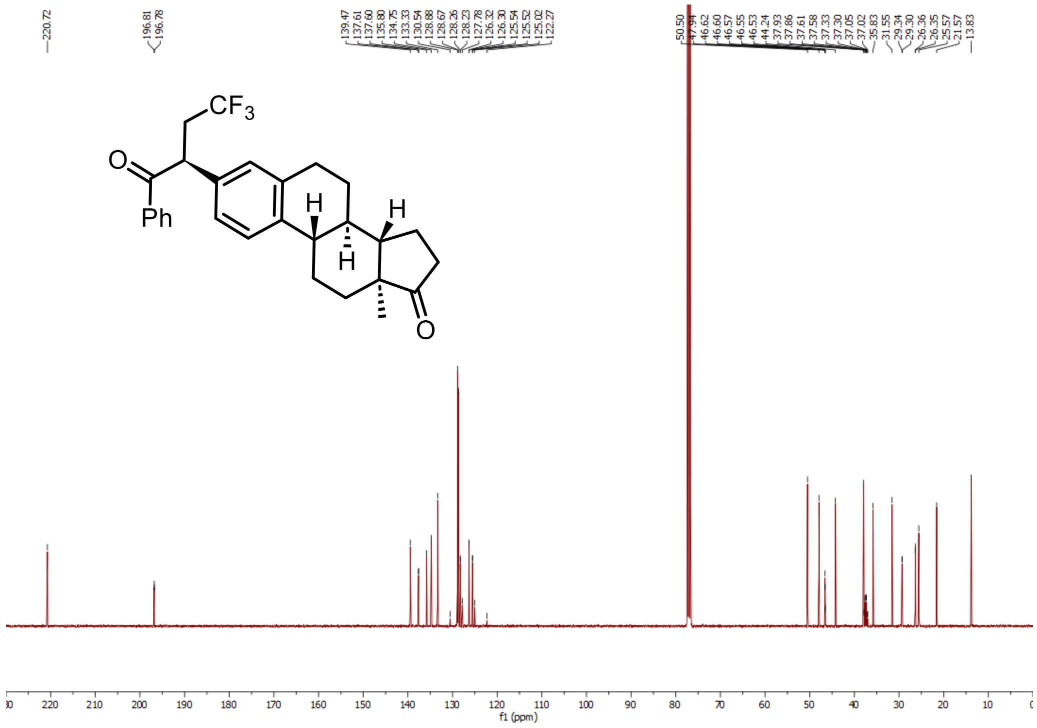

^{19}F NMR (376 MHz, Chloroform-*d*)

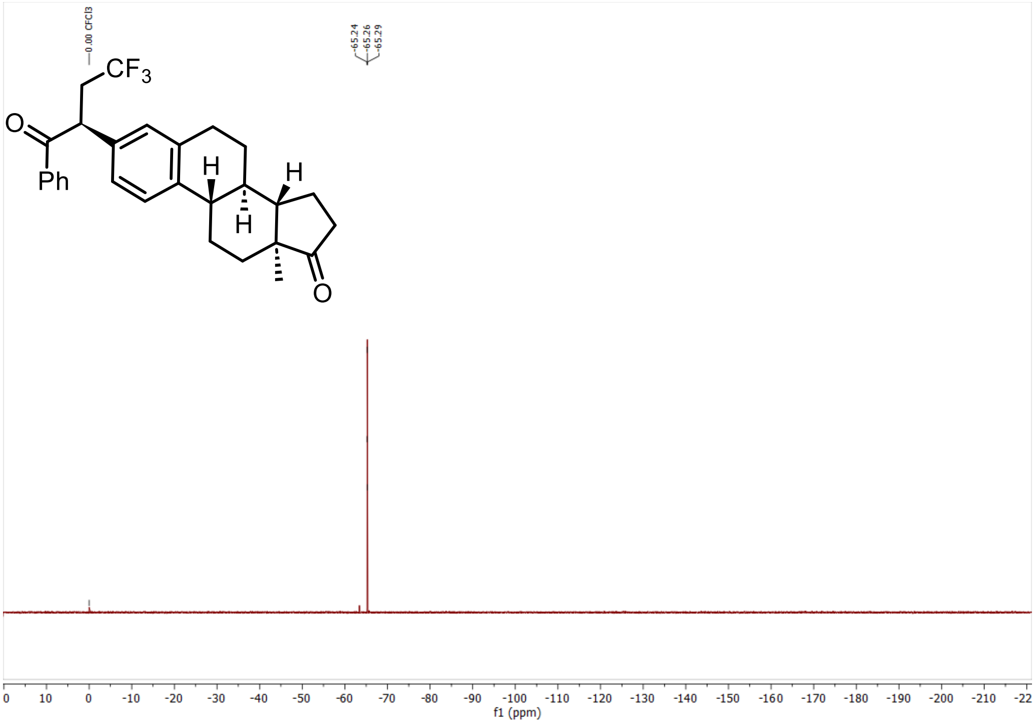

(*S*)-2-((*R*)-2,8-Dimethyl-2-((4*R*,8*R*)-4,8,12-trimethyltridecyl)chroman-6-yl)-4,4,4-trifluoro-1-phenylbutan-1-one (6c)

^{1}H NMR (400 MHz, Chloroform-*d*):

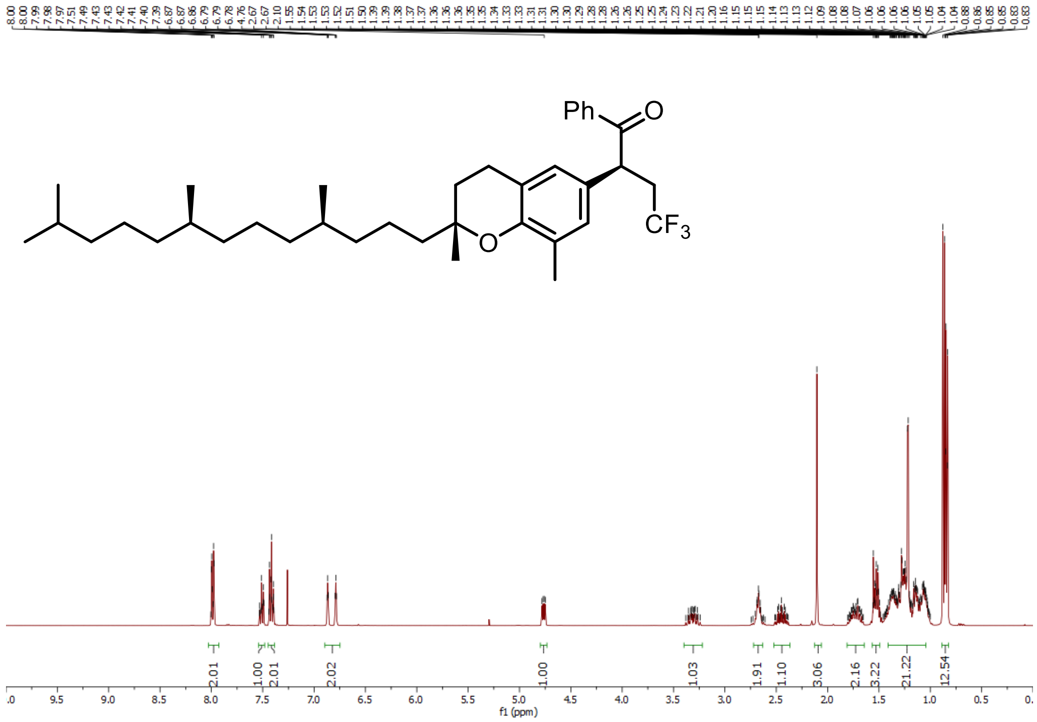

^{13}C NMR (101 MHz, Chloroform-*d*):

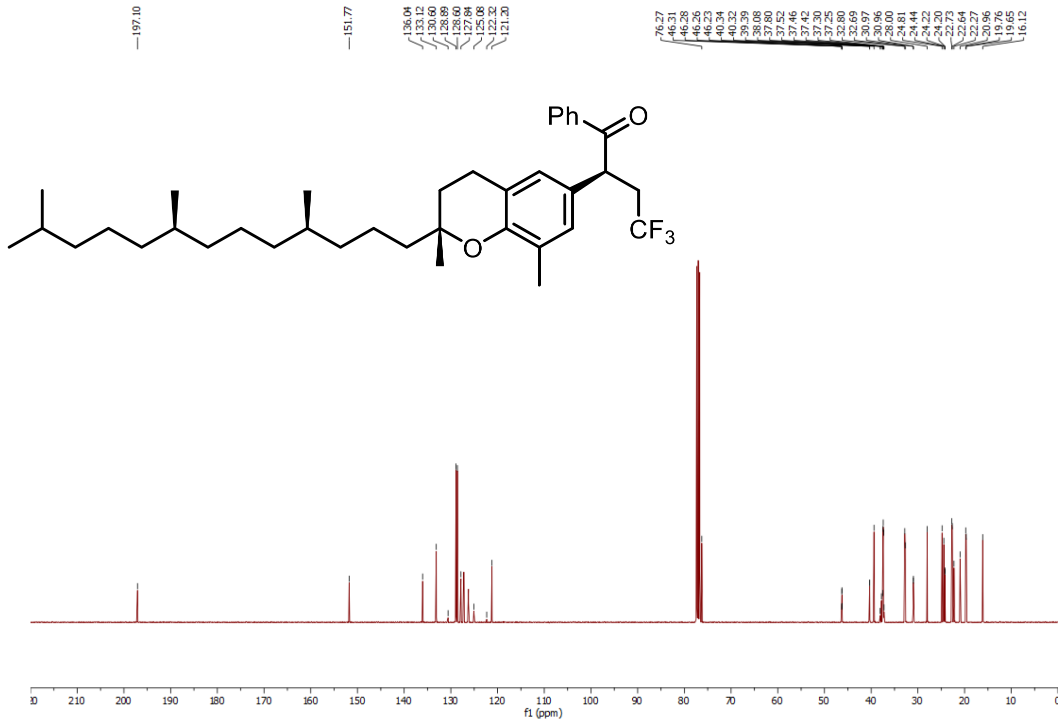

^{19}F NMR (376 MHz, Chloroform-*d*):

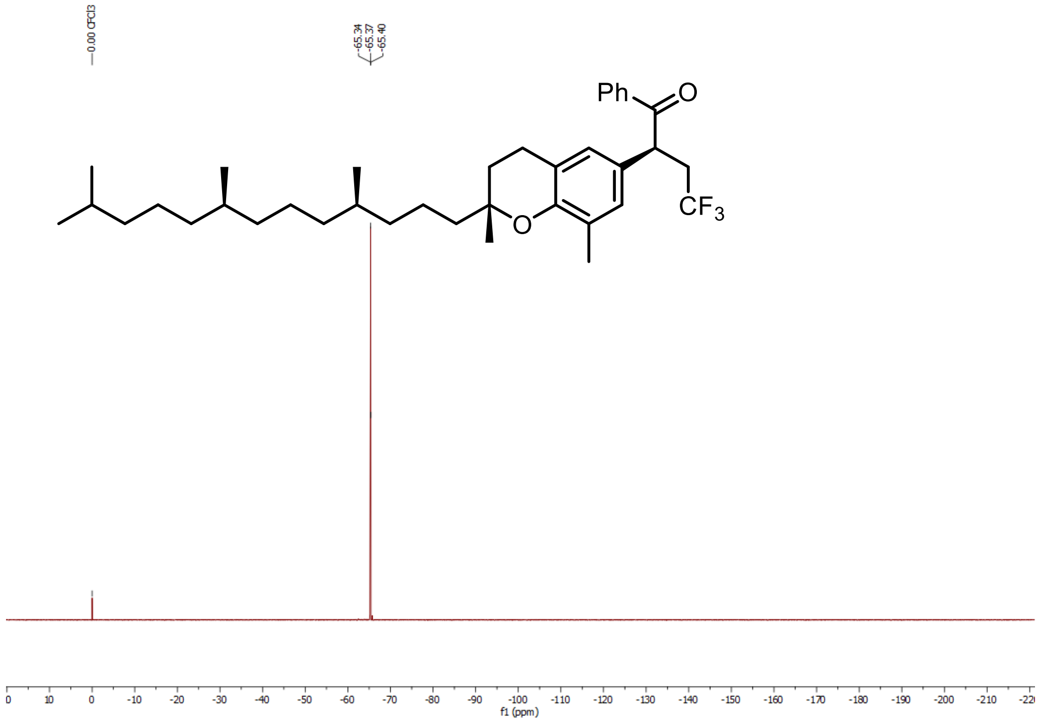

References

1. Diesel, J., Finogenova, A. M. & Cramer, N. Nickel-Catalyzed Enantioselective Pyridone C-H Functionalizations Enabled by a Bulky N- Heterocyclic Carbene Ligand. *J. Am. Chem. Soc.* **140**, 4489–4493 (2018).

2. Braconi, E. & Cramer, N. A Chiral Naphthyridine Diimine Ligand Enables Nickel-Catalyzed Asymmetric Alkylidenecyclopropanations. *Angew. Chem. Int. Ed.* **59**, 16425–16429 (2020).

3. Arienti, A. *et al.* Regioselective electrophilic alkylation of anilines with phenylacetylene in the presence of montmorillonite KSF. *Tetrahedron* **53**, 3795–3804 (1997).

4. Pesch, J., Harms, K. & Bach, T. Preparation of axially chiral N,N′-diarylimidazolium and N-arylthiazolium salts and evaluation of their catalytic potential in the benzoin and in the intramolecular stetter reactions. *Eur. J. Org. Chem.* 2025–2035 (2004).

5. Piel, I., Pawelczyk, M. D., Hirano, K., Fröhlich, R. & Glorius, F. A Family of Thiazolium Salt Derived N-Heterocyclic Carbenes (NHCs) for Organocatalysis: Synthesis, Investigation and Application in Cross-Benzoin Condensation. *Eur. J. Org. Chem.* 5475–5484 (2011).

6. Qi, X. K. *et al.* Metal-Free Amino(hetero)arylation and Aminosulfonylation of Alkenes Enabled by Photoinduced Energy Transfer. *J. Am. Chem. Soc.* **145**, 16630–16641 (2023).

7. Scheidt, F., Neufeld, J., Schäfer, M., Thiehoff, C. & Gilmour, R. Catalytic Geminal Difluorination of Styrenes for the Construction of Fluorine-rich Bioisosteres. *Org. Lett.* **20**, 8073–8076 (2018).

8. Jang, Y. J. *et al.* Green-Light-Driven Fe(III)(btz)$_3$Photocatalysis in the Radical Cationic [4+2] Cycloaddition Reaction. *Org. Lett.* **24**, 4479–4484 (2022).

9. Liebing, P. *et al.* Supramolecular Aggregation of Perfluoroorganyl Iodane Reagents in the Solid State and in Solution. *Eur. J. Org. Chem.* 3771–3781 (2018).

10. Li, J.-L. *et al.* Radical Acylfluoroalkylation of Olefins through N-Heterocyclic Carbene Organocatalysis. *Angew. Chem. Int. Ed.* **59**, 1863–1870 (2020).

11. Bourhis, L. J., Dolomanov, O. V, Gildea, R. J., Howard, J. A. K. & Puschmann, H. The anatomy of a comprehensive constrained, restrained refinement program for the modern computing environment {--} {\it Olex2} dissected. *Acta Cryst. Sect. A* **71**, 59–75 (2015).

12. Dolomanov, O. V., Bourhis, L. J., Gildea, R. J., Howard, J. A. K. & Puschmann, H. OLEX2: A complete structure solution, refinement and analysis program. *J. Appl. Cryst.* **42**, 339–341 (2009).

13. Sheldrick, G. M. SHELXT - Integrated space-group and crystal-structure determination. *Acta Crystallogr. Sect. A Found. Cryst.* **71**, 3–8 (2015).

14. CrysAlisPro Software System, Rigaku Oxford Diffraction, **2024**.

15. Pracht, P., Bohle, F. & Grimme, S. Automated exploration of the low-energy chemical space with fast quantum chemical methods. *Phys. Chem. Chem. Phys.* **22**, 7169–7192 (2020).

16. Laplaza, R., Wodrich, M. D. & Corminboeuf, C. Overcoming the Pitfalls of Computing Reaction Selectivity from Ensembles of Transition States. *J. Phys. Chem. Lett.* **15**, 7363–7370 (2024).

17. Perdew, J. P., Burke, K. & Ernzerhof, M. Generalized Gradient Approximation Made Simple. *Phys. Rev. Lett.* **77**, 3865–3868 (1996).

18. Adamo, C. & Barone, V. Toward reliable density functional methods without adjustable parameters: The PBE0 model. *J. Chem. Phys*. **110**, 6158–6170 (1998).

19. Grimme, S., Antony, J., Ehrlich, S. & Krieg, H. A consistent and accurate ab initio parametrization of density functional dispersion correction (DFT-D) for the 94 elements H-Pu. *J. Chem. Phys.* **132**, 154104 (2010).

20. Grimme, S., Ehrlich, S. & Goerigk, L. Effect of the damping function in dispersion corrected density functional theory. *J. Comput. Chem.* **32**, 1456–1465 (2011).

21. Weigend, F. & Ahlrichs, R. Balanced Basis Sets of Split Valence, Triple Zeta Valence and Quadruple Zeta Valence Quality for H to Rn: Design and Assessment of Accuracy. *Phys. Chem. Chem. Phys.* **7**, 3297–3305 (2005).

22. Gaussian 16, Revision C.01, Frisch, M. J.; Trucks, G. W.; Schlegel, H. B.; Scuseria, G. E.; Robb, M. A.; Cheeseman, J. R.; Scalmani, G.; Barone, V.; Petersson, G. A.; Nakatsuji, H.; Li, X.; Caricato, M.; Marenich, A. V.; Bloino, J.; Janesko, B. G.; Gomperts, R.; Mennucci, B.; Hratchian, H. P.; Ortiz, J. V.; Izmaylov, A. F.; Sonnenberg, J. L.; Williams-Young, D.; Ding, F.; Lipparini, F.; Egidi, F.; Goings, J.; Peng, B.; Petrone, A.; Henderson, T.; Ranasinghe, D.; Zakrzewski, V. G.; Gao, J.; Rega, N.; Zheng, G.; Liang, W.; Hada, M.; Ehara, M.; Toyota, K.; Fukuda, R.; Hasegawa, J.; Ishida, M.; Nakajima, T.; Honda, Y.; Kitao, O.; Nakai, H.; Vreven, T.; Throssell, K.; Montgomery, J. A., Jr.; Peralta, J. E.; Ogliaro, F.; Bearpark, M. J.; Heyd, J. J.; Brothers, E. N.; Kudin, K. N.; Staroverov, V. N.; Keith, T. A.; Kobayashi, R.; Normand, J.; Raghavachari, K.; Rendell, A. P.; Burant, J. C.; Iyengar, S. S.; Tomasi, J.; Cossi, M.; Millam, J. M.; Klene, M.; Adamo, C.; Cammi, R.; Ochterski, J. W.; Martin, R. L.; Morokuma, K.; Farkas, O.; Foresman, J. B.; Fox, D. J. Gaussian, Inc., Wallingford CT, 2016.

23. Marenich, A. V., Cramer, C. J. & Truhlar, D. G. Universal Solvation Model Based on Solute Electron Density and on a Continuum Model of the Solvent Defined by the Bulk Dielectric Constant and Atomic Surface Tensions. *J. Phys. Chem. B* **113**, 6378–6396 (2009).

24. Luchini, G., Alegre-Requena, J. V., Guan, Y., Funes-Ardoiz, I. & Paton, R. S. GoodVibes v3.0.1. (2019).

25. Luchini, G., Alegre-Requena, J. V., Funes-Ardoiz, I. & Paton, R. S. GoodVibes: Automated Thermochemistry for Heterogeneous Computational Chemistry Data. *F1000Research* **9**, 291 (2020).
