# Supplementary material for: Enantioselective acyl-trifluoromethylation of olefins by bulky thiazolium carbene catalysis
Source: Nat Commun. 2025 Apr 7;16:3293. doi: 10.1038/s41467-025-58423-z (PMC11977197; doi:10.1038/s41467-025-58423-z)
Supplement: Supplementary file 2 — Description of Additional Supplementary Files [file 41467_2025_58423_MOESM2_ESM.docx]

**File Name:** Supplementary Data 1

**Description:** XYZ coordinates of the global minima of NHC7’.

**File Name:** Supplementary Data 2

**Description:** XYZ coordinates of the global minimum of ketyl radical 7.

**File Name:** Supplementary Data 3

**Description:** XYZ coordinates of TS_1_ (*si-si*)

**File Name:** Supplementary Data 4

**Description:** XYZ coordinates of TS_2_ (*re-re*)

**File Name:** Supplementary Data 5

**Description:** XYZ coordinates of TS_3_ (*si-re*)

**File Name:** Supplementary Data 6

**Description:** XYZ coordinates of TS_4_ (*re-si*)
